# Electrochemical ⍺‐C─H Functionalization of Nitramines for Accessing Bifunctional Energetic Heterocycles

**DOI:** 10.1002/anie.202515252

**Published:** 2025-09-26

**Authors:** Wan‐Chen Cindy Lee, Luiz F. T. Novaes, Rojan Ali, Thomas Wirth, Song Lin

**Affiliations:** ^1^ Department of Chemistry and Chemical Biology Cornell University Ithaca NY 14853 USA; ^2^ School of Chemistry Cardiff University Cardiff CF10 3AT UK

**Keywords:** Azolation, Electrochemistry, Electroflow, Energetic compound, Nitramine

## Abstract

The synthesis of energetic materials (EMs) often involves hazardous reagents and harsh conditions, raising safety and environmental concerns. We herein present an electrochemical method for the ⍺‐C─H azolation of nitramines, enabling the integration of nitramines and various nitrogen‐rich azoles as dual energetic components within the same molecule. To enhance the practicality of the overall synthesis, we developed a tandem two‐step process that transforms free amines into nitramines using stable and readily available reagents, which was complemented by subsequent electrochemical azolation to complete a streamlined, scalable preparation of bifunctional energetic compounds. Finally, a continuous flow system was employed to further improve the practicality of the electrosynthetic method, which substantially reduced electrolyte usage and increased productivity. Computational and experimental data revealed that the introduction of azoles, particularly those with additional nitro substituents, improves the energy density and thermal stability of nitramines. This work provides a proof of concept that the reported electrochemical azolation reaction may not only offer a safer and more sustainable alternative to traditional approaches for energetic material synthesis, but it will also provide a platform for the discovery of novel compounds with favorable energetic properties.

## Graphical abstract

An electrochemical α‐C─H functionalization of nitramines enables the synthesis of molecules containing bifunctional energetic heterocycles with promising properties. A telescoped, HNO_3_‐free sequence involving nitration and azolation steps offers a safer, modular, and scalable platform for the synthesis of energetic compounds.

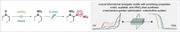

## Introduction

Energetic materials (EMs) play a vital role in applications ranging from civil construction and pyrotechnics to space exploration and defense.^[^ ^1^, ^2^, ^3^, ^4^, ^5^ ^]^ These critical applications have driven the design of numerous high‐energy organic compounds with innovative structures—often containing canonical energetic functional groups, including nitro groups, nitrogen‐rich heteroarenes, and strained rings—to achieve desired properties.^[^ ^6^, ^7^, ^8^ ^]^ Despite these advances, the synthesis of organic EMs remains predominantly reliant on traditional methodologies that often necessitate hazardous reagents and harsh reaction conditions. The requirement for corrosive and toxic oxidants such as fuming nitric acid and nitrogen oxides, as well as high temperatures and pressures, poses significant safety and environmental challenges.^[^ ^9^, ^10^, ^11^ ^]^ Discovering new strategies and methods that bypass the use of such hazardous chemicals and operate under milder conditions would not only enhance the safety and efficiency of EM synthesis but also enable access to novel energetic structures that remain inaccessible through conventional methods.

Electrochemistry provides a mild and efficient alternative to conventional methods for organic redox reactions and has recently been leveraged to improve the synthesis of known and new EMs.^[^ ^12^, ^13^, ^14^, ^15^, ^16^, ^17^, ^18^, ^19^, ^20^, ^21^ ^]^ By harnessing unique capabilities such as precise control over electrode potential and current output, we and others have shown that electrochemistry can enable previously challenging transformations with high efficiency and selectivity.^[^ ^22^, ^23^, ^24^, ^25^, ^26^, ^27^ ^]^ Moreover, using electricity to supply redox equivalents in place of traditional strong oxidants and reductants can reduce chemical hazards and environmental impact.^[^ ^28^, ^29^, ^30^, ^31^, ^32^, ^33^, ^34^, ^35^, ^36^, ^37^, ^38^, ^39^ ^]^ Thus, electrosynthesis has the potential to not only enhance the safety and practicality of EM preparation but also create opportunities to discover new functional compounds through unexplored reaction pathways.

Notwithstanding these promises, the application of electrochemistry in EM synthesis remains rare and has yet to be systematically investigated. The earliest example of the electrosynthesis of EMs was the preparation of hydroxylammonium nitrate via the reduction of nitrate anion, which required a divided electrolysis cell, excessive nitric acid, and strict temperature control.^[^ ^40^, ^41^ ^]^ Recently, Baran and coworkers applied electrochemistry to synthesize tetramethyl cyclobutane‐1,1,2,2‐tetracarboxylate, a precursor to 1,1,2,2‐tetrakis(nitroxymethyl)cyclobutane, via anodic oxidation.^[^ ^13^ ^]^ In addition, Piercey and coworkers obtained nitrogen‐rich biheteroaryls as insensitive energetic compounds by coupling pyrazines and tetrazoles through a sequential electrochemical‐photochemical process.^[^ ^15^ ^]^

**Scheme 1:**
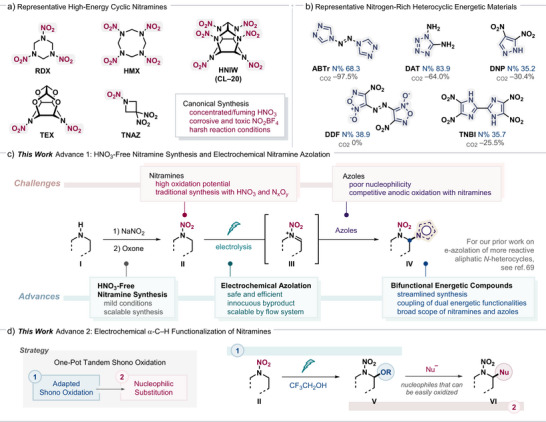
Electrochemical α‐C─H functionalization of nitramines. RDX = 1,3,5‐trinitro‐1,3,5‐triazinane. HMX = 1,3,5,7‐tetranitro‐1,3,5,7‐tetrazocane. HNIW = hexanitrohexaazaisowurtzitane. TEX = 4,10‐dinitro‐2,6,8,12‐tetraoxa‐4,10‐diazatetracyclo[5.5.0.0^5,9^.0^3,11^]‐dodecane. TNAZ = 1,3,3‐trinitroazetidine. ABTr = 4,4′‐azobis[1,2,4‐triazole]. DAT = 1,5‐diamino‐tetrazole. DNP = 3,4‐dinitropyrazole. DDF = 4,4′‐dinitro‐3,3′‐diazenofuroxan. TNBI = 4,4′,5,5′‐tetranitro‐2,2′‐biimidazole. Oxone = potassium peroxymonosulfate.

We became interested in developing a general approach for the functionalization of nitramines by means of electrochemical oxidation. Nitramines are a prominent class of EMs that are recognized for their superior energy density, thermal stability, and high detonation performance (Scheme 1a).^[^ ^42^, ^43^, ^44^ ^]^ Nevertheless, these compounds are often synthesized through nitration or nitrolysis of free or substituted amines using fuming nitric acid.^[^ ^45^, ^46^, ^47^ ^]^ Moreover, the further functionalization of nitramines into other energetic groups remains underexplored. We envision that methods that could directly substitute the α‐C─H bond will provide opportunities to conjugate nitramines with additional energetic groups, yielding diverse functional dyads with tunable structures and properties, with potential to facilitate the discovery and synthesis of novel EMs.

Following this notion, we focused our initial effort on the azolation of nitramines to forge difunctional energetic motifs. Nitrogen‐rich heterocycles such as pyrazoles, triazoles, and tetrazoles are promising energetic groups owing to the high nitrogen content, favorable energetic properties, and environmentally friendly byproducts upon detonation (Scheme 1b).^[^ ^48^, ^49^, ^50^, ^51^, ^52^, ^53^, ^54^, ^55^ ^]^ In previous work, Shreeve and Klapötke independently advanced chemical azolation methods to synthesize acyclic nitramines with α‐heteroaryl groups via nucleophilic substitution. Both protocols use pre‐functionalized nitramines with an α‐leaving group (─Cl) in addition to potassium or ammonium azole salts as nucleophiles.^[^ ^56^, ^57^, ^58^, ^59^ ^]^ While the resultant products show promising properties with respect to the heat of formation, thermal stability, crystal density, and detonation pressure and velocity, the scope of these synthetic methods remains underexplored, as only a total of two chlorinated nitramines have thus far been investigated as substrates. In general, no methods are currently available to directly substitute an α‐C─H bond of nitramines with azoles.

Toward achieving the aforementioned synthetic strategy, we were inspired by the venerable Shono oxidation reaction^[^ ^60^, ^61^, ^62^, ^63^, ^64^, ^65^, ^66^ ^]^ and envisioned that an electrochemical method leveraging an analogous mechanism could help achieve the desired transformation of nitramines, activating the α‐C─H bond for further azolation. Specifically, the initial anodic oxidation of a nitramine will give rise to a highly electrophilic *N*‐nitroiminium ion intermediate (**III**), which is expected to undergo nucleophilic attack by a suitable *N*‐heteroarene to yield α‐C─H azolated product **IV** with two distinct neighboring energetic substituents (Scheme 1c). Alternatively, if an alcohol is used as the nucleophile like in the canonical Shono oxidation, the newly installed leaving group (**V**) will allow for additional derivatizations via nucleophilic substitution to introduce diverse functionalities (**VI**) (Scheme 1d). Related to this proposed transformation, Wang, Lei, and our laboratory have independently disclosed electrochemical methods for the α‐C─H azolation of *N*‐substituted amines such as lactams and carbamates,^[^ ^67^, ^68^, ^69^, ^70^, ^71^, ^72^, ^73^, ^74^ ^]^ but similar transformations for nitramines remain unknown. While in principle, α‐azolated carbamates could be converted to the nitramine derivatives via sequential *N*‐deprotection and ‐nitration, the free amine intermediates are unstable, as azoles are good leaving groups, particularly under strongly acidic conditions that are commonly used for nitration.^[^ ^75^ ^]^

Against this backdrop, we herein report the development of an electrochemical method for the direct oxidative α‐C─H azolation of nitramines to access diverse bifunctional energetic units.^[^ ^76^ ^]^ This transformation has not been reported previously, and we expect that it would be challenging to achieve using traditional chemical oxidation due to the high potential needed to oxidize nitramines. By substituting azoles with trifluoroethanol as the nucleophile, we also obtained synthetic intermediates that could undergo further derivatization to introduce various secondary energetic functionalities. To further improve the practicality of the synthetic strategy, we developed a mild, nitric acid‐free protocol for nitramine synthesis. These developments ultimately enabled a telescoped, gram‐scale synthesis of azolated nitramines from readily available amine hydrogen chloride salts.

## Results and Discussion

### Improved Nitramine Preparation Under Nitric‐Acid‐Free Conditions

**Scheme 2:**
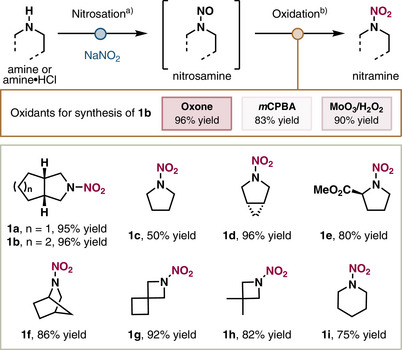
Nitramine synthesis under nitric‐acid‐free conditions. ^a)^With NaNO_2_ (3 equiv), HCl (2.2 equiv) in dichloromethane at 0 °C for 1 h. ^b)^With Oxone (2 equiv), in acetonitrile/H_2_O (1:1.5) at 40 °C for 16 h.

The synthesis of nitramine substrates remains traditionally reliant on the nitration of free amines or nitrolysis of *N*‐substituted amines; both approaches use fuming nitric acid.^[^ ^45^, ^46^, ^47^ ^]^ Additional methods employ strong and potentially hazardous oxidizers such as N_2_O_5_^[^ ^77^, ^78^, ^79^ ^]^ or (diacetoxyiodo)benzene, the latter affording a mixture of nitrosamine and nitramine products.^[^ ^80^, ^81^ ^]^ To improve the practicality of nitramine synthesis, we developed a two‐step, telescoped procedure to access nitramines under mild conditions with inexpensive and easy‐to‐handle feedstock reagents. This tandem process involves first a previously reported protocol that converts a secondary amine to a nitrosamine using industrial commodity NaNO_2_ salt^[^ ^81^, ^82^, ^83^ ^]^ followed by a newly developed oxidation reaction that transforms the nitrosamine into a desired nitramine, without chromatographical purification of the intermediate (only aqueous washes needed). For the second step, we screened various oxygen‐atom donors and found that *m*CPBA, MoO_3_/H_2_O_2_, and Oxone can all serve as effective oxidants (Scheme 2 and Table S7). We chose to use stable, nontoxic, and inexpensive Oxone^[^ ^84^ ^]^ to maximize scalability and practicality. We note that both steps could also be achieved using electrochemistry in batch or flow reactors following recently developed protocols.^[^ ^85^, ^86^, ^87^, ^88^ ^]^

Using this tandem protocol, we synthesized a panel of nitramines from secondary amines or their corresponding HCl salts (Scheme 2). Pyrrolidine substrates, including bicyclic frameworks and ester‐containing derivatives, were efficiently converted to nitramines **1a**–**1f** in high yields (80%–96%). The relatively low yield (50%) observed for **1c** is attributed to the high volatility of the nitrosamine intermediate. Additionally, azetidine derivatives and piperidine also proved to be suitable substrates, affording nitramines **1g**–**1i** in 75%–92% yield under standard conditions.

### Discovery and Optimization of Electrochemical C─H Azolation

**Scheme 3:**
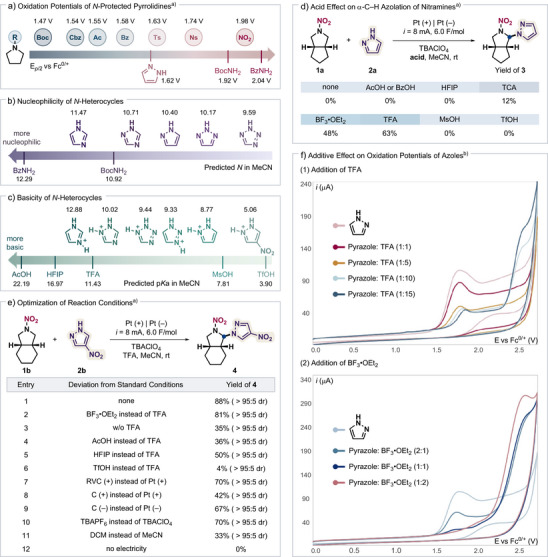
Development and optimization of reaction conditions for the azolation of nitramines. ^a)^Conducted with **1** (0.25 mmol), **2** (2 equiv), electrolyte (0.4 M) in presence of TFA (10 equiv) or BF_3_·OEt_2_ (2 equiv) in solvent (2.5 mL) at room temperature; constant current *i* = 8 mA, 6 F mol^−1^, 5 h; undivided cell (ElectraSyn 2.0); isolated yields; diastereomeric ratio (dr) determined by ^1^H NMR analysis of crude mixture. ^b)^Working electrode: glassy carbon disk; counter electrode: Pt wire; pyrazole (10 mM); solvent: MeCN; electrolyte: TBAClO_4_ (0.1 M); *υ* = 100 mV s^−1^. Boc = *tert*‐butoxycarbonyl. Cbz = benzyloxycarbonyl. Ac = acetyl. Bz = benzoyl. Ts = *p*‐toluenesulfonyl. Ns = 2‐nitrobenzenesulfonyl. TBA = tetrabutylammonium. MsOH = methanesulfonic acid. TCA = trichloroacetic acid. TFA = trifluoroacetic acid. BzOH = benzoic acid. AcOH = acetic acid. HFIP = hexafluoroisopropanol. TfOH = trifluoromethanesulfonic acid. RVC = reticulated vitreous carbon.

The Shono‐type oxidation has recently been adapted to the formation of C─N bonds from various protected amines such as amides, sulfonamides, and carbamates, in combination with *N*‐nucleophiles such as BocNH_2_ and BzNH_2_.^[^ ^68^, ^89^ ^]^ Compared to these existing systems, we envisioned several challenges that are inherent to the proposed nitramine functionalization outlined in the Introduction section. First, the high potentials required to oxidize nitramines and the comparatively low oxidation potentials of *N*‐rich heteroarenes make it difficult to selectively activate the former. Indeed, cyclic voltammetry (CV) data showed that pyrrolidines with various *N*‐protecting groups, including carbamate (Boc, Cbz), amide (Ac, Bz), and sulfonamide (Ts, Ns), require significantly lower potentials for oxidation than *N*‐nitropyrrolidine (Figures S3, S4 and Scheme 3a). Importantly, unlike oxidatively resistant nucleophiles such as BocNH_2_ and BzNH_2_, *N*‐heteroarenes like pyrazole are also oxidized at substantially less positive potentials than *N*‐nitropyrrolidine. Additionally, *N*‐rich heteroarenes are generally poorer nucleophile vis‐à‐vis BocNH_2_ and BzNH_2_, further complicating the envisioned strategy (Scheme 3b).^[^ ^90^ ^]^

We first chose to study the electrochemical coupling of nitramine **1a** and pyrazole **2a** to furnish bifunctional product **3**. To achieve chemoselective oxidation of **1a** in the presence of pyrazole, we surveyed a panel of acids featuring varying p*K*_a_ values as transient nucleophile protecting agents (Schemes 3c and 3d; Table S3),^[^ ^91^ ^]^ aiming at balancing deactivation of the azole toward oxidation while maintaining sufficient nucleophilicity. Indeed, it was found that the addition of trifluoroacetic acid (TFA) or Lewis acid BF_3_·OEt_2_ to the reaction provided the desired product in optimal yields (48%–63%). The former has a p*K*_a_ that is about 2.7 units larger than the conjugate acid of pyrazole in MeCN, and is thus likely to protect the nucleophile via H‐bonding rather than protonation. CV data showed that upon addition of these acids to pyrazole, both a decrease in peak intensity and an anodic shift of the peak potential were observed, and the magnitude of these changes increases with increasing acid concentration (Scheme 3f). As expected, weaker acids cannot effectively protect pyrazole from oxidation and therefore provide no to low yield, whereas methanesulfonic acid and trifluoromethanesulfonic acid with p*K*_a_’s that are lower than pyrazolium ion inhibited the reaction likely due to strong nucleophile deactivation.

In a separate model reaction, we also investigated the α‐C─H azolation of *N*‐nitramine **1b** using nitrated pyrazole **2b**. This nucleophile is attractive from an energetics perspective as it features an additional nitro group, but it is simultaneously less reactive. Nevertheless, the desired product **4** was obtained in excellent yield under previously optimized conditions using either TFA or BF_3_·OEt_2_ as an acid additive (Scheme 3e, entries 1 and 2, and Table S2). We attribute this high reactivity to the strong electrophilicity of the *N*‐nitroiminium ion intermediate, which makes it possible for weak nucleophiles like **2b** to participate in the addition reaction. Because **2b** is significantly more difficult to oxidize than **2a** (*E*_p/2_ = +2.32 V, which is higher than that of *N*‐nitropyrrolidine, *E*_p/2_ = +1.98 V), we hypothesized that the use of a protecting acid might not be necessary. Indeed, conditions without an acid or with a weak acid (e.g., AcOH or HFIP) still led to the formation of **4** albeit in diminished yields (35%–50% yield; entries 3–5), in contrast to the reaction with unsubstituted pyrazole **2a** (Scheme 3d). Thus, in the reaction between **1b** and **2b**, the acid likely participates as a proton source for the cathodic hydrogen evolution reaction rather than a protecting agent for the azole nucleophile. Nonetheless, the introduction of a super acid, TfOH, nearly completely inhibited the reaction due to nucleophile deactivation (entry 6).

Several sets of additional control experiments were carried out. Changing the anode material from Pt to RVC or graphite led to diminished yields of 70% and 42%, respectively (entries 7 and 8). Switching the Pt cathode with graphite also resulted in a lower yield (67%), likely due to the reduced efficiency of the hydrogen evolution counter reaction (entry 9). Using TBAPF_6_ as the electrolyte maintained high product yield (entry 10), whereas changing the solvent to dichloromethane inhibited the reaction (entry 11). As expected, a control experiment confirmed the necessity of electricity for the C─H azolation reaction, as no product was formed in the absence of an applied current (entry 12).

### Substrate Scope

**Scheme 4:**
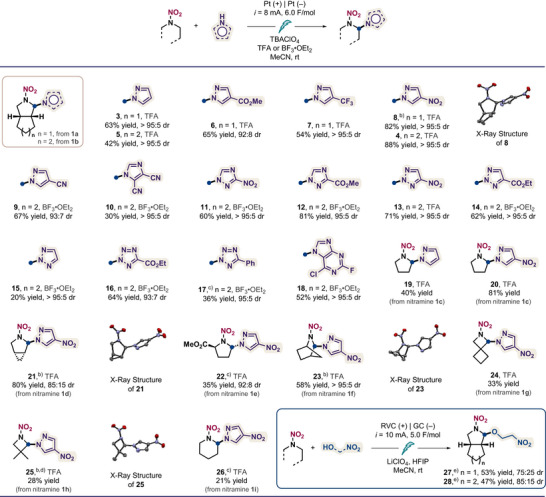
Scope for electrochemical α‐C─H azolation of nitramines. ^a)^Conducted with a 0.25 mmol scale with azole (2 equiv), TBAClO_4_ (0.4 M) in presence of TFA (0.2 mL) or BF_3_·OEt_2_ (2 equiv) in acetonitrile (2.5 mL) at room temperature; constant current *i* = 8 mA, 6 F mol^−1^, 5 h; platinum wire; undivided cell (ElectraSyn 2.0); isolated yields; diastereomeric ratio (dr) determined by ^1^H NMR analysis of crude mixture. ^b)^Relative configuration determined by X‐ray crystallography. ^c)^Yields determined by ^1^H NMR spectroscopy. ^d)^With LiClO_4_ (0.4 M). ^e)^Conducted with a 0.25 mmol scale with alcohol (2 equiv), LiClO_4_ (0.4 M) in the presence of HFIP (0.4 mL) in acetonitrile (2.1 mL) at room temperature; constant current *i* = 10 mA, 5 F mol^−1^, 3 h and 21 min; RVC anode and glassy carbon (GC) cathode; undivided cell (ElectraSyn 2.0); isolated yields.

Under the optimal conditions, we first explored the scope of azoles in the electrochemical functionalization of nitramines (Scheme 4). In addition to pyrazoles, we examined other nitrogen‐rich heteroarenes including imidazoles, triazoles, and tetrazoles, particularly those bearing electron‐withdrawing groups such as ester, trifluoromethyl, cyano, and nitro. These functionalities are often present in energetic molecules or their synthetic precursors, as they can be used to tune key energetic parameters, including oxygen balance and nitrogen content, or to provide sites for additional functionalization and structural modification. We found that depending on the azole, using different acid additives (TFA or BF_3_·OEt_2_) can significantly influence the reaction yield (Tables S3–S5). Parent and substituted pyrazoles underwent the desired transformation, furnishing azolated products **3**–**9** in good to excellent yields. 4,5‐Dicyanoimidazole was tolerated in this system, providing the corresponding product **10** in 30% yield. Both 1,2,3‐ and 1,2,4‐triazoles, including those containing nitro and ester groups, were converted to the desired bifunctional compounds (**11**–**15**) in useful yields (20%–81%). Importantly, this electrochemical reaction also accommodated tetrazoles, which are typically less nucleophilic than those with fewer ring nitrogen atoms, affording compounds **16** and **17** in promising yields. Additionally, 6‐chloro‐2‐fluoropurine was found to be a viable nucleophile, giving compound **18** in 52% yield. We note that in all cases, the azole selectively added to the convex face of the *N*‐nitroiminium ion intermediate, giving 92:8 or higher diastereomeric ratio. While we chose to study model substrates in this work, it may be helpful to know that the stereochemical outcome of this reaction is predominantly governed by intrinsic steric interactions during nucleophilic addition, as it was shown that the relative stereochemistry of energetic compounds can significantly affect their properties.^[^ ^13^ ^]^

We next examined nitramines with distinct carbon skeletons and found that in addition to parent *N*‐nitropyrrolidine, a variety of its analogues, such as strained bicyclic nitramines with fused (**1d**), bridged (**1f**), and spiro (**1g**) ring constructions produced azolated products (**21**, **23**, and **24**) in good to high yields. Of note, *N*‐nitropyrrolidine with an electron‐deficient ester group at the α‐carbon (**1e**), which we chose as a safer surrogate for 1,2‐dinitropyrrolidine with a similar electronic environment around the reactive site, reacted with 4‐nitropyrazole to furnish compound **22** in a synthetically useful 35% yield. Importantly, the azolation of substrates containing *N*‐nitroazetidine (**1g** and **1h**) and *N*‐nitropiperidine (**1i**) cores was successful giving products **24**–**26**, implicating that our method may be applied to the functionalization of structurally similar energetic compounds such as TNAZ and RDX (Scheme 1). The structures and relative stereochemistry (if applicable) of azolated nitramines **8**, **21**, **23**, and **25** were confirmed by X‐ray crystallography. Beyond azoles, we also evaluated 2‐nitroethanol as a nucleophile, which introduces an additional C‐substituted nitro group. The corresponding products **27** and **28** were obtained in good yields under similar conditions with slightly decreased diastereoselectivity owing to the smaller size of the nucleophile.

**Scheme 5:**
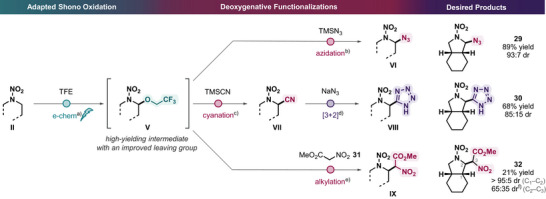
α‐C─H functionalizations via tandem Shono oxidation for the introduction of energetic functionalities. ^a)^With TBAClO_4_ (0.4 M) in TFE at room temperature; constant current *i* = 5 mA, 2.5 F mol^−1^, 3 h and 21 min; RVC anode and Pt foil cathode; undivided cell (ElectraSyn 2.0). ^b)^With TMSN_3_ (3 equiv), BF_3_·OEt_2_ (3 equiv) in MeCN at room temperature. ^c)^With TMSCN (3 equiv), BF_3_·OEt_2_ (3 equiv) in MeCN at room temperature. ^d)^With NaN_3_ (3 equiv), NH_4_Cl (3 equiv) in DMF at 100 °C. ^e)^With nitroacetate (3 equiv), AlCl_3_ (3 equiv) in DCM at room temperature. Diastereomeric ratio (dr) determined by ^1^H NMR analysis of crude mixture. ^f)^The isolated compound (65:35 dr) underwent isomerization in chloroform‐*d*, increasing the dr to 92:8. The relative configuration at C3 could not be confidently assigned.

To further enhance the utility of this synthetic method, we aimed to develop a strategy to expand the nucleophile scope beyond azoles (Scheme 5). In particular, various pertinent nucleophiles in energetics chemistry exhibit significantly lower oxidation potentials than nitramines, but some of them are not amenable to the acid protection approach because they are either poorly basic (e.g., α‐nitroacetate **31**) or yield hazardous species upon protonation (e.g., CN^−^, N_3_^−^). To address this challenge, we employed a two‐step, telescoped protocol that decouples the electrochemical oxidation and nucleophilic substitution processes, thereby allowing for improved flexibility in nucleophile selection. Specifically, a Shono‐type oxidation of a nitramine substrate **II** gives rise to *N*,*O*‐acetal intermediates **V**, which undergo nucleophilic substitution in a subsequent, nonelectrochemical step. While the classical Shono oxidation typically employs methanol as the nucleophile,^[^ ^60^, ^61^ ^]^ we previously showed that modified conditions using trifluoroethanol (TFE) as both the solvent and nucleophile can substantially enhance the utility of the method.^[^ ^92^, ^93^ ^]^ Because TFE is more oxidatively resistant than methanol, the modified conditions can be used to activate amines with strongly electron‐withdrawing *N*‐substituents without competing oxidation of the nucleophile (i.e., oxidation of MeOH). Furthermore, this approach delivers the *N*,*O*‐acetal (**V**) with a better leaving group (─OCH_2_CF_3_) to enhance the efficiency of the following substitution step (for reaction optimization, see Table S6).

This approach indeed proved to be effective in the envisioned synthetic route to construct diverse energetic functional dyads. By directly subjecting intermediate **V** to nucleophilic substitution without purification using trimethylsilyl azide (TMSN_3_) in the presence of Lewis acid BF_3_·OEt_2_, the nitrogen‐rich ⍺‐azidonitramine **29** was obtained in 89% yield. Analogously, using trimethylsilyl cyanide (TMSCN) as a nucleophile led to the formation of ⍺‐cyanonitramine **Vll**, which was further transformed into the tetrazole‐containing product **30** via [3 + 2] cycloaddition with NaN_3_ in an overall 68% yield. Notably, such type of products are constitutional isomers of nitramines obtained through direct electrochemical functionalization (e.g., **16** and **17**) with a newly formed C─C rather than C─N bond. Finally, nitroacetate **31** appeared to be a compatible nucleophile in the presence of AlCl_3_, giving rise to trifunctional molecule **32** in 21% yield.

### Telescoped Synthesis

**Scheme 6:**
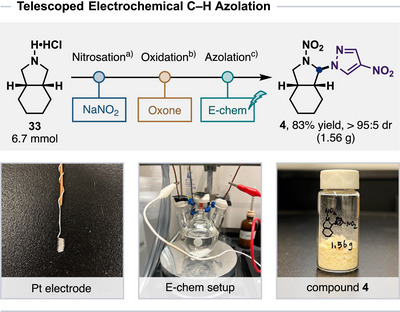
Telescoped reaction for scalable synthesis. ^a)^With NaNO_2_ (3 equiv), HCl (2.2 equiv) in dichloromethane at 0 °C for 1 h. ^b)^With Oxone (2 equiv), in acetonitrile/H_2_O (1:1.5) at 40 °C for 16 h. ^c)^With azole (2 equiv), TBAClO_4_ (0.4 M) in the presence of TFA (10 equiv) in acetonitrile at room temperature; Constant current *i* = 8 mA, 6 F mol^−1^, 5 h; platinum wire; undivided cell; isolated yield.

This electrochemical approach allowed us to further develop a telescoped, gram‐scale synthesis of ⍺‐azolated nitramines (Scheme 6). Starting from 6.7 mmol of ammonium salt **33** as the substrate, the three‐step process delivered 1.56 g of azolated product **4** as a pale yellow solid in 83% overall yield without requiring chromatographical purification of intermediates. We anticipate that this telescoped procedure will provide a robust, scalable, and practical process for the synthesis of new EMs featuring both nitramine and *N*‐rich azole explosophores.

### Continuous Synthesis Using Electroflow

**Scheme 7:**
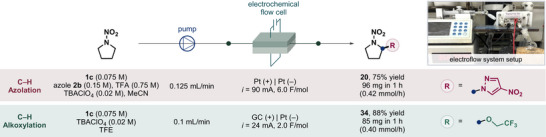
Electroflow system for the synthesis of functionalized nitramines.

Flow electrochemistry has emerged as a promising strategy for scalable electrosynthesis^[^ ^94^, ^95^, ^96^, ^97^, ^98^ ^]^ and has recently been employed in the preparation of active pharmaceutical ingredients on ∼200 kg scale for clinical trials.^[^ ^99^ ^]^ The high electrode surface area‐to‐volume ratio in flow reactors enhances mass transport, thus maximizing contact between the bulk solution and the electrode surface. The small interelectrode distance in many electroflow reactors reduces the Ohmic drop, which allows for reduced use or even elimination of supporting electrolytes.^[^ ^96^ ^]^ Mindful of these benefits, we conducted electrochemical nitramine functionalization under constant current conditions in an undivided‐cell flow reactor (Scheme 7).^[^ ^85^, ^100^, ^101^ ^]^

Using a 125 µm fluorinated ethylene propylene spacer to separate the electrodes, the reactor features a channel volume of 0.15 mL, with an active electrode surface area of 12 cm^2^ per electrode. With this setup, the use of supporting electrolyte was reduced by a factor of 20 under the optimized conditions (Tables S8–S11). Azolated nitramine **20** was generated at a rate of 0.42 mmol h^−1^ (compared to 0.04 mmol h^−1^ in batch), and alkoxylated nitramine **34** was produced at 0.40 mmol h^−1^ (compared to 0.06 mmol h^−1^ in batch). The two products were formed in 75% and 88% yield, respectively, after a single pass of the reaction solution through the flow reactor. These results show that our method could be adapted in a continuous electroflow setting with superior efficiency. Moreover, the flow process affords higher space‐time yield (STY) and reduced electrolyte usage compared to batch reactions (Section 9.5 in Supporting Information), indicating improved scalability and cost‐efficiency for potential larger‐scale applications. While not attempted in this work, we anticipate that with appropriate device and condition optimization, this system may be used to synthesize energetic compounds on preparative scales with improved safety and practicality.^[^ ^102^ ^]^

### Computational and Experimental Physical Properties

**Scheme 8:**
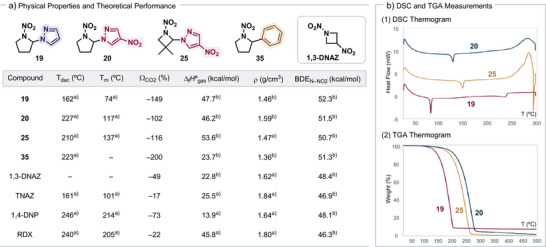
Comparison of physical properties across nitramine derivatives. ^a)^Based on experimental measurements. ^b)^Based on computation. *T*_dec_ = onset temperature of decomposition. *T*_m_ = onset temperature of melting. *Ω*_CO2_ = CO_2_ oxygen balance.^[^ ^103^ ^]^ Δ_f_ *H*°_gas_ = gas‐phase heat of formation.^[^ ^104^ ^]^ *ρ* = crystal density.^[^ ^105^ ^]^ BDE = bond‐dissociation energy. 1,3‐DNAZ = 1,3‐dinitroazetidine. TNAZ = 1,3,3‐trinitroazetidine. 1,4‐DNP = 1,4‐dinitropiperazine. RDX = 1,3,5‐trinitro‐1,3,5‐triazinane. DSC = differential scanning calorimetry. TGA = thermal gravimetric analysis.

To understand how incorporating azoles into nitramine compounds may influence their energetic properties, we performed density functional theory (DFT) calculations and thermal analysis, including differential scanning calorimetry (DSC) and thermogravimetric analysis (TGA), on selected products **19**, **20**, and **25**. These data are compared with properties of mono‐functional, phenyl‐substituted nitramine **35** and well‐established EMs such as TNAZ and RDX (Scheme 8).^[^ ^106^, ^107^, ^108^, ^109^ ^]^ The DSC and TGA thermograms measured the onset temperatures for both melting and decomposition of azolated nitramines (Figures S6–S14), providing information on the melt‐castable property, an useful feature for EMs.^[^ ^110^ ^]^ While nitramine **19** shows a CO_2_ oxygen balance (*Ω*_CO2_) that is well below −100%, suggesting limited detonation potential, compound **20**, which contains an additional nitro group compared with **19**, improves the CO_2_ oxygen balance to −102%, approaching that of insensitive high explosives like 2,4‐dinitroanisole (DNAN, *Ω*_CO2_ = −97%). We also found that the introduction of an additional nitro group enhances the crystal density of **20** by nearly 10%, reaching 1.59 g cm^−3^ that is comparable to 1,3‐DNAZ and 1,4‐DNP. In general, bifunctional compounds **19**, **20**, and **25** show higher crystal density than simple nitramine **35**. According to DFT calculations,^[^ ^111^ ^]^ nitramines **19**, **20**, and **25** exhibit relatively high heat of formation (Δ_f_ *H*°_gas_) in the range of 46–53 kcal mol^−1^, similar to RDX and significantly higher than TNAZ, which shows promising levels of energy densities. In contrast, replacing the azole group in **19** and **20** with a phenyl group (**35**) lead to significant reduction in energy (Δ_f_ *H*°_gas_ = 23.7 kcal mol^−1^). These findings clearly showed that the introduction of an *N*‐rich heteroarene adjacent to the nitramine can enhance its energetic properties. Furthermore, introducing an electron‐withdrawing nitro substituent on the pyrazole ring increases the decomposition temperature (compound **19** versus compound **20**), indicating improved thermal stability.

As discussed previously, we chose to study carbon‐rich nitramines as model systems as they are easier to access and safer to handle in conventional organic chemistry laboratories. While these model compounds are unlikely to serve as candidates for energetic compounds themselves, our work provides proof of concept that the presence of adjacent functional dyads of nitramines and azoles—both of which are classical explosophores but have rarely been coupled in EM design—can substantially improve pertinent energetic properties, thus offering a new avenue for the discovery and synthesis of novel energetic compounds.

## Conclusion

In summary, we present an electrochemical approach for the ⍺‐C─H azolation of nitramines, facilitating the integration of two common energetic functionalities and resulting in products with enhanced energetic properties such as thermal stability and energy density. A wide variety of azoles, including pyrazoles, triazoles, tetrazoles, and purines, were readily incorporated into nitramine scaffolds. Additionally, we advanced a two‐step procedure that allowed for the installation of diverse energetic functionalities beyond azoles, such as azide, cyanide, and nitroacetates, onto nitramines. Finally, we developed a telescoped, three‐step process for the gram‐scale preparation of azolated nitramines under mild, HNO_3_‐free conditions via tandem chemical and electrochemical oxidation. These methodology and process advances offer a new platform for the design and synthesis of nitrogen‐rich energetic compounds with potential applications in various industrial sectors.

## Conflict of Interests

The authors declare no conflict of interest.

## Supporting information



Supporting Information

## Data Availability

The data that support the findings of this study are available in the Supporting Information of this article.
