# Supplementary material for: Electrochemical ⍺‐C─H Functionalization of Nitramines for Accessing Bifunctional Energetic Heterocycles
Source: Angew Chem Int Ed Engl. 2025 Sep 26;64(47):e202515252. doi: 10.1002/anie.202515252 (PMC12624331; doi:10.1002/anie.202515252)

Supporting Information

Electrochemical α-C–H Functionalization of Nitramines for Accessing Bifunctional Energetic Heterocycles

Wan-Chen Cindy Lee,[1] Luiz F. T. Novaes,[†,1] Rojan Ali,[†,2]
Thomas Wirth,[*,2] and Song Lin[*,1]

[1]Department of Chemistry and Chemical Biology, Cornell University, Ithaca, New York 14850, US

[2]School of Chemistry, Cardiff University, Cardiff CF10 3AT, UK

[†]Authors contributed equally

Table of Contents

1. General Information **S3**

2. Optimization of Reaction Conditions **S4**

2.1. Optimization of Electrochemical C–H Azolation S4

2.2. Optimization of Electrochemical C–H Alkoxylation S8

2.3. Optimization of Nitramine Synthesis S8

3. Reaction Setup **S9**

3.1. Experimental Setup for Electrochemical C–H Azolation S9

4. Synthesis of Nitramines **S10**

4.1. General Procedure for Preparation of Nitramines S10

5. Synthesis of Azolated Nitramines **S13**

5.1. General Procedure for Electrochemical C–H Azolation S13

6. Synthesis of Alkoxylated Nitramines **S20**

6.1. General Procedure for Electrochemical C–H Alkoxylation S20

7. Synthesis of Nitramines Containing Energetic Functionalities **S22**

7.1. General Procedures for Electrochemical C–H Functionalizations S22

8. Cyclic Voltammetry Analysis **S26**

9. Electrochemical Flow System **S30**

9.1. General Procedures for Electroflow C–H Functionalizations S30

9.2. Optimization of Electroflow C–H Azolation S31

9.3. Optimization of Electroflow C–H Alkoxylation S32

9.4. Experimental Setup for Electroflow System S33

9.5. Batch vs. Flow Processes S34

10. Measurements of Thermal Properties **S35**

10.1. DSC Thermograms S35

10.2. TGA Thermograms S37

11. X-ray Crystallography **S40**

12. DFT Calculations **S45**

13. References **S46**

14. NMR Spectra **S46**

1. General Information

All reactions were performed in anhydrous solvents. Flash chromatography was performed using silica gel 60 (230-400 mesh) from SiliCycle. Thin layer chromatography (TLC) experiments were performed on aluminum sheets coated with silica gel 60 F_{254}. Commercial reagents were purchased from Sigma Aldrich, Alfa Aesar, Acros, TCI, AK Scientific, AmBeed, Combi-Blocks and Oakwood and used as received without further purification. NMR spectra were acquired using Varian Inova 400 MHz, and Inova 500 MHz spectrometer. Spectra were processed using MNova software. Chemical shifts are reported in parts per million (ppm), coupling constants (*J*) in Hz and are calibrated to residual protonated solvent (^{1}H NMR: $CDCl_3$ = δ 7.26; Acetone-d_6 = δ 2.05 and ^{13}C NMR: $CDCl_3$ = δ 77.16; Acetone-d_6 = δ 29.84). Data are represented as follows: chemical shift, multiplicity (br = broad, s = singlet, d = doublet, t = triplet, q = quartet, d = doublet of doublets, td = triplet of doublets, qd = quartet of doublets, m = multiplet), coupling constants in Hertz (Hz), integration. Diastereomeric ratio (dr) determined by ^{1}H NMR analysis of crude reaction mixture and all spectral data correspond to the major isomer. Cyclic voltammetry data were measured with a BASi Epsilon potentiostat. The high-resolution mass spectrometry (HRMS) data were obtained on a Thermo Fisher Scientific Exactive series DART Mass Spectrometer and Agilent 8860 GC System with an Agilent 5977B Mass Selective Detector. The X-ray diffraction data were collected using Rigaku XtaLAB Synergy diffractometer. The TGA data were measured using TA Discovery TGA 5500. The DSC data were measured using TA DSC SC Q20 V24.11 Build 124. All electrolysis reactions were performed using ElectraSyn 2.0. Electrochemical flow experiments were performed using a stand-alone Vapourtec Ion Electrochemical Reactor with an Aim-TTi EX354RD Dual Power Supply from Thurlby Thandar Instruments Ltd. A Chemyx Fusion 100 Touch Syringe Pump or a Vapourtec SF-10 Reagent Pump were used in the flow setup.

2. Optimization of Reaction Conditions

2.1. Optimization of Electrochemical C–H Azolation

Table S1. Optimization of Electrochemical C–H Azolation

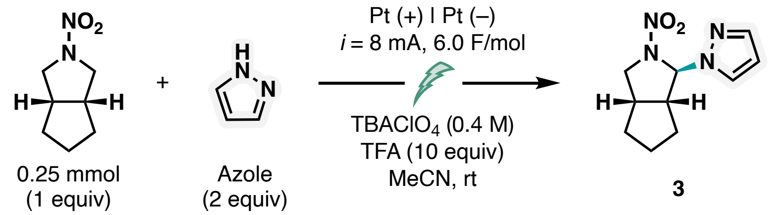

Entry		Deviation from Standard Conditions	Yield of **3**
1		none	63% (> 20:1 dr)
2	atmosphere	under N_2	61% (> 20:1 dr)
3	electrode effect	RVC (+) I Pt (–)	37% (> 20:1 dr)
4		GC (+) I Pt (–)	36% (> 20:1 dr)
5	acid effect	w/o TFA	0%
6		TFA (5 equiv)	32% (> 20:1 dr)
7		TFA (20 equiv)	65% (> 20:1 dr)
8		TsOH (10 equiv)	0%
9		AcOH (10 equiv)	0%
10	azole amount	Azole (1.5 equiv) with TFA (5 equiv)	48% (> 20:1 dr)
11		Azole (3 equiv) with TFA (30 equiv)	65% (> 20:1 dr)
12	temperature	40 °C	35% (> 20:1 dr)
13	solvent effect	MeCN/H_2O (5:1)	0%
14		MeCN/HFIP (5:1)	60% (> 20:1 dr)
15	charge effect	i = 5 mA, 5.0 F/mol	52% (> 20:1 dr)
16		i = 7 mA, 6.0 F/mol	56% (> 20:1 dr)
17		i = 8 mA, 7.0 F/mol	41% (> 20:1 dr)
18		i = 10 mA, 5.0 F/mol	58% (> 20:1 dr)
19		i = 10 mA, 7.0 F/mol	47% (> 20:1 dr)

Table S2. Electrolyte Effect on Electrochemical C–H Azolation

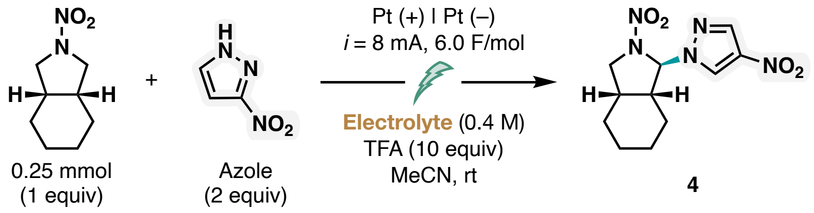

Entry	Deviation from Standard Conditions	Yield of **4**
1	$TBAClO_4$	88% (> 20:1 dr)
2	$TBAPF_6$	70% (> 20:1 dr)
3	$TBABF_4$	49% (> 20:1 dr)
4	TBAOTf	47% (> 20:1 dr)

Table S3. Brønsted Acid Effect on Electrochemical C–H Azolation

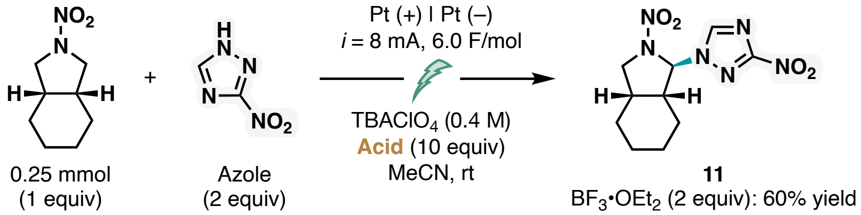

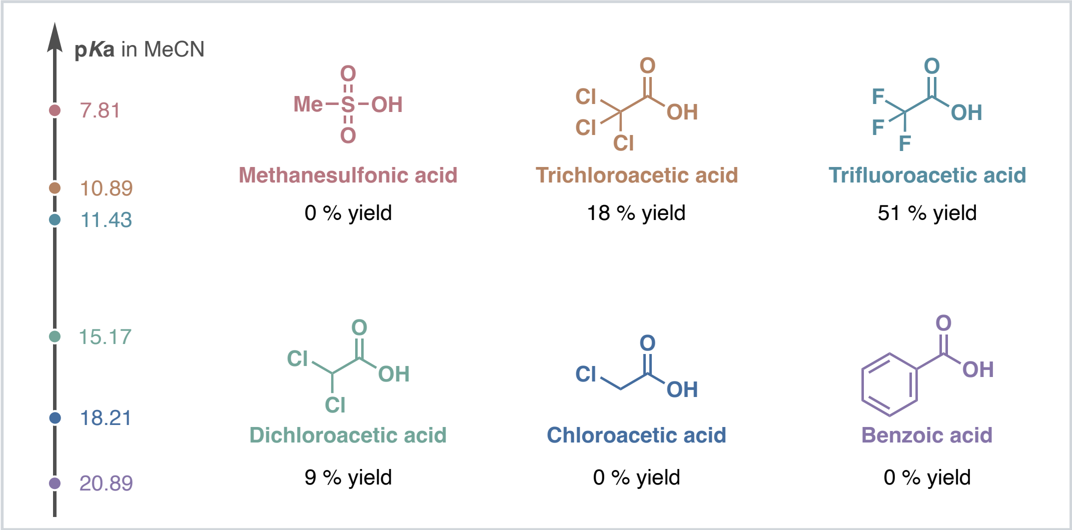

The p*K*a value is predicted by DeepSynthesis (http://isyn.luoszgroup.com/prediction).[1]

Table S4. Lewis Acid Effect on Electrochemical C–H Azolation

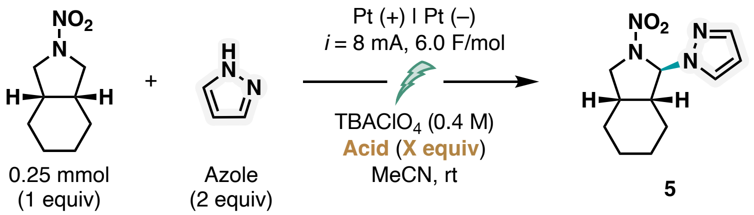

Entry	Deviation from Standard Conditions	Yield of **5**
1	BF_3•OEt_2 (0.5 equiv)	0%
2	BF_3•OEt_2 (2.0 equiv)	34%
3	$AlCl_3$ (2.0 equiv)	0%
4	$Sc(OTf)_3$ (2.0 equiv)	0%
5	BF_3•OEt_2 (2.0 equiv) in MeCN/HFIP (5:1)	19%
6	TFA (10 equiv)	42%

Table S5. Representative Azolation Examples Highlighting Acid Effect

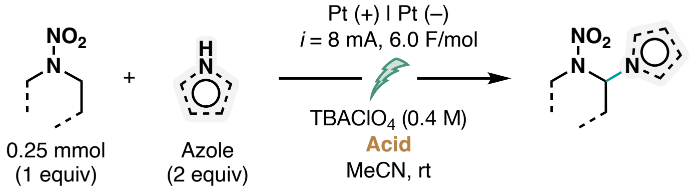

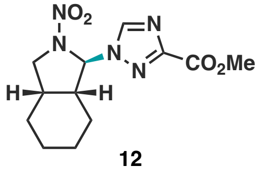

12

TFA : 28% yield

BF_3•OEt_2 : 81% yield

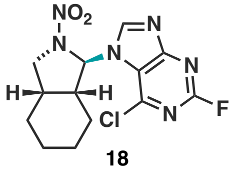

18

TFA : 25% yield

BF_3•OEt_2 : 52% yield

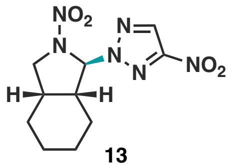

13

TFA : 71% yield

BF_3•OEt_2 : 33% yield

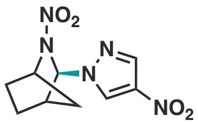

23

TFA : 58% yield

BF_3•OEt_2 : 28% yield

Scheme S1. Unsuccessful Heterocyclic Nucleophiles

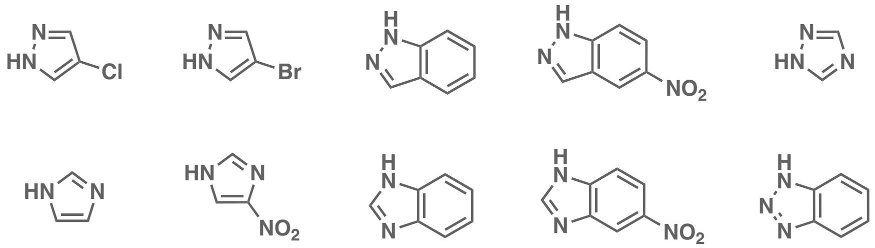

2.2. Optimization of Electrochemical C–H Alkoxylation

Table S6. Optimization of Electrochemical C–H Alkoxylation

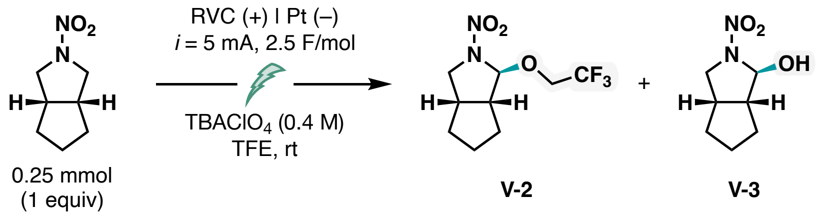

Entry		Deviation from Standard Conditions	Yield of **V-2** / **V-3**
1		none	88% / 9%
2	– moisture	add 4Å MS	87% / 7%
3	cathode effect	RVC (+) I Al (–)	69% / 10%
4		RVC (+) I SS (–)	40% / 20%
5		RVC (+) I C (–)	49% / 19%
6		RVC (+) I Cu (–)	23% / 17%
7	anode effect	GC (+) I Pt (–)	81% / 4%
8		Pt (+) I Pt (–)	57% / 18%
9	electrolyte effect	$LiClO_4$	61% / 0%
10		$TBAPF_6$	28% / 0%
11		TBABr	0% / 0%

2.3. Optimization of Nitramine Synthesis

Table S7. Optimization of Nitramine Synthesis

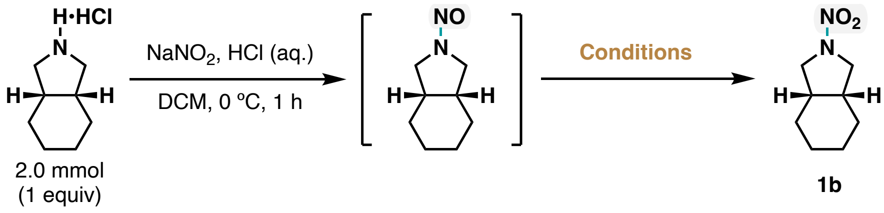

Entry	Oxidation Conditions	Yield of **1b**
1	Oxone (2 equiv), $MeCN/H_2O$ (1:1.5), 40 °C, 16 h	96%
2	*m*CPBA (1.5 equiv), DCE, 40 °C, 16 h	83%
3	MoO_3 (0.3 equiv), H_2O_2 (3 equiv), MeCN, 50 °C, 16 h	90%
4	O_2 (1 atm), Pt (+) I Pt (–), i = 3 mA, 5.0 F/mol, $TBAClO_4$ (0.1 M), MeCN, rt	32%

3. Reaction Setup

3.1. Experimental Setup for Electrochemical C–H Azolation

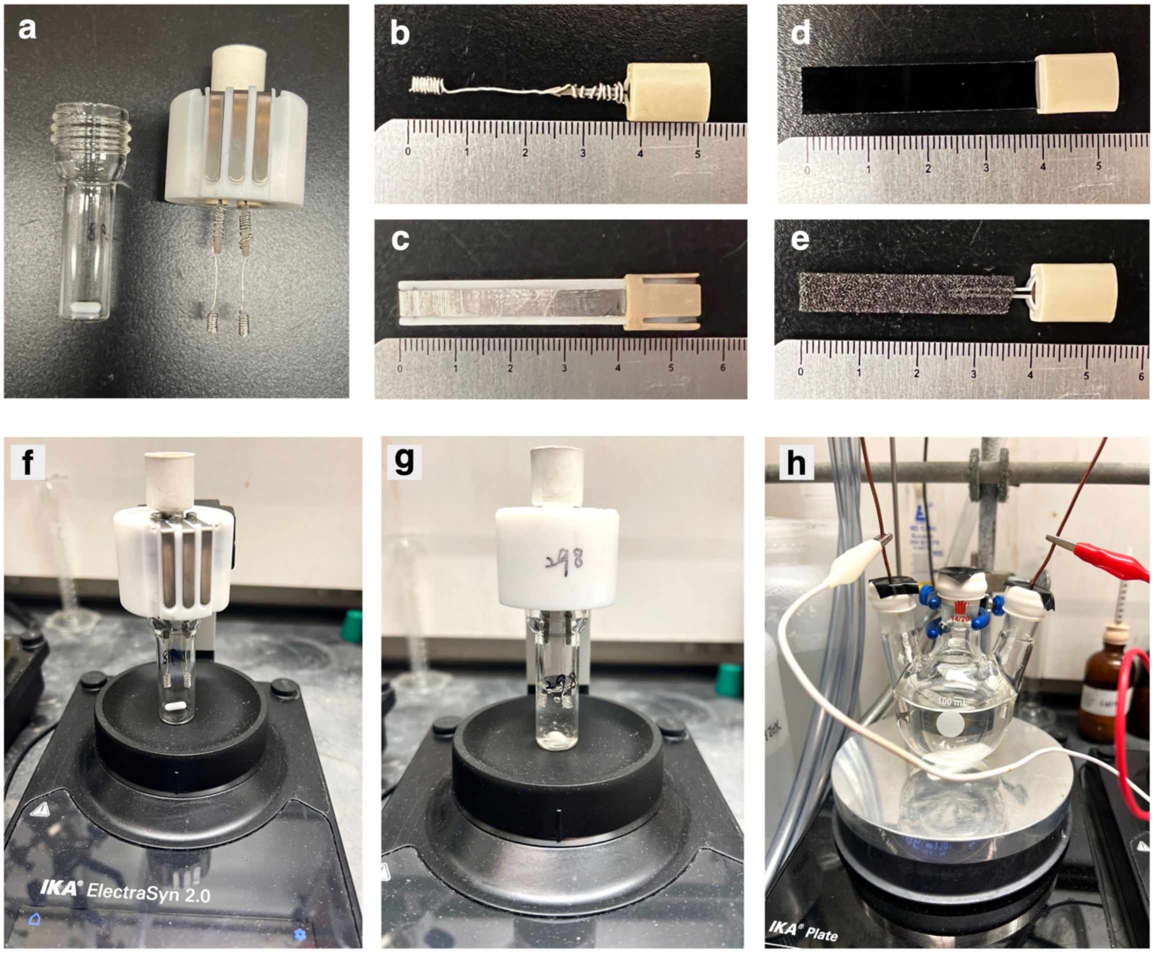

Figure S1. Experimental Setup. (a) A ElectraSyn 5 mL vial and the cap containing a Pt wire anode and a Pt wire cathode. (b) A platinum wire electrode (24 gauge, diameter 2.5 mm). (c) A platinum foil electrode from IKA. (d) A glassy carbon electrode from IKA. (e) A RVC electrode (100 ppi). (f) Assembled ElectraSyn vial and cap for electrolysis. (g) Ongoing electrochemical azolation. (h) Experimental setup for scalable synthesis of electrochemical azolation.

4. Synthesis of Nitramines

4.1. General Procedure for Preparation of Nitramines

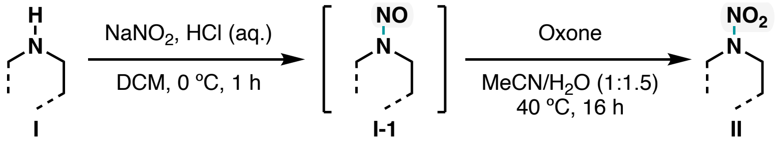

General Procedure: A 50 mL round-bottom flask was charged with 3 mmol of amine **I** or amine salt **I**·HCl (1 equiv), $NaNO_2$ (3 equiv), and DCM (0.2 M). The mixture was cooled to 0 ºC in an ice bath and then aqueous hydrochloric acid (12 M, 2.2 equiv) was added dropwise to the mixture. The solution was stirred at 0 ºC for 1 h, and quenched by the addition of water (10 mL) at 0 ºC. The crude mixture was then extracted with DCM and the combined organic layers were washed with brine, dried over anhydrous Na_2SO_4, filtered, and the mixture was concentrated *in vacuo* to give the nitrosamine **I-1**, which was used directly in the next step without purification. *(**Caution**: The addition of HCl may lead to the formation of NO_2, and the nitrosamine compounds are known to be carcinogenic.)*

A 50 mL round-bottom flask was charged with the resulting nitrosamine **I-1** (1 equiv), Oxone (2 equiv), and $MeCN/H_2O$ (1:1.5, 0.5 M). The mixture was stirred at 40 ºC for 16 h. After the reaction completed, the crude mixture was then extracted with ethyl acetate and the combined organic layers were washed with brine, dried over anhydrous Na_2SO_4, filtered, and the mixture was concentrated *in vacuo*. The residue was purified via a flash chromatography to deliver the nitramine **II**. (***Safety Precaution**: The use of appropriate personal protective equipment is strongly recommended. **Safety Recommendation**: Use a blast shield when performing reactions and concentrating under reduced pressure.)*

2-Nitrooctahydrocyclopenta[*c*]pyrrole (1a)

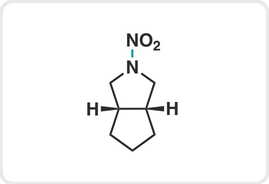

Yield: 95%. Pale yellow solid. ^{1}H NMR (400 MHz, $CDCl_3$) δ 4.05 – 3.94 (m, 2H), 3.59 (dq, *J* = 11.5, 3.4 Hz, 2H), 2.76 (dhept, *J* = 8.4, 4.1 Hz, 2H), 1.87 (dp, *J* = 13.5, 6.9 Hz, 2H), 1.75 (dp, *J* = 13.0, 6.5 Hz, 1H), 1.63 (dq, *J* = 13.2, 6.8 Hz, 1H), 1.56 – 1.44 (m, 2H). ^{13}C NMR (101 MHz, $CDCl_3$) δ 56.1, 41.4, 32.2, 25.4. HRMS-(DART+) calculated for $C_7H_{13}N_2O_2$ $[M+H]^+$: 157.0972, found: 157.0974.

2-Nitrooctahydro-1*H*-isoindole (1b)

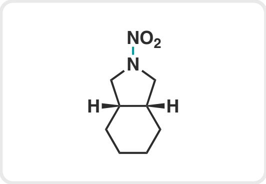

Yield: 96%. White solid. ^{1}H NMR (500 MHz, $CDCl_3$) δ 3.90 – 3.82 (m, 2H), 3.78 – 3.70 (m, 2H), 2.35 (ddd, *J* = 10.6, 6.3, 4.4 Hz, 2H), 1.65 (dtd, *J* = 10.6, 6.1, 2.7 Hz, 2H), 1.58 – 1.37 (m, 6H). ^{13}C NMR (126 MHz, $CDCl_3$) δ 53.9, 36.2, 25.8, 22.5. HRMS-(DART+) calculated for $C_8H_{15}N_2O_2$ $[M+H]^+$: 171.1128, found: 171.1130.

1-Nitropyrrolidine (1c)

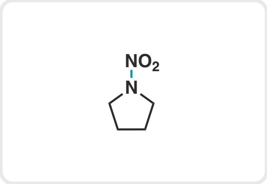

Yield: 50%. White solid. ^{1}H NMR (500 MHz, $CDCl_3$) δ 3.96 – 3.61 (m, 4H), 2.09 – 1.96 (m, 4H). ^{13}C NMR (126 MHz, $CDCl_3$) δ 50.0, 24.2. HRMS-(DART+) calculated for $C_4H_9N_2O_2$ $[M+H]^+$: 117.0659, found: 117.0663.

3-Nitro-3-azabicyclo[3.1.0]hexane (1d)

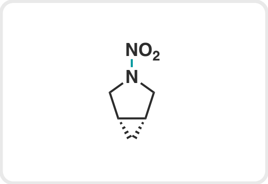

Yield: 96%. Pale yellow solid. ^{1}H NMR (500 MHz, $CDCl_3$) δ 4.03 (d, *J* = 11.7 Hz, 2H), 3.92 (ddd, *J* = 11.4, 3.0, 1.6 Hz, 2H), 1.72 – 1.64 (m, 2H), 0.97 – 0.90 (m, 1H), 0.33 (dt, *J* = 5.7, 4.2 Hz, 1H). ^{13}C NMR (126 MHz, $CDCl_3$) δ 52.8, 14.6, 11.4. HRMS-(DART+) calculated for $C_5H_9N_2O_2$ $[M+H]^+$: 129.0659, found: 129.0661.

Methyl nitro-*L*-prolinate (1e)

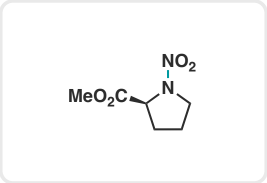

Yield: 80%. Pale yellow solid. ^{1}H NMR (500 MHz, $CDCl_3$) δ 4.83 – 4.76 (m, 1H), 4.11 – 4.02 (m, 1H), 3.98 – 3.89 (m, 1H), 3.78 (s, 3H), 2.45 – 2.32 (m, 1H), 2.21 – 2.03 (m, 3H). ^{13}C NMR (126 MHz, $CDCl_3$) δ 170.1, 61.8, 52.9, 50.3, 29.2, 22.7. HRMS-(DART+) calculated for $C_6H_{11}N_2O_4$ $[M+H]^+$: 175.0713, found: 175.0715.

2-Nitro-2-azabicyclo[2.2.1]heptane (1f)

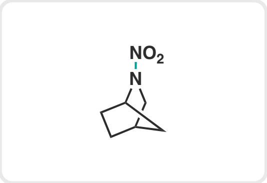

Yield: 86%. Pale yellow liquid. ^{1}H NMR (500 MHz, $CDCl_3$) δ 4.77 (s, 1H), 3.74 (ddd, *J* = 11.0, 3.6, 2.3 Hz, 1H), 3.57 (dd, *J* = 11.1, 2.0 Hz, 1H), 2.67 (dqd, *J* = 3.8, 2.7, 1.7 Hz, 1H), 1.99 – 1.89 (m, 1H), 1.88 – 1.67 (m, 3H), 1.58 – 1.46 (m, 2H). ^{13}C NMR (126 MHz, $CDCl_3$) δ 62.1, 58.5, 37.2, 37.0, 27.3, 26.9. HRMS-(DART+) calculated for $C_6H_{14}N_3O_2$ $[M+NH_4]^+$: 160.1081, found: 160.1083.

2-Nitro-2-azaspiro[3.3]heptane (1g)

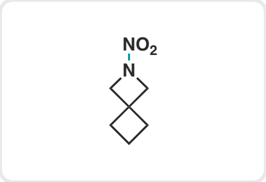

Yield: 92%. Pale yellow solid. ^{1}H NMR (400 MHz, Acetone-d_6) δ 4.33 (s, 4H), 2.24 (t, J = 7.7 Hz, 4H), 1.91 – 1.82 (m, 2H). ^{13}C NMR (101 MHz, Acetone-d_6) δ 69.6, 35.9, 32.8, 16.7. HRMS-(DART+) calculated for $C_6H_{11}N_2O_2$ [M+H]$^+$: 143.0815, found: 143.0819.

3,3-Dimethyl-1-nitroazetidine (1h)

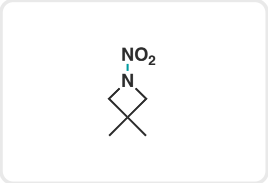

Yield: 82%. White solid. ^{1}H NMR (500 MHz, $CDCl_3$) δ 4.06 (s, 4H), 1.32 (s, 6H). ^{13}C NMR (126 MHz, $CDCl_3$) δ 68.9, 28.8, 26.7. HRMS-(DART+) calculated for $C_5H_{11}N_2O_2$ [M+H]$^+$: 131.0815, found: 131.0818.

1-Nitropiperidine (1i)

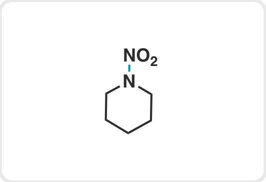

Yield: 75%. Pale yellow solid. ^{1}H NMR (400 MHz, $CDCl_3$) δ 3.86 (t, J = 5.8 Hz, 4H), 1.72 (p, J = 5.8 Hz, 4H), 1.58 (dt, J = 6.8, 3.5 Hz, 2H). ^{13}C NMR (101 MHz, $CDCl_3$) δ 49.4, 24.3, 22.9. HRMS-(DART+) calculated for $C_5H_{11}N_2O_2$ [M+H]$^+$: 131.0815, found: 131.0819.

1-Nitro-2-phenylpyrrolidine (35)

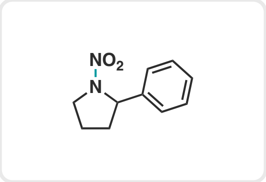

Yield: 53%. Yellow liquid. ^{1}H NMR (500 MHz, $CDCl_3$) δ 7.39 – 7.33 (m, 2H), 7.32 – 7.27 (m, 1H), 7.22 (dd, J = 7.2, 1.7 Hz, 2H), 5.37 (dd, J = 8.2, 3.9 Hz, 1H), 4.12 (dd, J = 7.8, 6.0 Hz, 2H), 2.60 – 2.40 (m, 1H), 2.17 – 1.99 (m, 3H). ^{13}C NMR (126 MHz, $CDCl_3$) δ 140.2, 128.9, 127.9, 125.6, 64.9, 51.5, 34.4, 22.5. HRMS-(DART+) calculated for $C_{10}H_{13}N_2O_2$ [M+H]$^+$: 193.0972, found: 193.0975.

5. Synthesis of Azolated Nitramines

5.1. General Procedure for Electrochemical C–H Azolation

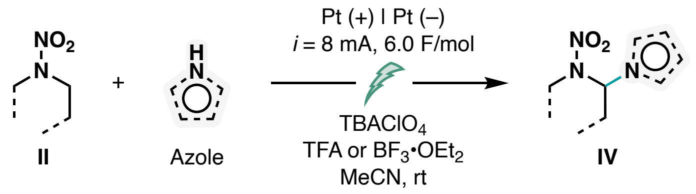

General Procedure: An oven-dried 5 mL ElectraSyn vial was charged with nitramine **II** (0.25 mmol, 1 equiv), azole (0.50 mmol, 2 equiv), $TBAClO_4$ (1.00 mmol, 0.4 M), an additive (10 equiv of TFA, or 2 equiv of $BF_3 \cdot OEt_2$) and anhydrous MeCN (2.5 mL). The mixture was then stirred until a clear solution formed. The vial was fitted with caps containing a Pt wire anode and a Pt wire cathode, and electrolysis was conducted at a constant current of 8 mA for 6.0 F/mol. After the reaction completed, the mixture was transferred to a round-bottom flask, and the electrodes were rinsed with acetone. The solution was then concentrated *in vacuo*, and the crude mixture was purified by silica gel flash chromatography to afford the azolated product **IV**. (***Safety Precaution****: The use of appropriate personal protective equipment is strongly recommended.* ***Safety Recommendation****: Use a blast shield when performing reactions and concentrating under reduced pressure.)*

2-Nitro-1-(1*H*-pyrazol-1-yl)octahydrocyclopenta[*c*]pyrrole (3)

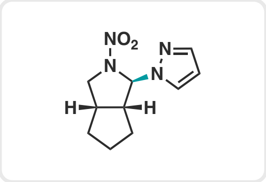

General Procedure using 10 equiv of TFA (0.2 mL) in 2.3 mL of MeCN. Yield: 63%. Yellow oil. dr: > 20:1. ^1H NMR (400 MHz, Acetone-d_6) δ 7.86 (d, J = 2.3 Hz, 1H), 7.52 (d, J = 1.8 Hz, 1H), 6.38 (d, J = 1.3 Hz, 1H), 6.23 (t, J = 2.1 Hz, 1H), 4.27 (dd, J = 11.1, 9.0 Hz, 1H), 3.74 (dd, J = 11.2, 5.0 Hz, 1H), 3.25 (ddt, J = 13.0, 8.7, 4.4 Hz, 1H), 2.98 – 2.90 (m, 1H), 2.14 – 2.07 (m, 1H), 2.00 – 1.90 (m, 1H), 1.88 – 1.73 (m, 2H), 1.72 – 1.62 (m, 2H). ^{13}C NMR (101 MHz, Acetone-d_6) δ 141.3, 131.9, 105.6, 80.1, 57.4, 50.8, 40.8, 33.2, 31.9, 26.1. HRMS-(DART+) calculated for $C_{10}H_{15}N_4O_2$ $[M+H]^+$: 223.1190, found: 223.1195.

2-Nitro-1-(4-nitro-1*H*-pyrazol-1-yl)octahydro-1*H*-isoindole (4)

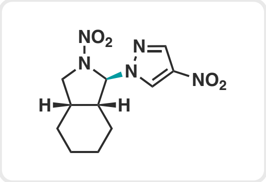

General Procedure using 10 equiv of TFA (0.2 mL) in 2.3 mL of MeCN. Yield: 88%. Pale yellow solid. dr: > 20:1. ^1H NMR (400 MHz, $CDCl_3$) δ 8.43 (s, 1H), 8.08 (s, 1H), 6.01 (d, J = 2.7 Hz, 1H), 4.05 (dd, J = 11.1, 7.6 Hz, 1H), 3.96 (dd, J = 11.1, 8.9 Hz, 1H), 3.01 (tq, J = 9.7, 5.5 Hz, 1H), 2.78 (qt, J = 6.1, 2.4 Hz, 1H), 1.87 (dt, J = 12.3, 6.3 Hz, 1H), 1.74 – 1.32 (m, 7H). ^{13}C NMR (101

MHz, Acetone-d_6) δ 136.9, 130.9, 80.6, 53.7, 44.8, 34.3, 30.6, 25.5, 25.2, 23.9, 22.1. HRMS-(DART+) calculated for $C_{11}H_{16}N_5O_4$ $[M+H]^+$: 282.1197, found: 282.1202.

2-Nitro-1-(1*H*-pyrazol-1-yl)octahydro-1*H*-isoindole (5)

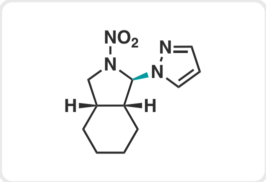

General Procedure using 10 equiv of TFA (0.2 mL) in 2.3 mL of MeCN. Yield: 42%. Colorless oil. dr: > 20:1. ^{1}H NMR (400 MHz, $CDCl_3$) δ 7.70 (d, *J* = 2.5 Hz, 1H), 7.56 (d, *J* = 1.8 Hz, 1H), 6.26 (t, *J* = 2.1 Hz, 1H), 6.03 (d, *J* = 2.7 Hz, 1H), 4.05 (dd, *J* = 11.2, 7.6 Hz, 1H), 3.95 (dd, *J* = 11.2, 9.0 Hz, 1H), 3.12 (tq, *J* = 9.9, 5.5 Hz, 1H), 2.83 (qt, *J* = 5.9, 2.5 Hz, 1H), 1.90 – 1.80 (m, 1H), 1.71 (ddd, *J* = 14.8, 9.9, 5.0 Hz, 2H), 1.64 – 1.45 (m, 3H), 1.39 – 1.27 (m, 2H). ^{13}C NMR (101 MHz, $CDCl_3$) δ 140.7, 130.9, 105.7, 79.2, 52.8, 43.2, 33.8, 25.6, 24.6, 23.4, 21.6. HRMS-(DART+) calculated for $C_{11}H_{17}N_4O_2$ $[M+H]^+$: 237.1346, found: 237.1351.

Methyl 1-(2-nitrooctahydrocyclopenta[*c*]pyrrol-1-yl)-1*H*-pyrazole-4-carboxylate (6)

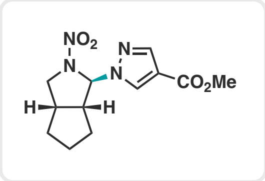

General Procedure using 10 equiv of TFA (0.2 mL) in 2.3 mL of MeCN. Yield: 65%. White solid. dr: 92:8. ^{1}H NMR (400 MHz, Acetone-d_6) δ 8.41 (s, 1H), 7.91 (s, 1H), 6.47 – 6.42 (m, 1H), 4.30 (dd, *J* = 11.1, 9.1 Hz, 1H), 3.80 – 3.75 (m, 4H), 3.31 – 3.21 (m, 1H), 2.98 (tdd, *J* = 8.5, 6.7, 1.4 Hz, 1H), 2.15 – 2.07 (m, 1H), 1.99 – 1.92 (m, 1H), 1.89 – 1.76 (m, 2H), 1.68 (dddd, *J* = 13.0, 11.8, 6.0, 2.9 Hz, 2H). ^{13}C NMR (101 MHz, Acetone-d_6) δ 163.4, 142.5, 135.3, 115.0, 80.5, 57.5, 51.5, 50.8, 40.7, 32.9, 31.8, 25.9. HRMS-(DART+) calculated for $C_{12}H_{17}N_4O_4$ $[M+H]^+$: 281.1244, found: 281.1251.

2-Nitro-1-(4-(trifluoromethyl)-1*H*-pyrazol-1-yl)octahydrocyclopenta[*c*]pyrrole (7)

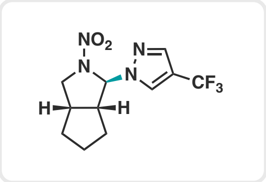

General Procedure using 10 equiv of TFA (0.2 mL) in 2.3 mL of MeCN. Yield: 54%. White solid. dr: > 20:1. ^{1}H NMR (400 MHz, $CDCl_3$) δ 7.97 (t, *J* = 1.0 Hz, 1H), 7.73 (s, 1H), 6.13 (d, *J* = 1.4 Hz, 1H), 4.24 (dd, *J* = 11.3, 9.0 Hz, 1H), 3.74 (dd, *J* = 11.3, 5.2 Hz, 1H), 3.29 (tp, *J* = 8.6, 4.4 Hz, 1H), 3.11 – 3.00 (m, 1H), 2.13 (ddd, *J* = 12.6, 8.8, 6.3 Hz, 1H), 2.06 – 1.93 (m, 1H), 1.91 – 1.75 (m, 1H), 1.71 – 1.59 (m, 3H). ^{19}F NMR (376 MHz, $CDCl_3$) δ -56.60. ^{13}C NMR (101 MHz, $CDCl_3$) δ 138.4 (q, *J* = 2.7 Hz), 130.6 (q, *J* = 3.7 Hz), 122.4 (q, *J* = 266.2 Hz), 113.6 (q, *J* = 38.5 Hz), 79.6, 56.6, 49.4, 40.3, 32.7, 31.7, 25.6. HRMS-(DART+) calculated for $C_{11}H_{14}F_3N_4O_2$ $[M+H]^+$: 291.1063, found: 291.1069.

2-Nitro-1-(4-nitro-1*H*-pyrazol-1-yl)octahydrocyclopenta[*c*]pyrrole (8)

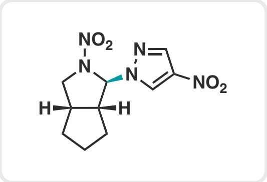

General Procedure using 10 equiv of TFA (0.2 mL) in 2.3 mL of MeCN. Yield: 82%. White solid. dr: > 20:1. ^{1}H NMR (400 MHz, Acetone-d_6) δ 8.86 (s, 1H), 8.22 (s, 1H), 6.49 (d, J = 1.5 Hz, 1H), 4.32 (dd, J = 11.1, 9.0 Hz, 1H), 3.80 (dd, J = 11.1, 5.7 Hz, 1H), 3.33 – 3.20 (m, 1H), 3.03 (tdd, J = 8.5, 6.6, 1.5 Hz, 1H), 2.21 – 2.09 (m, 1H), 2.02 – 1.78 (m, 3H), 1.78 – 1.59 (m, 2H). ^{13}C NMR (101 MHz, Acetone-d_6) δ 137.3, 131.3, 81.1, 57.4, 50.9, 40.7, 32.7, 31.6, 25.8. HRMS-(DART+) calculated for $C_{10}H_{17}N_6O_4$ [M+NH$_4$]$^+$: 285.1306, found: 285.1314. The structure was characterized by X-ray crystallography.

1-(2-Nitrooctahydro-1*H*-isoindol-1-yl)-1*H*-pyrazole-4-carbonitrile (9)

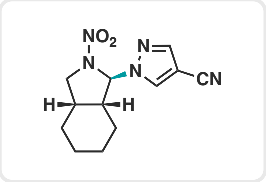

General Procedure using 2 equiv of $BF_3\cdot OEt_2$.
Yield: 67%. White solid. dr: 93:7. ^{1}H NMR (400 MHz, $CDCl_3$) δ 8.14 (s, 1H), 7.82 (s, 1H), 6.01 (d, J = 2.6 Hz, 1H), 4.03 (dd, J = 11.1, 7.6 Hz, 1H), 3.95 (dd, J = 11.1, 9.0 Hz, 1H), 3.05 (tq, J = 9.5, 5.4 Hz, 1H), 2.79 (dtd, J = 11.7, 5.6, 2.4 Hz, 1H), 1.85 (dd, J = 12.7, 6.3 Hz, 1H), 1.74 – 1.61 (m, 3H), 1.58 – 1.42 (m, 2H), 1.41 – 1.26 (m, 2H). ^{13}C NMR (101 MHz, $CDCl_3$) δ 143.1, 136.0, 112.9, 92.9, 79.6, 52.9, 43.3, 33.8, 25.5, 24.5, 23.4, 21.3. HRMS-(DART+) calculated for $C_{12}H_{16}N_5O_2$ [M+H]$^+$: 262.1299, found: 262.1305.

1-(2-Nitrooctahydro-1*H*-isoindol-1-yl)-1*H*-imidazole-4,5-dicarbonitrile (10)

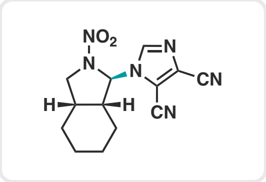

General Procedure using 2 equiv of $BF_3\cdot OEt_2$.
Yield: 30%. White solid. dr: > 20:1. ^{1}H NMR (400 MHz, $CDCl_3$) δ 7.85 (s, 1H), 6.16 (d, J = 5.7 Hz, 1H), 4.14 (qd, J = 11.6, 6.0 Hz, 2H), 2.73 – 2.52 (m, 2H), 1.93 – 1.72 (m, 2H), 1.69 – 1.59 (m, 2H), 1.58 – 1.37 (m, 4H). ^{13}C NMR (101 MHz, $CDCl_3$) δ 139.9, 124.8, 111.2, 110.1, 107.7, 76.6, 55.4, 45.4, 34.8, 25.7, 24.2, 22.3, 21.9. HRMS-(DART+) calculated for $C_{13}H_{15}N_6O_2$ [M+H]$^+$: 287.1251, found: 287.1257.

2-Nitro-1-(3-nitro-1*H*-1,2,4-triazol-1-yl)octahydro-1*H*-isoindole (11)

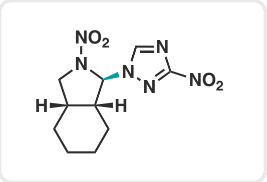

General Procedure using 2 equiv of $BF_3\cdot OEt_2$.
Yield: 60%. Yellow solid. dr: > 20:1. ^{1}H NMR (400 MHz, Acetone-d_6) δ 8.93 (s, 1H), 6.56 (d, J = 2.6 Hz, 1H), 4.15 (dd, J = 10.9, 7.6 Hz, 1H), 4.05 (dd, J = 10.9, 9.1 Hz, 1H), 3.08 (h, J = 5.4 Hz, 1H), 2.81 (tt, J = 7.8, 3.9 Hz, 1H), 2.01 – 1.89 (m, 1H), 1.74 (dt, J = 12.1, 5.0 Hz, 3H), 1.64 – 1.50 (m, 3H), 1.47 – 1.31 (m, 1H). ^{13}C NMR (101 MHz, Acetone-d_6) δ 147.7, 79.2, 53.4, 44.7, 34.4, 25.4, 24.9, 24.0, 21.9. HRMS-(DART+) calculated for $C_{10}H_{18}N_7O_4$ [M+NH$_4$]$^+$: 300.1415, found: 300.1424.

Methyl 1-(2-nitrooctahydro-1*H*-isoindol-1-yl)-1*H*-1,2,4-triazole-3-carboxylate (12)

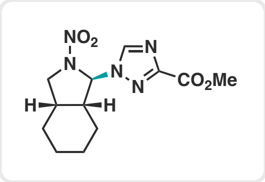

General Procedure using 2 equiv of $BF_3 \cdot OEt_2$.

Yield: 81%. White solid. dr: 95:5. ^{1}H NMR (400 MHz, $CDCl_3$) δ 8.45 (s, 1H), 6.16 (d, *J* = 3.0 Hz, 1H), 4.04 (dd, *J* = 11.1, 7.4 Hz, 1H), 3.95 (s, 4H), 3.01 (h, *J* = 5.8 Hz, 1H), 2.83 (tq, *J* = 6.1, 2.8 Hz, 1H), 1.84 (dt, *J* = 11.8, 6.0 Hz, 1H), 1.68 (qd, *J* = 10.2, 5.0 Hz, 2H), 1.60 – 1.42 (m, 3H), 1.34 (dtt, *J* = 13.0, 9.4, 4.9 Hz, 2H). ^{13}C NMR (101 MHz, $CDCl_3$) δ 160.0, 155.4, 145.9, 77.7, 53.0, 52.9, 43.1, 33.8, 25.2, 24.5, 23.2, 21.3. HRMS-(DART+) calculated for $C_{12}H_{18}N_5O_4$ $[M+H]^+$: 296.1353, found: 296.1361.

2-Nitro-1-(4-nitro-2*H*-1,2,3-triazol-2-yl)octahydro-1*H*-isoindole (13)

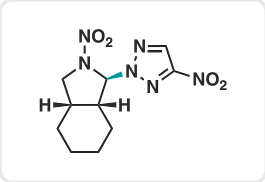

General Procedure using 10 equiv of TFA (0.2 mL) in 2.3 mL of MeCN.

Yield: 71%. White solid. dr: > 20:1. ^{1}H NMR (400 MHz, $CDCl_3$) δ 8.21 (s, 1H), 6.44 (d, *J* = 2.4 Hz, 1H), 4.20 (dd, *J* = 10.9, 7.8 Hz, 1H), 4.00 (dd, *J* = 10.9, 8.9 Hz, 1H), 2.97 (tq, *J* = 9.5, 5.1 Hz, 1H), 2.57 (dtd, *J* = 9.8, 5.6, 2.3 Hz, 1H), 1.94 (dq, *J* = 10.2, 4.2 Hz, 1H), 1.72 (ddt, *J* = 12.3, 6.0, 3.6 Hz, 1H), 1.68 – 1.61 (m, 2H), 1.57 (dq, *J* = 13.1, 4.0 Hz, 1H), 1.51 – 1.42 (m, 1H), 1.36 (dtd, *J* = 13.6, 10.5, 7.7 Hz, 2H). ^{13}C NMR (101 MHz, $CDCl_3$) δ 131.2, 82.9, 52.6, 44.6, 33.2, 25.3, 24.4, 23.3, 21.1. HRMS-(DART+) calculated for $C_{10}H_{18}N_7O_4$ $[M+NH_4]^+$: 300.1415, found: 300.1421.

Ethyl 2-(2-nitrooctahydro-1*H*-isoindol-1-yl)-2*H*-1,2,3-triazole-4-carboxylate (14)

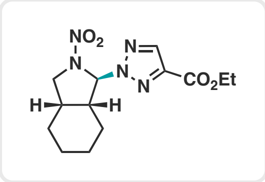

General Procedure using 2 equiv of $BF_3 \cdot OEt_2$.

Yield: 62%. Pale yellow oil. dr: > 20:1. ^{1}H NMR (400 MHz, $CDCl_3$) δ 8.05 (s, 1H), 6.47 (d, *J* = 2.5 Hz, 1H), 4.41 (q, *J* = 7.1 Hz, 2H), 4.19 (dd, *J* = 10.9, 7.8 Hz, 1H), 3.97 (dd, *J* = 11.0, 8.9 Hz, 1H), 2.99 (ddt, *J* = 13.7, 8.5, 4.8 Hz, 1H), 2.56 – 2.49 (m, 1H), 1.93 – 1.83 (m, 1H), 1.69 (ddt, *J* = 12.0, 5.8, 2.7 Hz, 1H), 1.63 (dt, *J* = 8.1, 4.8 Hz, 2H), 1.59 – 1.44 (m, 2H), 1.39 (d, *J* = 7.1 Hz, 3H), 1.36 – 1.26 (m, 2H). ^{13}C NMR (101 MHz, $CDCl_3$) δ 160.4, 140.4, 137.3, 81.9, 61.7, 52.6, 44.5, 33.2, 25.3, 24.5, 23.4, 21.2, 14.4. HRMS-(DART+) calculated for $C_{13}H_{23}N_6O_4$ $[M+NH_4]^+$: 327.1775, found: 327.1781.

2-Nitro-1-(2*H*-1,2,3-triazol-2-yl)octahydro-1*H*-isoindole (15)

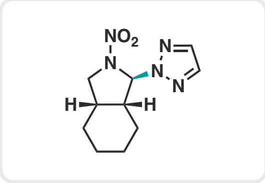

General Procedure using 2 equiv of $BF_3 \cdot OEt_2$.

Yield: 20%. Colorless oil. dr: > 20:1. ^{1}H NMR (400 MHz, $CDCl_3$) δ 7.65 (s, 2H), 6.46 (d, *J* = 2.4 Hz, 1H), 4.18 (dd, *J* = 11.0, 7.9 Hz, 1H), 3.98 (dd, *J* = 11.0, 8.9 Hz, 1H), 3.05 (td, *J* = 8.6, 4.4 Hz, 1H), 2.54 (dtd, *J* = 10.1, 5.7, 2.3 Hz, 1H), 1.96 – 1.86 (m, 1H), 1.72 (ddd, *J* = 11.0, 6.2, 2.8 Hz, 1H), 1.66 (q, *J* = 4.3 Hz, 2H), 1.58 – 1.52 (m, 1H), 1.50 – 1.42 (m, 1H), 1.39 – 1.30 (m, 2H). ^{13}C NMR (101 MHz, $CDCl_3$) δ 135.0, 81.4,

52.6, 44.3, 33.3, 25.5, 24.6, 23.5, 21.2. HRMS-(DART+) calculated for $C_{10}H_{16}N_5O_2$ $[M+H]^+$: 238.1299, found: 238.1304.

Ethyl 2-(2-nitrooctahydro-1*H*-isoindol-1-yl)-2*H*-tetrazole-5-carboxylate (16)

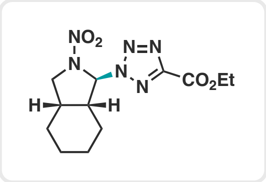

General Procedure using 2 equiv of $BF_3{\cdot}OEt_2$.

Yield: 64%. Pale yellow solid. dr: 93:7. ^{1}H NMR (400 MHz, $CDCl_3$) δ 6.72 (d, *J* = 2.4 Hz, 1H), 4.54 – 4.48 (m, 2H), 4.22 (dd, *J* = 10.9, 7.8 Hz, 1H), 4.05 – 3.99 (m, 1H), 3.01 (tt, *J* = 9.7, 4.9 Hz, 1H), 2.57 (dtd, *J* = 10.7, 5.6, 2.4 Hz, 1H), 1.96 (dtt, *J* = 11.1, 5.6, 2.4 Hz, 1H), 1.74 (dt, *J* = 14.2, 3.5 Hz, 1H), 1.67 (d, *J* = 5.1 Hz, 2H), 1.58 (dq, *J* = 17.8, 4.6 Hz, 1H), 1.51 – 1.46 (m, 1H), 1.44 (t, *J* = 7.1 Hz, 3H), 1.40 – 1.24 (m, 2H). ^{13}C NMR (101 MHz, $CDCl_3$) δ 158.0, 157.6, 80.9, 62.9, 52.5, 44.6, 33.3, 25.2, 24.3, 23.3, 20.9, 14.3. HRMS-(DART+) calculated for $C_{12}H_{22}N_7O_4$ $[M+NH_4]^+$: 328.1728, found: 328.1735.

2-Nitro-1-(5-phenyl-2*H*-tetrazol-2-yl)octahydro-1*H*-isoindole (17)

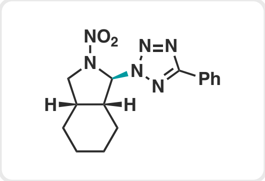

General Procedure using 2 equiv of $BF_3{\cdot}OEt_2$.

Yield: 36%. Colorless oil. dr: 95:5. ^{1}H NMR (400 MHz, $CDCl_3$) δ 8.20 – 8.12 (m, 2H), 7.49 (dd, *J* = 5.0, 2.0 Hz, 3H), 6.72 (d, *J* = 2.2 Hz, 1H), 4.25 (dd, *J* = 10.9, 7.9 Hz, 1H), 4.05 (dd, *J* = 10.9, 9.2 Hz, 1H), 3.13 (tt, *J* = 8.8, 4.2 Hz, 1H), 2.61 (dtd, *J* = 10.8, 5.6, 2.1 Hz, 1H), 2.07 – 1.94 (m, 1H), 1.81 – 1.75 (m, 1H), 1.74 – 1.58 (m, 3H), 1.56 – 1.31 (m, 3H). ^{13}C NMR (101 MHz, $CDCl_3$) δ 165.6, 130.8, 129.1, 127.2, 127.1, 80.4, 52.5, 44.5, 33.4, 25.5, 24.4, 23.5, 21.1. HRMS-(DART+) calculated for $C_{15}H_{19}N_6O_2$ $[M+H]^+$: 315.1564, found: 315.1571.

6-Chloro-2-fluoro-7-(2-nitrooctahydro-1*H*-isoindol-1-yl)-7*H*-purine (18)

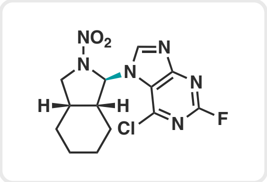

General Procedure using 2 equiv of $BF_3{\cdot}OEt_2$.

Yield: 52%. Pale yellow solid. dr: > 20:1. ^{1}H NMR (400 MHz, $CDCl_3$) δ 8.24 (s, 1H), 6.31 (d, *J* = 5.4 Hz, 1H), 4.23 (dd, *J* = 11.4, 6.5 Hz, 1H), 4.09 (dd, *J* = 11.4, 6.0 Hz, 1H), 3.00 (p, *J* = 5.8 Hz, 1H), 2.79 (p, *J* = 6.2 Hz, 1H), 1.84 (dq, *J* = 9.4, 4.2 Hz, 1H), 1.79 – 1.73 (m, 1H), 1.67 – 1.58 (m, 2H), 1.48 (tdd, *J* = 10.1, 7.1, 2.9 Hz, 4H). ^{19}F NMR (376 MHz, $CDCl_3$) δ -48.43. ^{13}C NMR (101 MHz, $CDCl_3$) δ 157.2 (d, *J* = 221.0 Hz), 153.4 (d, *J* = 17.5 Hz), 152.8 (d, *J* = 16.9 Hz), 145.1 (d, *J* = 3.2 Hz), 131.1 (d, *J* = 5.0 Hz), 74.7, 54.9, 42.9, 34.6, 25.6, 24.5, 22.3, 22.2. HRMS-(DART+) calculated for $C_{13}H_{15}ClFN_6O_2$ $[M+H]^+$: 341.0924, found: 341.0934.

1-(1-Nitropyrrolidin-2-yl)-1*H*-pyrazole (19)

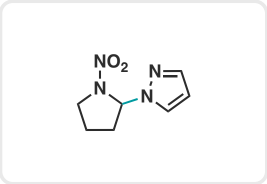

General Procedure using 10 equiv of TFA (0.2 mL) in 2.3 mL of MeCN. Yield: 40%. White solid. ^{1}H NMR (500 MHz, $CDCl_3$) δ 7.75 (d, *J* = 2.4 Hz, 1H), 7.58 (d, *J* = 1.7 Hz, 1H), 6.43 (dd, *J* = 8.0, 1.7 Hz, 1H), 6.28 (t, *J* = 2.1 Hz, 1H), 4.14 (ddd, *J* = 11.1, 8.3, 2.6 Hz, 1H), 3.94 (ddd, *J* = 11.1, 9.7, 7.4 Hz, 1H), 2.80 – 2.67 (m, 1H), 2.63 (ddt, *J* = 13.4, 7.2, 2.1 Hz, 1H), 2.49 (ddt, *J* = 13.4, 11.4, 7.8 Hz, 1H), 2.20 (dtt, *J* = 12.4, 7.5, 2.5 Hz, 1H). ^{13}C NMR (126 MHz, $CDCl_3$) δ 140.9, 131.0, 105.8, 73.9, 50.6, 31.4, 22.7. HRMS-(DART+) calculated for $C_7H_{11}N_4O_2$ $[M+H]^+$: 183.0876, found: 183.0877.

4-Nitro-1-(1-nitropyrrolidin-2-yl)-1*H*-pyrazole (20)

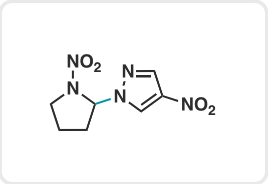

General Procedure using 10 equiv of TFA (0.2 mL) in 2.3 mL of MeCN. Yield: 81%. White solid. ^{1}H NMR (400 MHz, $CDCl_3$) δ 8.46 (s, 1H), 8.09 (s, 1H), 6.43 – 6.33 (m, 1H), 4.12 (td, *J* = 8.6, 4.1 Hz, 1H), 3.94 (q, *J* = 9.0 Hz, 1H), 2.69 – 2.52 (m, 3H), 2.22 (dt, *J* = 15.8, 5.7 Hz, 1H). ^{13}C NMR (101 MHz, $CDCl_3$) δ 136.8, 135.9, 129.9, 75.0, 50.8, 31.3, 22.4. HRMS-(DART+) calculated for $C_7H_{13}N_6O_4$ $[M+NH_4]^+$: 245.0993, found: 245.0989.

3-Nitro-2-(4-nitro-1*H*-pyrazol-1-yl)-3-azabicyclo[3.1.0]hexane (21)

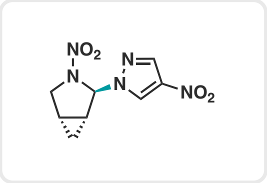

General Procedure using 10 equiv of TFA (0.2 mL) in 2.3 mL of MeCN. Yield: 80%. White solid. dr: 85:15. ^{1}H NMR (400 MHz, $CDCl_3$) δ 8.44 (s, 1H), 8.13 (s, 1H), 6.34 (s, 1H), 4.24 – 4.13 (m, 2H), 2.06 (tt, *J* = 7.9, 4.2 Hz, 1H), 1.96 (ddd, *J* = 8.1, 6.9, 4.0 Hz, 1H), 1.14 (td, *J* = 8.0, 6.2 Hz, 1H), 0.59 (dt, *J* = 6.2, 4.2 Hz, 1H). ^{13}C NMR (101 MHz, $CDCl_3$) δ 137.3, 136.0, 130.1, 75.8, 52.2, 20.3, 14.9, 9.5. HRMS-(DART+) calculated for $C_8H_{13}N_6O_4$ $[M+NH_4]^+$: 257.0993, found: 257.0998. The structure was characterized by X-ray crystallography.

Methyl 1-nitro-5-(4-nitro-1*H*-pyrazol-1-yl)pyrrolidine-2-carboxylate (22)

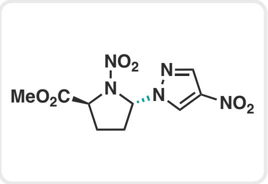

General Procedure using 10 equiv of TFA (0.2 mL) in 2.3 mL of MeCN. Yield: 35%. Pale yellow solid. dr: 92:8. ^{1}H NMR (400 MHz, Acetone-d_6) δ 8.79 (d, *J* = 0.7 Hz, 1H), 8.12 (s, 1H), 6.71 (d, *J* = 8.1 Hz, 1H), 5.02 (dd, *J* = 8.7, 1.0 Hz, 1H), 3.69 (s, 3H), 2.89 (tdd, *J* = 13.2, 8.8, 7.4 Hz, 1H), 2.65 (tt, *J* = 13.5, 7.8 Hz, 1H), 2.30 (dd, *J* = 14.0, 7.5 Hz, 1H), 2.25 – 2.18 (m, 1H). ^{13}C NMR (101 MHz, Acetone-d_6) δ 170.5, 137.4, 131.2, 76.1, 63.6, 53.3, 30.9, 30.6, 27.5. HRMS-(DART+) calculated for $C_9H_{12}N_5O_6$ $[M+H]^+$: 286.0782, found: 286.0789.

2-Nitro-3-(4-nitro-1*H*-pyrazol-1-yl)-2-azabicyclo[2.2.1]heptane (23)

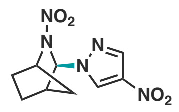

General Procedure using 10 equiv of TFA (0.2 mL) in 2.3 mL of MeCN. Yield: 58%. White solid. dr: > 20:1. ^{1}H NMR (400 MHz, $CDCl_3$) δ 8.41 (s, 1H), 8.11 (s, 1H), 5.77 (d, *J* = 1.2 Hz, 1H), 4.80 (dq, *J* = 2.6, 1.3 Hz, 1H), 3.13 (dd, *J* = 4.4, 1.8 Hz, 1H), 2.37 (dp, *J* = 11.0, 2.1 Hz, 1H), 2.07 – 1.91 (m, 2H), 1.90 – 1.80 (m, 1H), 1.68 – 1.61 (m, 2H). ^{13}C NMR (101 MHz, $CDCl_3$) δ 136.5, 136.2, 128.6, 80.6, 63.1, 43.1, 34.6, 26.1, 25.3. HRMS-(DART+) calculated for $C_9H_{12}N_5O_4$ $[M+H]^+$: 254.0884, found: 254.0889. The structure was characterized by X-ray crystallography.

2-Nitro-1-(4-nitro-1*H*-pyrazol-1-yl)-2-azaspiro[3.3]heptane (24)

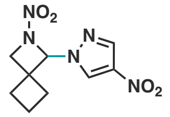

General Procedure using 10 equiv of TFA (0.2 mL) in 2.3 mL of MeCN. Yield: 33%. Pale yellow solid. ^{1}H NMR (400 MHz, $CDCl_3$) δ 8.41 (s, 1H), 8.21 (s, 1H), 6.22 (s, 1H), 4.36 (q, *J* = 9.1 Hz, 2H), 2.41 (ddd, *J* = 14.2, 9.6, 7.2 Hz, 1H), 2.34 – 2.21 (m, 1H), 2.05 (dtd, *J* = 11.9, 6.2, 3.6 Hz, 1H), 1.99 – 1.89 (m, 1H), 1.87 – 1.74 (m, 2H). ^{13}C NMR (101 MHz, $CDCl_3$) δ 137.1, 127.7, 86.1, 64.6, 41.8, 30.9, 27.9, 16.4. HRMS-(DART+) calculated for $C_9H_{12}N_5O_4$ $[M+H]^+$: 254.0884, found: 254.0884.

1-(3,3-Dimethyl-1-nitroazetidin-2-yl)-4-nitro-1*H*-pyrazole (25)

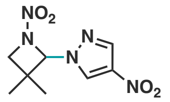

General Procedure using $LiClO_4$ (0.4 M), and 10 equiv of TFA (0.2 mL) in 2.3 mL of MeCN.

Yield: 28%. White solid. ^{1}H NMR (400 MHz, $CDCl_3$) δ 8.45 (s, 1H), 8.18 (s, 1H), 6.09 (s, 1H), 4.14 – 4.01 (m, 2H), 1.48 (s, 3H), 1.03 (s, 3H). ^{13}C NMR (101 MHz, $CDCl_3$) δ 137.0, 127.3, 86.4, 64.5, 35.7, 25.9, 21.1. HRMS-(DART+) calculated for $C_8H_{15}N_6O_4$ $[M+NH_4]^+$: 259.1149, found: 259.1146. The structure was characterized by X-ray crystallography.

1-Nitro-2-(4-nitro-1*H*-pyrazol-1-yl)piperidine (26)

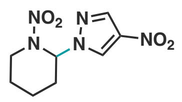

General Procedure using 10 equiv of TFA (0.2 mL) in 2.3 mL of MeCN. Yield: 21%. Yellow solid. ^{1}H NMR (400 MHz, Acetone-d_6) δ 8.82 (s, 1H), 8.17 (s, 1H), 7.02 (dd, *J* = 5.0, 2.5 Hz, 1H), 4.56 (dq, *J* = 12.2, 2.4 Hz, 1H), 3.73 (td, *J* = 12.4, 4.0 Hz, 1H), 2.52 – 2.43 (m, 1H), 2.31 (ddt, *J* = 14.8, 12.7, 4.8 Hz, 1H), 2.08 (d, *J* = 4.4 Hz, 1H), 1.87 – 1.78 (m, 2H), 1.76 – 1.67 (m, 1H). ^{13}C NMR (101 MHz, Acetone-d_6) δ 136.3, 130.7, 70.4, 46.9, 30.6, 24.3, 17.6. HRMS-(DART+) calculated for $C_8H_{15}N_6O_4$ $[M+NH_4]^+$: 259.1149, found: 259.1158.

6. Synthesis of Alkoxylated Nitramines

6.1. General Procedure for Electrochemical C–H Alkoxylation

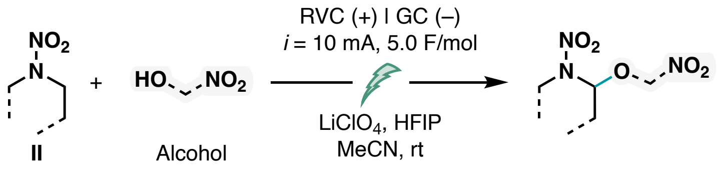

General Procedure: An oven-dried 5 mL ElectraSyn vial was charged with nitramine **II** (0.25 mmol, 1 equiv), alcohol (0.50 mmol, 2 equiv), $LiClO_4$ (1.00 mmol, 0.4 M), HFIP (0.4 mL) and anhydrous MeCN (2.1 mL). The mixture was then stirred until a clear solution formed. The vial was fitted with caps containing a RVC anode and a glassy carbon cathode, and electrolysis was conducted at a constant current of 10 mA for 5.0 F/mol. After the reaction completed, the mixture was transferred to a round-bottom flask, and the electrodes were rinsed with acetone. The solution was then concentrated *in vacuo*, and the crude mixture was purified by silica gel flash chromatography to afford the alkoxylated product. (***Safety Precaution****: The use of appropriate personal protective equipment is strongly recommended.* ***Safety Recommendatio****n: Use a blast shield when performing reactions and concentrating under reduced pressure.)*

2-Nitro-1-(2-nitroethoxy)octahydrocyclopenta[*c*]pyrrole (27)

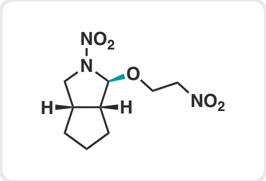

Yield: 53%. Yellow oil. dr: 75:25. ^{1}H NMR (400 MHz, $CDCl_3$) δ 5.51 (s, 1H), 4.57 – 4.47 (m, 2H), 4.35 – 4.19 (m, 2H), 3.98 (dd, *J* = 11.8, 8.9 Hz, 1H), 3.62 (dd, *J* = 11.7, 6.0 Hz, 1H), 2.96 – 2.87 (m, 1H), 2.54 (q, *J* = 8.3 Hz, 1H), 2.05 – 1.96 (m, 1H), 1.84 (dt, *J* = 12.5, 7.5 Hz, 1H), 1.71 (ddd, *J* = 14.0, 8.1, 6.3 Hz, 1H), 1.60 (dt, *J* = 7.3, 5.4 Hz, 1H), 1.53 (ddd, *J* = 9.6, 6.1, 3.8 Hz, 1H), 1.45 – 1.38 (m, 1H). ^{13}C NMR (101 MHz, $CDCl_3$) δ 95.6, 75.2, 66.0, 55.3, 49.6, 39.6, 32.3, 29.6, 25.1. HRMS-(DART+) calculated for $C_9H_{19}N_4O_5$ $[M+NH_4]^+$: 263.1350, found: 263.1355.

2-Nitro-1-(2-nitroethoxy)octahydro-1*H*-isoindole (28)

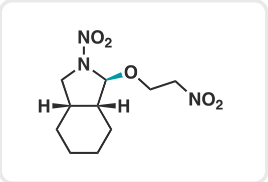

Yield: 47%. White solid. dr: 85:15. ^{1}H NMR (400 MHz, $CDCl_3$) δ 5.36 (s, 1H), 4.52 (t, *J* = 4.9 Hz, 2H), 4.36 – 4.20 (m, 2H), 3.87 – 3.77 (m, 2H), 2.68 (ddq, *J* = 13.9, 9.1, 4.6 Hz, 1H), 2.09 (dt, *J* = 11.6, 5.4 Hz, 1H), 1.71 – 1.52 (m, 5H), 1.38 – 1.16 (m, 2H), 0.99 (qd, *J* = 13.4, 3.9 Hz, 1H). ^{13}C NMR (101 MHz, $CDCl_3$) δ 95.9, 75.1, 65.5, 51.3, 43.7, 32.9, 24.2, 23.9, 23.8 20.7. HRMS-(DART+) calculated for $C_{10}H_{21}N_4O_5$ $[M+NH_4]^+$: 277.1507, found: 277.1501.

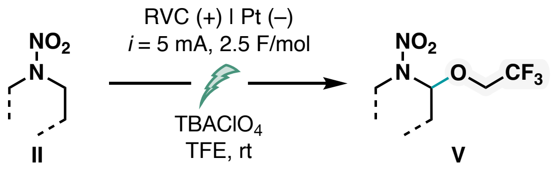

General Procedure: An oven-dried 5 mL ElectraSyn vial was charged with nitramine **II** (0.25 mmol, 1 equiv), $TBAClO_4$ (1.00 mmol, 0.4 M), and trifluoroethanol (TFE, 2.5 mL). The mixture was then stirred until a clear solution formed. The vial was fitted with caps containing a RVC anode and a Pt foil cathode, and electrolysis was conducted at a constant current of 5 mA for 2.5 F/mol. After the reaction completed, the mixture was transferred to a round-bottom flask, and the electrodes were rinsed with acetone. The solution was then concentrated *in vacuo*, and the crude mixture was purified by silica gel flash chromatography to afford the alkoxylated product **V**. (***Safety Precaution****: The use of appropriate personal protective equipment is strongly recommended.* ***Safety Recommendation****: Use a blast shield when performing reactions and concentrating under reduced pressure.)*

1-Nitro-2-(2,2,2-trifluoroethoxy)pyrrolidine (34)

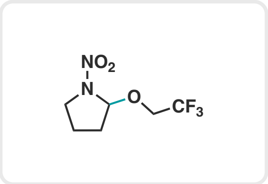

Yield: 80%. Colorless oil. ^1H NMR (400 MHz, $CDCl_3$) δ 5.74 (d, *J* = 4.7 Hz, 1H), 4.28 – 4.08 (m, 2H), 3.99 – 3.90 (m, 1H), 3.76 (td, *J* = 11.0, 6.4 Hz, 1H), 2.24 (tt, *J* = 10.3, 7.0 Hz, 1H), 2.15 – 2.01 (m, 3H). ^{19}F NMR (376 MHz, $CDCl_3$) δ -74.83. ^{13}C NMR (101 MHz, $CDCl_3$) δ 123.7 (q, *J* = 278.5 Hz), 91.2, 67.6 (q, *J* = 34.7 Hz), 50.2, 32.1, 21.7. HRMS-(DART+) calculated for $C_6H_{13}F_3N_3O_3$ $[M+NH_4]^+$: 232.0909, found: 232.0912.

2-Nitro-1-(2,2,2-trifluoroethoxy)octahydro-1*H*-isoindole (V-1)

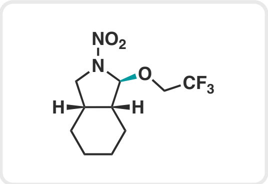

Yield: 91%. Colorless oil. dr: 95:5. ^1H NMR (400 MHz, $CDCl_3$) δ 5.38 (s, 1H), 4.28 – 4.06 (m, 2H), 3.89 – 3.76 (m, 2H), 2.76 (tq, *J* = 9.1, 4.6 Hz, 1H), 2.22 (dt, *J* = 11.6, 5.5 Hz, 1H), 1.76 – 1.54 (m, 5H), 1.42 – 1.20 (m, 2H), 1.13 – 0.96 (m, 1H). ^{19}F NMR (376 MHz, $CDCl_3$) δ -74.82. ^{13}C NMR (126 MHz, $CDCl_3$) δ 123.7 (q, *J* = 278.5 Hz), 96.5, 67.7 (q, *J* = 34.6 Hz), 51.3, 43.8, 32.8, 24.2, 23.9, 23.8, 20.7. HRMS-(DART+) calculated for $C_{10}H_{19}F_3N_3O_3$ $[M+NH_4]^+$: 286.1378, found: 286.1375.

7. Synthesis of Nitramines Containing Energetic Functionalities

7.1. General Procedures for Electrochemical C–H Functionalizations

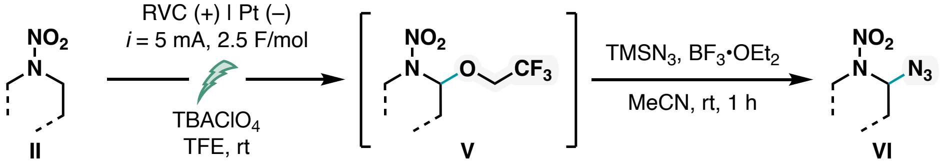

General Procedure: 1) An oven-dried 5 mL ElectraSyn vial was charged with nitramine **II** (0.25 mmol, 1 equiv), $TBAClO_4$ (1.00 mmol, 0.4 M), and trifluoroethanol (TFE, 2.5 mL). The mixture was then stirred until a clear solution formed. The vial was fitted with caps containing a RVC anode and a Pt foil cathode, and electrolysis was conducted at a constant current of 5 mA for 2.5 F/mol. After the reaction completed, the mixture was transferred to a round-bottom flask, and the electrodes were rinsed with acetone. The solution was then concentrated *in vacuo* to give the *N*,*O*-acetal **V**, which was used directly in the next step without purification.

2) A 10 mL round-bottom flask was charged with the *N*,*O*-acetal **V** (1 equiv), $TMSN_3$ (3 equiv), $BF_3{\cdot}OEt_2$ (3 equiv), and anhydrous MeCN (0.1 M). The mixture was stirred at room temperature for 1 h. After the reaction completed, the crude mixture was quenched by the addition of saturated aqueous solution of $NaHCO_3$, followed by extraction with ethyl acetate and the combined organic layers were washed with brine, dried over anhydrous Na_2SO_4, filtered, and the mixture was concentrated *in vacuo*. The residue was purified via a flash chromatography to deliver the azidation product **VI**. (***Safety Precaution****: The use of appropriate personal protective equipment is strongly recommended.* ***Safety Recommendatio****n: Use a blast shield when performing reactions and concentrating under reduced pressure.)*

1-Azido-2-nitrooctahydro-1*H*-isoindole (29)

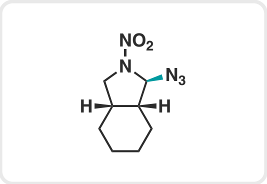

Yield: 89%. White solid. dr: 93:7. ^{1}H NMR (400 MHz, Acetone-d_6) δ 5.73 (d, J = 2.8 Hz, 1H), 3.92 (dd, J = 11.3, 7.8 Hz, 1H), 3.83 (dd, J = 11.3, 8.6 Hz, 1H), 2.56 (ddt, J = 13.5, 8.2, 5.3 Hz, 1H), 2.21 – 2.12 (m, 1H), 1.79 (ddq, J = 10.6, 5.3, 2.9 Hz, 1H), 1.71 – 1.26 (m, 7H). ^{13}C NMR (101 MHz, Acetone-d_6) δ 81.7, 52.6, 44.6, 34.1, 25.3, 25.0, 23.9, 21.9. HRMS-(DART+) calculated for $C_8H_{14}N_3O_2$ $[M-N_2+H]^+$: 184.1086, found: 184.1090.

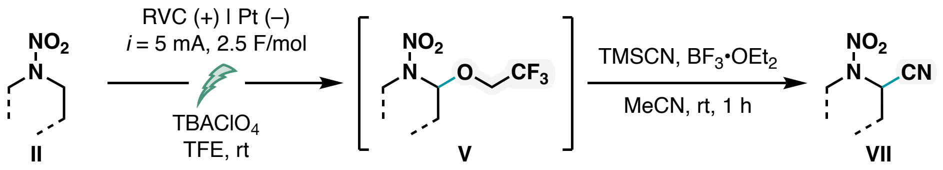

General Procedure: 1) An oven-dried 5 mL ElectraSyn vial was charged with nitramine **II** (0.25 mmol, 1 equiv), $TBAClO_4$ (1.00 mmol, 0.4 M), and trifluoroethanol (TFE, 2.5 mL). The mixture was then stirred until a clear solution formed. The vial was fitted with caps containing a RVC anode and a Pt foil cathode, and electrolysis was conducted at a constant current of 5 mA for 2.5 F/mol. After the reaction completed, the mixture was transferred to a round-bottom flask, and the electrodes were rinsed with acetone. The solution was then concentrated *in vacuo* to give the *N*,*O*-acetal **V**, which was used directly in the next step without purification.

2) A 10 mL round-bottom flask was charged with the *N*,*O*-acetal **V** (1 equiv), TMSCN (3 equiv), $BF_3 \cdot OEt_2$ (3 equiv), and anhydrous MeCN (0.1 M). The mixture was stirred at room temperature for 1 h. After the reaction completed, the crude mixture was quenched by the addition of saturated aqueous solution of $NaHCO_3$, followed by extraction with ethyl acetate and the combined organic layers were washed with brine, dried over anhydrous Na_2SO_4, filtered, and the mixture was concentrated *in vacuo*. The residue was purified via a flash chromatography to deliver the cyanation product **VII**. (***Safety Precaution****: The use of appropriate personal protective equipment is strongly recommended.* ***Safety Recommendation****: Use a blast shield when performing reactions and concentrating under reduced pressure.)*

2-Nitrooctahydro-1*H*-isoindole-1-carbonitrile (VII-1)

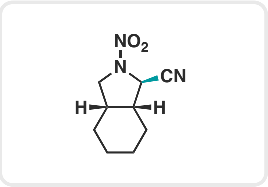

Yield: 87%. White solid. dr: 85:15. ^{1}H NMR (500 MHz, $CDCl_3$) δ 4.71 (d, *J* = 3.9 Hz, 1H), 3.95 (dd, *J* = 11.3, 7.4 Hz, 1H), 3.86 (dd, *J* = 11.3, 7.7 Hz, 1H), 2.73 – 2.59 (m, 2H), 1.81 (dtd, *J* = 12.2, 5.3, 3.3 Hz, 1H), 1.74 – 1.58 (m, 2H), 1.56 – 1.35 (m, 5H). ^{13}C NMR (126 MHz, $CDCl_3$) δ 116.0, 54.9, 52.9, 43.2, 35.3, 25.4, 24.8, 22.9, 21.3. HRMS-(DART+) calculated for $C_9H_{14}N_3O_2$ $[M+H]^+$: 196.1081, found: 196.1081.

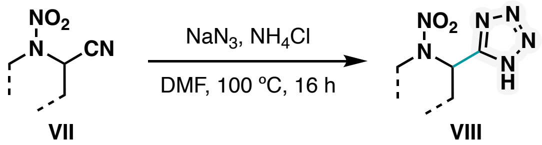

General Procedure: 1) A 10 mL round-bottom flask was charged with nitramine **VII** (0.15 mmol, 1 equiv), NaN_3 (3 equiv), NH_4Cl (3 equiv), and anhydrous DMF (0.2 M). The mixture was stirred at 100 ºC for 16 h. After the reaction completed, the crude mixture was cooled to room temperature and diluted with ethyl acetate. The resulting solution was filtered through a short Celite plug, washed with additional ethyl acetate. The filtrate was concentrated *in vacuo* at 50 ºC. Mesitylene was then added as an internal standard to the crude mixture for NMR yield determination of product **VIII**. (***Safety Precaution****: The use of appropriate personal protective equipment is strongly recommended.* ***Safety Recommendatio****n: Use a blast shield when performing reactions and concentrating under reduced pressure.)*

2-Nitro-1-(1*H*-tetrazol-5-yl)octahydro-1*H*-isoindole (30)

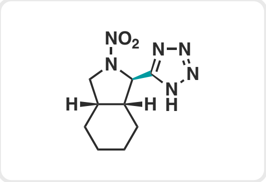

This compound was not purified due to lability and safety concerns.

NMR yield: 78%. dr: 85:15. ^{1}H NMR (400 MHz, $CDCl_3$) δ 5.49 (d, J = 4.2 Hz, 1H), 4.03 (dd, J = 11.6, 7.4 Hz, 1H), 3.94 (dd, J = 11.6, 7.7 Hz, 1H), 2.73 (q, J = 5.8 Hz, 1H), 2.62 (dq, J = 10.0, 5.1 Hz, 1H), 1.88 – 1.78 (m, 1H), 1.65 (dq, J = 10.9, 5.7 Hz, 2H), 1.60 – 1.36 (m, 5H). ^{13}C NMR (101 MHz, $CDCl_3$) δ 61.3, 53.6, 43.9, 34.6, 25.9, 25.2, 23.1, 21.8. HRMS-(DART+) calculated for $C_9H_{15}N_6O_2$ $[M+H]^+$: 239.1251, found: 239.1252.

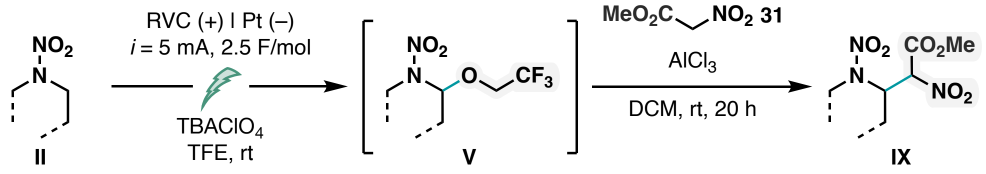

General Procedure: 1) An oven-dried 5 mL ElectraSyn vial was charged with nitramine **II** (0.25 mmol, 1 equiv), $TBAClO_4$ (1.00 mmol, 0.4 M), and trifluoroethanol (TFE, 2.5 mL). The mixture was then stirred until a clear solution formed. The vial was fitted with caps containing a RVC anode and a Pt foil cathode, and electrolysis was conducted at a constant current of 5 mA for 2.5 F/mol. After the reaction completed, the mixture was transferred to a round-bottom flask, and the electrodes were rinsed with acetone. The solution was then concentrated *in vacuo* to give the *N*,*O*-acetal **V**, which was used directly in the next step without purification.

2) A 10 mL round-bottom flask was charged with the *N*,*O*-acetal **V** (1 equiv), nitroacetate **31** (3 equiv), $AlCl_3$ (3 equiv), and anhydrous DCM (0.1 M). The mixture was stirred at room temperature for 20 h. After the reaction completed, the crude mixture was quenched by the addition of saturated aqueous solution of $NaHCO_3$, followed by extraction with DCM and the combined organic layers were washed with brine, dried over anhydrous Na_2SO_4, filtered, and the mixture was concentrated *in vacuo*. The residue was purified via a flash chromatography to deliver the alkylation product **IX**. (***Safety Precaution***: *The use of appropriate personal protective equipment is strongly recommended.* ***Safety Recommendation***: *Use a blast shield when performing reactions and concentrating under reduced pressure.)*

Methyl 2-nitro-2-(2-nitrooctahydro-1*H*-isoindol-1-yl)acetate (32)

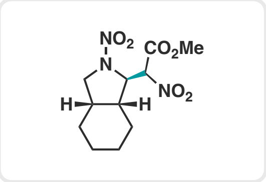

Yield: 21%. Colorless oil. dr: 65:35. ^1H NMR (500 MHz, $CDCl_3$) δ 5.60 (dd, *J* = 6.4, 5.4 Hz, 1H), 4.76 (ddd, *J* = 17.0, 5.4, 2.2 Hz, 1H), 3.91 – 3.81 (m, 5H), 2.44 (dddd, *J* = 14.4, 8.1, 5.4, 3.0 Hz, 1H), 2.35 (dtt, *J* = 11.3, 5.6, 2.7 Hz, 1H), 1.92 (td, *J* = 11.8, 6.1 Hz, 1H), 1.76 – 1.58 (m, 3H), 1.53 – 1.19 (m, 4H). ^{13}C NMR (126 MHz, $CDCl_3$) δ 162.8, 85.9, 67.5, 54.2, 52.2, 40.1, 33.4, 27.9, 24.2, 24.0, 20.8. HRMS-(DART+) calculated for $C_{11}H_{21}N_4O_6$ $[M+NH_4]^+$: 305.1456, found: 305.1468.

8. Cyclic Voltammetry Analysis

Cyclic voltammetry (CV) studies were measured with a BASi Epsilon potentiostat. The solution of substrate (10 mM) was analyzed with 0.1 M $TBAClO_4$ in anhydrous MeCN at room temperature, employing a scan rate = 100 mV/s. The supporting electrolyte ($TBAClO_4$) was recrystallized in ethyl acetate and dried under vacuum overnight. The working electrode is a 3 mm diameter glassy carbon, polished with 1.0, 0.3 and 0.05 μm aluminum oxide. The counter electrode is a platinum wire that was previous burnt with a butane torch. The reference electrode consisted of $Ag/AgClO_4$. After each set of scans, ferrocene was added to reference the final potential to ferrocene/ferrocenium redox couple ($Fc^{0/+}$).

Working electrode: Glassy C
Counter electrode: Pt
Reference electrode: Ag/Ag^+
Solvent: MeCN
Substrate concentration: 10 mM
Bu_4NClO_4 concentration: 0.1 M
υ = 100 mV/s

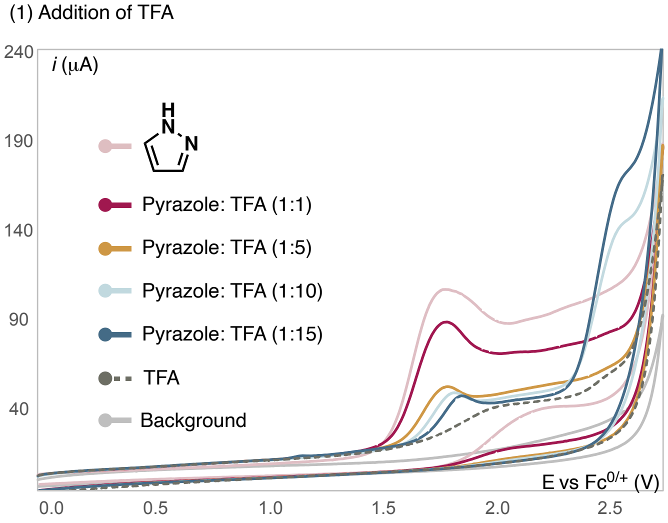

Working electrode: Glassy C
Counter electrode: Pt
Reference electrode: Ag/Ag^+
Solvent: MeCN
Substrate concentration: 10 mM
Bu_4NClO_4 concentration: 0.1 M
υ = 100 mV/s

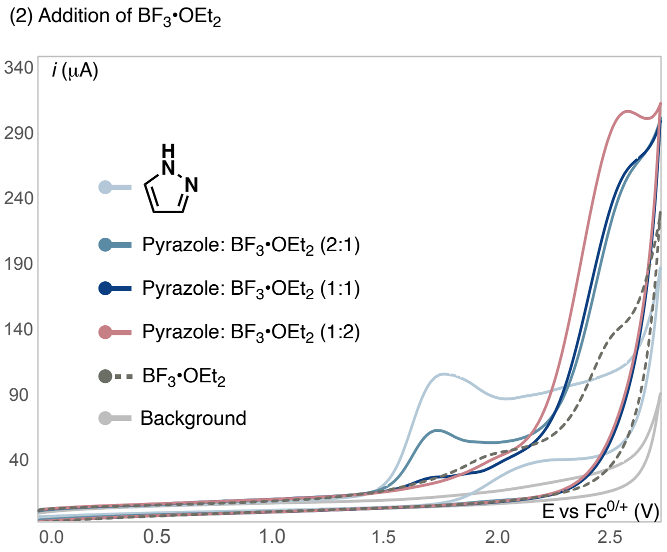

Figure S2. Cyclic Voltammetry of Acid Effect on C–H Azolation

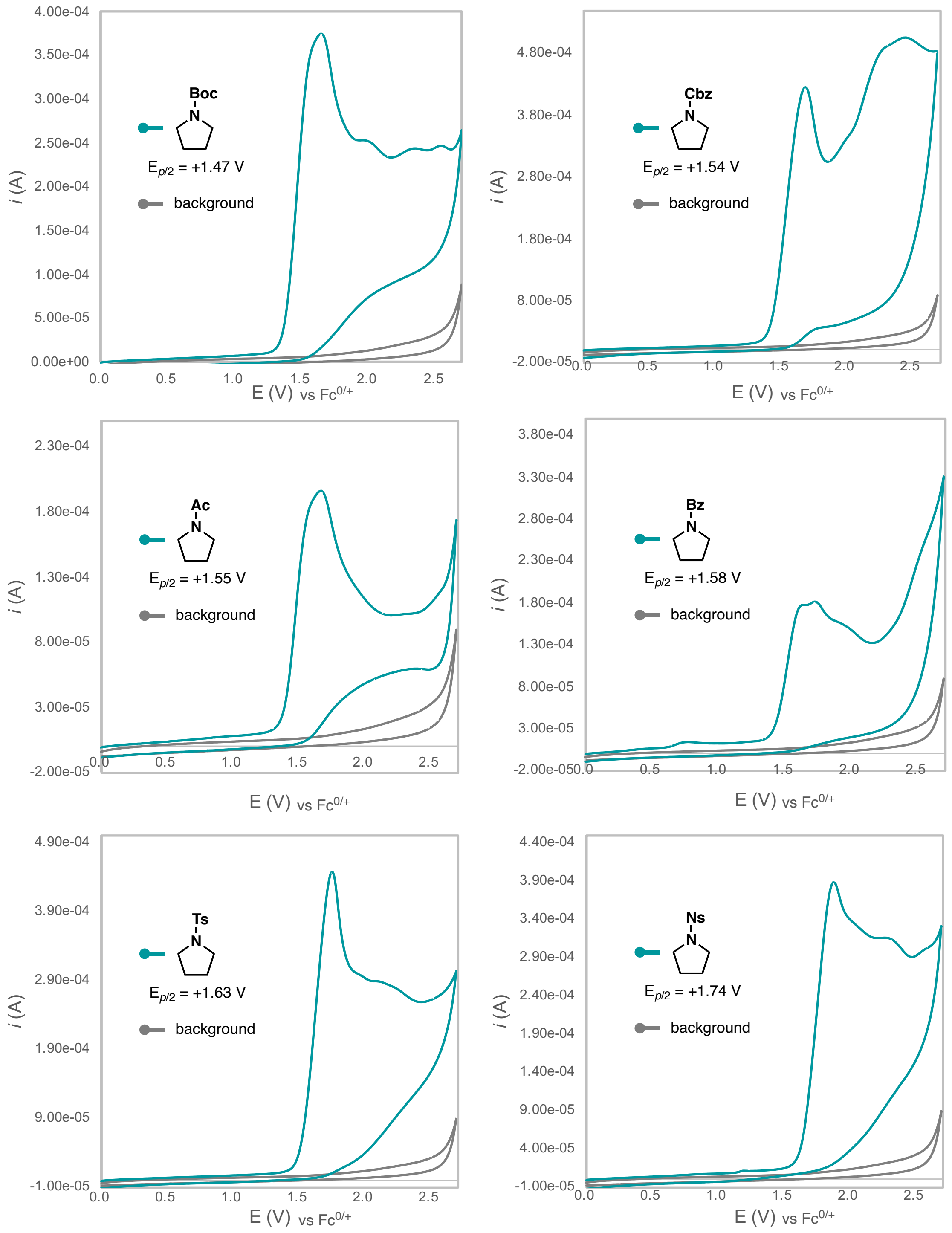

Figure S3. Cyclic Voltammetry of *N*-Substituted Pyrrolidines

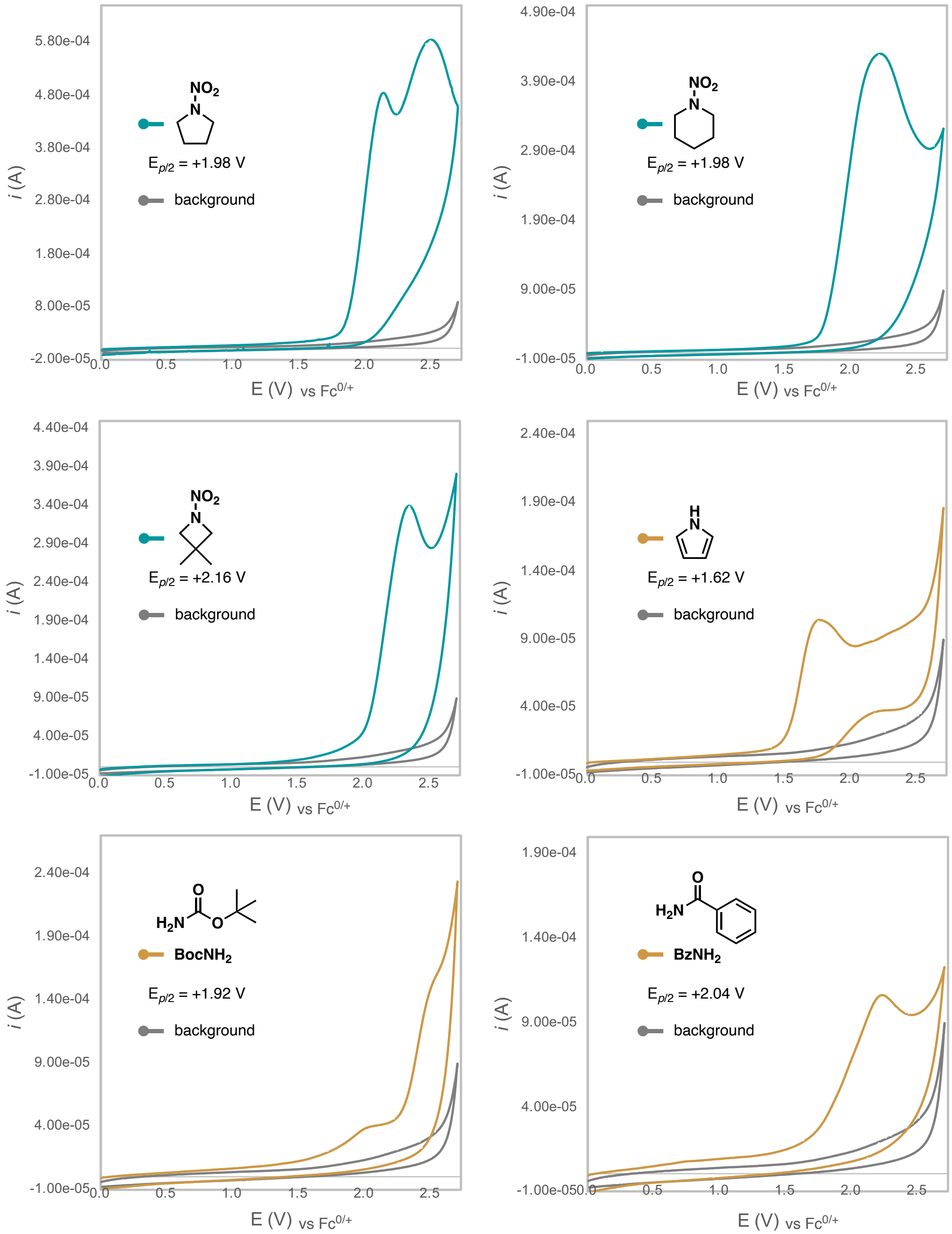

Figure S4. Cyclic Voltammetry of Nitramines and *N*-Nucleophiles

Scheme S2. Potential–Time (E/*t*) Curve of the Model Reaction with Anodic Potential Range of 2.17–2.30 V

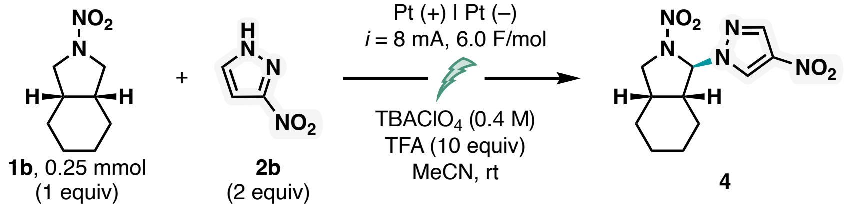

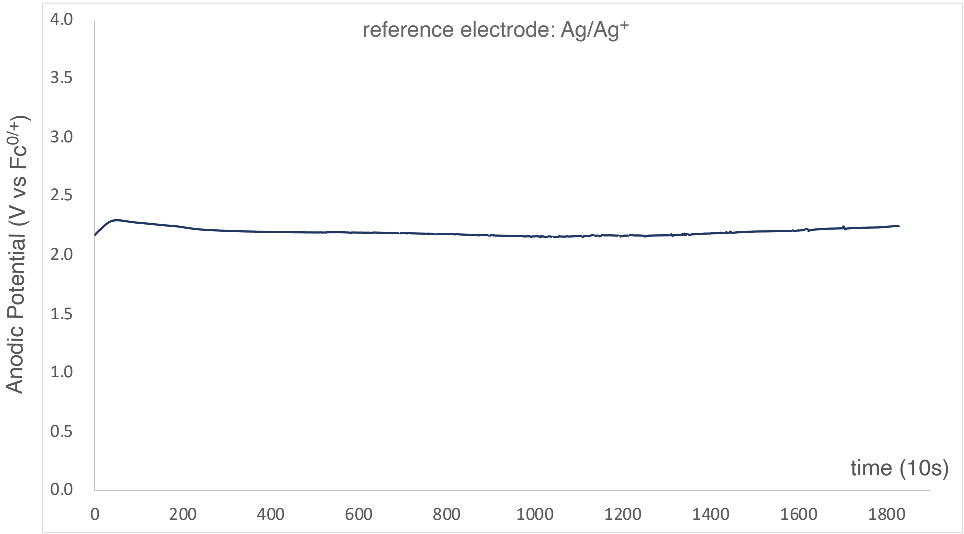

9. Electrochemical Flow System

Electrochemical flow experiments were performed using a stand-alone Vapourtec Ion Electrochemical Reactor with an Aim-TTi EX354RD Dual Power Supply from Thurlby Thandar Instruments Ltd. A Chemyx Fusion 100 Touch Syringe Pump or a Vapourtec SF-10 Reagent Pump were used in the flow setup. Electrode materials employed were platinum (Pt) and glassy carbon (GC) purchased from Goodfellow. The electrodes (5 x 5 cm^2) were separated by a 0.125 mm, 0.25 mm, or 0.5 mm FEP spacer or a 1.0 mm PTFE spacer resulting in a reactor volume of 0.15 mL, 0.3 mL, 0.6 mL or 1.2 mL, respectively, with an exposed electrode surface area of 12 cm^2.

9.1. General Procedures for Electroflow C–H Functionalizations

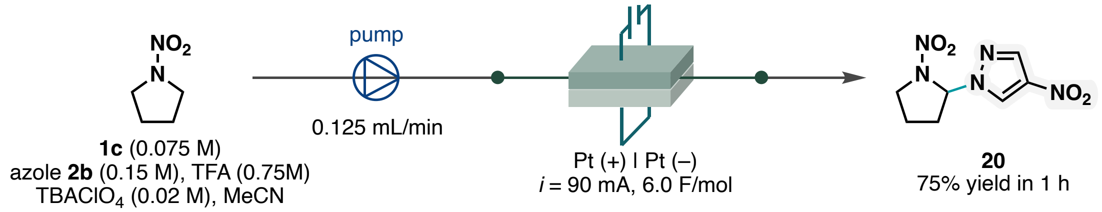

General Procedure for Azolation: The electrolysis was performed in an undivided cell using a Vapourtec Ion Electrochemical Reactor (FEP spacer = 0.125 mm, reactor volume = 0.15 mL), employing a platinum electrode as the anode and a platinum electrode as the cathode. A solution of nitramine **1c** (0.075 M, 1 equiv), azole **2b** (0.15 M, 2 equiv), TFA (0.75 M, 10 equiv) and $TBAClO_4$ (0.02 M) in MeCN was pumped with a flow rate of 0.125 mL/min into the electrochemical reactor and was electrolyzed under constant current conditions (90 mA, j = 7.5 mA/cm^2, 6 F/mol). The first one and a half reactor volumes were disposed to ensure a steady state of the system had been reached. After collection for a known period, the residue was concentrated *in vacuo* and purified via a flash chromatography to deliver the azolated product **20** in 75% yield within 1 h.

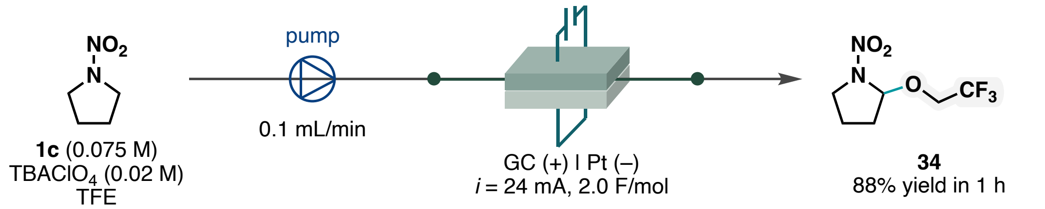

General Procedure for Alkoxylation: The electrolysis was performed in an undivided cell using a Vapourtec Ion Electrochemical Reactor (FEP spacer = 0.125 mm, reactor volume = 0.15 mL), employing a glassy carbon electrode as the anode and a platinum electrode as the cathode. A

solution of nitramine **1c** (0.075 M, 1 equiv) and $TBAClO_4$ (0.02 M) in trifluoroethanol (TFE) was pumped with a flow rate of 0.1 mL/min into the electrochemical reactor and was electrolyzed under constant current conditions (24 mA, j = 2 mA/cm^2, 2 F/mol). The first one and a half reactor volumes were disposed to ensure a steady state of the system had been reached. After collection for a known period, the residue was concentrated *in vacuo* and purified via a flash chromatography to deliver the alkoxylated product **34** in 88% yield within 1 h.

9.2. Optimization of Electroflow C–H Azolation

Table S8. Optimization of Electroflow C–H Azolation

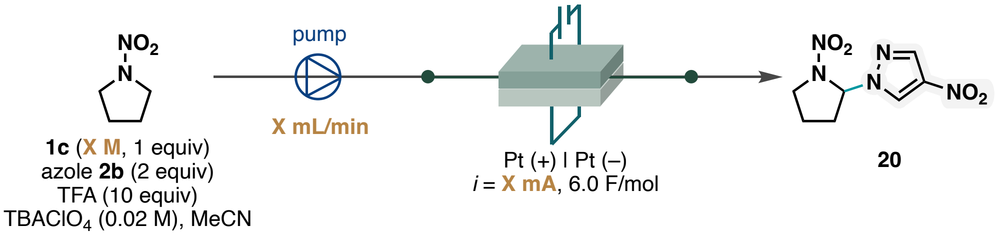

Entry		[**1c**] (M)	Flow Rate (mL/min)	Current (mA)	Yield of **20**
1	flow rate effect	0.075	0.150	108	68%
2		0.075	0.125	90	77%
3		0.075	0.100	72	63%
4		0.075	0.075	54	63%
5		0.075	0.050	36	53%
6	concentration effect	0.025	0.125	30	66%
7		0.050	0.125	60	73%
8		0.075	0.125	90	77%
9		0.100	0.125	120	10%

9.3. Optimization of Electroflow C–H Alkoxylation

Table S9. Optimization of Electroflow C–H Alkoxylation

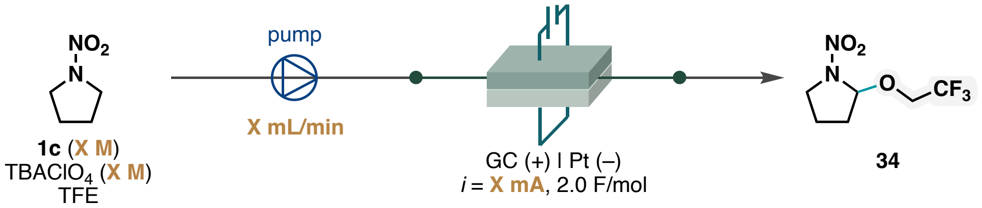

Entry		[**1c**] (M)	[TBAClO$_4$] (M)	Flow Rate (mL/min)	Current (mA)	Yield of **34**
1	flow rate effect	0.075	0.020	0.050	12	88%
2		0.075	0.020	0.075	18	85%
3		0.075	0.020	0.100	24	85%
4		0.075	0.020	0.125	30	75%
5		0.075	0.020	0.150	36	74%
6	concentration effect	0.025	0.100	0.050	4	52%
7		0.050	0.100	0.050	8	76%
8		0.075	0.100	0.050	12	75%
9		0.100	0.100	0.050	16	61%
10	electrolyte concentration effect	0.075	0.200	0.050	12	44%
11		0.075	0.100	0.050	12	75%
12		0.075	0.075	0.050	12	72%
13		0.075	0.050	0.050	12	85%
14		0.075	0.025	0.050	12	86%
15		0.075	0.020	0.050	12	88%
16		0.075	0.010	0.050	12	85%
17		0.075	0.005	0.050	12	82%
18		0.075	0	0.050	12	5%

Table S10. Optimization of Interelectrode Distance for Electroflow C–H Alkoxylation

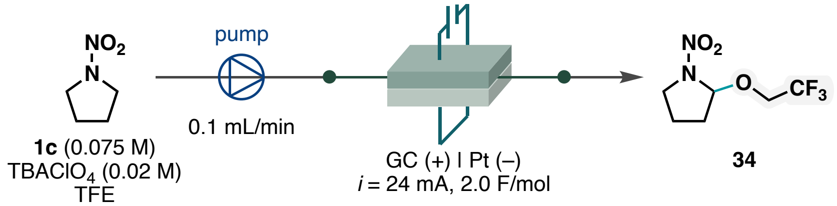

Entry	Interelectrode Distance (mm)	Yield of **34**
1	1.000	67%
2	0.500	85%
3	0.250	85%
4	0.125	93%

Table S11. Time Dependent Study on Electroflow C–H Alkoxylation

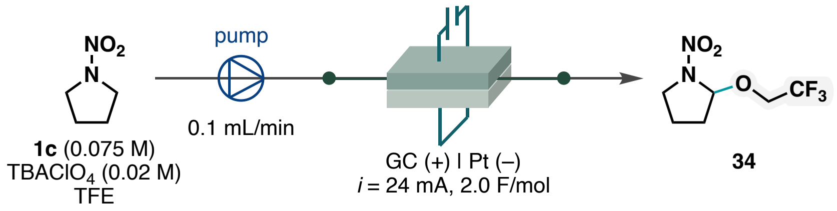

Entry	t (min)	$\bar{V}$ (V)	Yield of **34**
1	0	3.15	100%
2	10	3.26	100%
3	20	3.34	100%
4	30	3.44	87%
5	60	3.79	73%
6	90	3.84	73%
7	120	3.84	68%
8	150	3.86	67%
9	180	3.86	72%
10	210	3.85	74%

9.4. Experimental Setup for Electroflow System

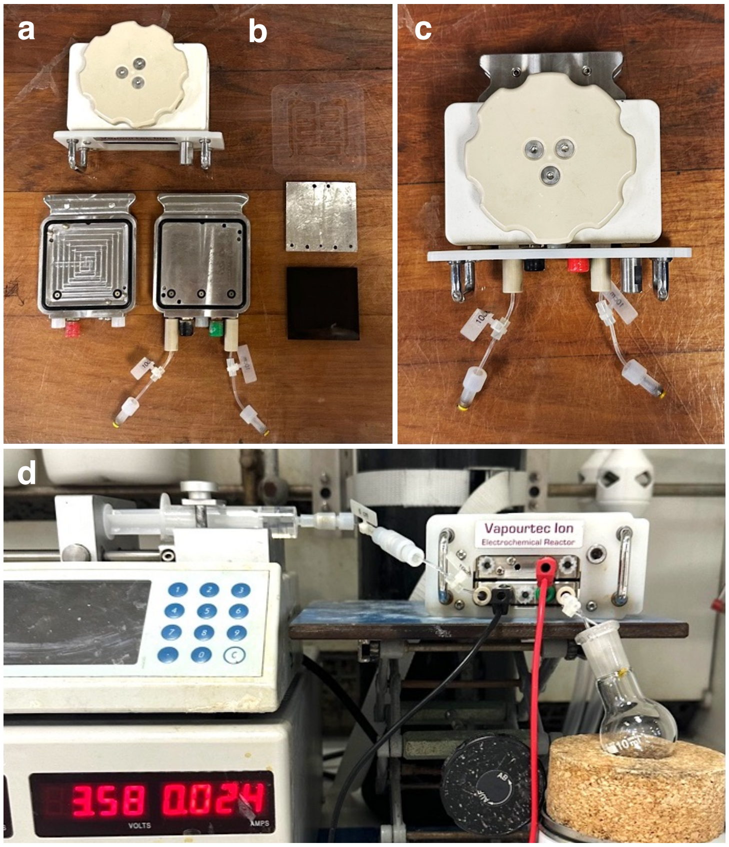

Figure S5. Electroflow Setup.

(a) An open Vapourtec Ion Electrochemical Reactor.

(b) 0.125 mm FEP spacer, Pt electrode and GC electrode.

(c) A closed Vapourtec Ion Electrochemical Reactor.

(d) Electroflow Setup.

9.5. Batch vs. Flow Processes

STY (Space–Time Yield)

$$\text{STY} = \frac{\text{mass of product (g)}}{\text{reaction volume (L)} \times \text{reaction time (h)}}$$

For flow reactions, the reactor volume is the *internal volume of the flow reactor*, and time corresponds to *residence time* multiplied by flow cycles if relevant.

Batch: Compound **20** (STY = 3.68 $gL^{-1}h^{-1}$); Compound **4** (STY = 4.96 $gL^{-1}h^{-1}$)
Flow: Compound **20** (STY = 640 $gL^{-1}h^{-1}$)
Telescoped: Compound **4** (STY = 0.17 $gL^{-1}h^{-1}$)

Solvent Usage per Gram of Product

$$\text{Solvent usage} = \frac{\text{total solvent volume used (mL)}}{\text{mass of product (g)}}$$

Batch: Compound **20** (Solvent Usage = 54.34 mLg^{-1}); Compound **4** (Solvent Usage = 40.32 mLg^{-1})
Flow: Compound **20** (Solvent Usage = 78.12 mLg^{-1})
Telescoped: Compound **4** (Solvent Usage = 42.95 mLg^{-1})

Electrolyte Usage per Gram of Product

$$\text{Electrolyte usage} = \frac{\text{mass of electrolyte used (g)}}{\text{mass of product (g)}}$$

Batch: Compound **20** (Electrolyte Usage = 7.43); Compound **4** (Electrolyte Usage = 5.51)
Flow: Compound **20** (Electrolyte Usage = 0.53)
Telescoped: Compound **4** (Electrolyte Usage = 5.87)

10. Measurements of Thermal Properties

10.1. DSC Thermograms

Differential scanning calorimetry (DSC) thermograms were recorded on TA DSC SC Q20 V24.11 Build 124. The experiments were carried out under a nitrogen purge of 50 mL/min at a heating rate of 10 °C/min, covering a temperature range of 25 ºC to 300 ºC. The samples were analyzed in TzeroTM hermetic aluminum DSC pans. The instruments were calibrated using an indium standard, the DSC thermograms were analyzed using Smart Lab studio II x64.

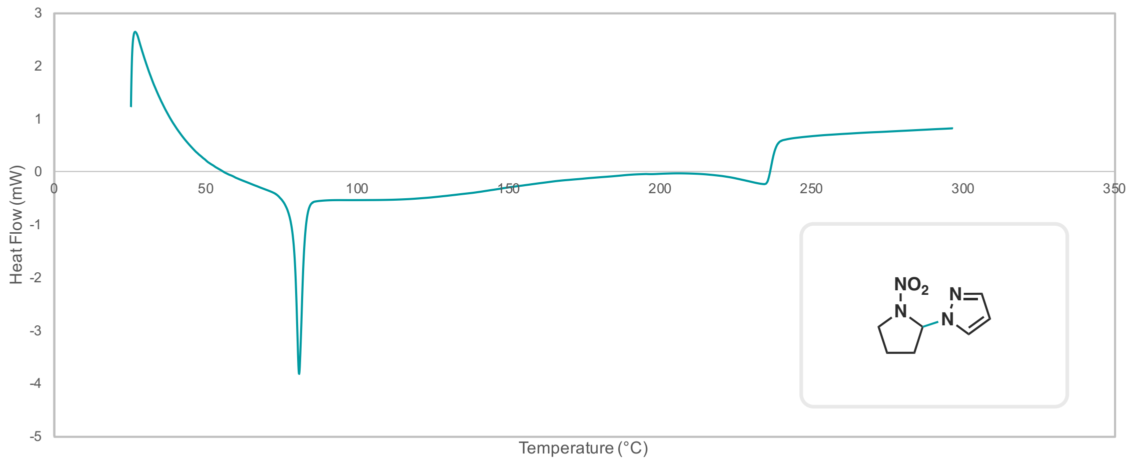

Figure S6. DSC Thermogram of **19**. The Onset Melting Temperature is 74 ºC.

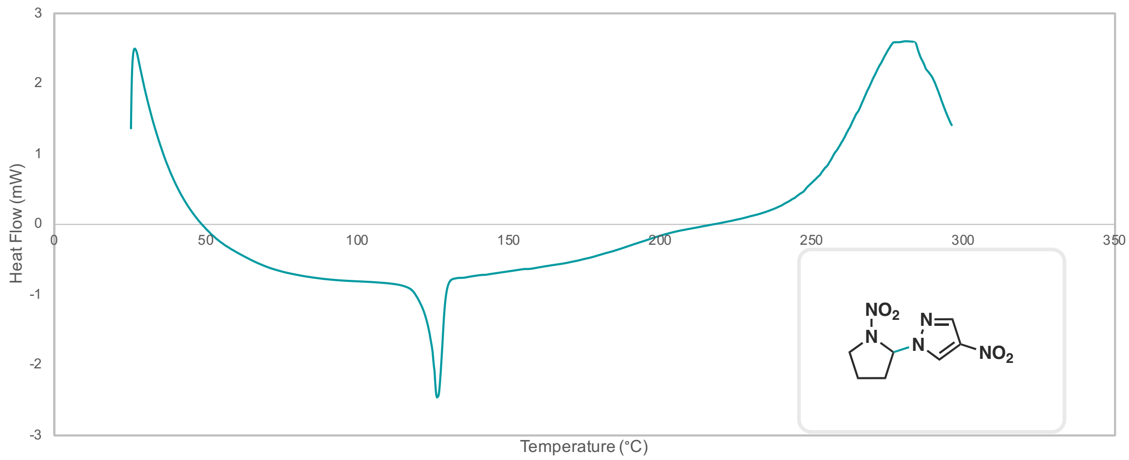

Figure S7. DSC Thermogram of **20**. The Onset Melting Temperature is 117 ºC.

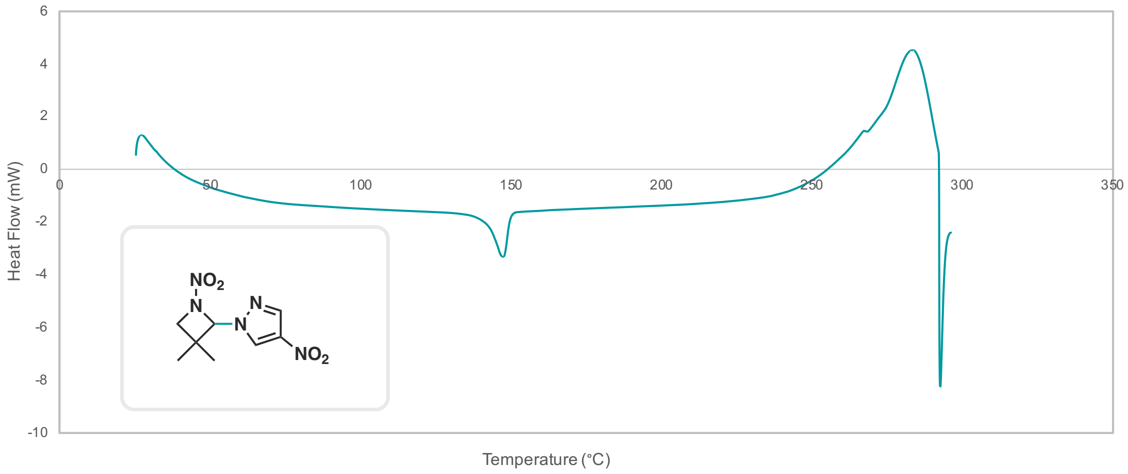

Figure S8. DSC Thermogram of **25**. The Onset Melting Temperature is 137 ºC.

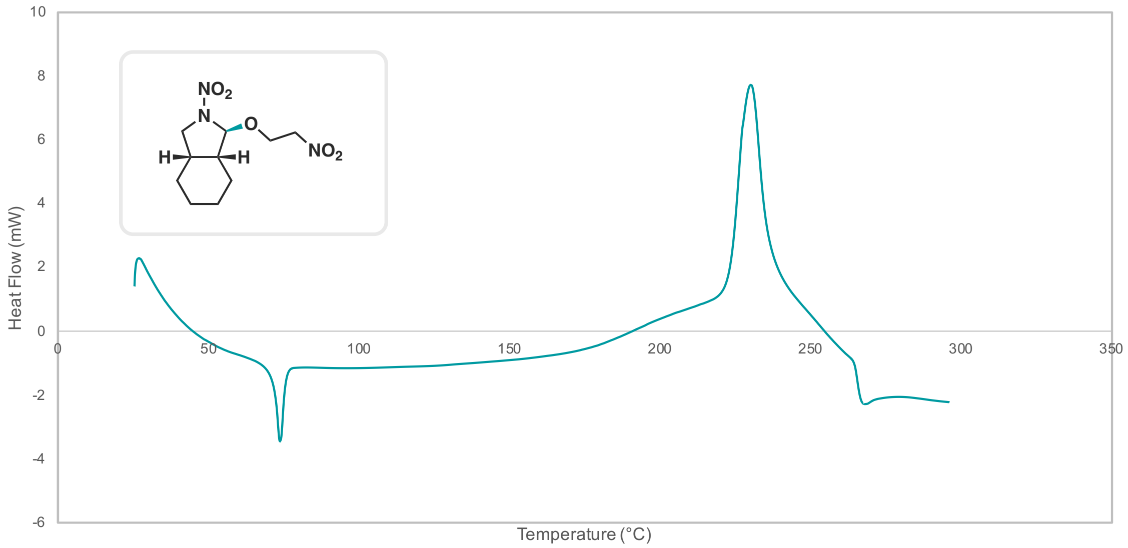

Figure S9. DSC Thermogram of **28**. The Onset Melting Temperature is 69 ºC.

10.2. TGA Thermograms

Thermogravimetric analysis (TGA) thermograms were recorded on TA Discovery TGA 5500. The experiments were conducted in platinum TGA sample pans under air purge of 50 mL/min with a heating rate of 10 ºC/min, covering a temperature range of 25 ºC to 500 ºC. The instrument was calibrated using the Curie points of alumel and nickel standards and the TGA thermograms were analyzed using TA Instruments Trios.

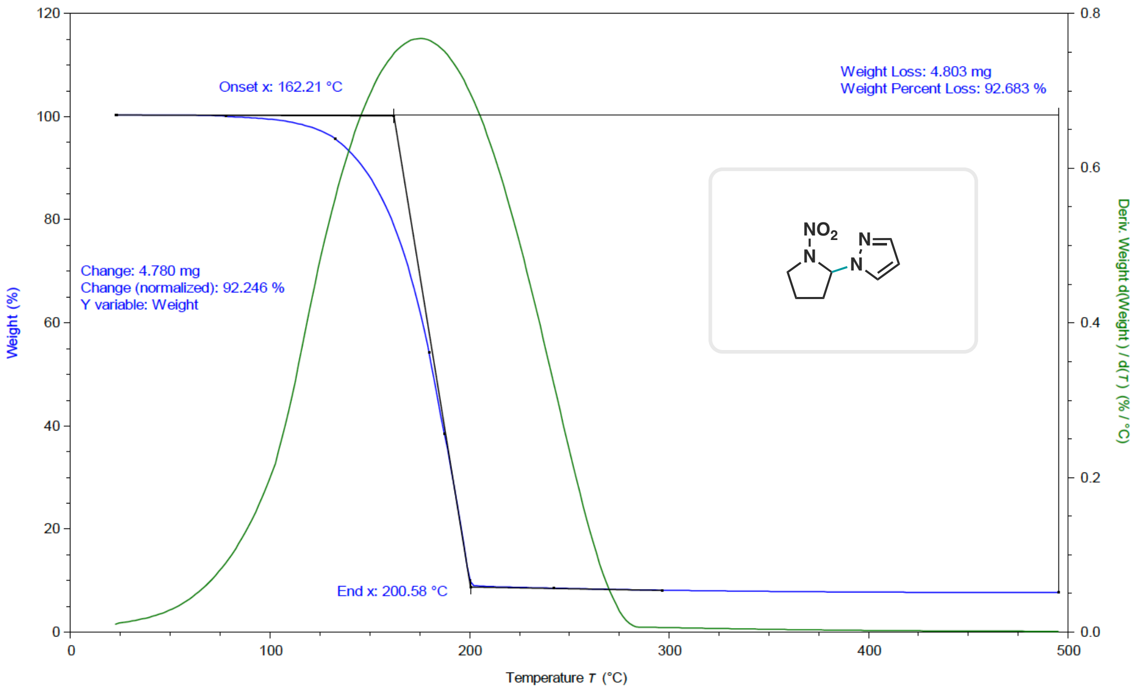

Figure S10. TGA Thermogram of **19**. The Onset Decomposition Temperature is 162 ºC.

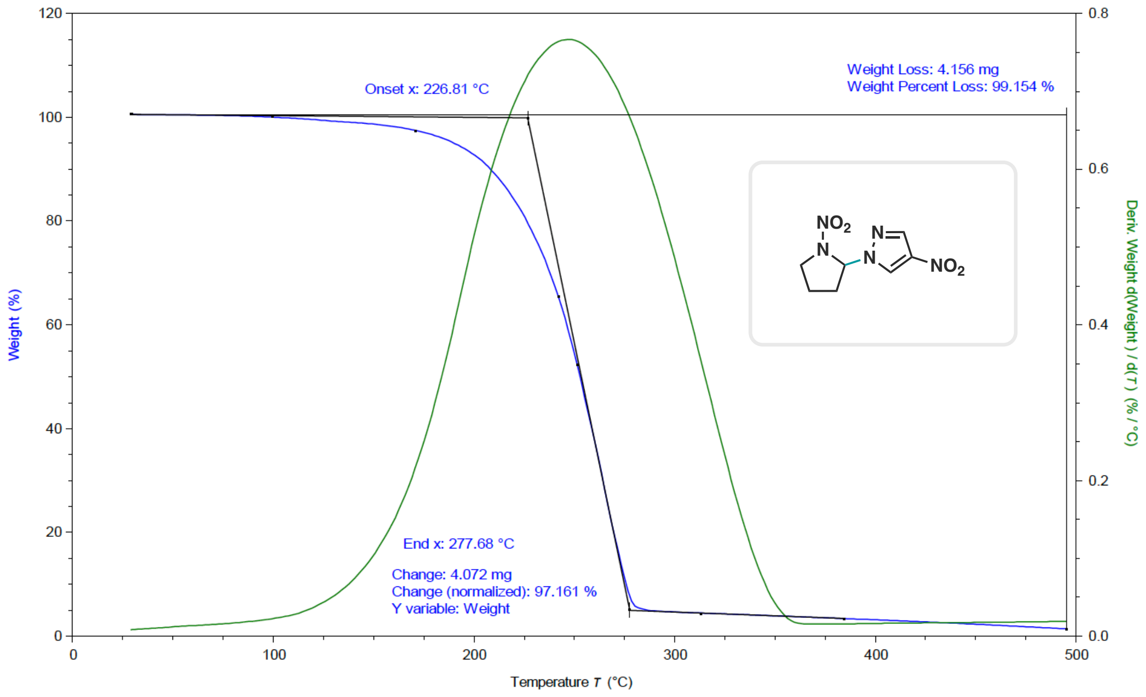

Figure S11. TGA Thermogram of **20**. The Onset Decomposition Temperature is 227 ºC.

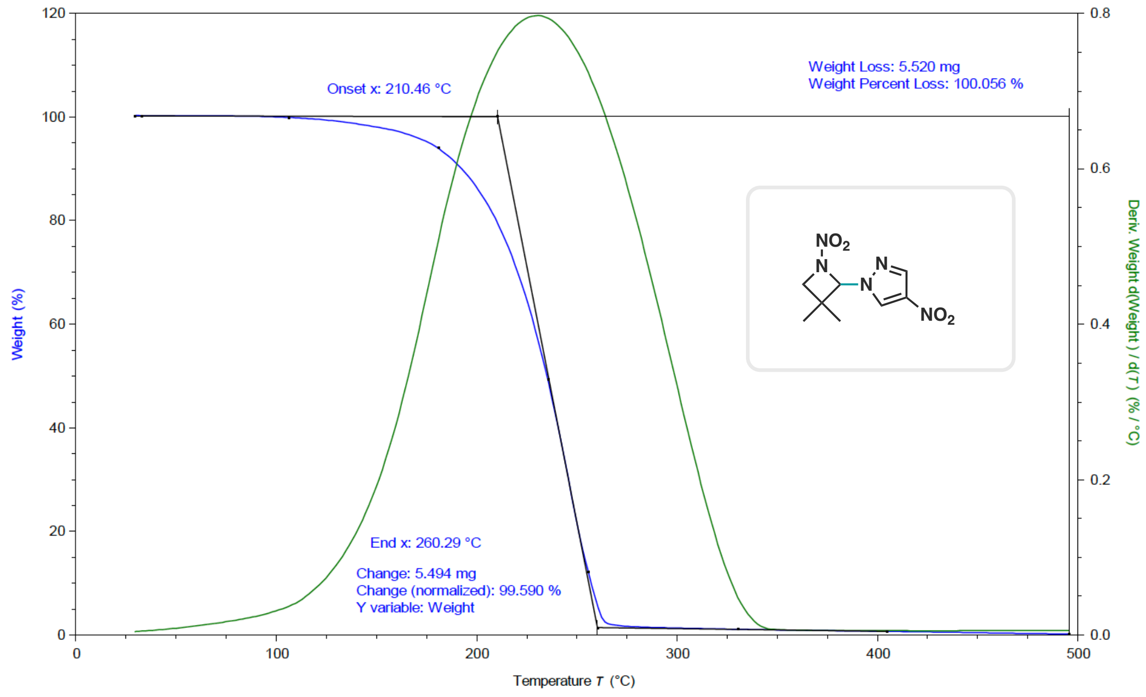

Figure S12. TGA Thermogram of **25**. The Onset Decomposition Temperature is 210 ºC.

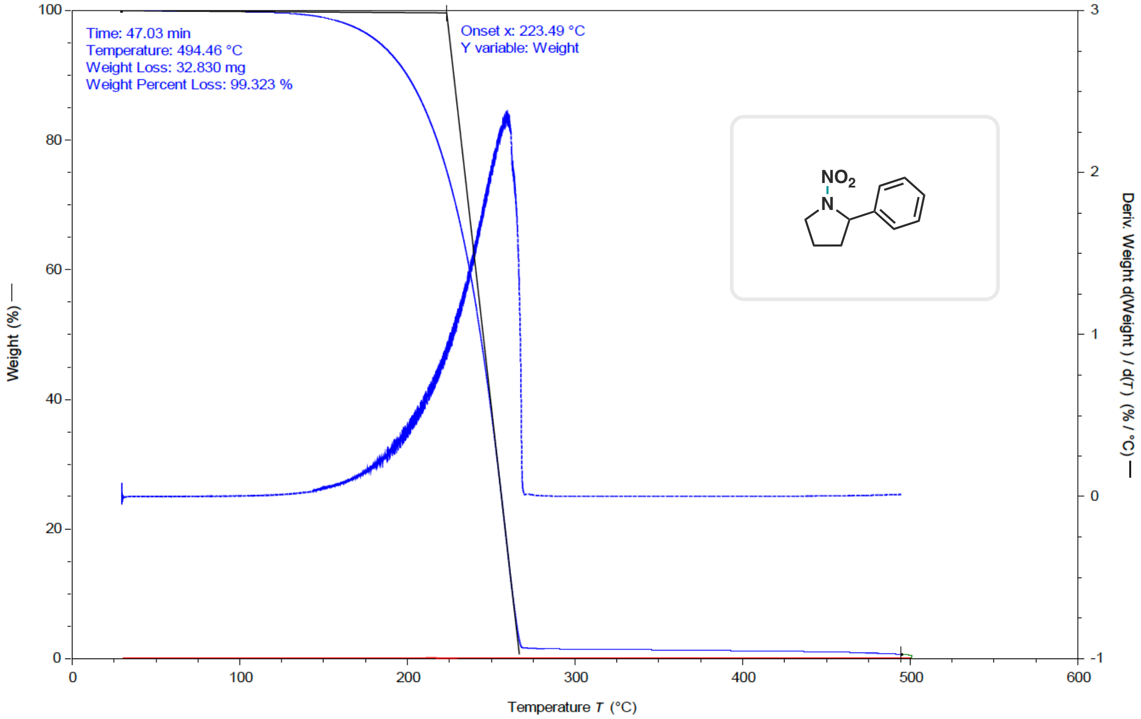

Figure S13. TGA Thermogram of **35**. The Onset Decomposition Temperature is 223 ºC.

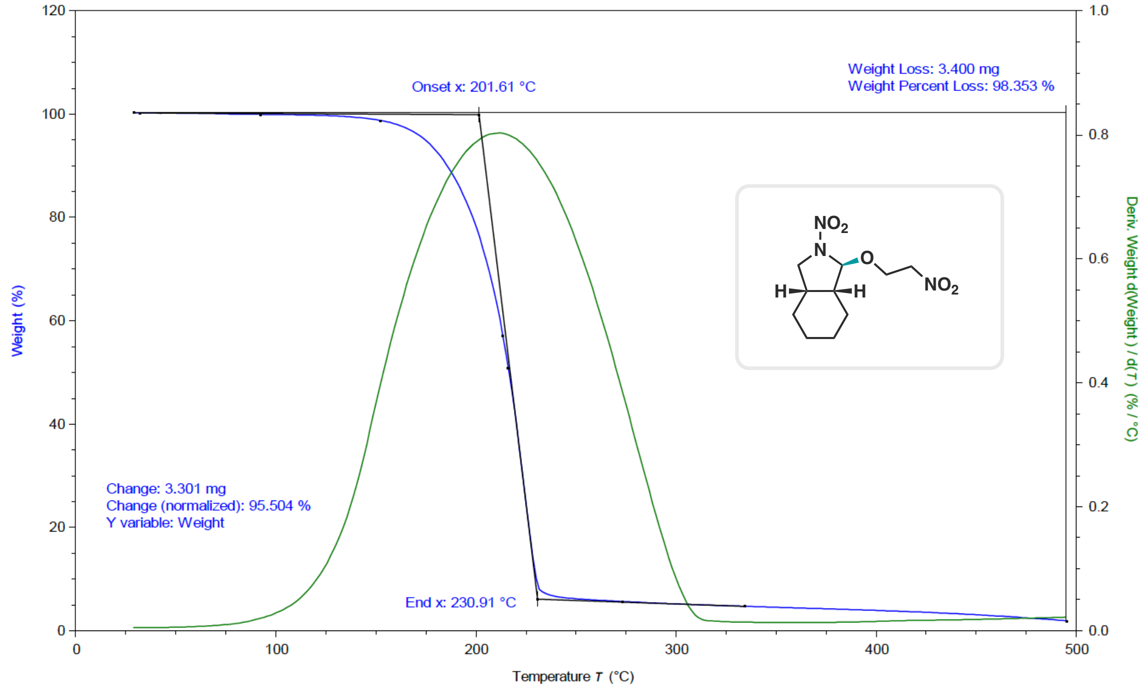

Figure S14. TGA Thermogram of **28**. The Onset Decomposition Temperature is 202 ºC.

11. X-ray Crystallography

Low-temperature X-ray diffraction data were collected on a Rigaku XtaLAB Synergy diffractometer coupled to a Rigaku Hypix detector with either Mo Kα radiation ($\lambda = 0.71073$ Å) or Cu Kα radiation ($\lambda = 1.54184$ Å), from a PhotonJet micro-focus X-ray source at 100 K. The diffraction images were processed and scaled using the CrysAlisPro software.[2] The structures were solved through intrinsic phasing using SHELXT[3] and refined against F^2 on all data by full-matrix least squares with SHELXL[4] following established refinement strategies.[5] All non-hydrogen atoms were refined anisotropically. All hydrogen atoms bound to carbon were included in the model at geometrically calculated positions and refined using a riding model. The isotropic displacement parameters of all hydrogen atoms were fixed to 1.2 times the U_{eq} value of the atoms they are linked to (1.5 times for methyl groups). Crystallographic data are available from the Cambridge Crystallographic Database Centre under accession codes CCDC 2406682, 2406683, 2406684, and 2406685.

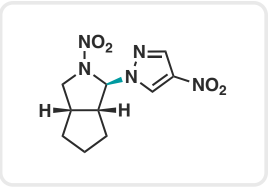

CCDC: 2406682

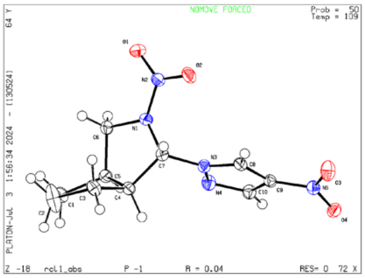

Table S12. Crystal Data and Structure Refinement for $C_{10}H_{13}N_5O_4$

Identification code	rcl1_abs	
Empirical formula	C10 H13 N5 O4	
Formula weight	267.25	
Temperature	109(1) K	
Wavelength	1.54184 Å	
Crystal system	Triclinic	
Space group	P -1	
Unit cell dimensions	a = 6.3033(2) Å	α= 93.549(2)°.
	b = 7.0294(2) Å	β= 99.490(2)°.
	c = 13.3001(2) Å	γ = 99.608(2)°.
Volume	570.66(3) Å^3	
Z	2	
Density (calculated)	1.555 Mg/m^3	
Absorption coefficient	1.047 mm^{-1}	
F(000)	280	
Crystal size	0.163 x 0.11 x 0.077 mm^3	
Theta range for data collection	3.383 to 79.763°.	
Index ranges	-8<=h<=7, -8<=k<=8, -16<=l<=16	
Reflections collected	12382	
Independent reflections	2449 [R(int) = 0.0282]	
Completeness to theta = 67.684°	100.0 %	
Absorption correction	Gaussian	
Max. and min. transmission	1.000 and 0.734	
Refinement method	Full-matrix least-squares on F^2	
Data / restraints / parameters	2449 / 0 / 172	
Goodness-of-fit on F^2	1.085	
Final R indices [I>2sigma(I)]	R1 = 0.0387, wR2 = 0.1003	
R indices (all data)	R1 = 0.0403, wR2 = 0.1018	
Extinction coefficient	n/a	
Largest diff. peak and hole	0.319 and -0.340 e.Å^{-3}	

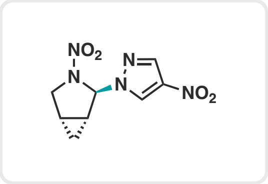

CCDC: 2406683

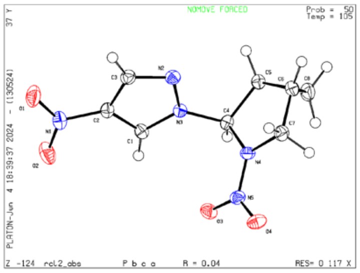

Table S13. Crystal Data and Structure Refinement for $C_8H_9N_5O_4$

Identification code	rcl2_abs	
Empirical formula	C8 H9 N5 O4	
Formula weight	239.20	
Temperature	105(10) K	
Wavelength	1.54184 Å	
Crystal system	Orthorhombic	
Space group	P b c a	
Unit cell dimensions	a = 14.7587(4) Å	α= 90°.
	b = 7.1235(2) Å	β= 90°.
	c = 19.3762(5) Å	γ = 90°.
Volume	2037.09(10) Å^3	
Z	8	
Density (calculated)	1.560 Mg/m^3	
Absorption coefficient	1.102 mm^{-1}	
F(000)	992	
Crystal size	0.15 x 0.117 x 0.043 mm^3	
Theta range for data collection	4.564 to 79.594°.	
Index ranges	-18<=h<=17, -6<=k<=9, -24<=l<=24	
Reflections collected	12823	
Independent reflections	2204 [R(int) = 0.0337]	
Completeness to theta = 67.684°	99.9 %	
Absorption correction	Gaussian	
Max. and min. transmission	1.000 and 0.654	
Refinement method	Full-matrix least-squares on F^2	
Data / restraints / parameters	2204 / 0 / 154	
Goodness-of-fit on F^2	1.069	
Final R indices [I>2sigma(I)]	R1 = 0.0386, wR2 = 0.0912	
R indices (all data)	R1 = 0.0440, wR2 = 0.0944	
Extinction coefficient	n/a	
Largest diff. peak and hole	0.264 and -0.247 e.Å^{-3}	

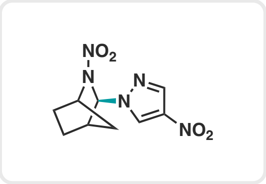

CCDC: 2406684

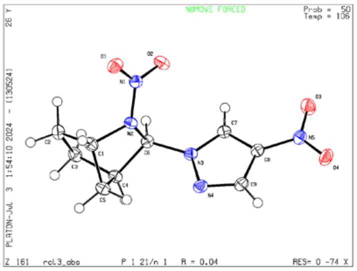

Table S14. Crystal Data and Structure Refinement for $C_9H_{11}N_5O_4$

Identification code	rcl3_abs	
Empirical formula	C9 H11 N5 O4	
Formula weight	253.23	
Temperature	106(5) K	
Wavelength	0.71073 Å	
Crystal system	Monoclinic	
Space group	P 1 21/n 1	
Unit cell dimensions	a = 9.5538(3) Å	α= 90°.
	b = 5.8732(2) Å	β= 96.385(3)°.
	c = 19.3531(6) Å	γ = 90°.
Volume	1079.19(6) $Å^3$	
Z	4	
Density (calculated)	1.559 Mg/m^3	
Absorption coefficient	0.125 mm^{-1}	
F(000)	528	
Crystal size	0.239 x 0.202 x 0.115 mm^3	
Theta range for data collection	3.627 to 27.097°.	
Index ranges	-12<=h<=12, -7<=k<=7, -24<=l<=24	
Reflections collected	12489	
Independent reflections	2394 [R(int) = 0.0432]	
Completeness to theta = 25.242°	99.8 %	
Absorption correction	Gaussian	
Max. and min. transmission	1.000 and 0.499	
Refinement method	Full-matrix least-squares on F^2	
Data / restraints / parameters	2394 / 0 / 163	
Goodness-of-fit on F^2	1.038	
Final R indices [I>2sigma(I)]	R1 = 0.0382, wR2 = 0.0945	
R indices (all data)	R1 = 0.0491, wR2 = 0.1014	
Extinction coefficient	n/a	
Largest diff. peak and hole	0.302 and -0.273 e.$Å^{-3}$	

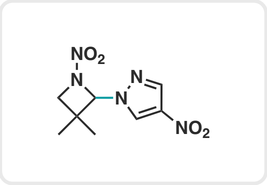

CCDC: 2406685

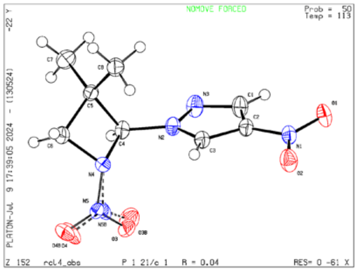

Table S15. Crystal data and structure refinement for $C_8H_{11}N_5O_4$

Identification code	rcl4_abs	
Empirical formula	C8 H11 N5 O4	
Formula weight	241.22	
Temperature	112.84(10) K	
Wavelength	1.54184 Å	
Crystal system	Monoclinic	
Space group	P 1 21/c 1	
Unit cell dimensions	a = 13.9842(2) Å	α= 90°.
	b = 6.00810(10) Å	β= 90.2170(10)°.
	c = 12.97190(10) Å	γ = 90°.
Volume	1089.87(3) Å^3	
Z	4	
Density (calculated)	1.470 Mg/m^3	
Absorption coefficient	1.030 mm^{-1}	
F(000)	504	
Crystal size	0.172 x 0.073 x 0.05 mm^3	
Theta range for data collection	3.160 to 79.871°.	
Index ranges	-17<=h<=17, -7<=k<=7, -15<=l<=16	
Reflections collected	23470	
Independent reflections	2373 [R(int) = 0.0465]	
Completeness to theta = 67.684°	100.0 %	
Absorption correction	Gaussian	
Max. and min. transmission	1.000 and 0.763	
Refinement method	Full-matrix least-squares on F^2	
Data / restraints / parameters	2373 / 93 / 184	
Goodness-of-fit on F^2	1.039	
Final R indices [I>2sigma(I)]	R1 = 0.0388, wR2 = 0.0985	
R indices (all data)	R1 = 0.0409, wR2 = 0.1001	
Extinction coefficient	n/a	
Largest diff. peak and hole	0.304 and -0.226 e.Å^{-3}	

12. DFT Calculations

Initial molecular structures of all compounds were generated using a distance geometry algorithm.[6] For each species, up to 25 molecular conformers were generated. The generated structures were optimized using the hybrid M06-2X functional[7] combined with the D3 dispersion correction,[8] and the def2-TZVPP triple-zeta set[9] of Gaussian-type atomic orbitals. Vibrational frequencies (unscaled), moments of inertia, and the associated thermodynamic functions were calculated within the ideal gas, rigid rotor, and harmonic oscillator partition functions at the same level of DFT. The resulting structures with the lowest absolute free energies were used in the subsequent simulations of molecular volumes and as the initial structures in the composite ab initio simulations.

Gas-phase van der Waals volumes (V_{gas}^{vdW})[10] were computed based on the M06-2X/def2-TZVPP structures using a modified marching-cube algorithm as implemented in the pyMoVe code.[11] The computed volumes were combined with molar masses (M_{gas}) and an empirical scaling constant (α) to provide a simple empirical estimate for the crystal densities: $\rho = M_{gas}/V_{gas}^{vdW}$. Previous studies have shown that V_{gas}^{vdW} values correlate with known crystal densities of nitrated compounds, with α ranging from 0.7 to 1.1 depending on the precise definition.[12] Here, α was set to 0.8, based on the average ratio between M_{gas}/V_{gas}^{vdW} and ρ values for 1,3-DNAZ, TNAZ, 1,4-DNP, and RDX. By design, ρ does not account for the differences in the intermolecular interactions in different crystals and/or polymorphs, and therefore is a qualitative metric and should be interpreted as such.

Gas-phase heats of formation ($\Delta_f H_{gas}^{\circ}$) were estimated based on the corresponding enthalpies of atomization ($\Delta_a H_{gas}^{\circ}$) computed with the G4(MP2) composite method.[13] G4(MP2) combines DFT structure optimizations and RRHO thermal corrections with electronic energies computed using coupled cluster singles and doubles with perturbative treatment of triple excitations (CCSD(T)) as well as complete-basis-set extrapolations at the Hartree-Fock (HF) and second-order Moller-Plesset perturbation theory (MP2) levels, spin-orbit corrections, and one-parameter additive empirical corrections. Previous studies showed that G4(MP2) delivers $\Delta_f H_{gas}^{\circ}$ values within about 2 kcal/mol of known experimental values for smaller molecules,[14] although the errors tend to be larger for larger molecules with many nitrogen atoms.[15]

Gas-phase bond dissociation energies (BDEs) for N–NO_2 bonds were estimated using the CBS-QB3 composite method. Similar to G4(MP2), CBS-QB3 method combines structures and thermochemistry computed with DFT and electronic energies computed using a combination of electron-correlation methods. CBS-QB3 is computationally more efficient than G4(MP2), which facilitates simulations of radicals, wherein the need for unrestricted orbitals doubles the number of

orbitals. Previous studies showed that CBS-QB3 delivers $\Delta_f H^{\circ}_{gas}$ values for CHO radicals within about 4 kcal/mol of known experimental values;[16] however, its accuracy for radicals with multiple nitrogen atoms has not been evaluated.

13. References

[1] Q. Yang, Y. Li, J. D. Yang, Y. D. Liu, L. Zhang, S. Z. Luo, J. P. Cheng, *Angew. Chem., Int. Ed.* **2020**, *59*, 19282–19291.

[2] R. O. D. CrysAlisPro, *Rigaku Corporation, The Woodlands, TX* **2015**.

[3] G. M. Sheldrick, *Acta Crystallogr. A* **2015**, *71*, 3–8.

[4] G. M. Sheldrick, *Acta Crystallogr. A* **2008**, *64*, 112–122.

[5] P. Müller, *Crystallogr. Rev.* **2009**, *15*, 57–83.

[6] S. Riniker, G. A. Landrum, *J. Chem. Inf. Model.* **2015**, *55*, 2562–2574.

[7] Y. Zhao, D. G. Truhlar, *Theor. Chem. Acc.* **2008**, *120*, 215–241.

[8] S. Grimme, J. Antony, S. Ehrlich, H. Krieg, *J. Chem. Phys.* **2010**, *132*.

[9] F. Weigend, R. Ahlrichs, *Phys. Chem. Chem. Phys.* **2005**, *7*, 3297–3305.

[10] J. C. McGowan, A. Mellors, *Molecular Volumes in Chemistry and Biology: Applications Including Partitioning and Toxicity.* **1986**.

[11] I. Bier, N. Marom, *J. Phys. Chem. A* **2020**, *124*, 10330–10345.

[12] L. Qiu, H. M. Xiao, X. D. Gong, X. H. Ju, W. H. Zhu, *J. Hazard. Mater.* **2007**, *141*, 280–288.

[13] L. A. Curtiss, P. C. Redfern, K. Raghavachari, *J. Chem. Phys.* **2007**, *127*.

[14] N. K. Dandu, R. S. Assary, P. C. Redfern, L. Ward, I. Foster, L. A. Curtiss, *J. Phys. Chem. A* **2022**, *126*, 4528–4536.

[15] N. Dandu, L. Ward, R. S. Assary, P. C. Redfern, B. Narayanan, I. T. Foster, L. A. Curtiss, *J. Phys. Chem. A* **2020**, *124*, 5804–5811.

[16] K. P. Somers, J. M. Simmie, *J. Phys. Chem. A* **2015**, *119*, 8922–8933.

14. NMR Spectra

^{1}H NMR of **1a** in Chloroform-*d*

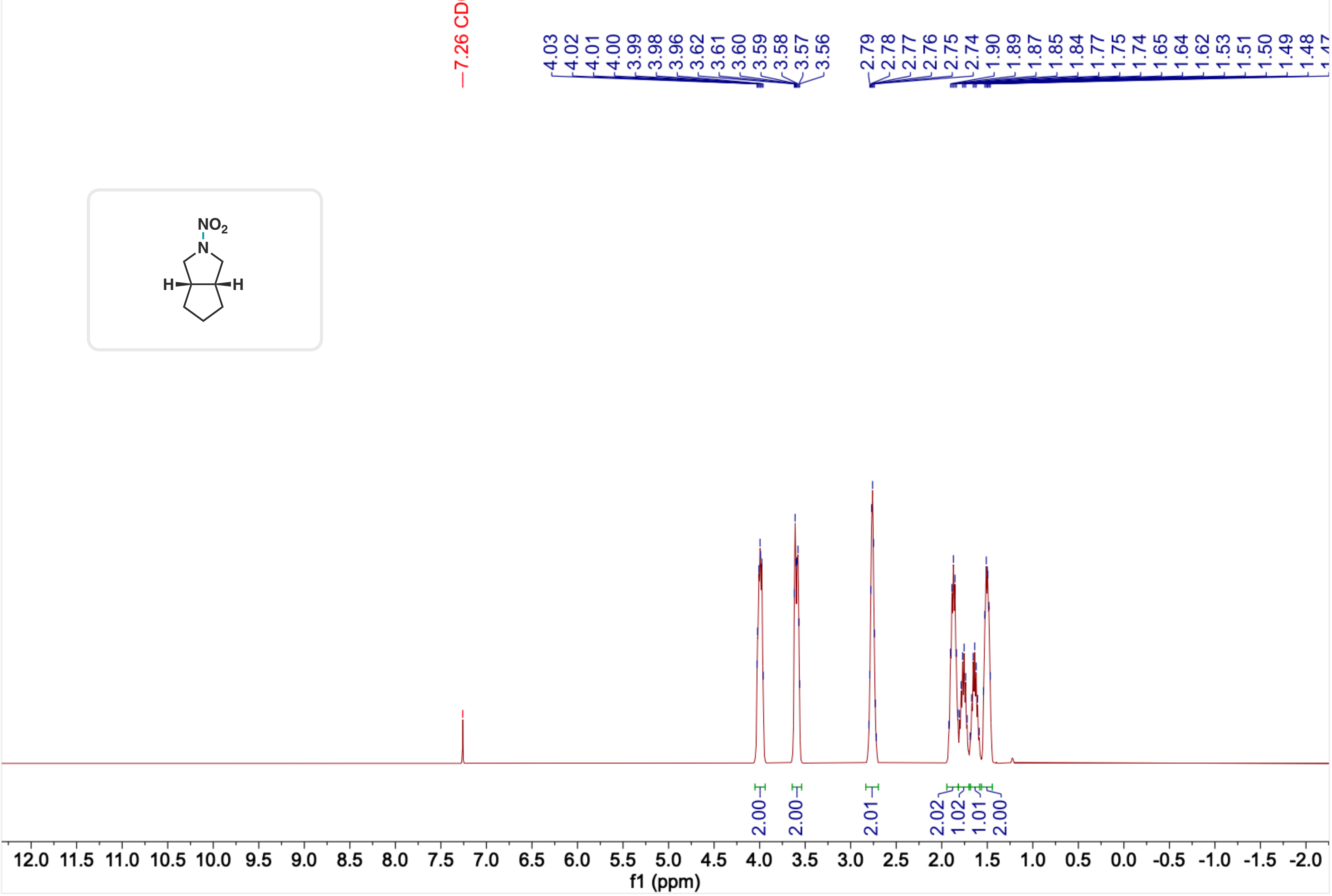

^{13}C NMR of **1a** in Chloroform-*d*

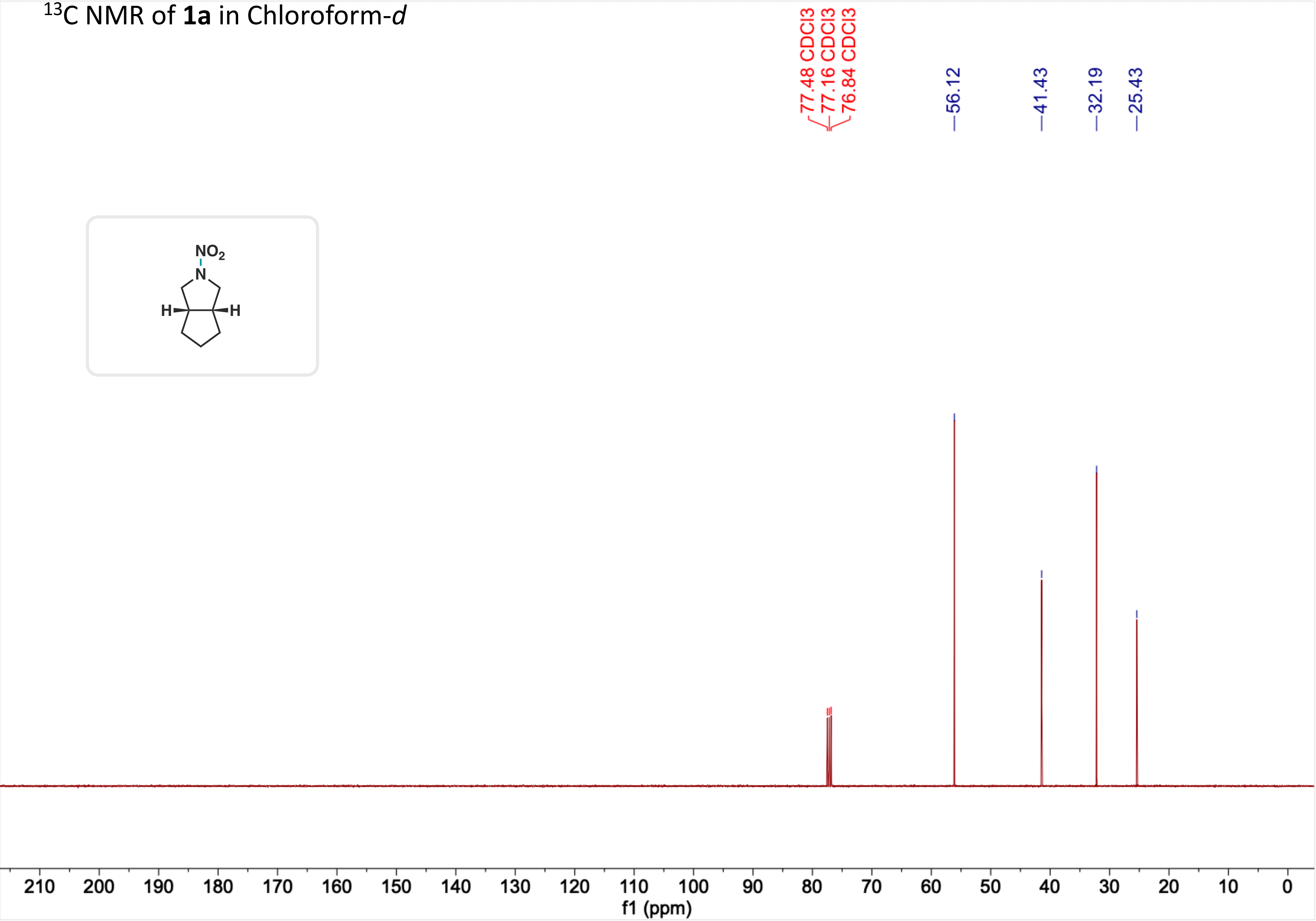

^{1}H NMR of **1b** in Chloroform-*d*

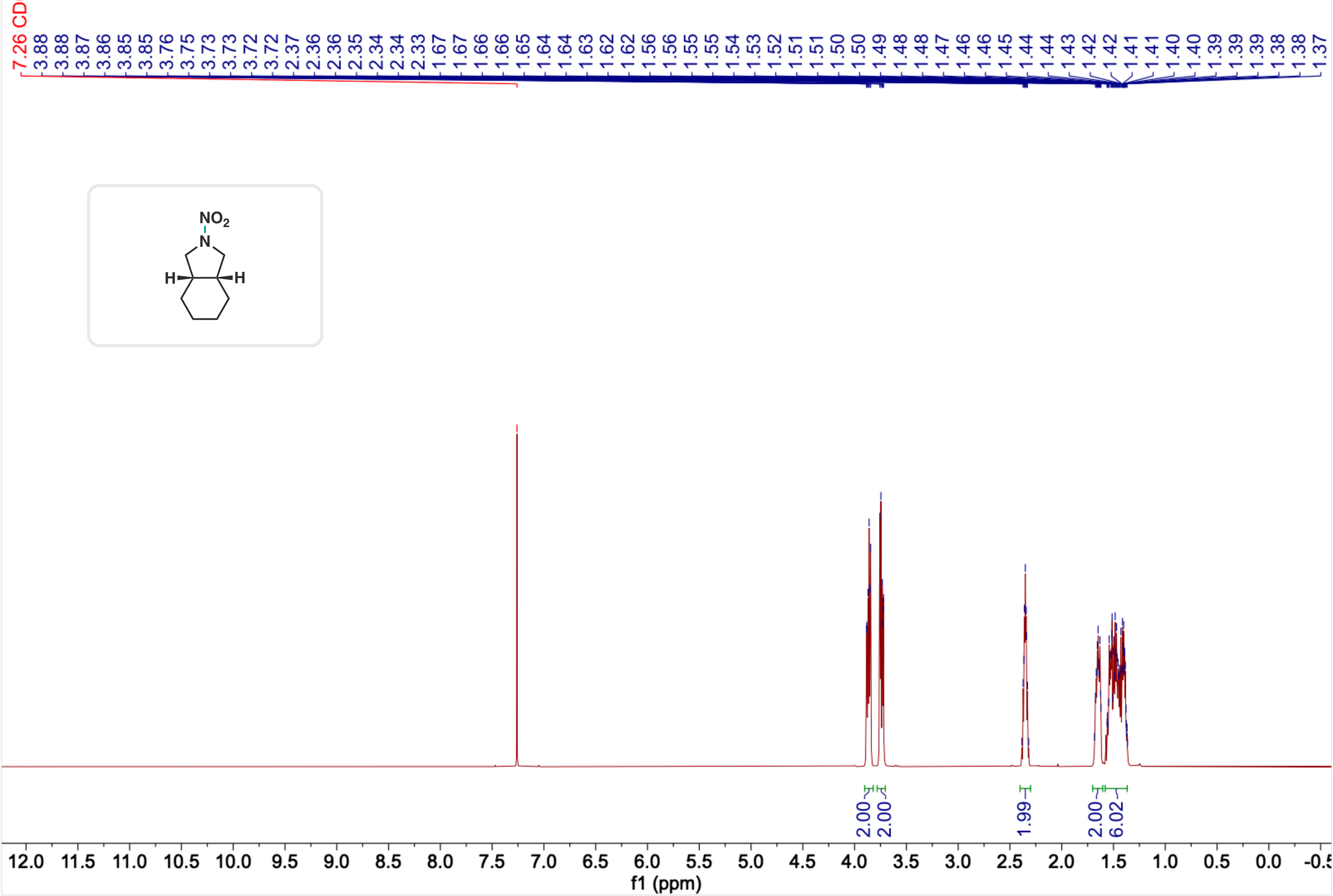

^{13}C NMR of **1b** in Chloroform-*d*

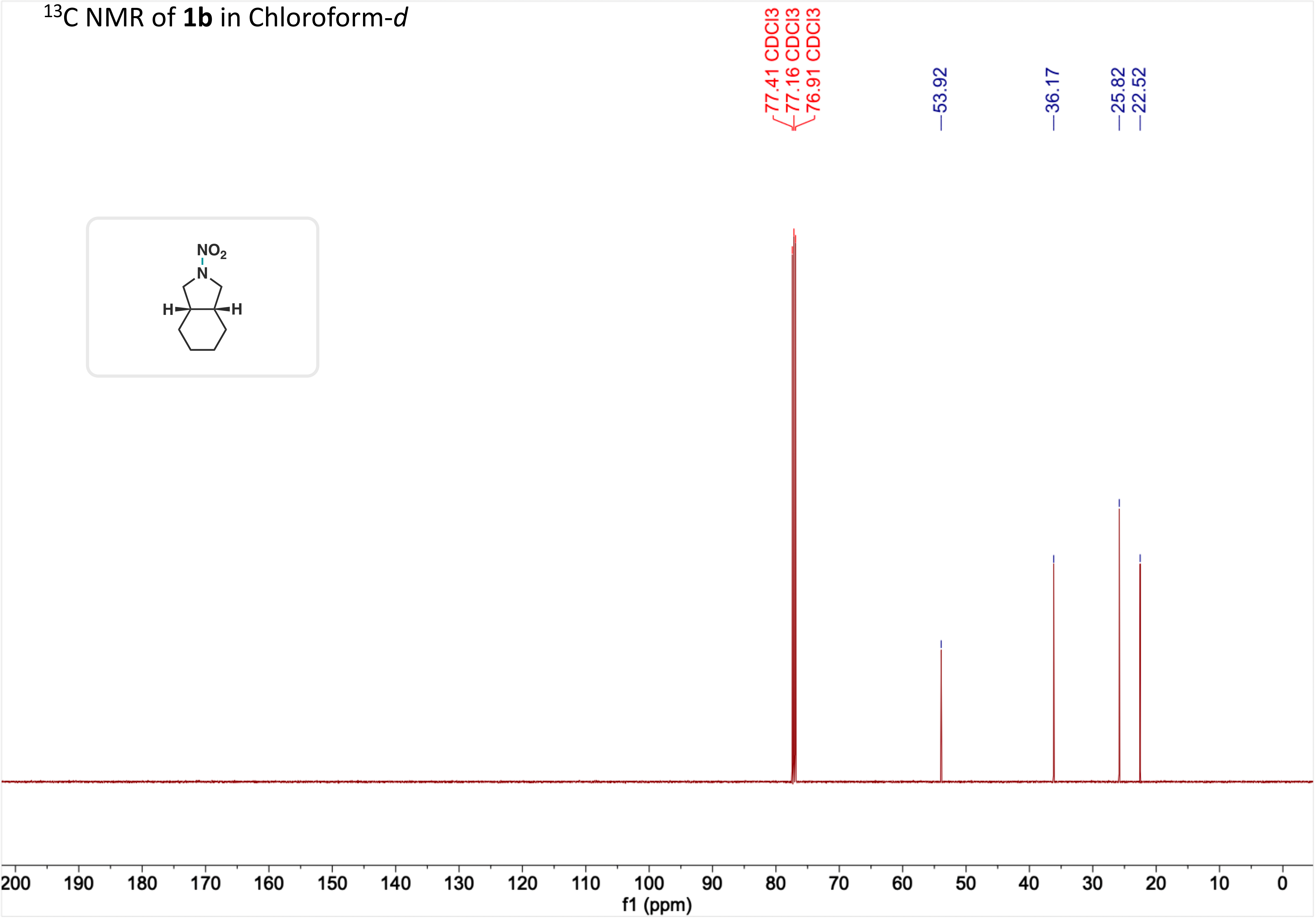

^{1}H NMR of **1c** in Chloroform-*d*

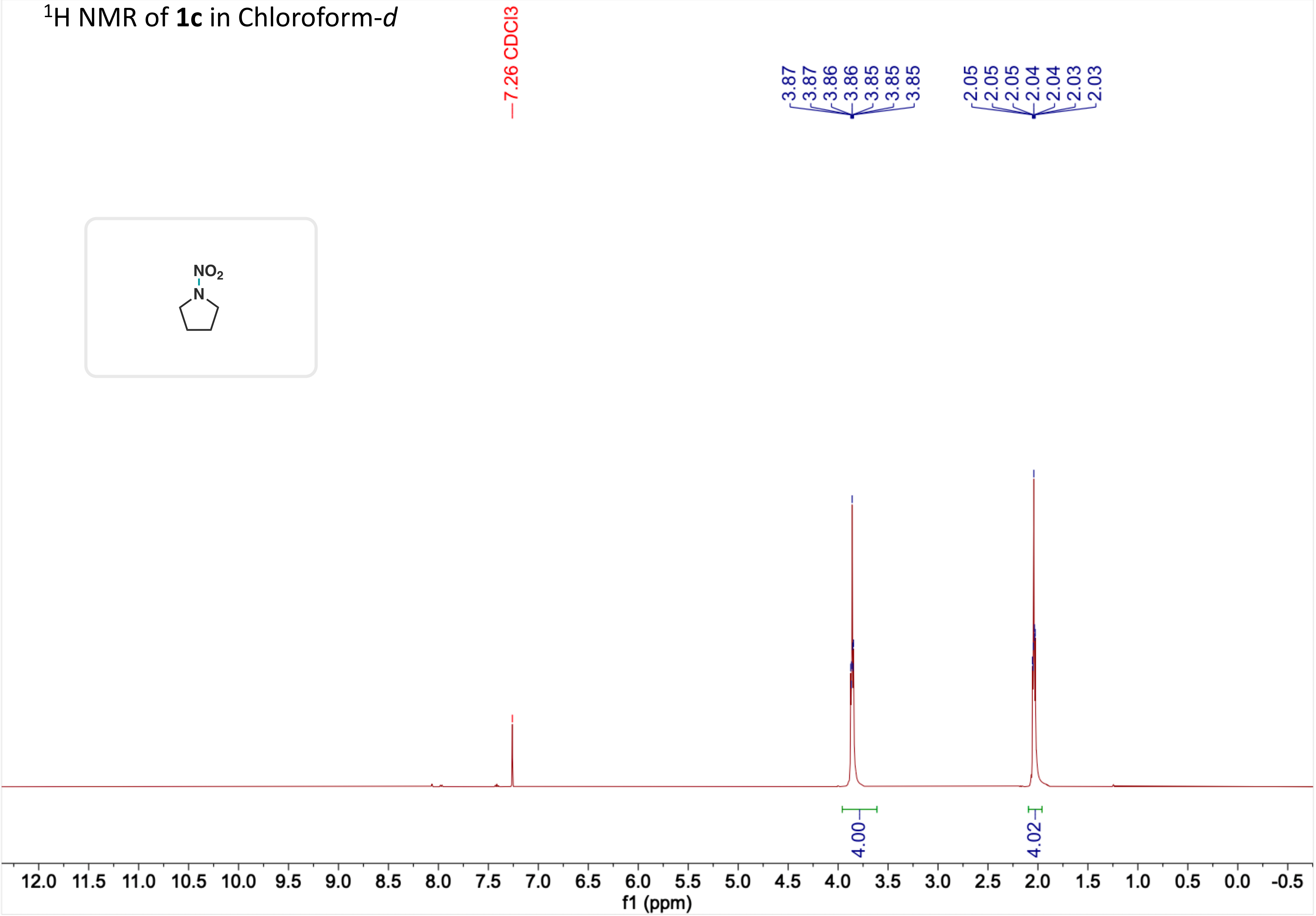

^{13}C NMR of **1c** in Chloroform-*d*

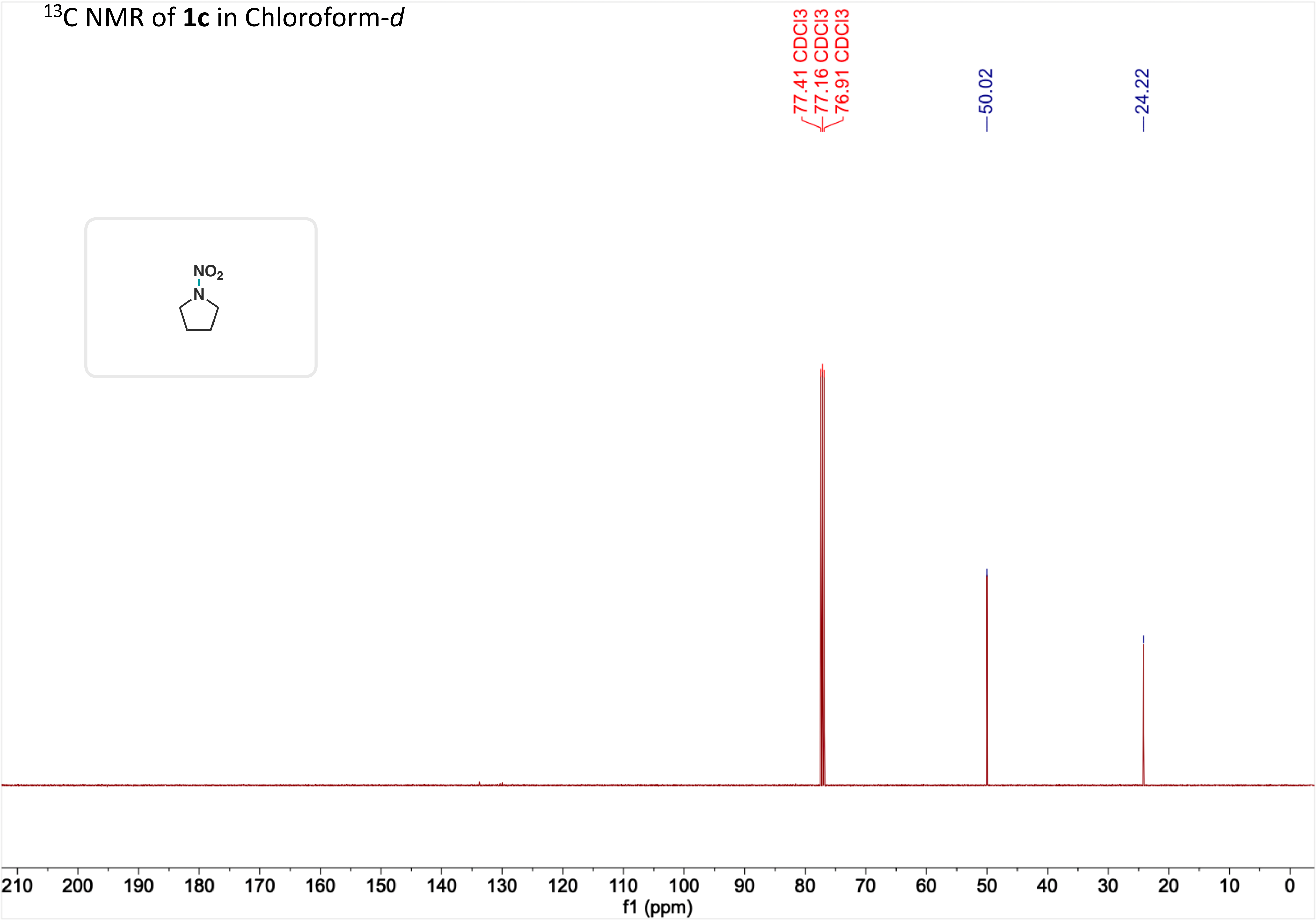

^{1}H NMR of **1d** in Chloroform-*d*

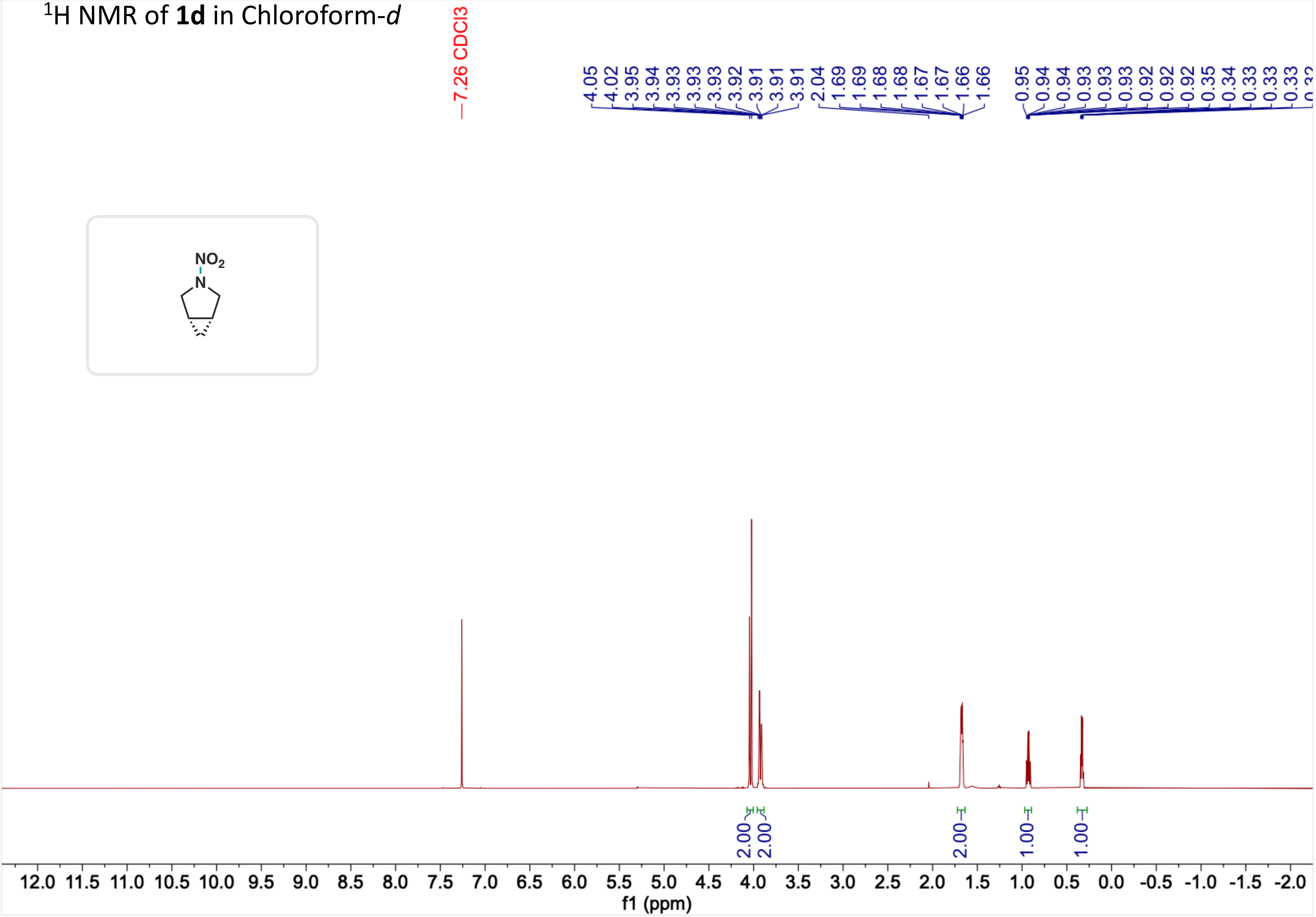

^{13}C NMR of **1d** in Chloroform-*d*

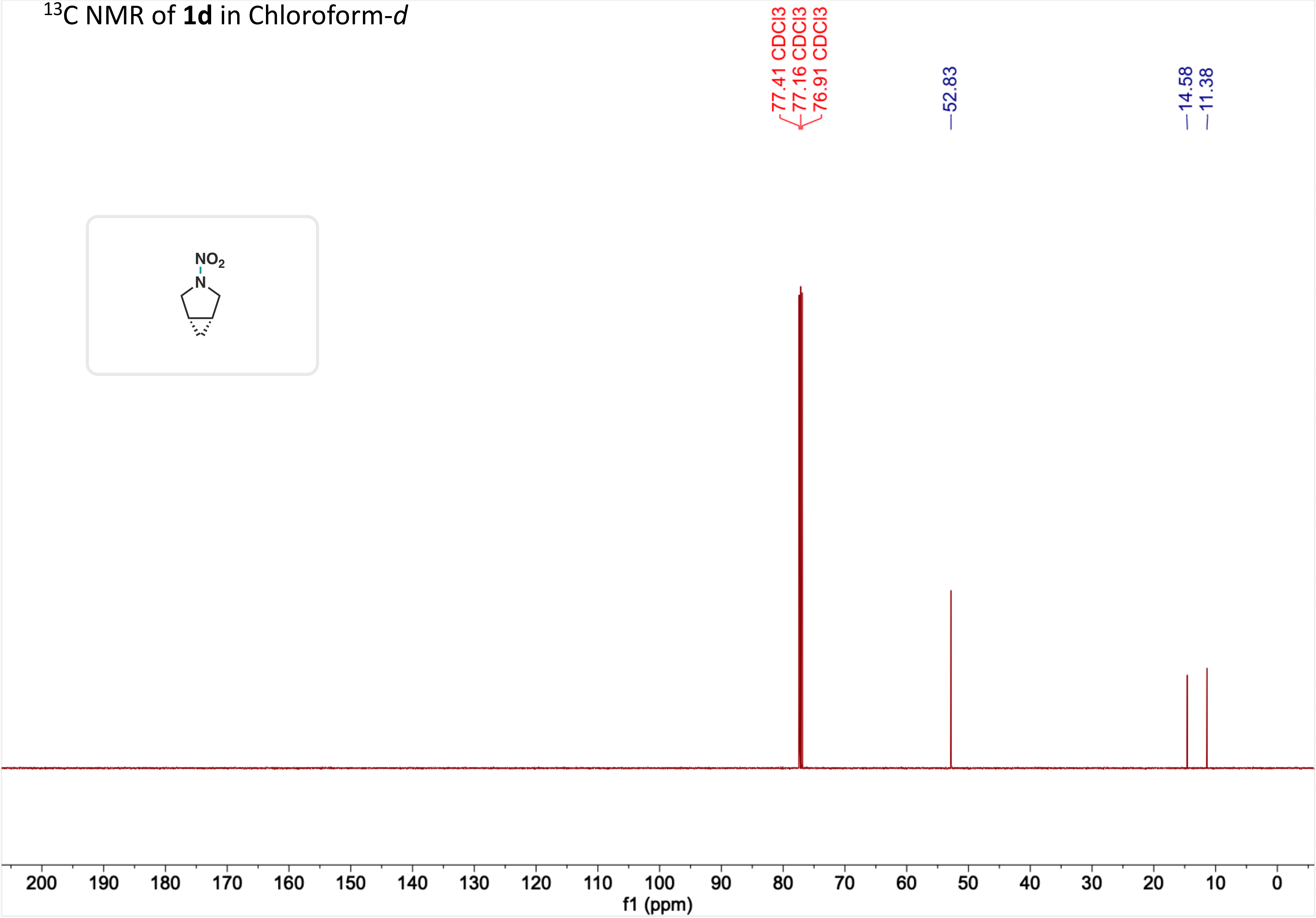

^1H NMR of **1e** in Chloroform-*d*

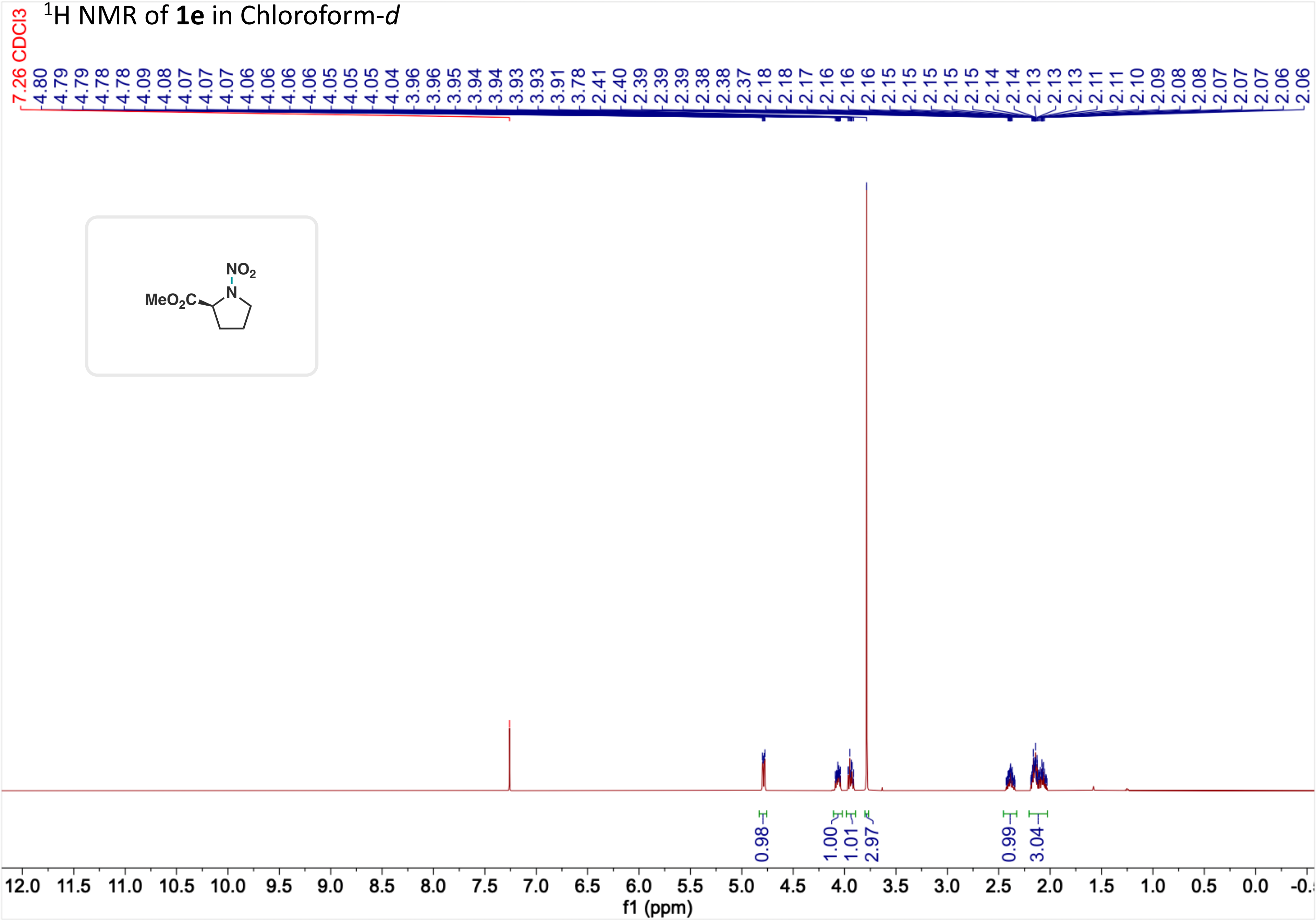

^{13}C NMR of **1e** in Chloroform-*d*

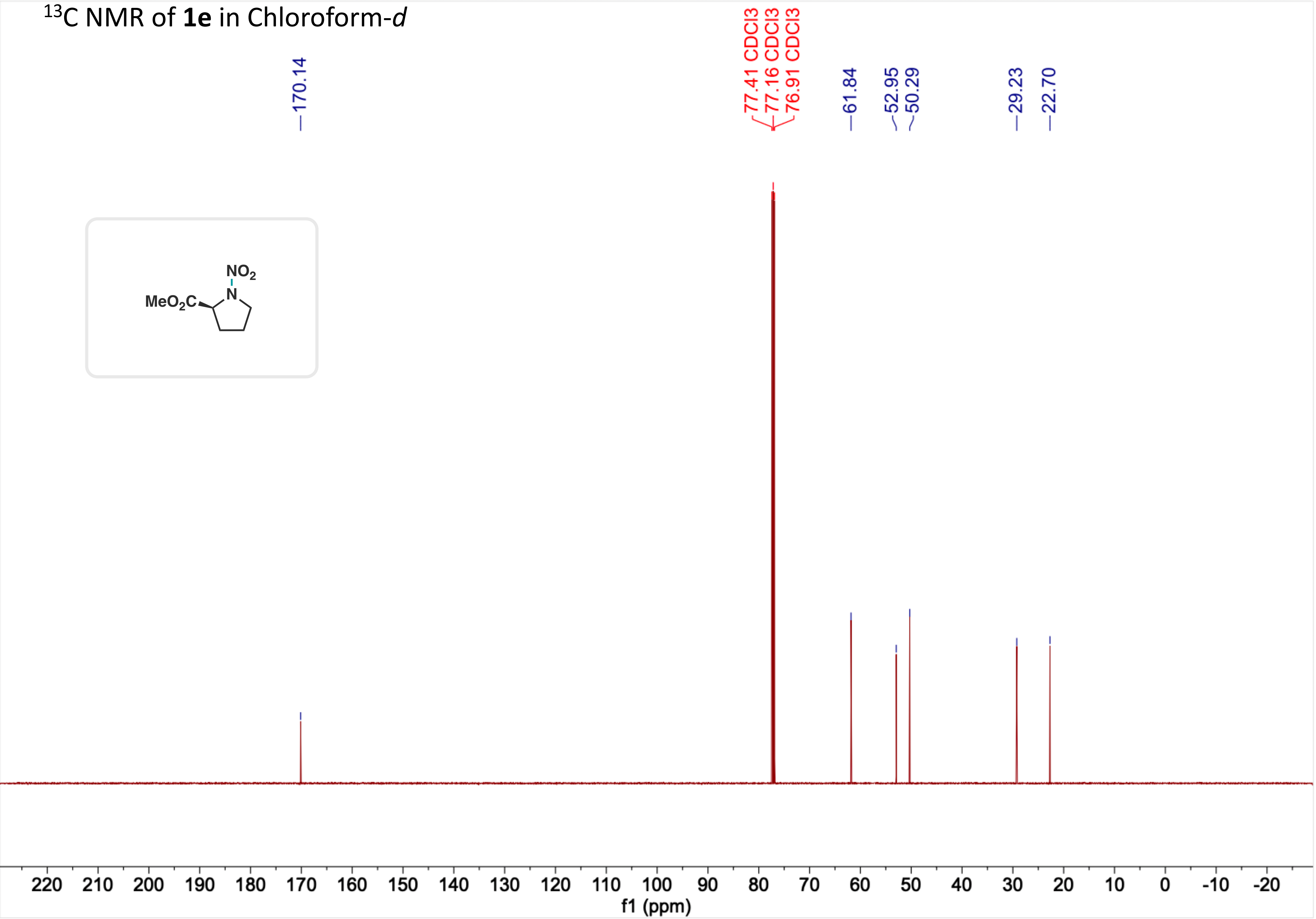

^{1}H NMR of **1f** in Chloroform-*d*

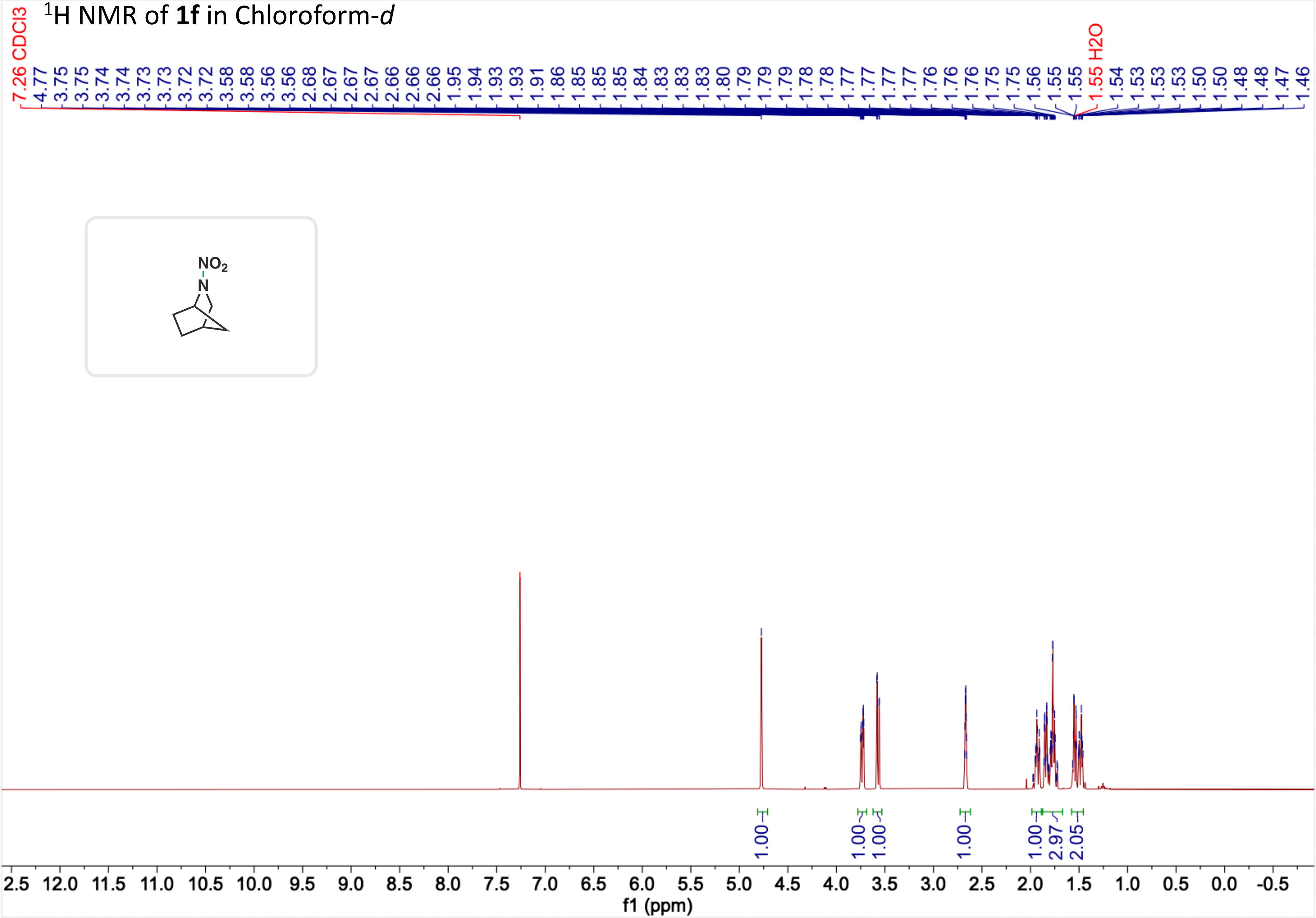

¹³C NMR of **1f** in Chloroform-*d*

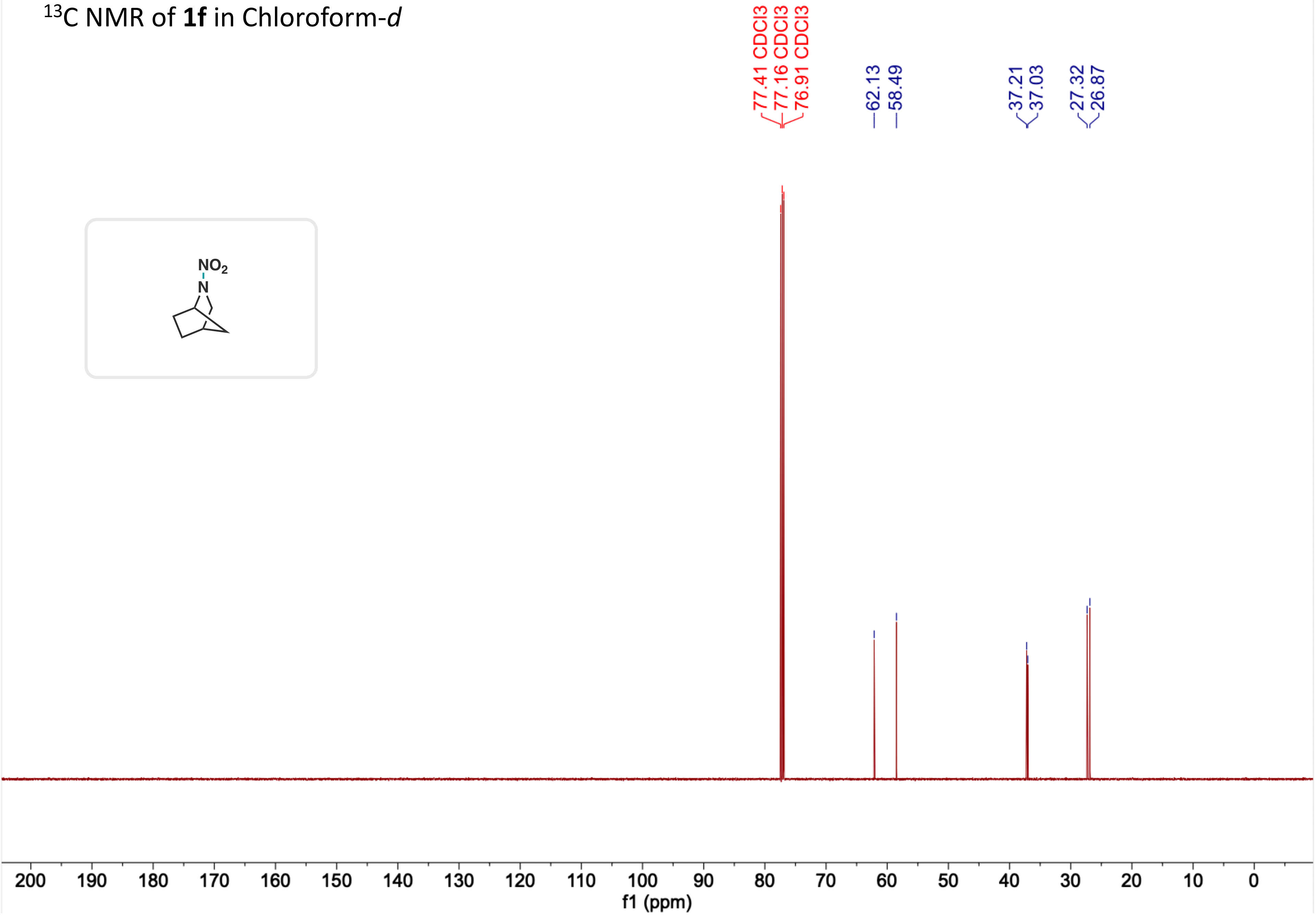

^{1}H NMR of **1g** in Acetone-d_6

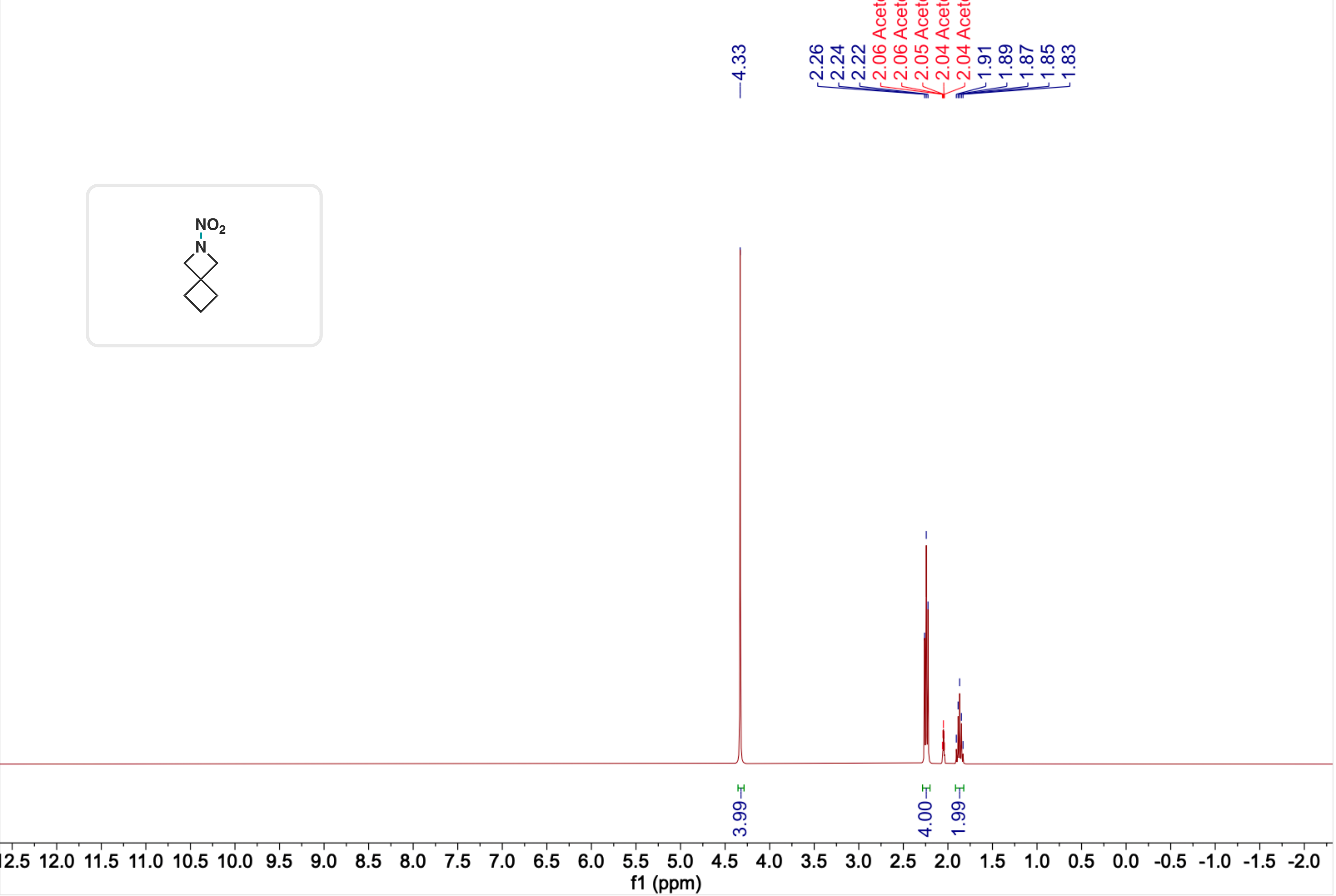

^{13}C NMR of **1g** in Acetone-d_6

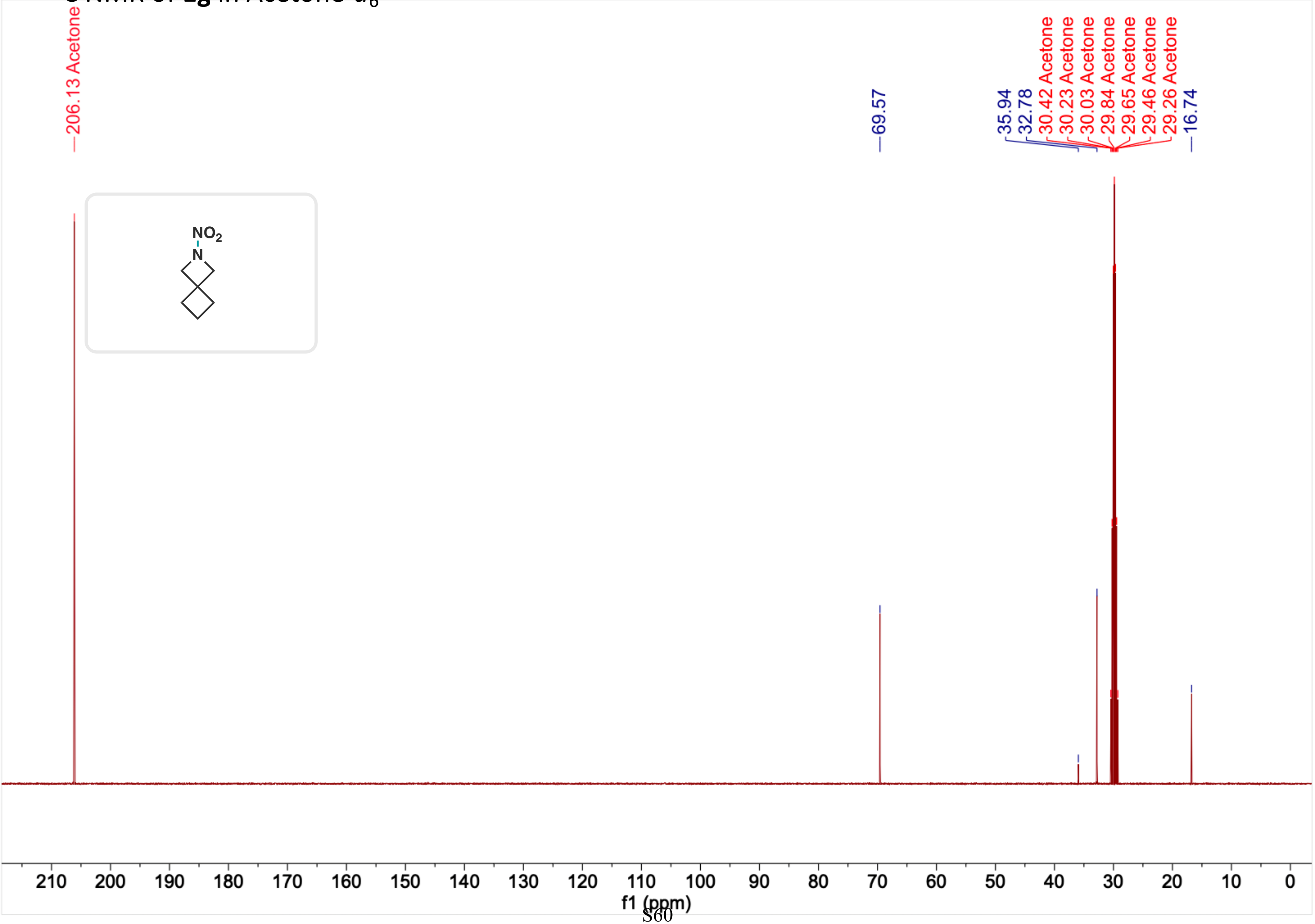

^{1}H NMR of **1h** in Chloroform-*d*

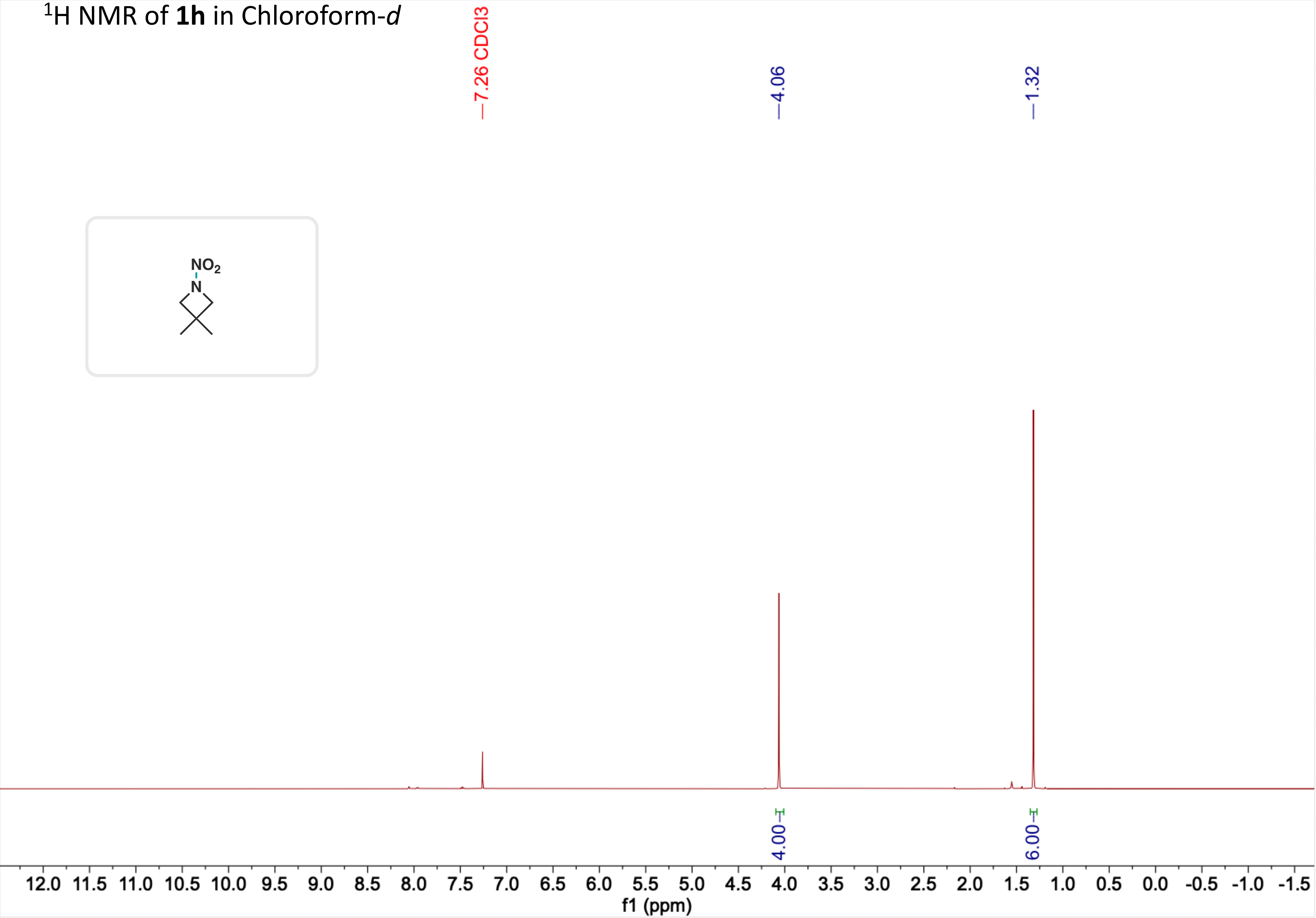

^{13}C NMR of **1h** in Chloroform-*d*

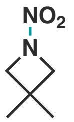

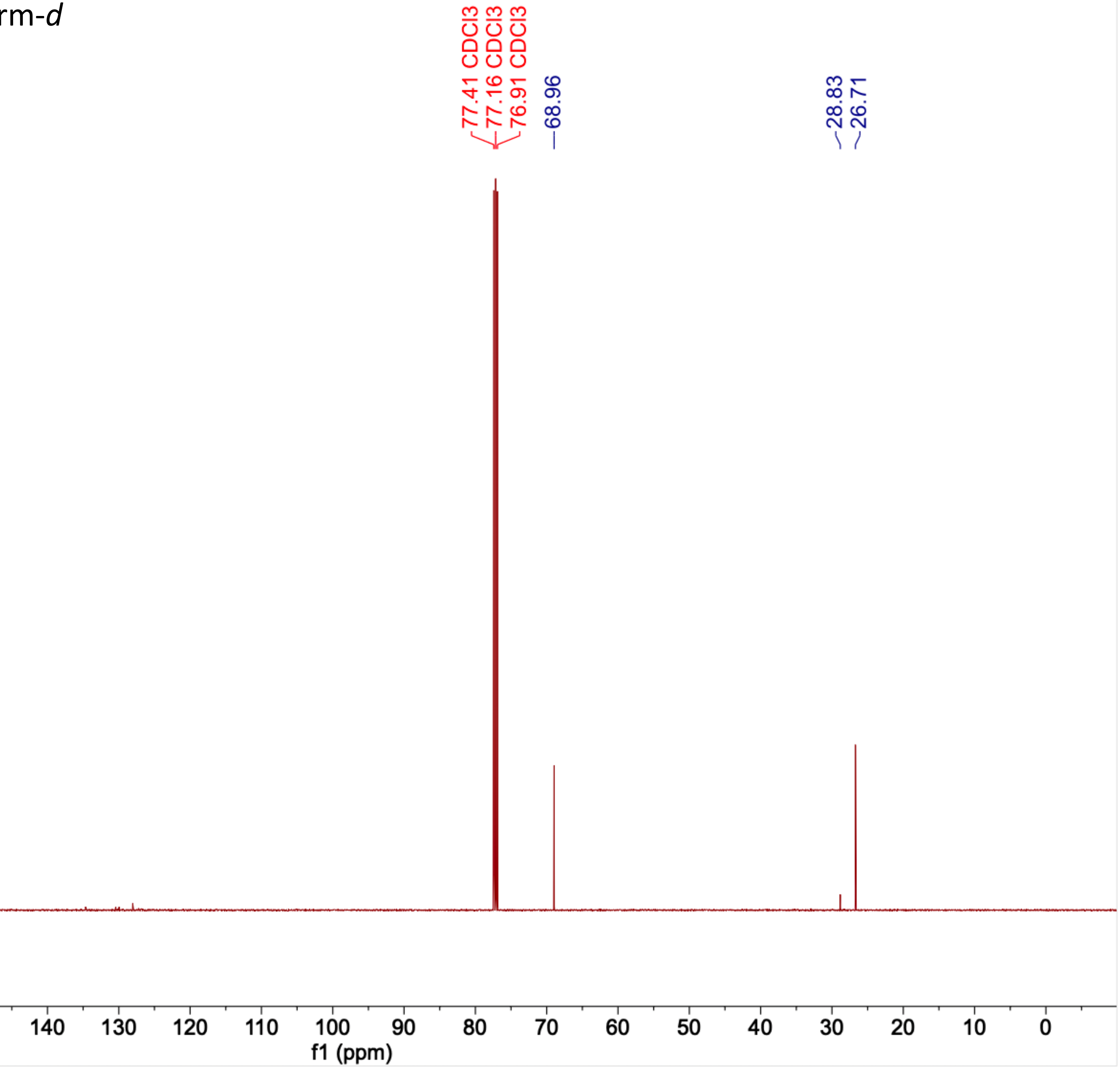

^{1}H NMR of **1i** in Chloroform-*d*

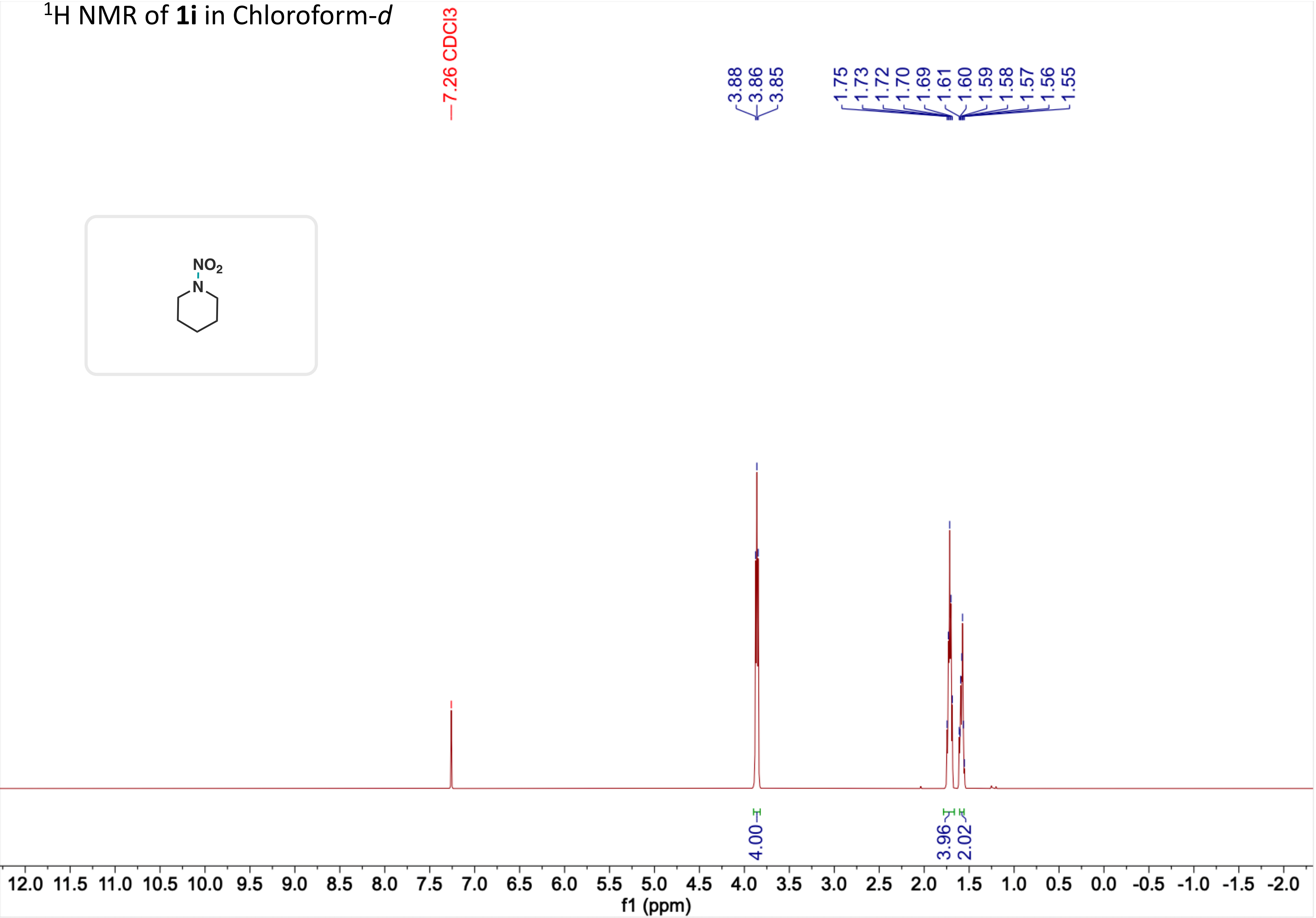

^{13}C NMR of **1i** in Chloroform-*d*

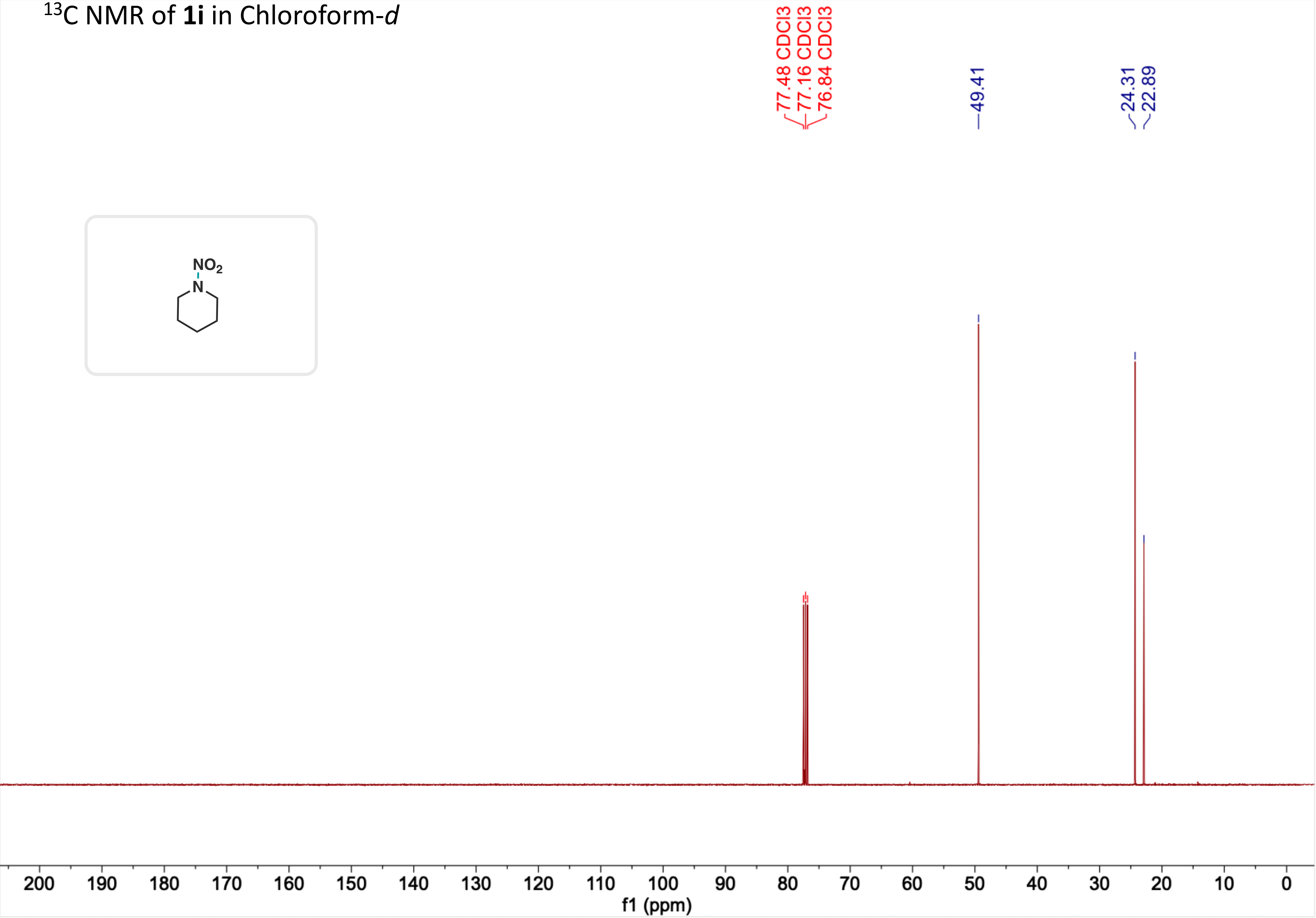

^{1}H NMR of **35** in Chloroform-*d*

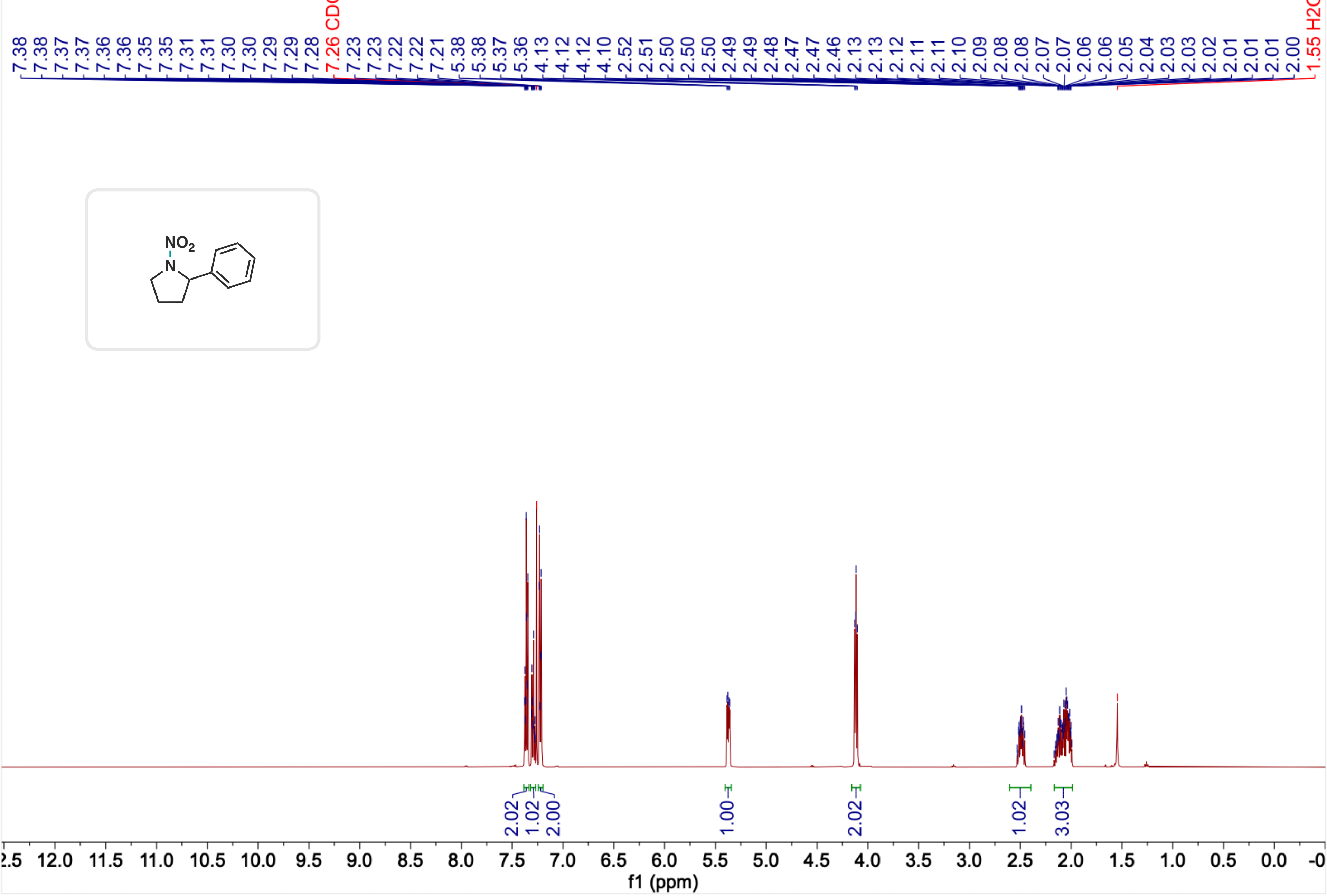

^{13}C NMR of **35** in Chloroform-*d*

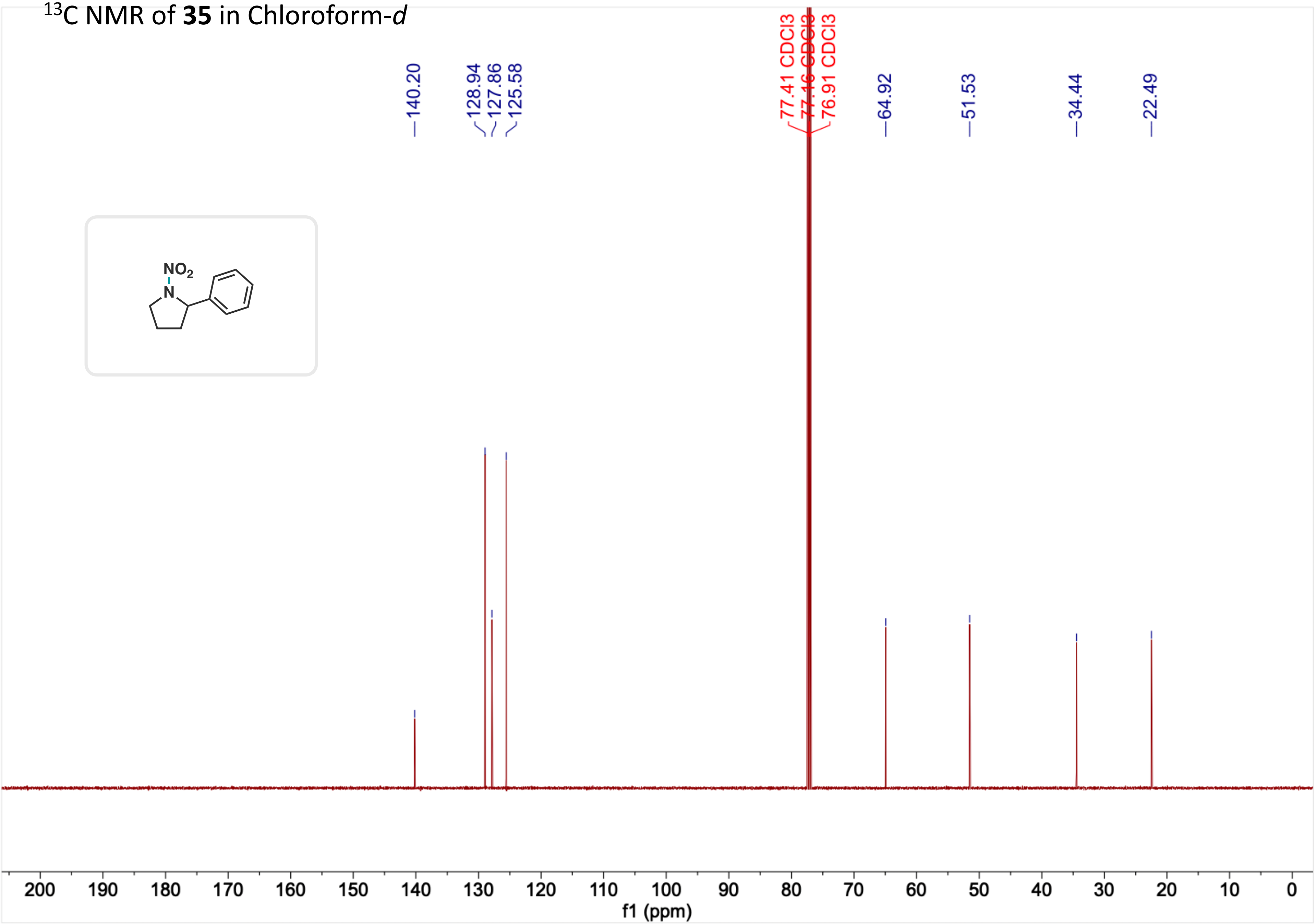

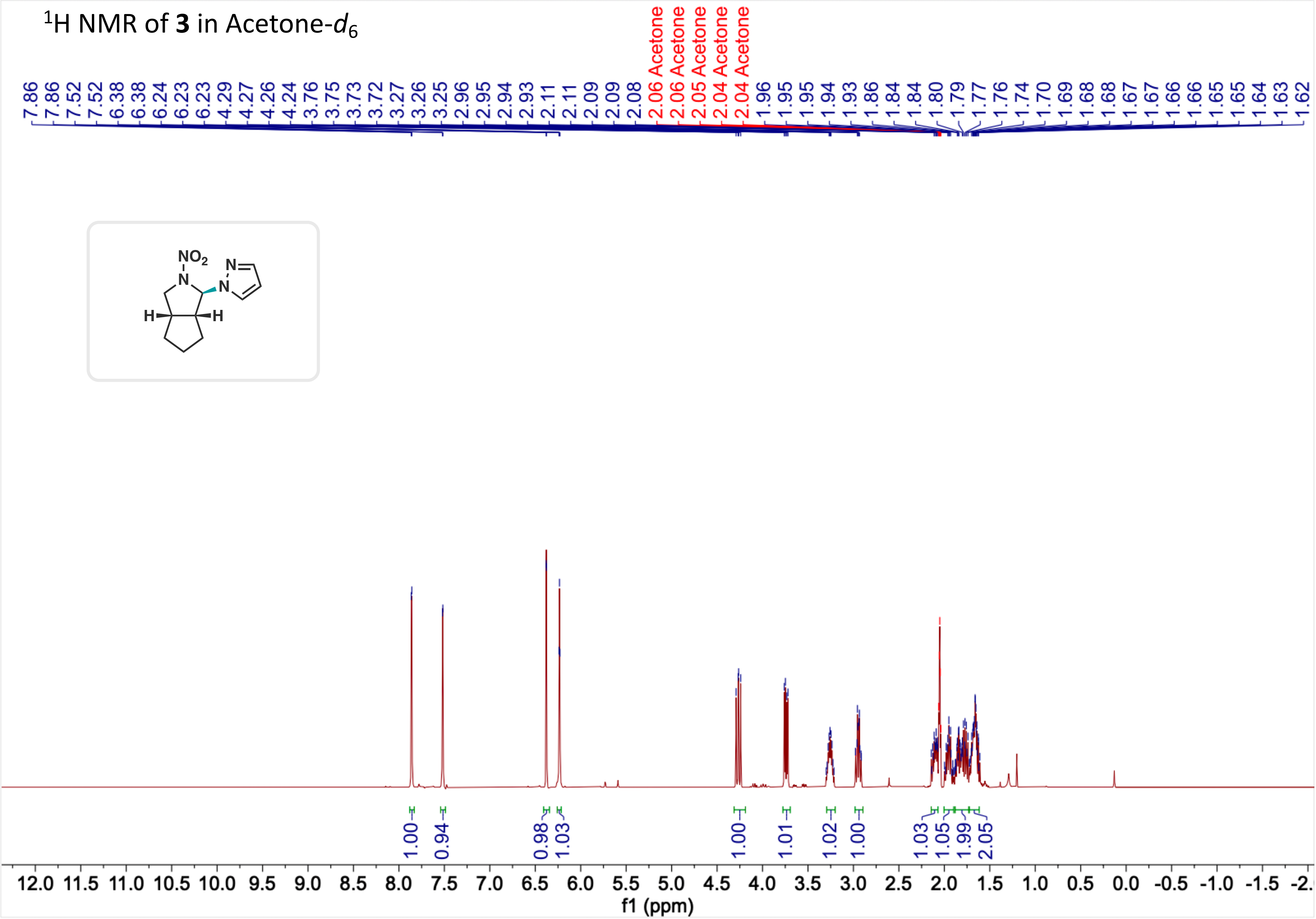

^{1}H NMR of **3** in Acetone-d_6
7.86 7.86 7.52 7.52 6.38 6.38 6.24 6.23 6.23 4.29 4.27 4.26 4.24 3.76 3.75 3.73 3.72 3.27 3.26 3.25 2.96 2.95 2.94 2.93 2.11 2.11 2.09 2.09 2.08
2.06 Acetone 2.06 Acetone 2.05 Acetone 2.04 Acetone 2.04 Acetone
1.96 1.95 1.95 1.94 1.93 1.86 1.84 1.84 1.80 1.79 1.77 1.76 1.74 1.70 1.69 1.68 1.68 1.67 1.67 1.66 1.66 1.65 1.65 1.64 1.63 1.62
NO2
N
N
N
H
H
1.00 0.94 0.98 1.03 1.00 1.01 1.02 1.00 1.03 1.05 1.99 2.05
12.0 11.5 11.0 10.5 10.0 9.5 9.0 8.5 8.0 7.5 7.0 6.5 6.0 5.5 5.0 4.5 4.0 3.5 3.0 2.5 2.0 1.5 1.0 0.5 0.0 -0.5 -1.0 -1.5 -2.
f1 (ppm)

^{13}C NMR of **3** in Acetone-d_6

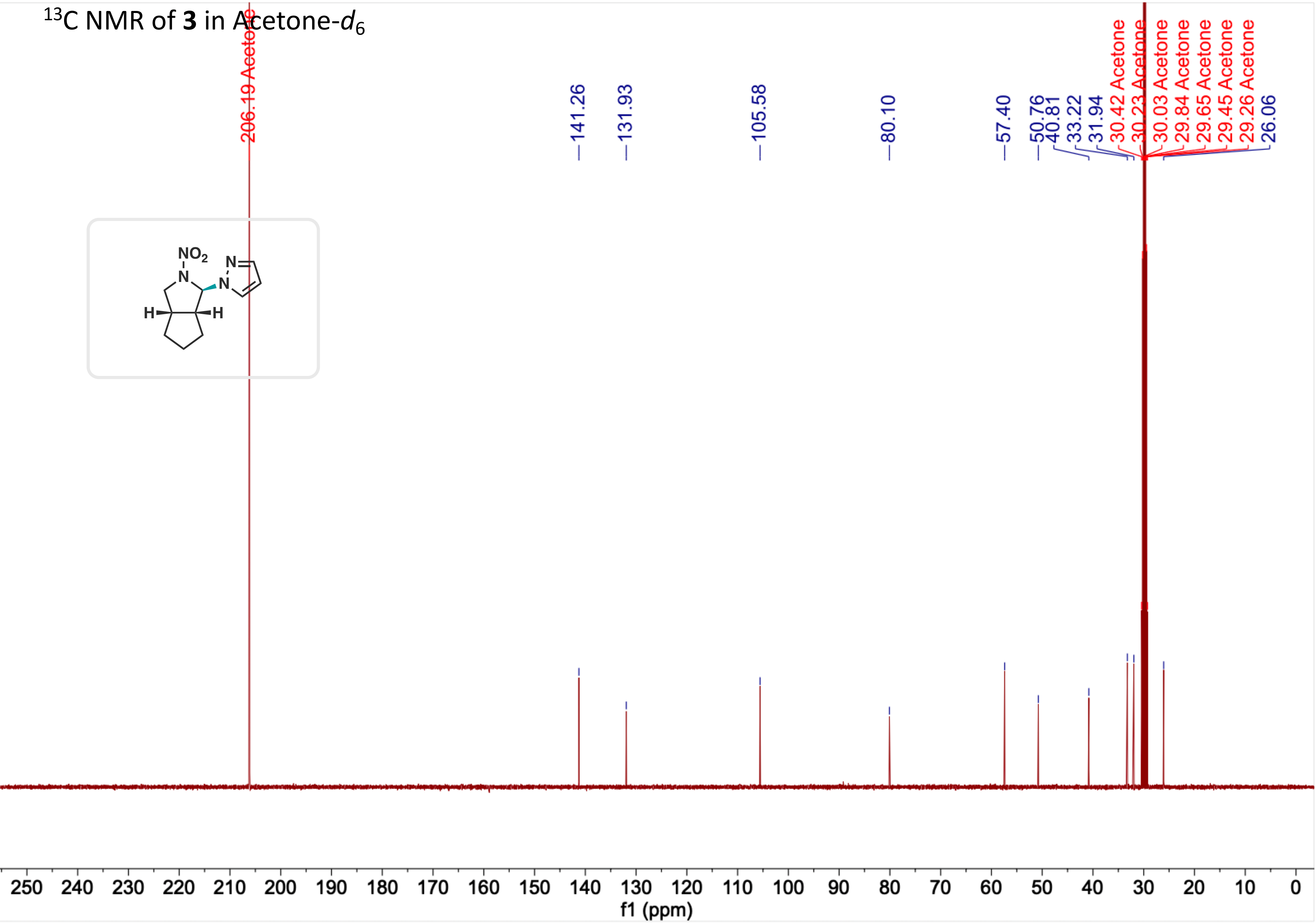

^{1}H NMR of **4** in Chloroform-*d*

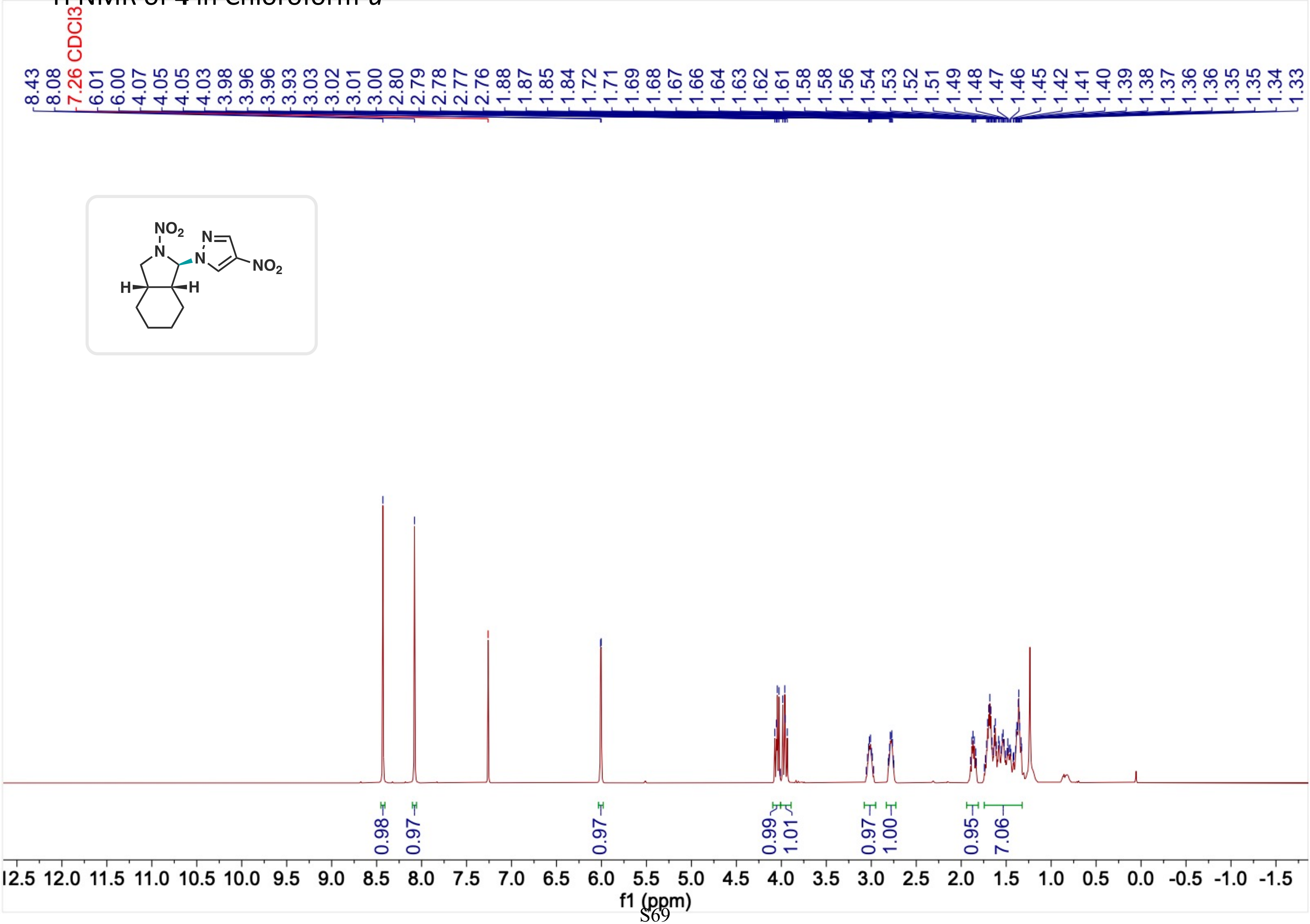

^{13}C NMR of **4** in Acetone-d_6

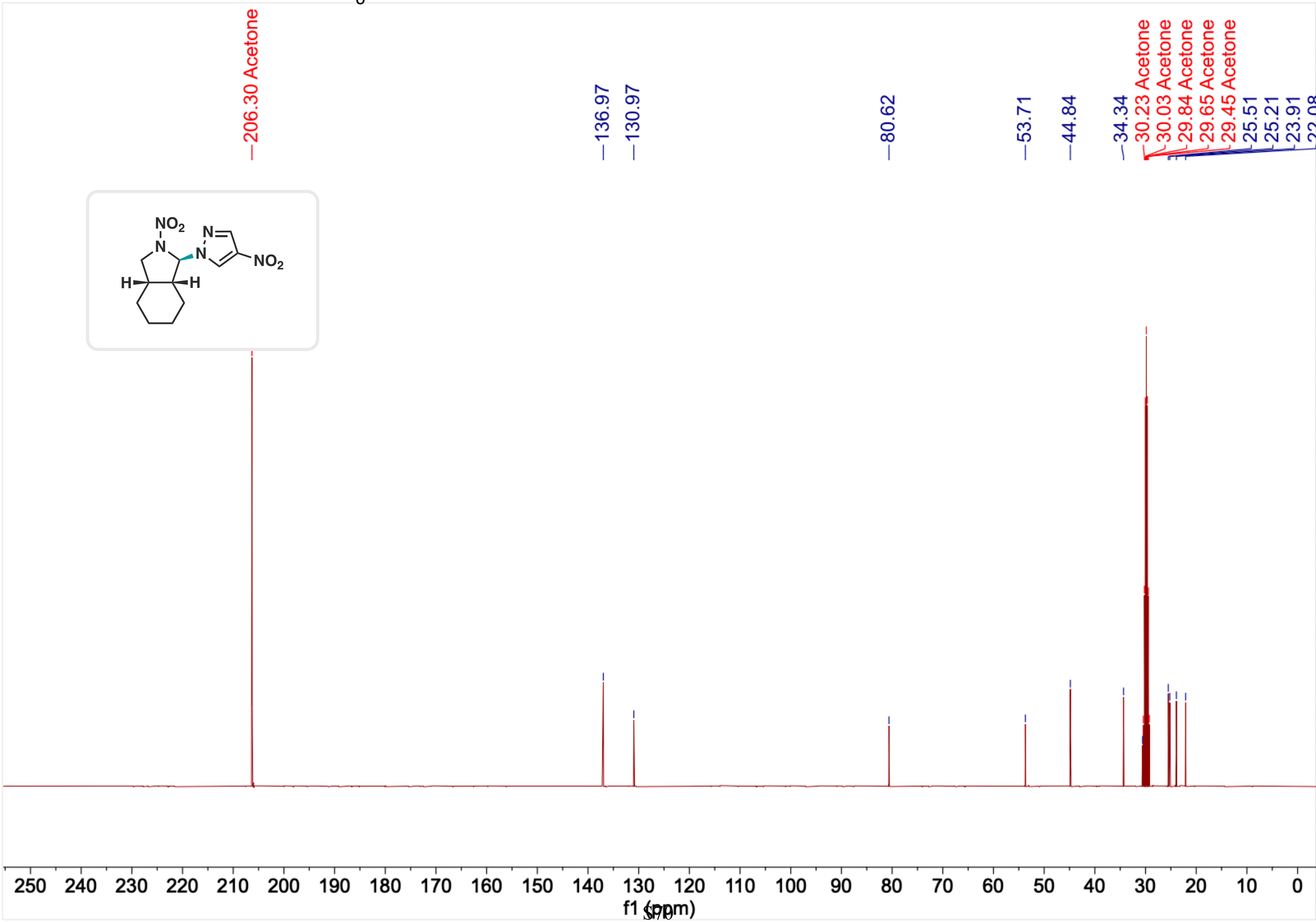

^{1}H NMR of **5** in Chloroform-*d*

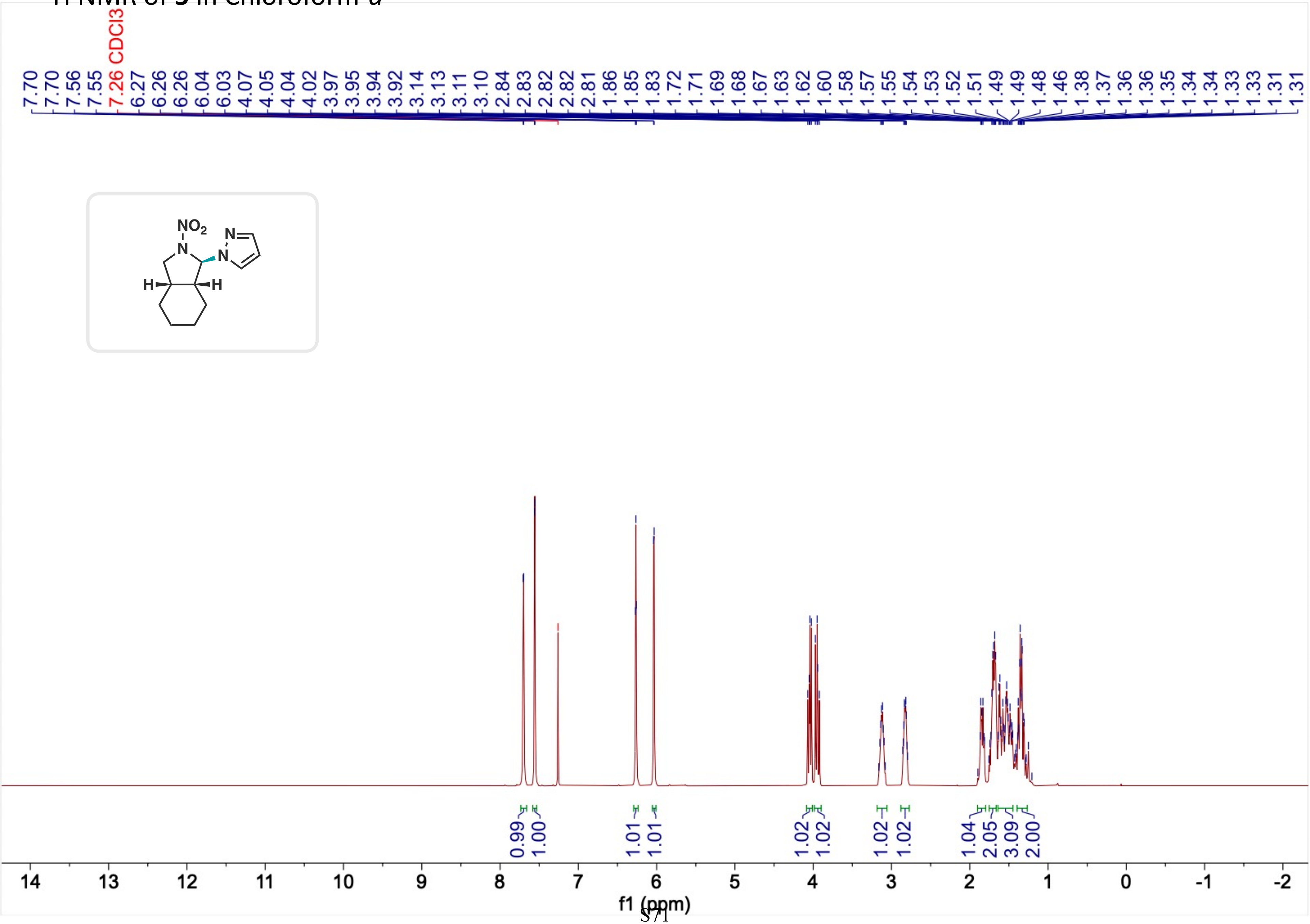

^{13}C NMR of **5** in Chloroform-*d*

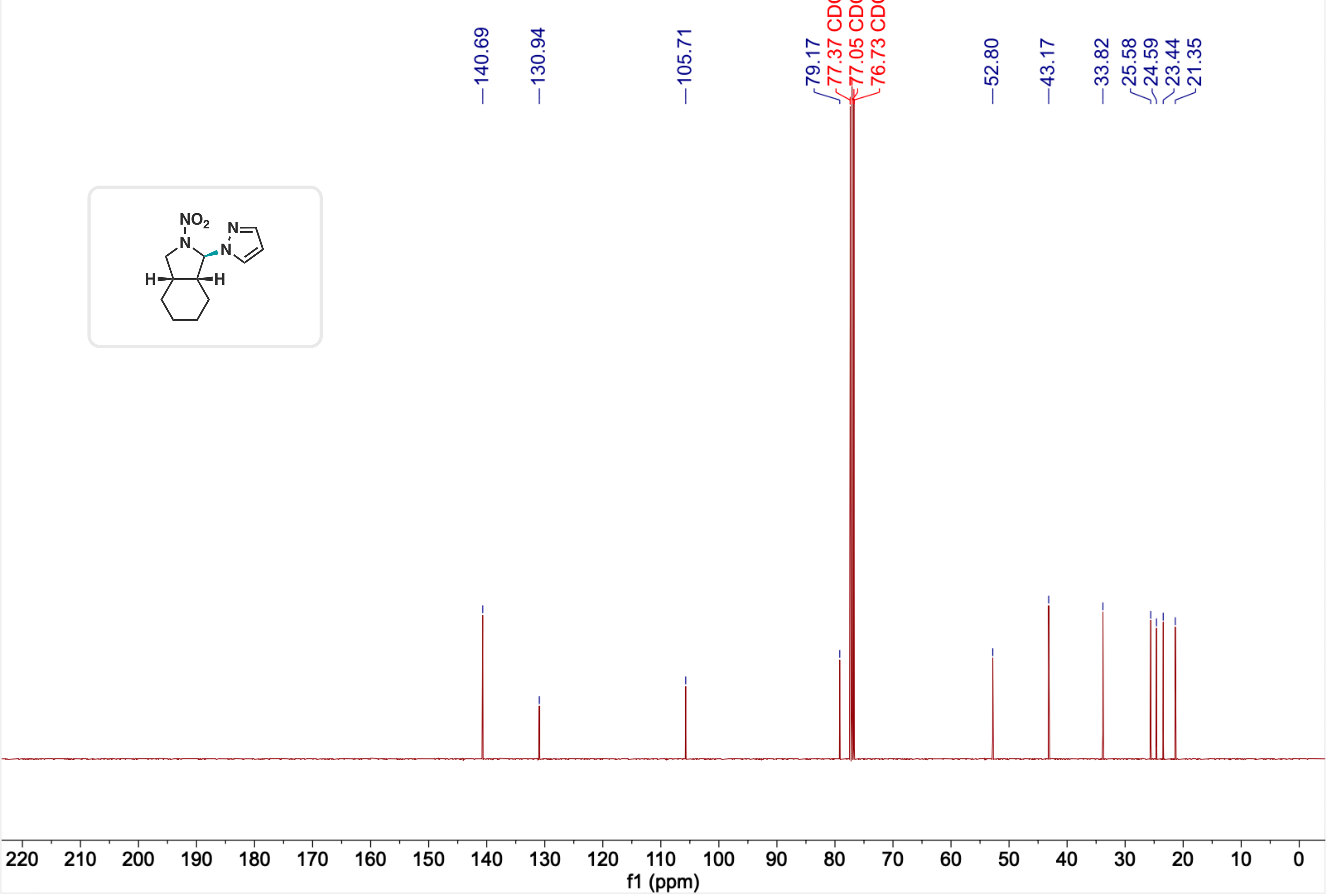

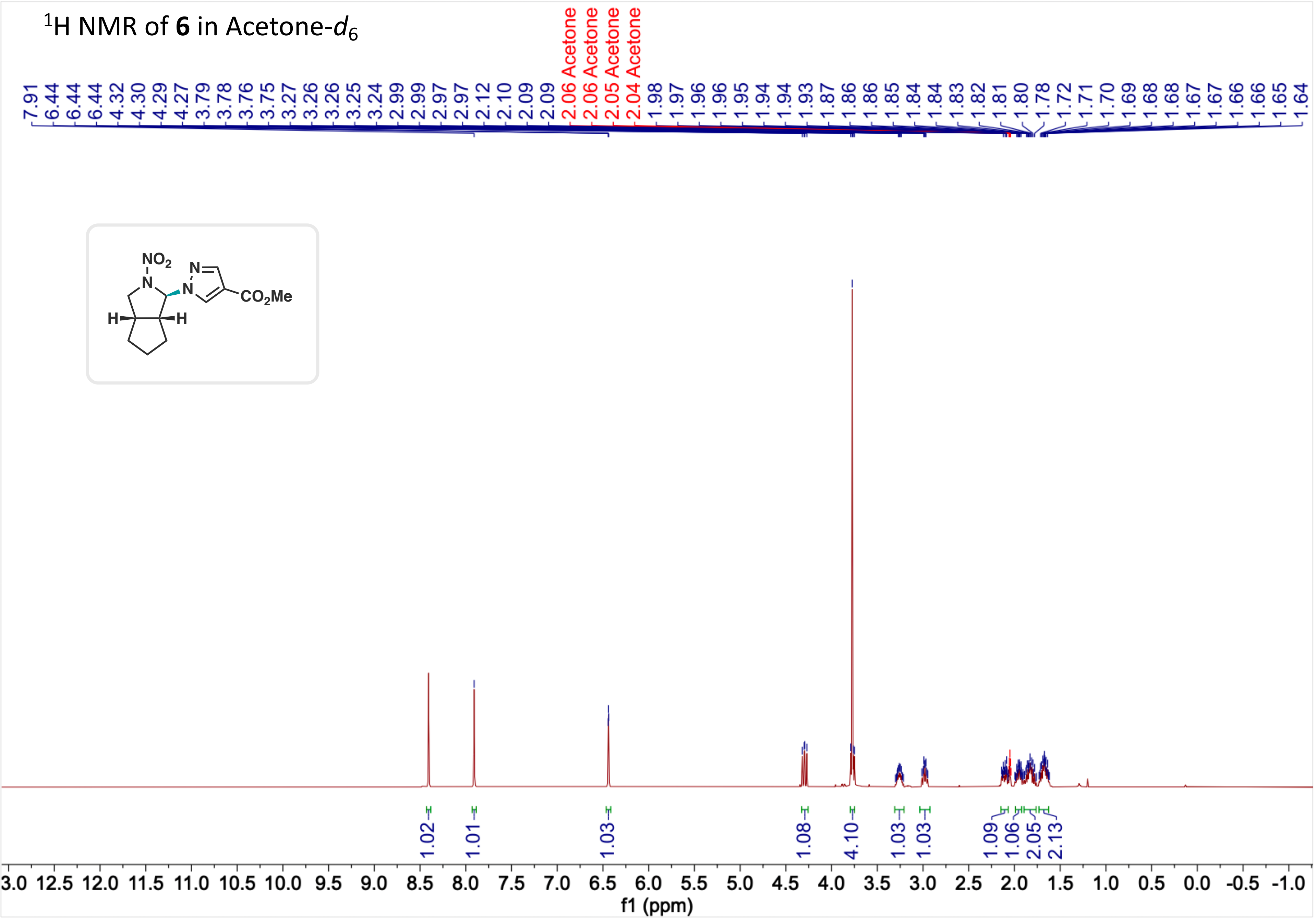

^{1}H NMR of **6** in Acetone-d_6
7.91 6.44 6.44 6.44 4.32 4.30 4.29 4.27 3.79 3.78 3.76 3.75 3.27 3.26 3.26 3.25 3.24 2.99 2.99 2.97 2.97 2.12 2.10 2.09 2.09 2.06 Acetone 2.06 Acetone 2.05 Acetone 2.04 Acetone 1.98 1.97 1.96 1.96 1.95 1.94 1.94 1.93 1.87 1.86 1.86 1.85 1.84 1.84 1.83 1.82 1.81 1.80 1.78 1.72 1.71 1.70 1.69 1.68 1.68 1.67 1.66 1.66 1.65 1.64
NO_2
N
N
N
H
H
CO_2Me
1.02
1.01
1.03
1.08
4.10
1.03
1.03
1.09
1.06
2.05
2.13
13.0 12.5 12.0 11.5 11.0 10.5 10.0 9.5 9.0 8.5 8.0 7.5 7.0 6.5 6.0 5.5 5.0 4.5 4.0 3.5 3.0 2.5 2.0 1.5 1.0 0.5 0.0 -0.5 -1.0
f1 (ppm)

13C NMR of **6** in Acetone-d_6

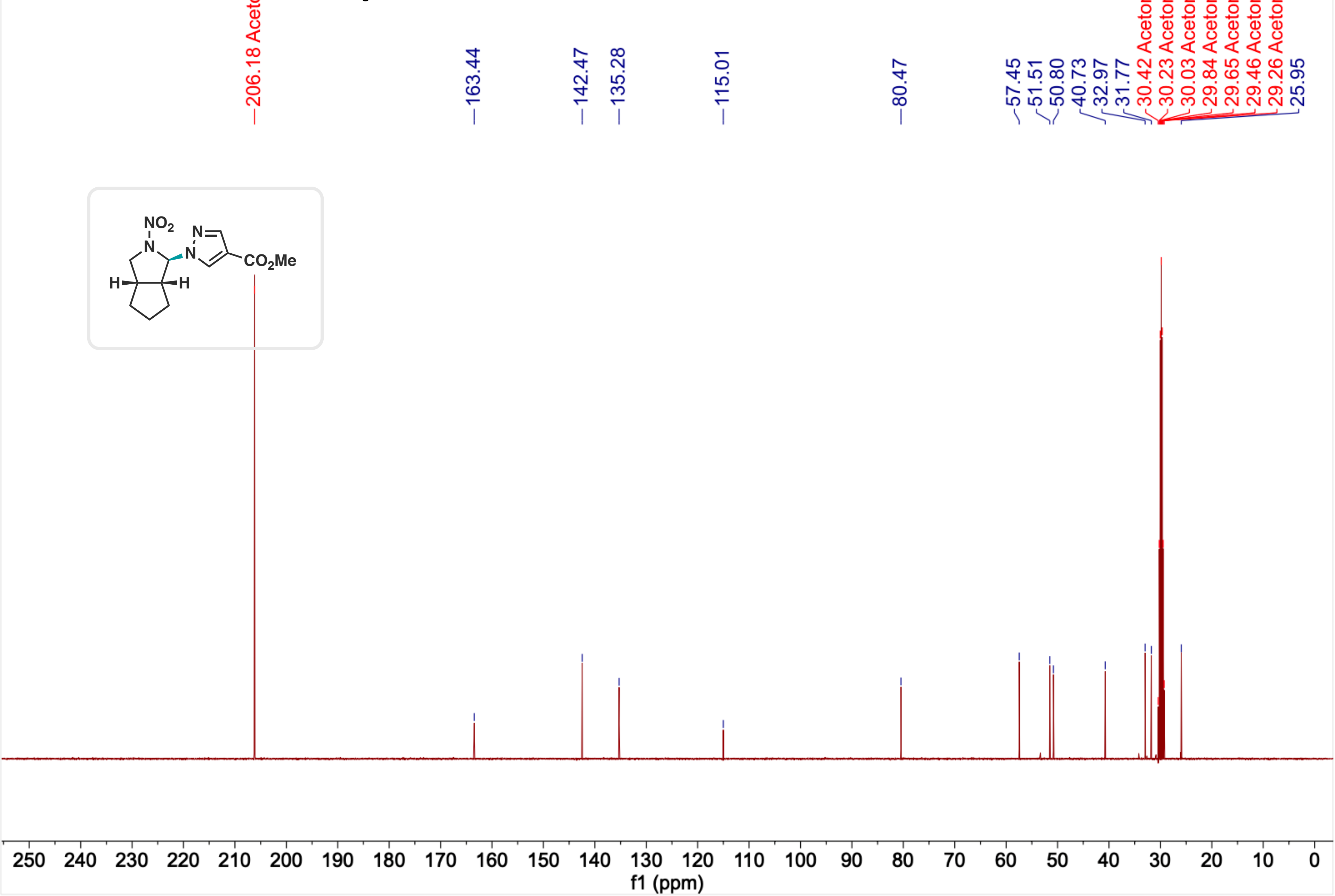

^{1}H NMR of **7** in Chloroform-*d*

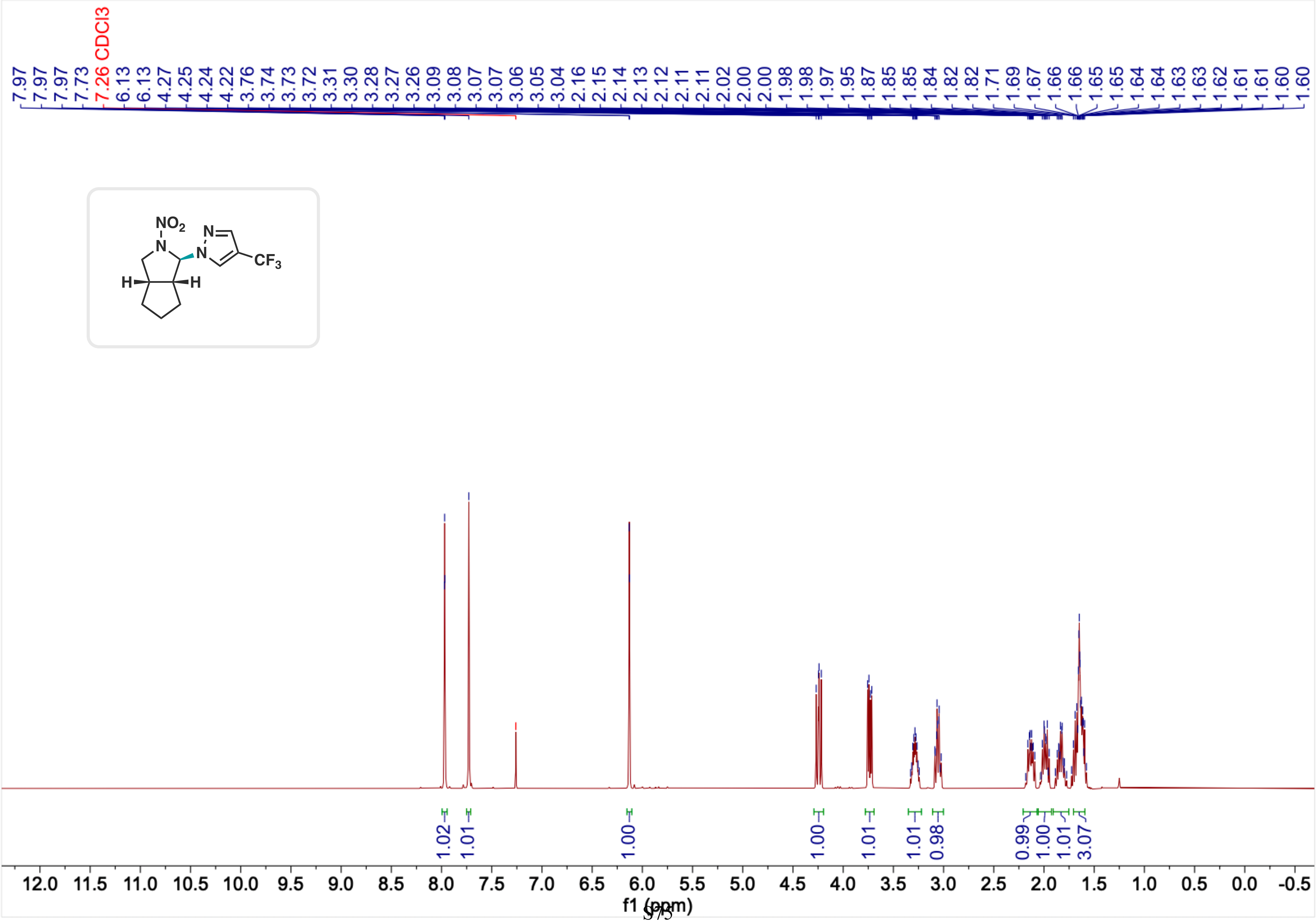

^{19}F NMR of **7** in Chloroform-*d*

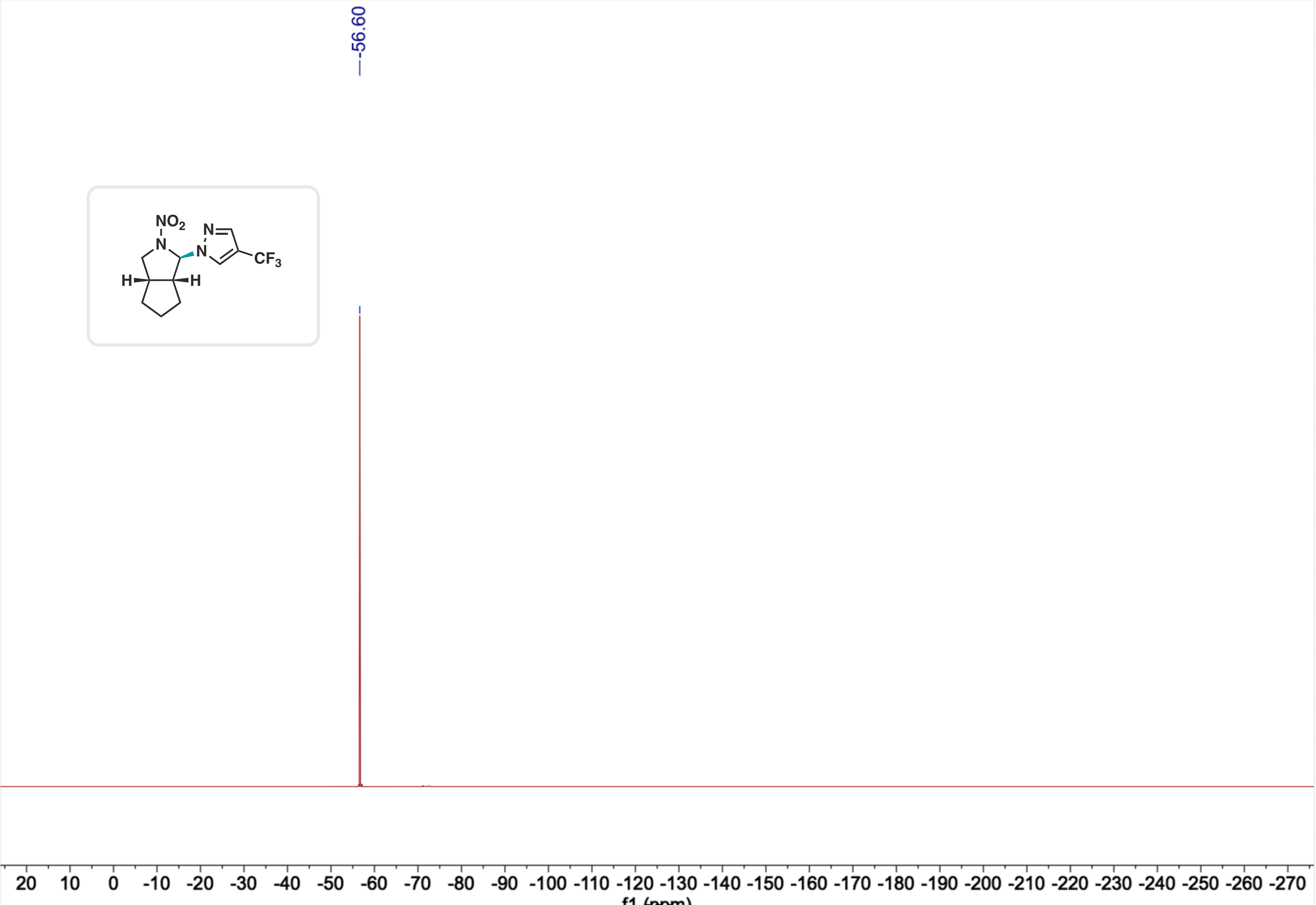

^{13}C NMR of **7** in Chloroform-*d*

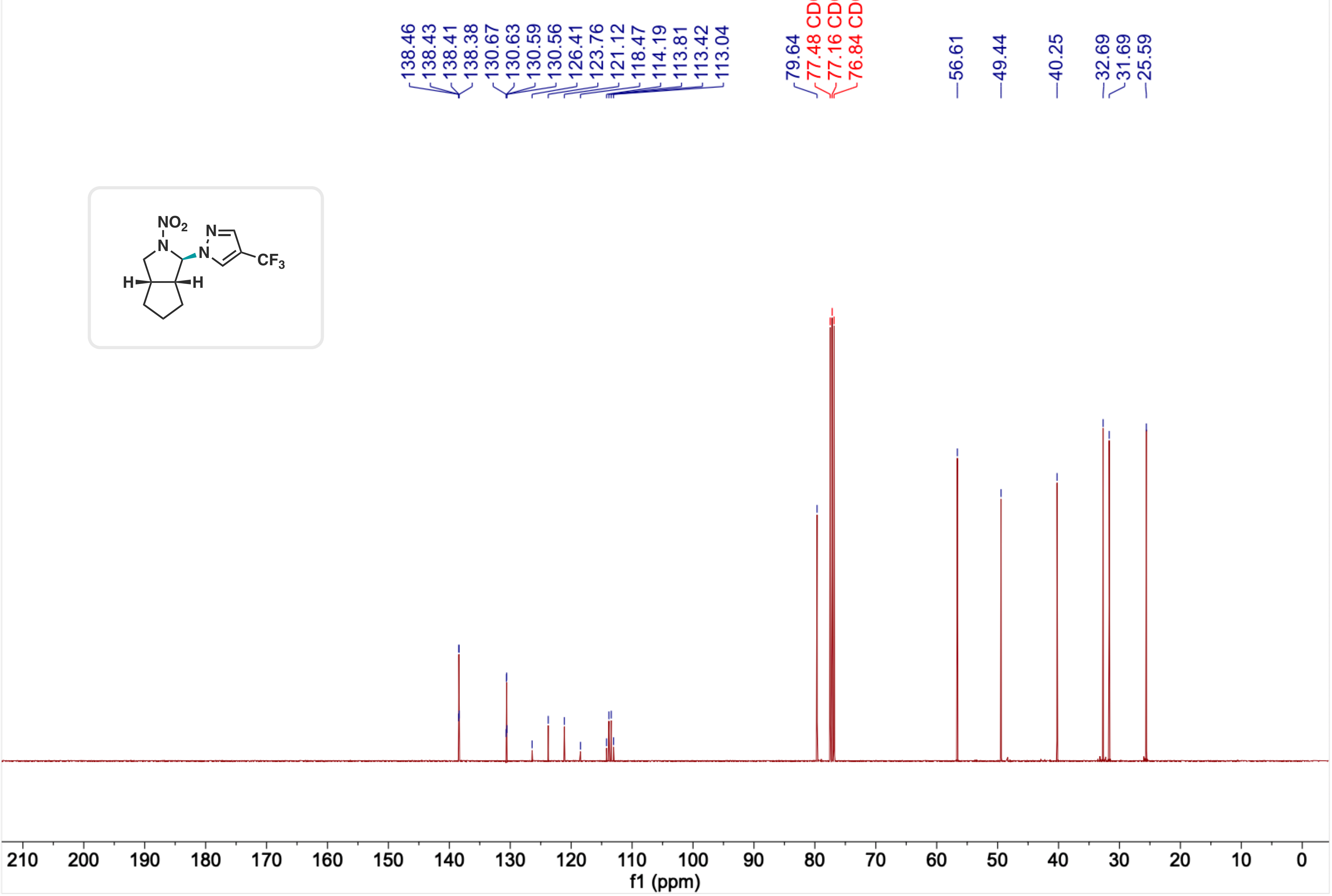

^{1}H NMR of **8** in Acetone-d_6

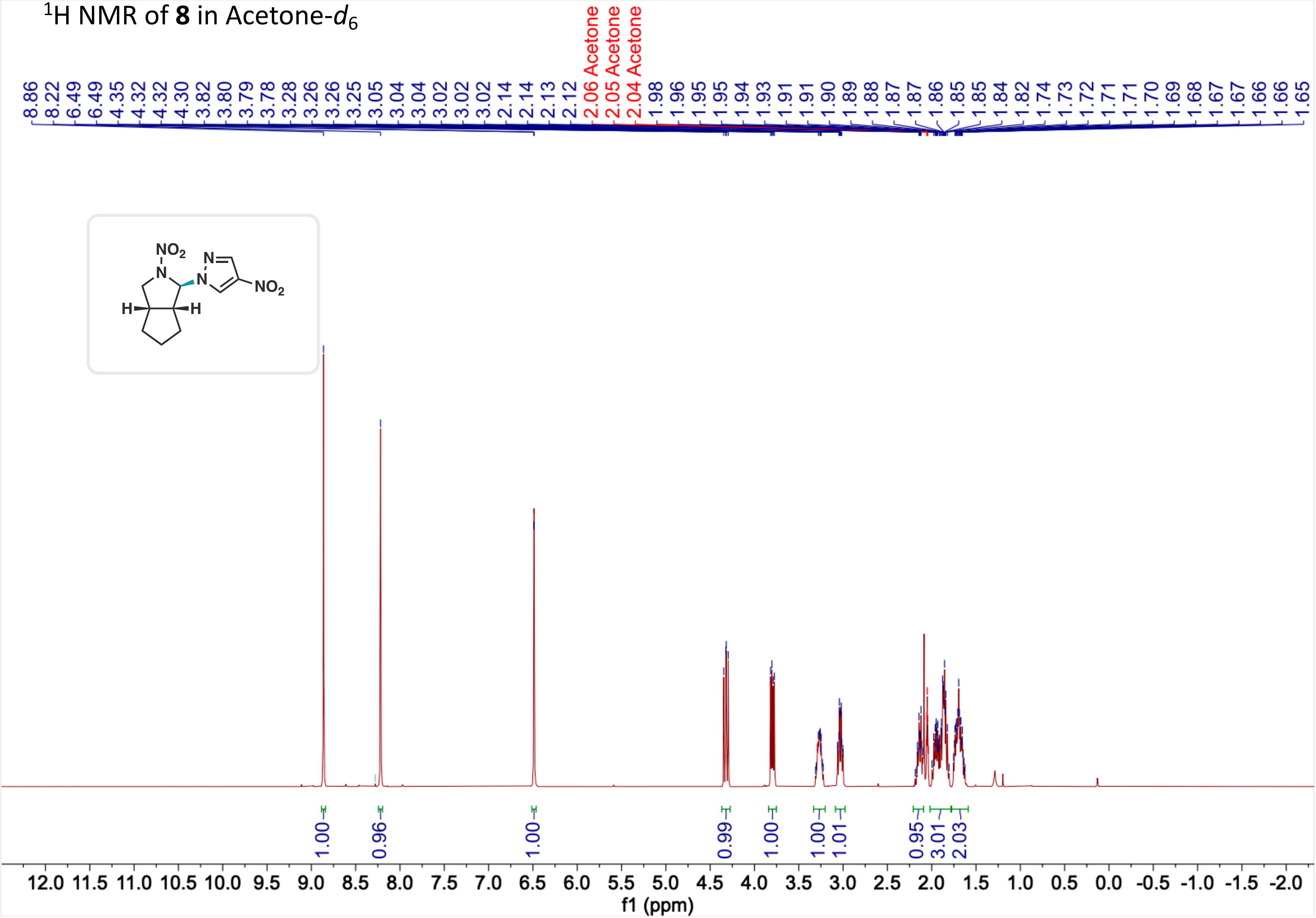

^{13}C NMR of **8** in Acetone-d_6

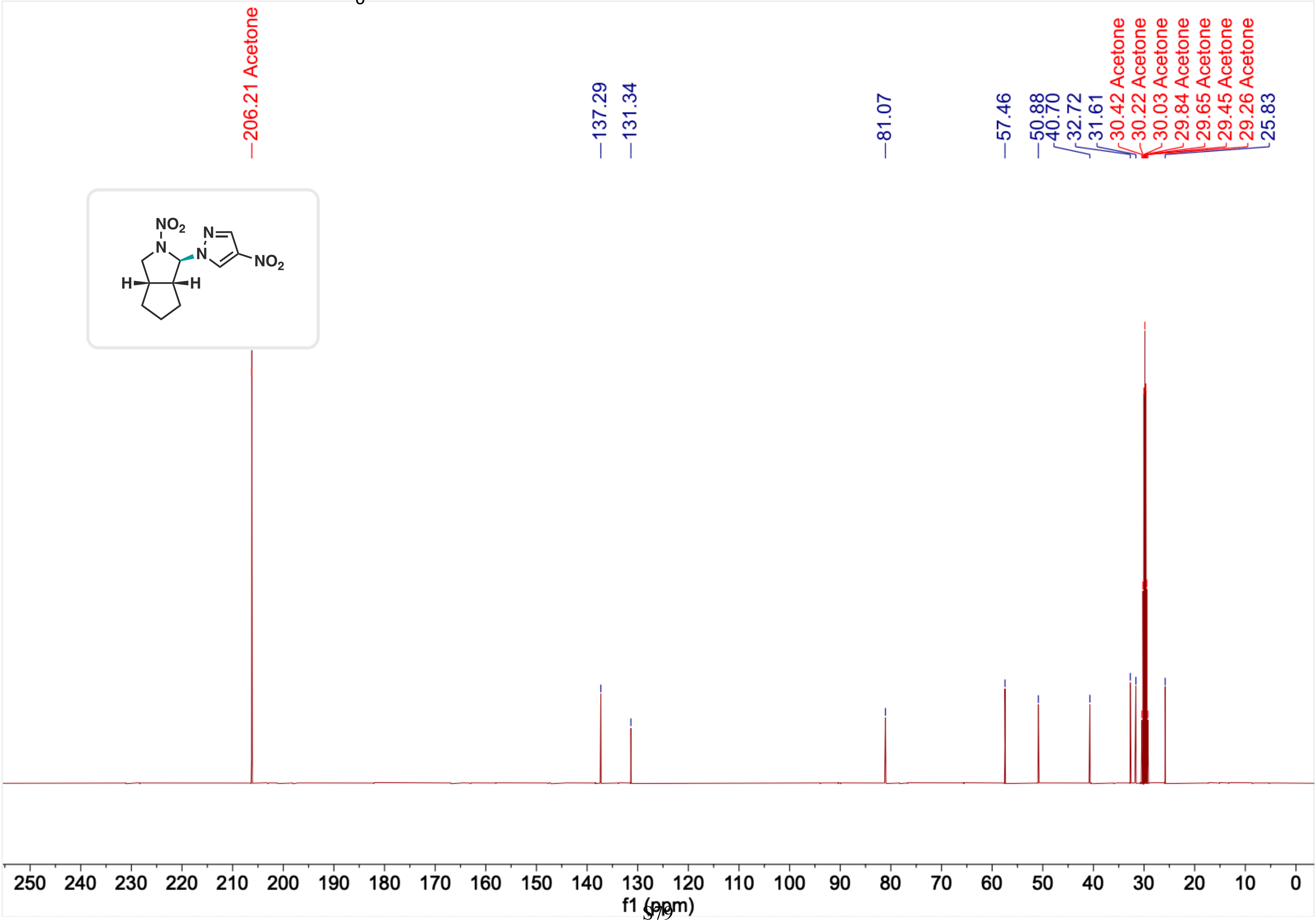

^{1}H NMR of **9** in Chloroform-*d*

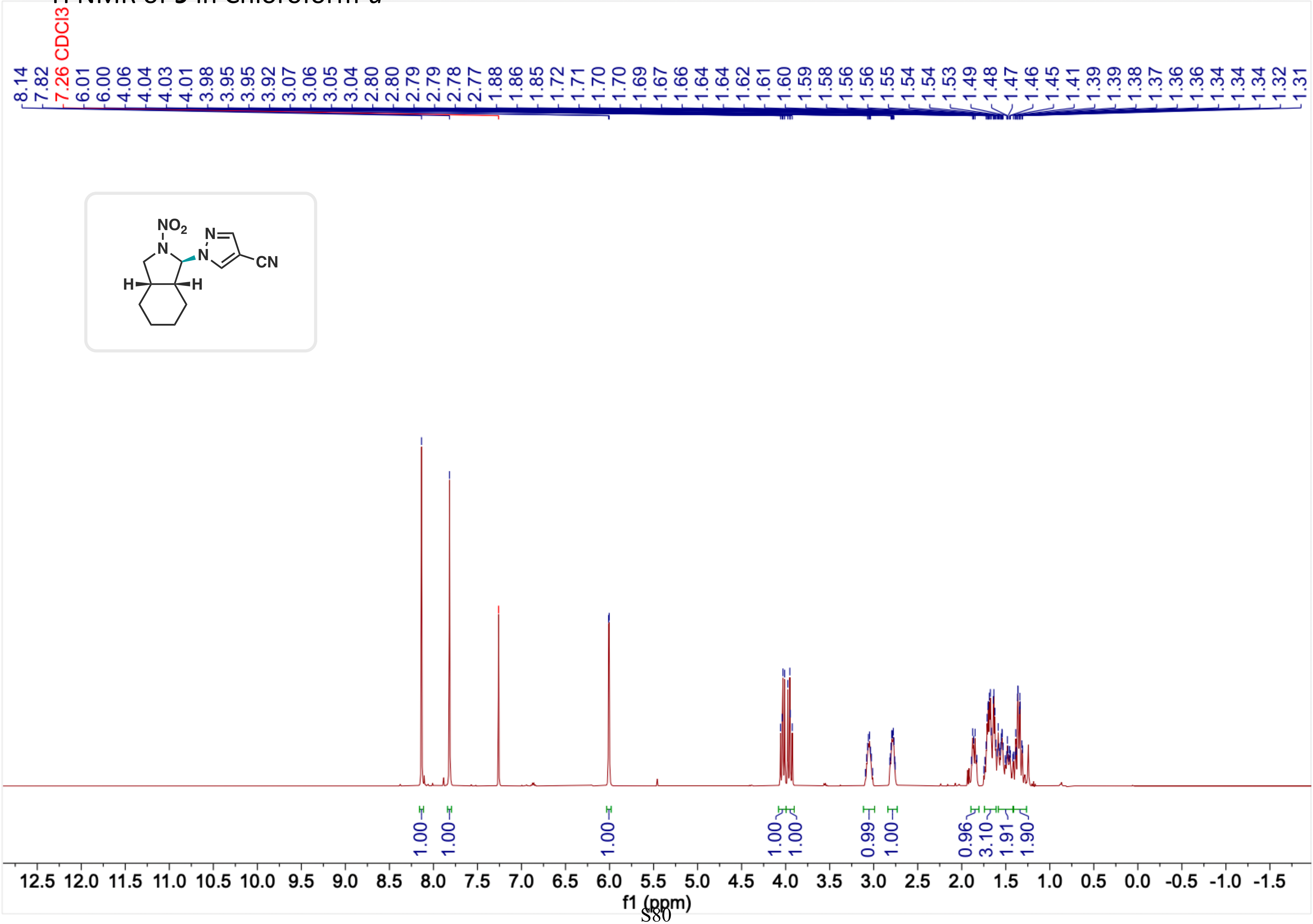

^{13}C NMR of **9** in Chloroform-*d*

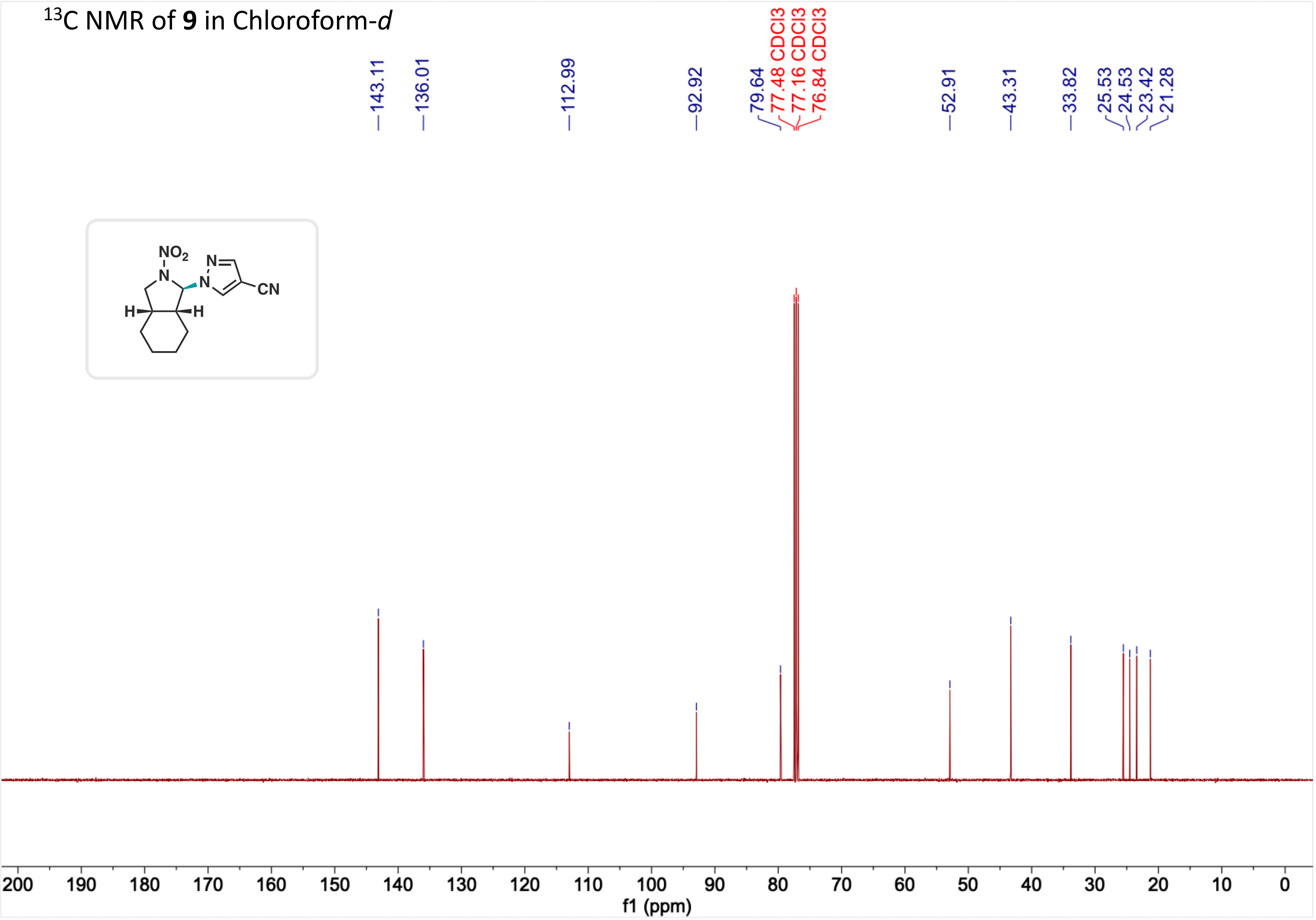

^{1}H NMR of **10** in Chloroform-*d*

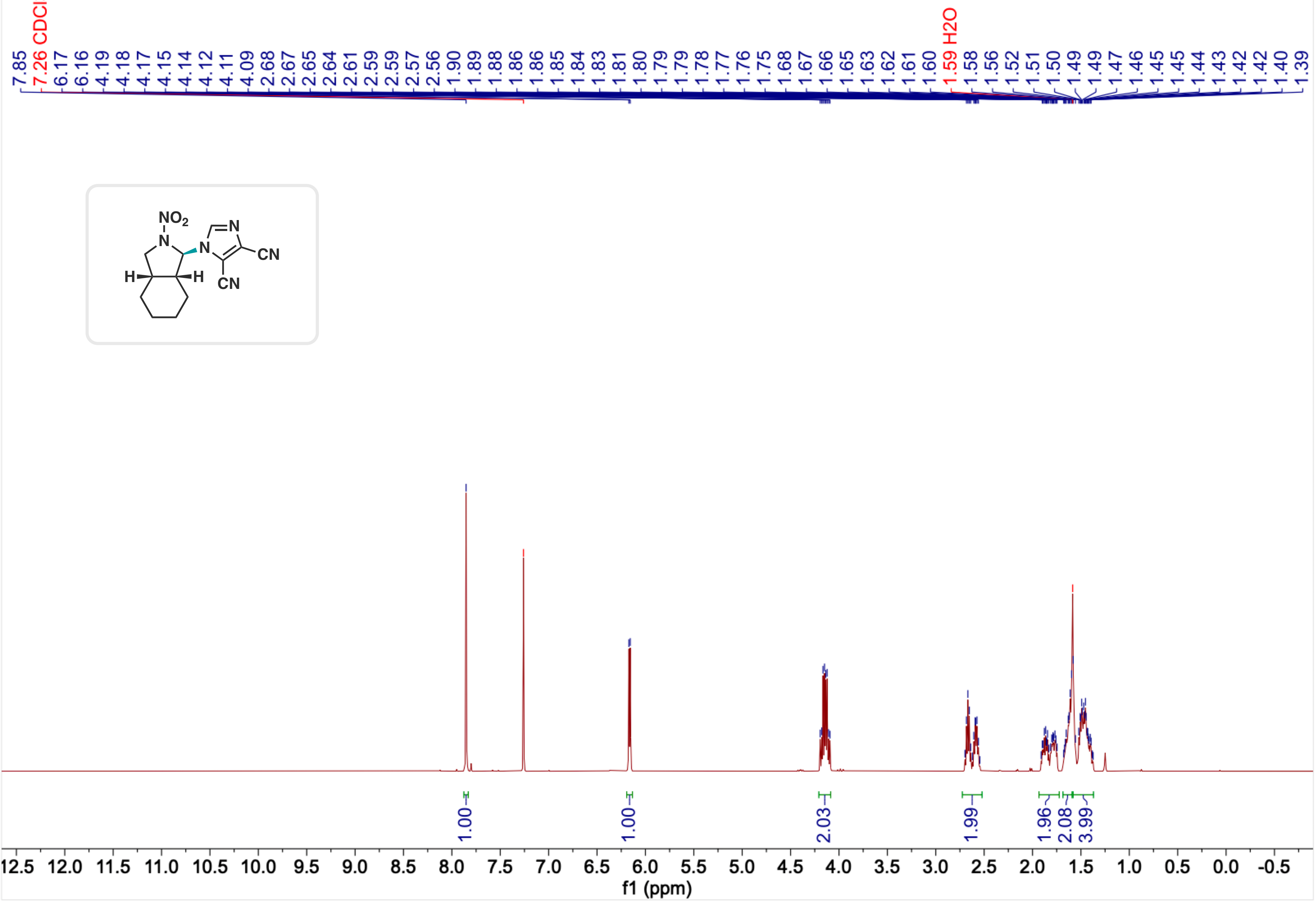

^{13}C NMR of **10** in Chloroform-*d*

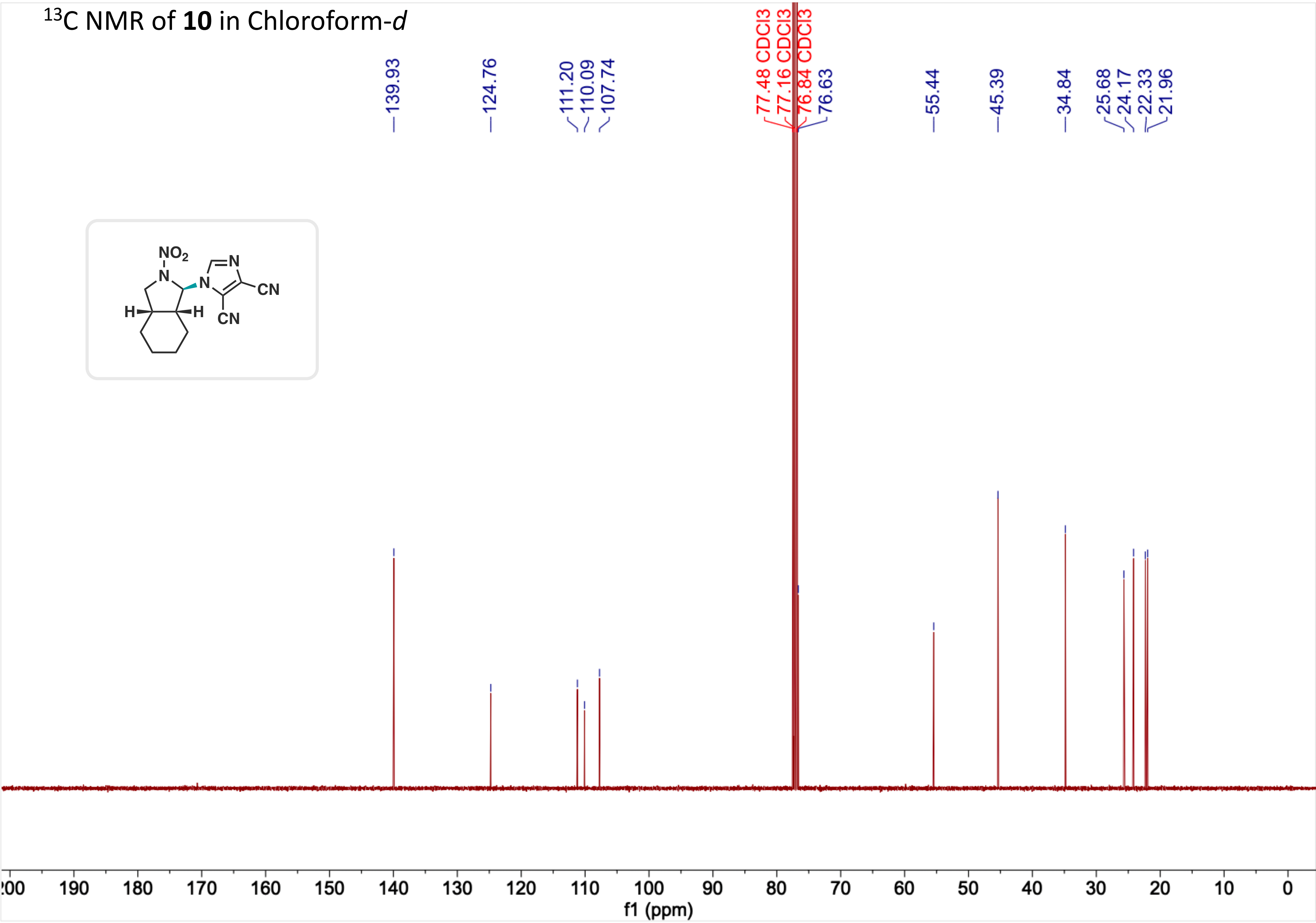

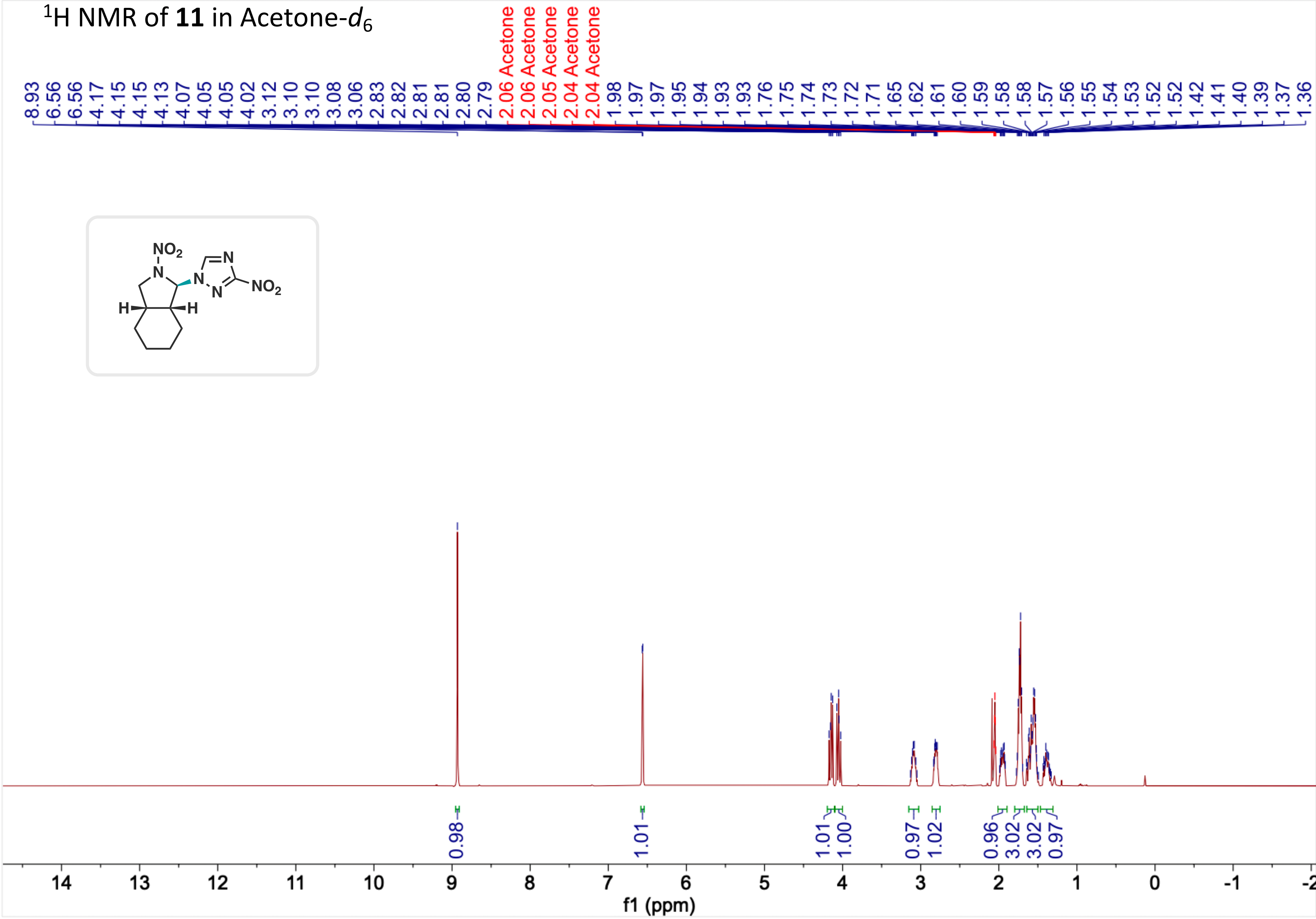

^{1}H NMR of **11** in Acetone-d_6
8.93 6.56 6.56 4.17 4.15 4.15 4.13 4.07 4.05 4.05 4.02 3.12 3.10 3.10 3.08 3.06 2.83 2.82 2.81 2.81 2.80 2.79
2.06 Acetone 2.06 Acetone 2.05 Acetone 2.04 Acetone 2.04 Acetone
1.98 1.97 1.97 1.95 1.94 1.93 1.93 1.76 1.75 1.74 1.73 1.72 1.71 1.65 1.62 1.61 1.60 1.59 1.58 1.58 1.57 1.56 1.55 1.54 1.53 1.52 1.52 1.42 1.41 1.40 1.39 1.37 1.36
0.98 1.01 1.01 1.00 0.97 1.02 0.96 3.02 3.02 0.97
f1 (ppm)

^{13}C NMR of **11** in Acetone-d_6

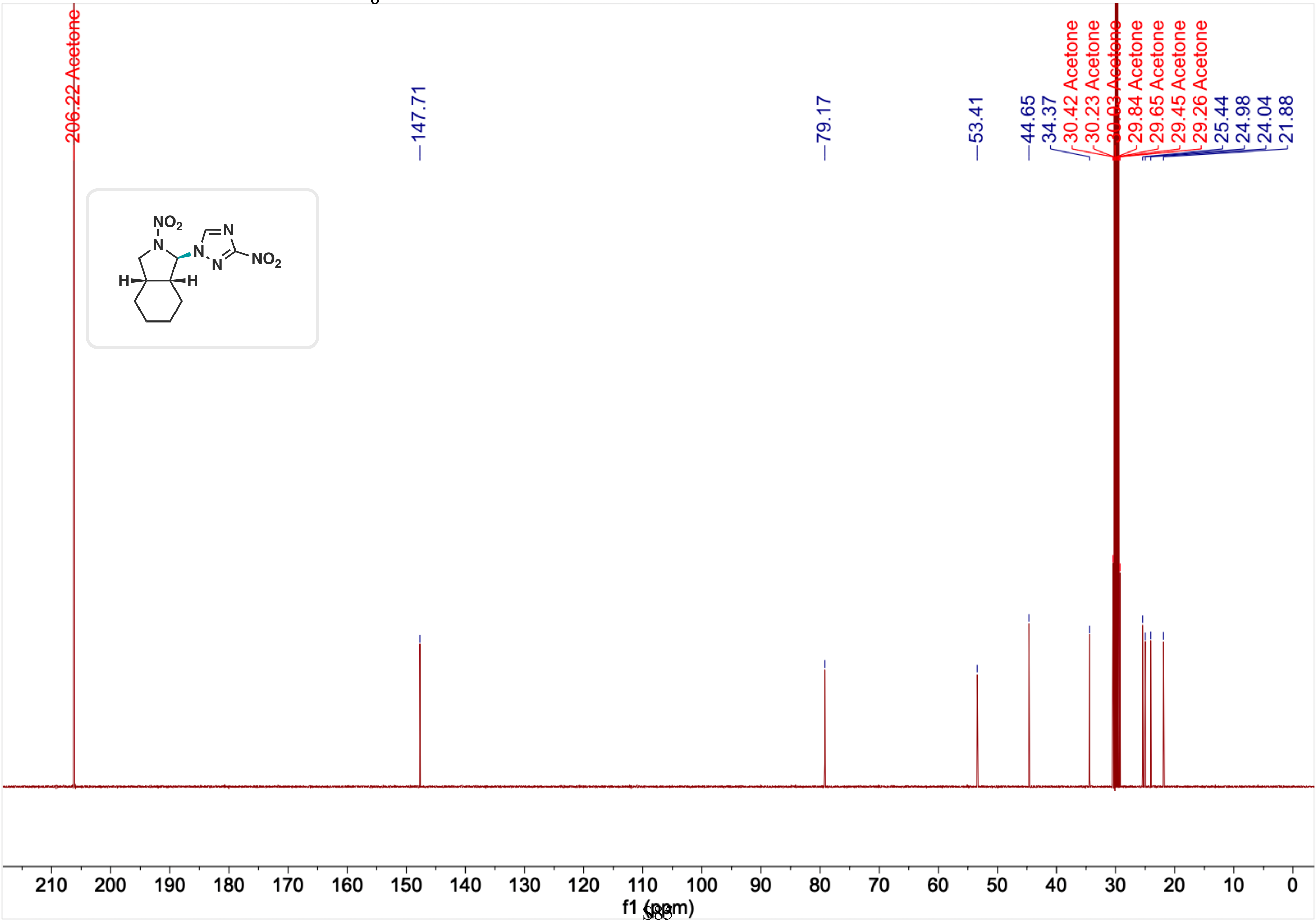

^1H-^{13}C HMBC NMR of **11** in Chloroform-*d*

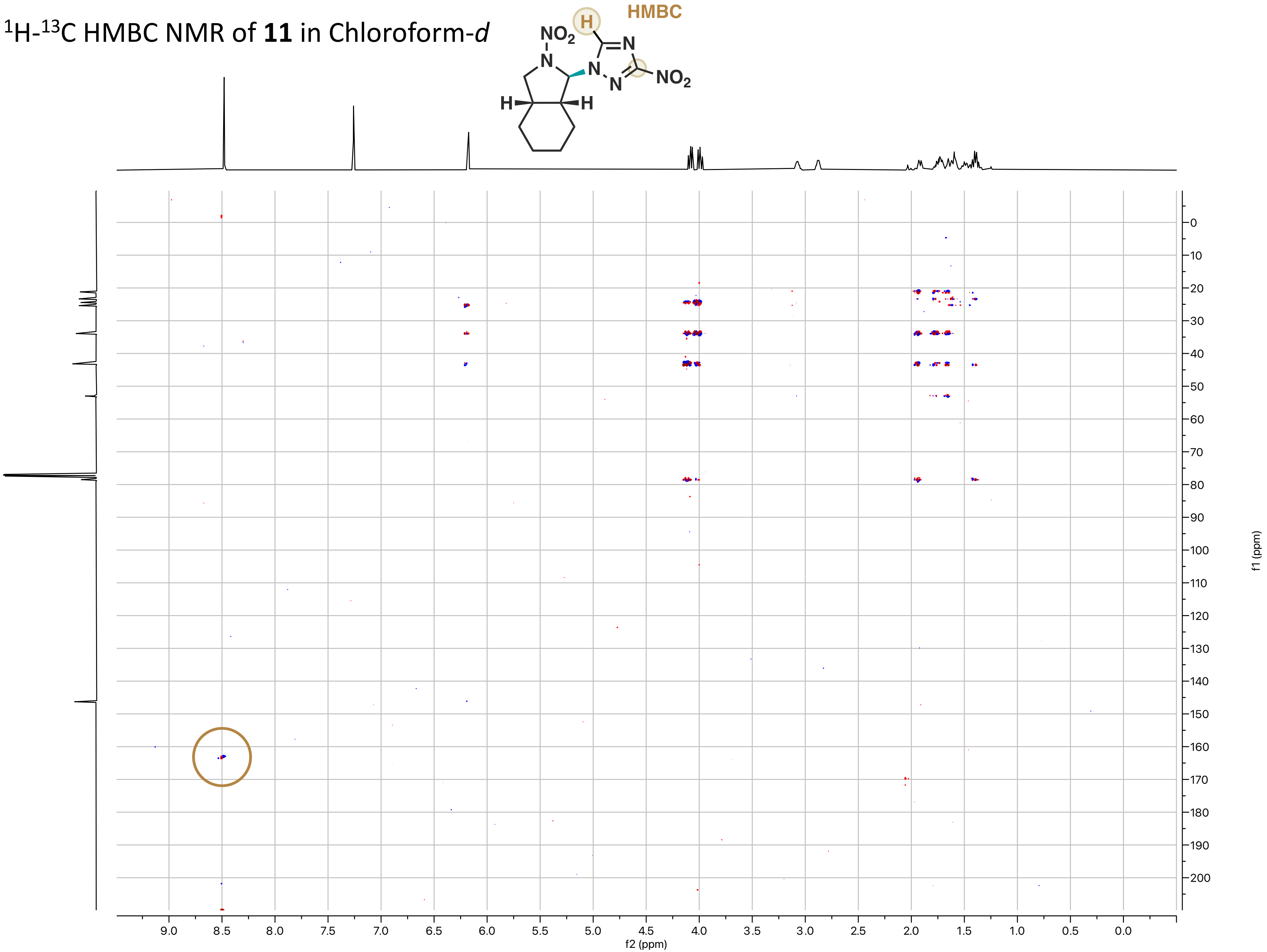

NOESY NMR of **11** in Chloroform-*d*

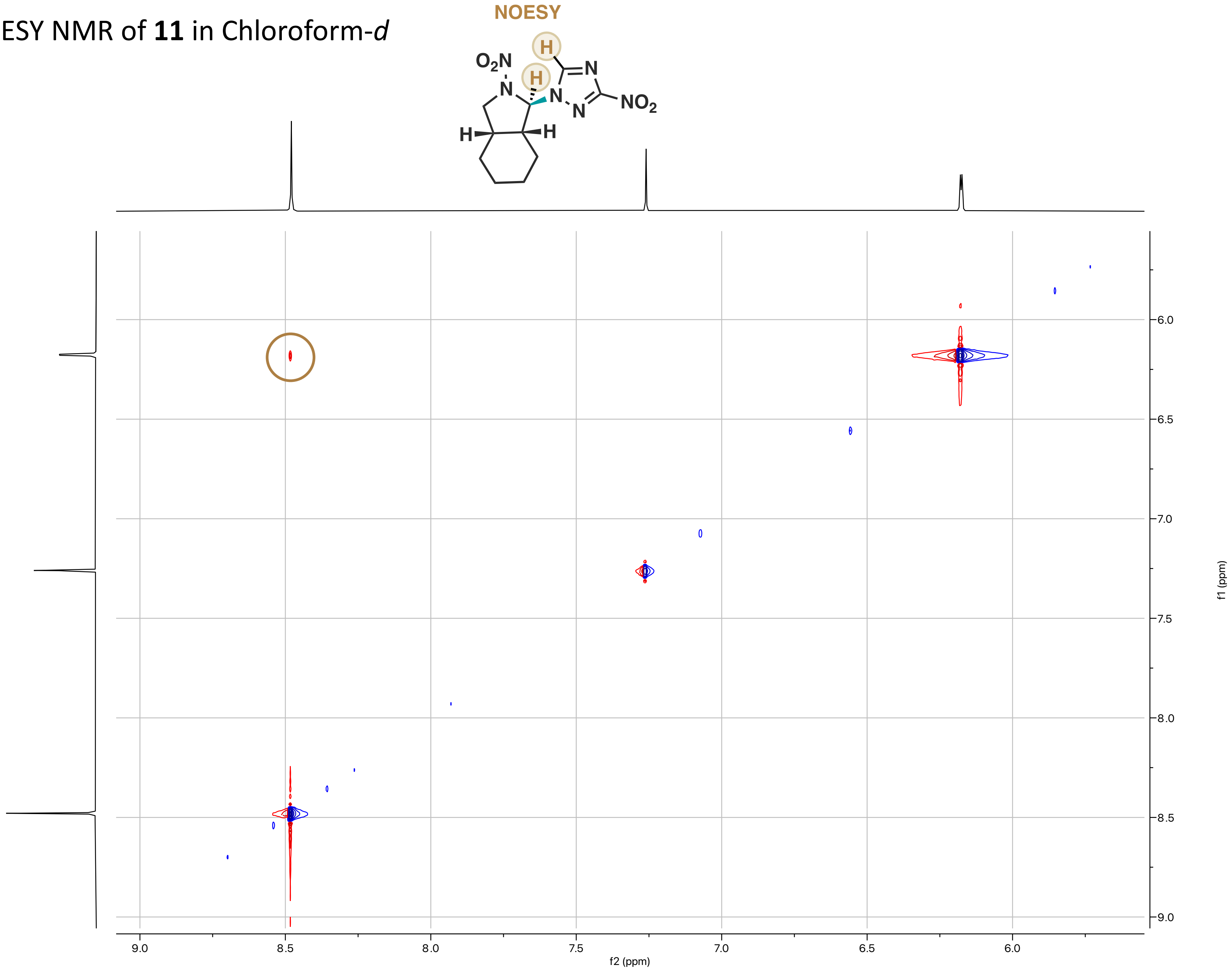

^{1}H NMR of **12** in Chloroform-*d*

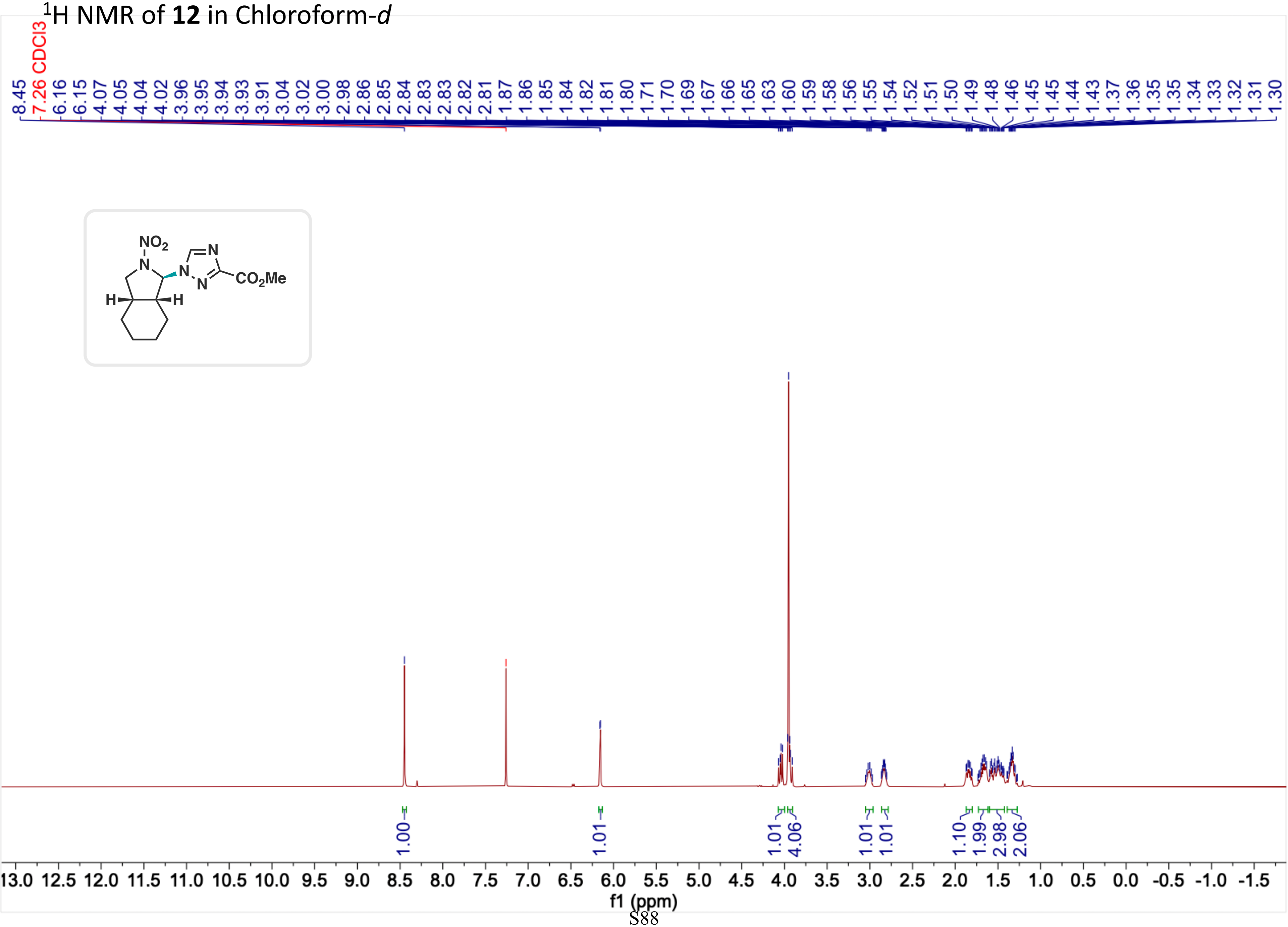

^{13}C NMR of **12** in Chloroform-*d*

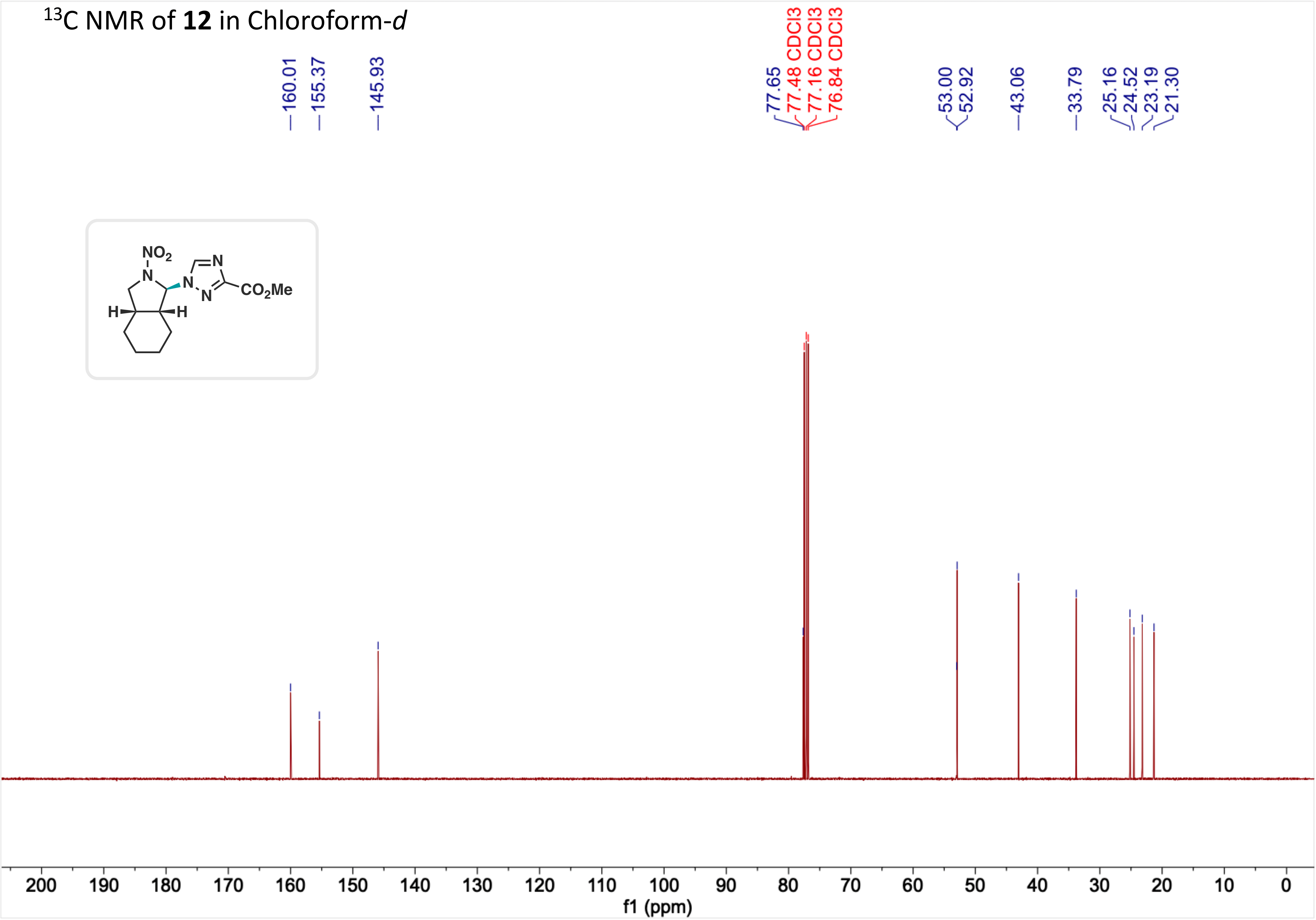

^{1}H NMR of **13** in Chloroform-*d*

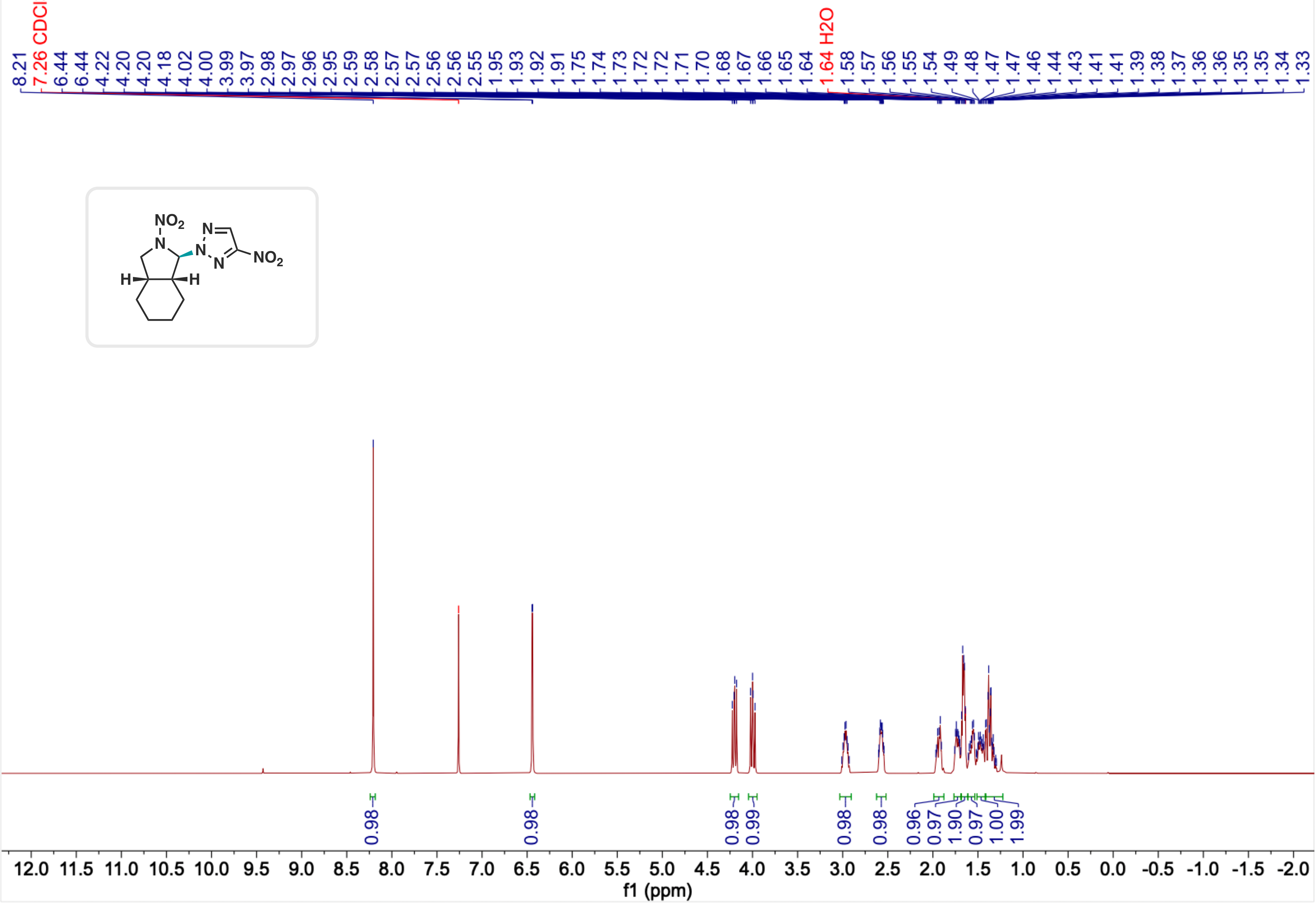

^{13}C NMR of **13** in Chloroform-*d*

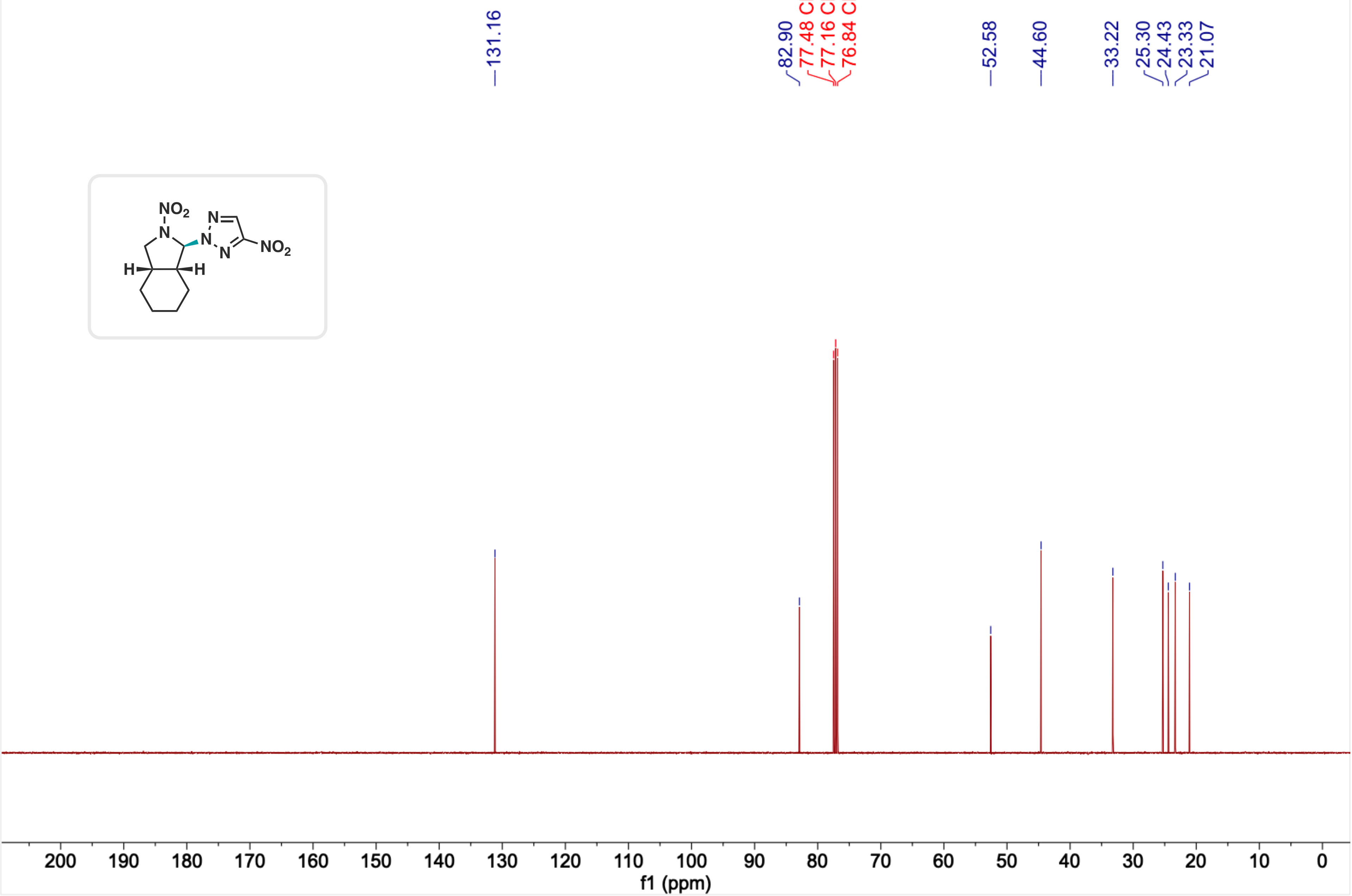

^{1}H NMR of **14** in Chloroform-*d*

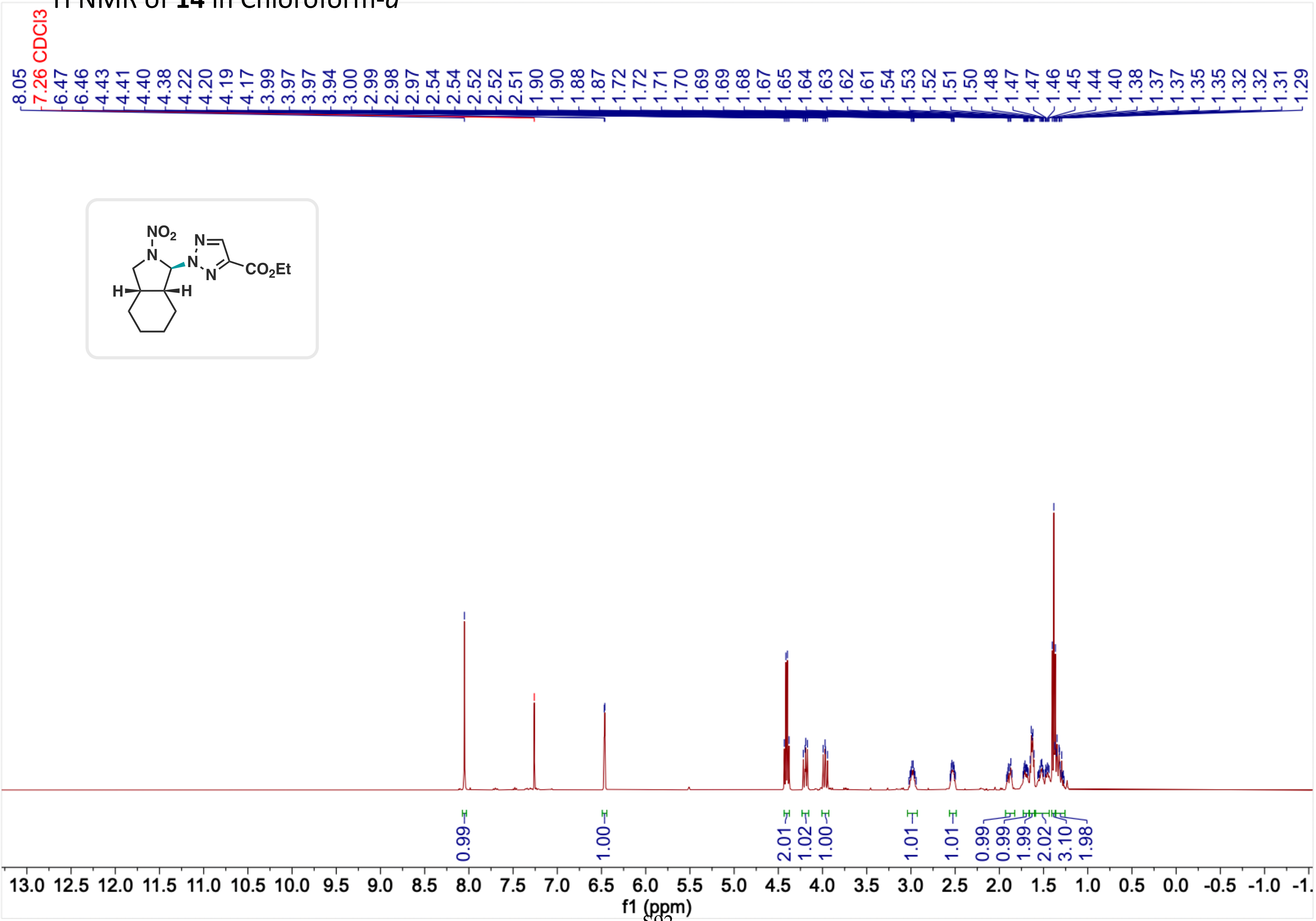

^{13}C NMR of **14** in Chloroform-*d*

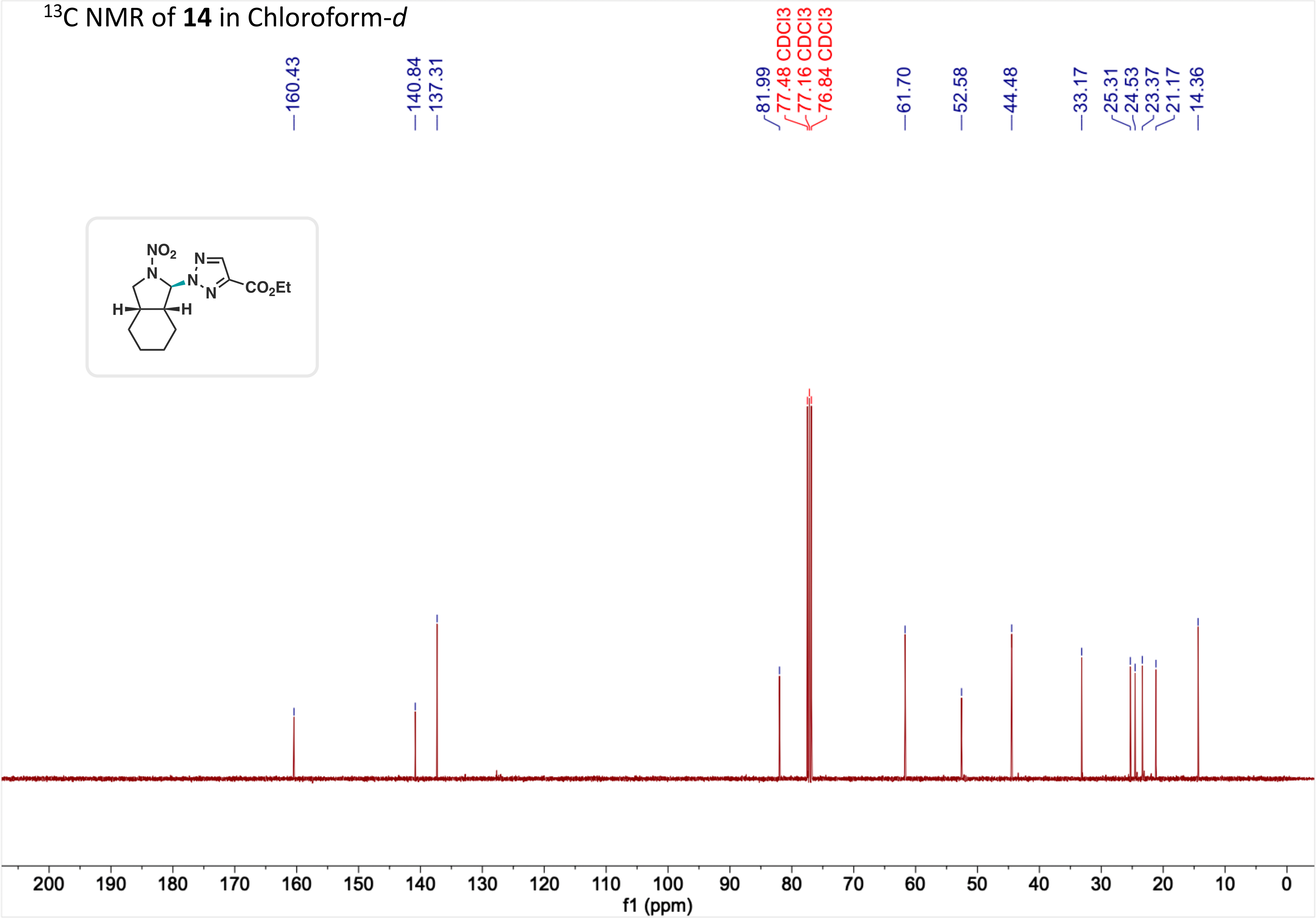

^{1}H-^{15}N HMBC NMR of **14** in Chloroform-*d*

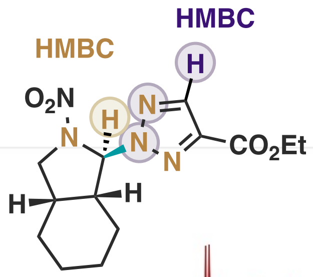

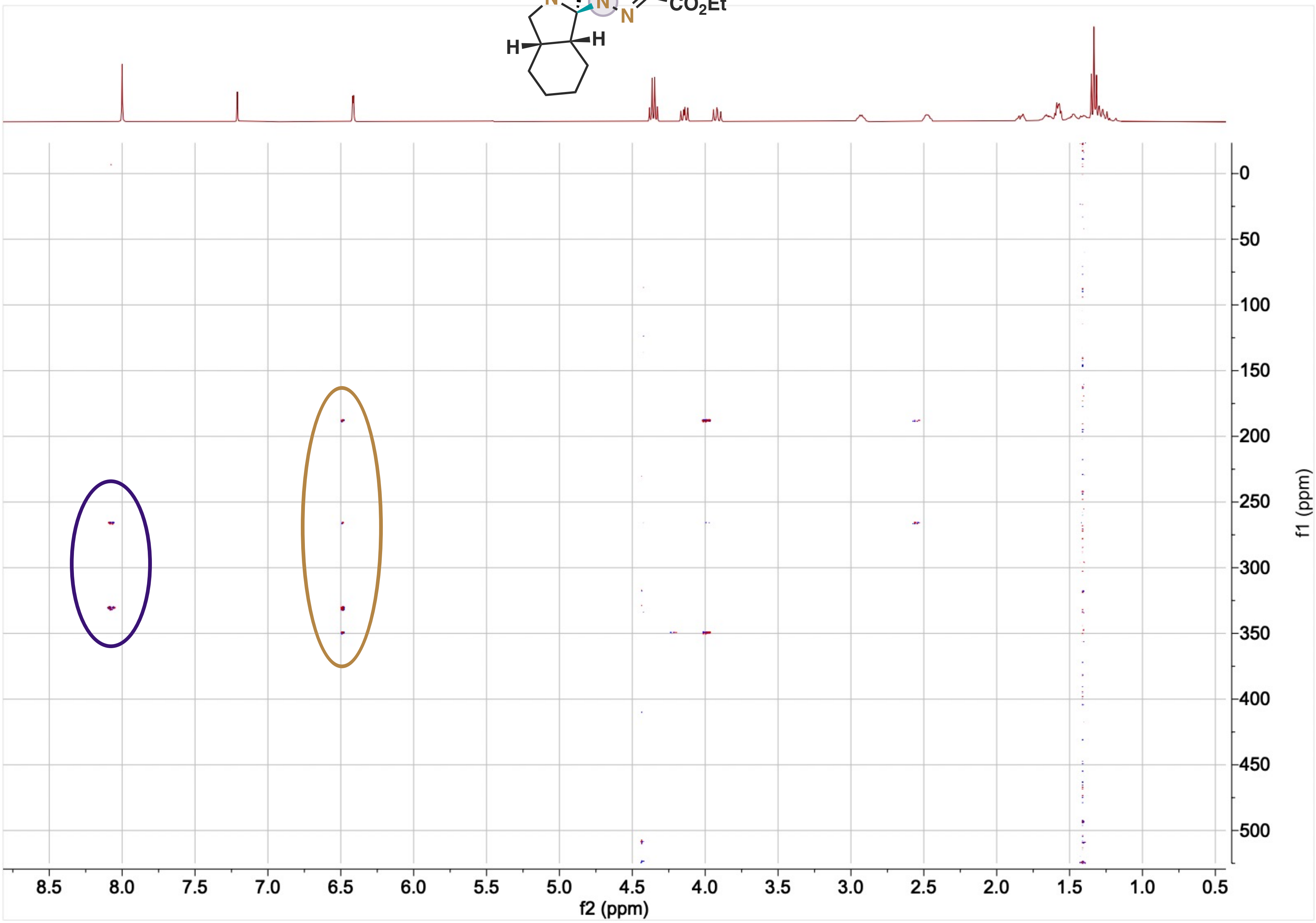

^{1}H NMR of **15** in Chloroform-*d*

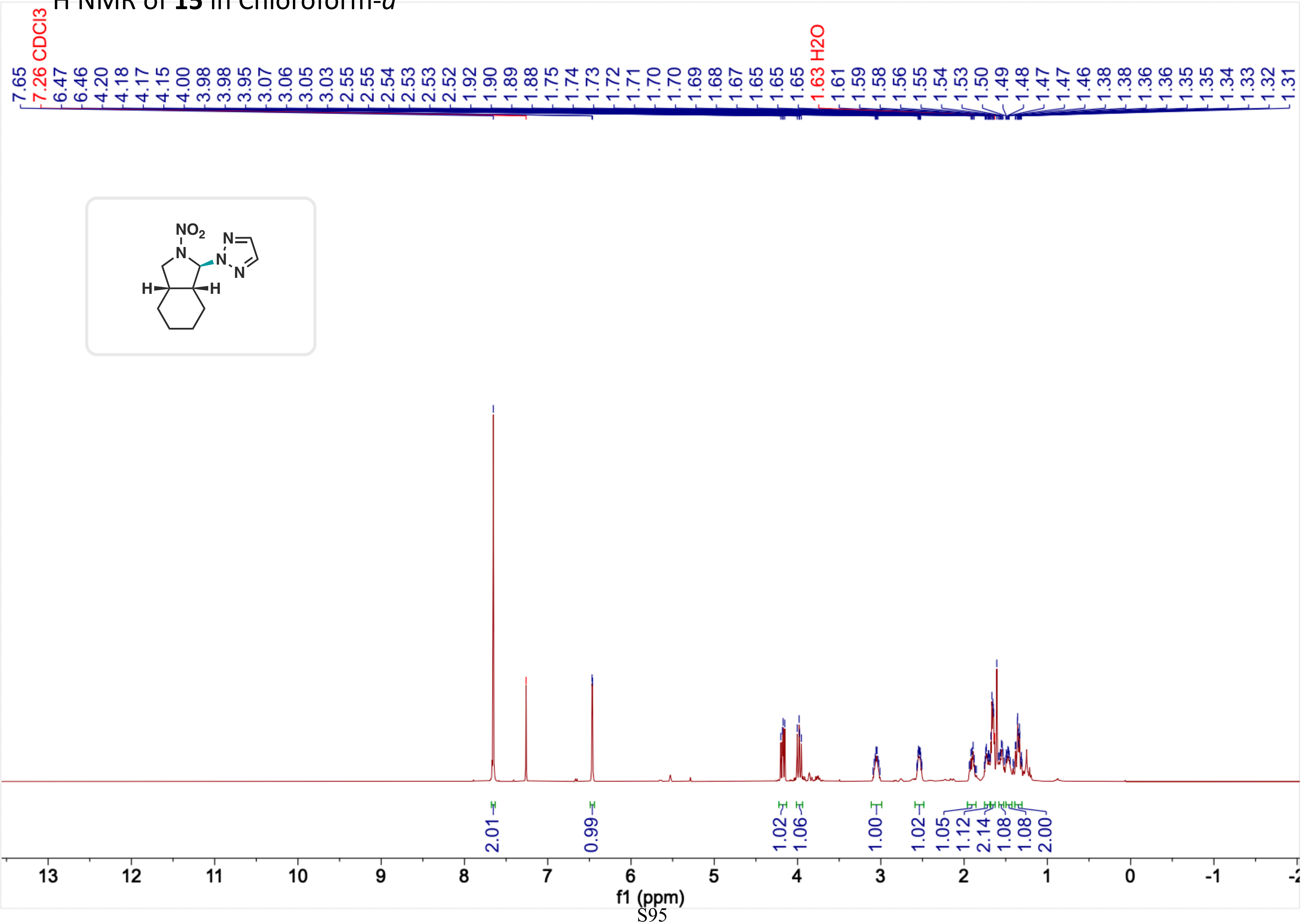

^{13}C NMR of **15** in Chloroform-*d*

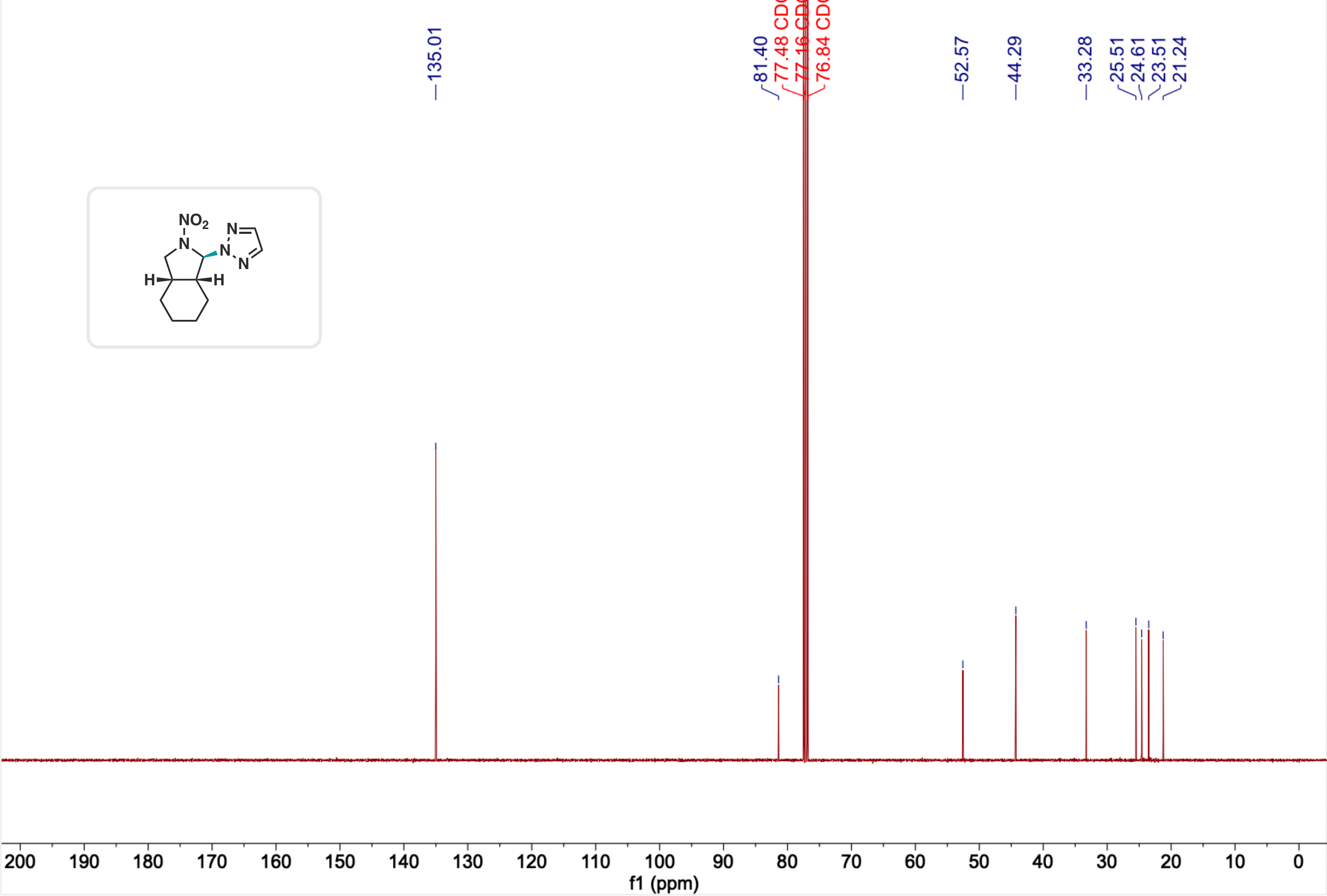

^{1}H NMR of **16** in Chloroform-*d*

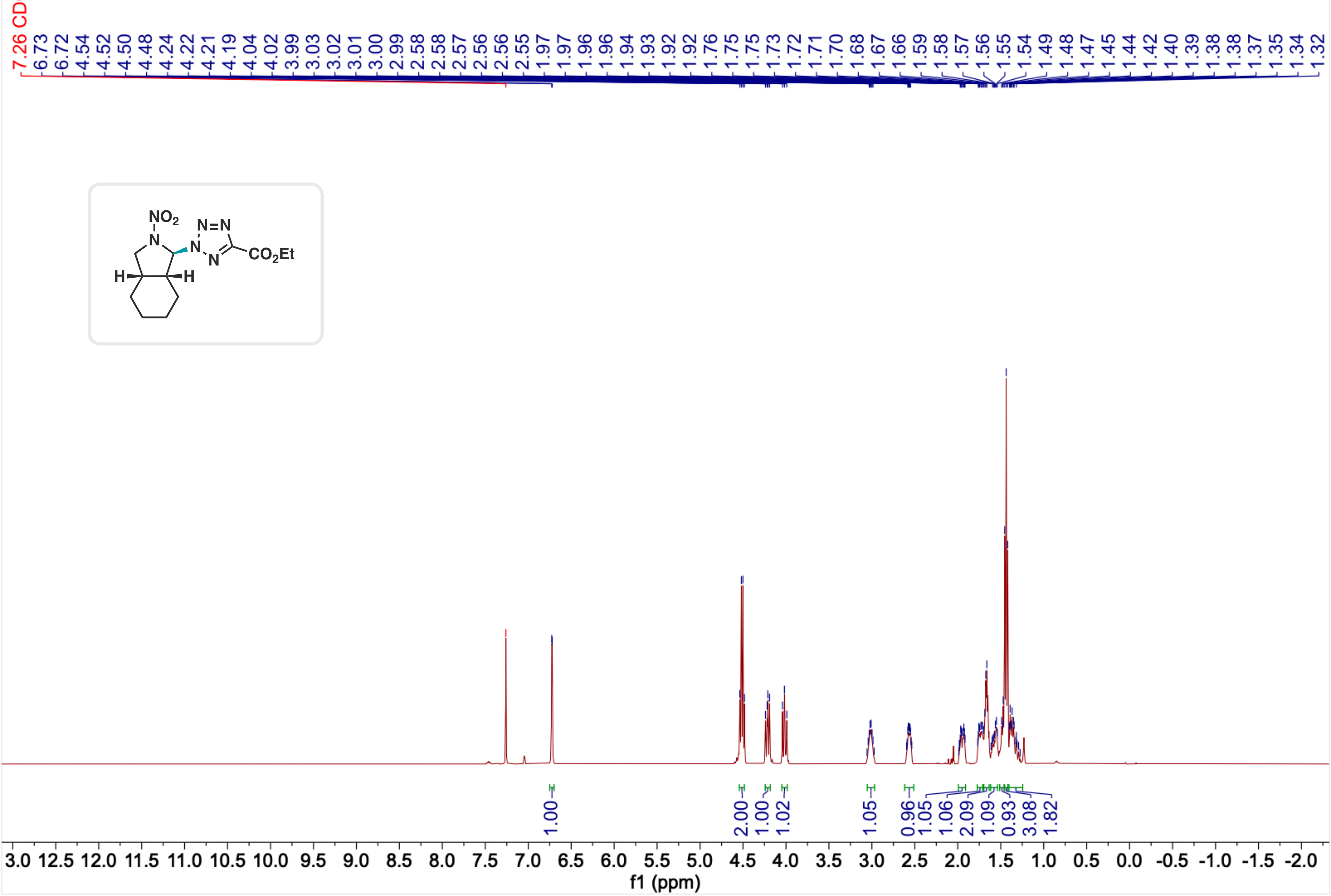

^{13}C NMR of **16** in Chloroform-*d*

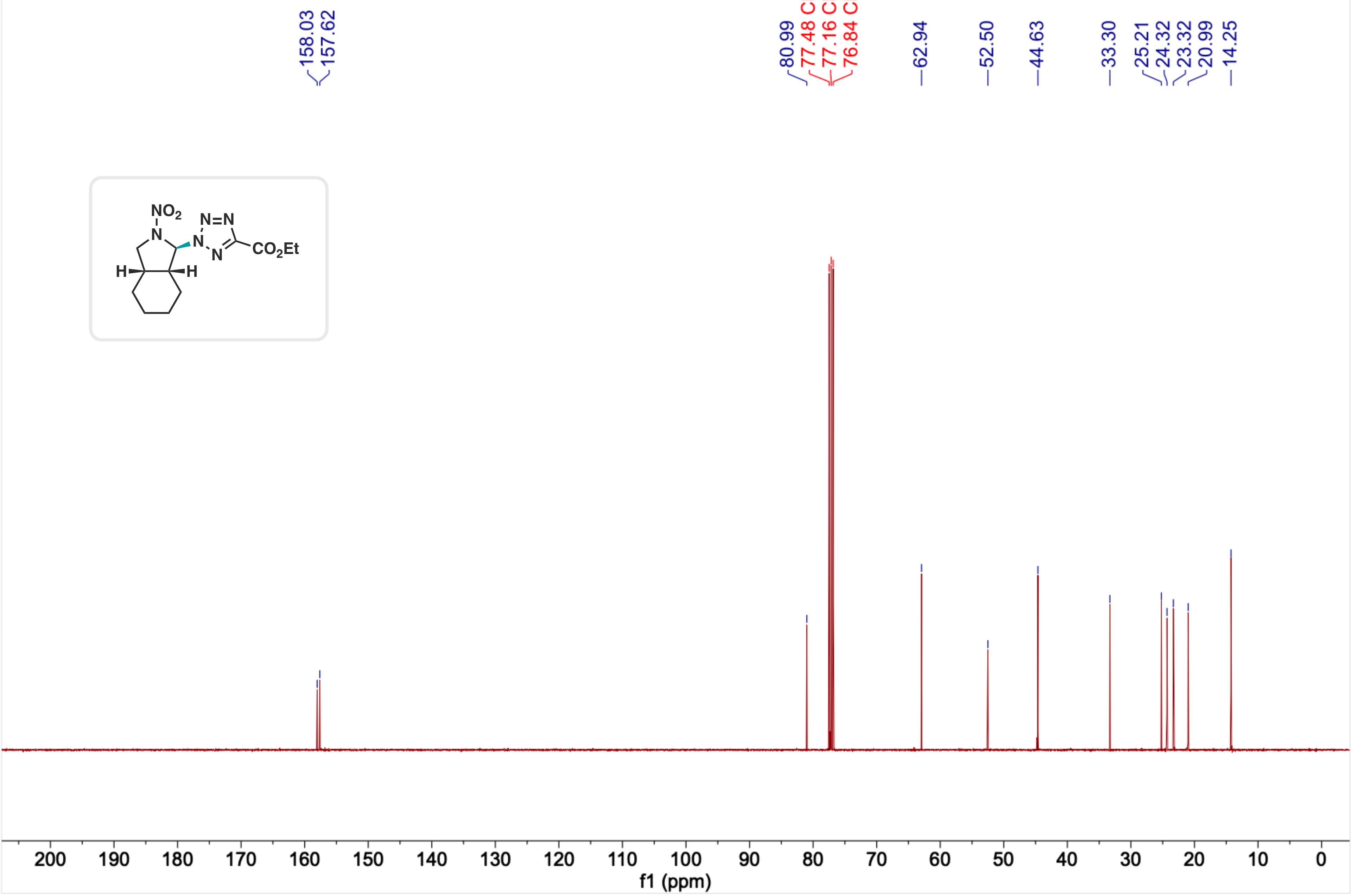

^{1}H-^{15}N HMBC NMR of **16** in Chloroform-*d*

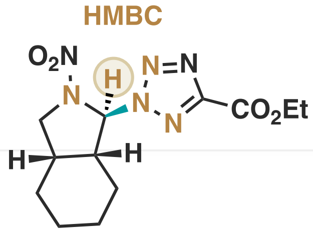

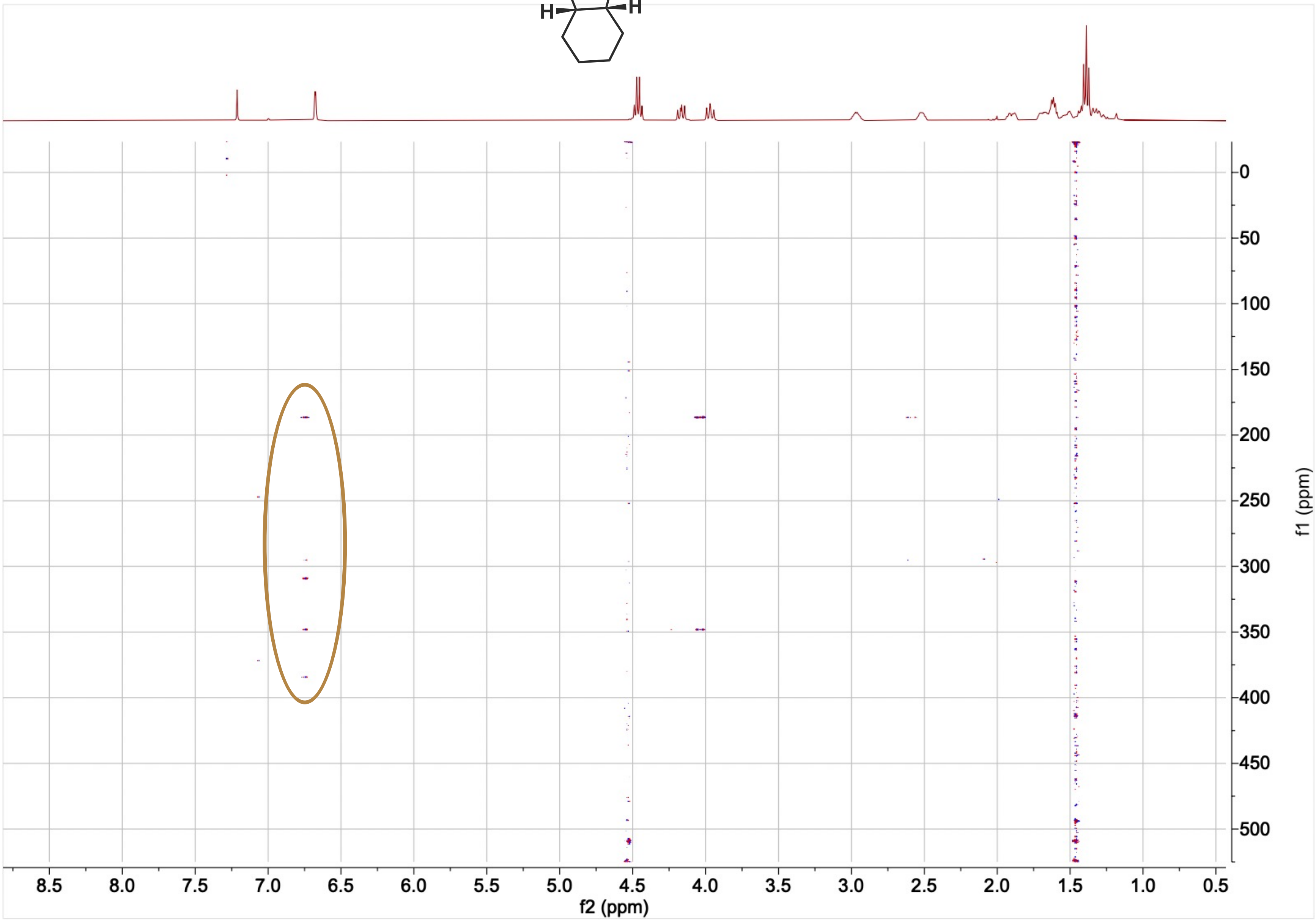

^{1}H NMR of **17** in Chloroform-*d*

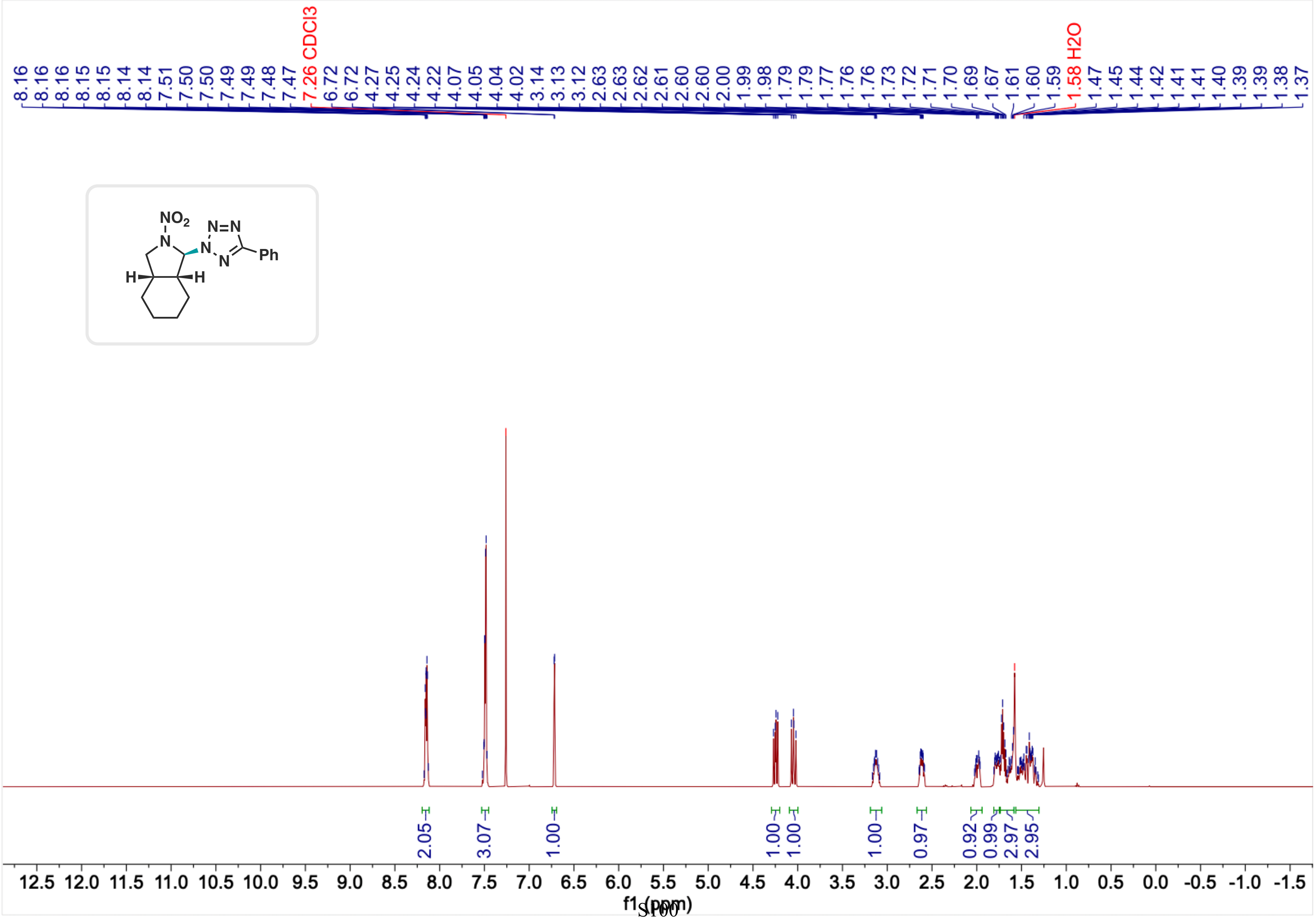

^{13}C NMR of **17** in Chloroform-*d*

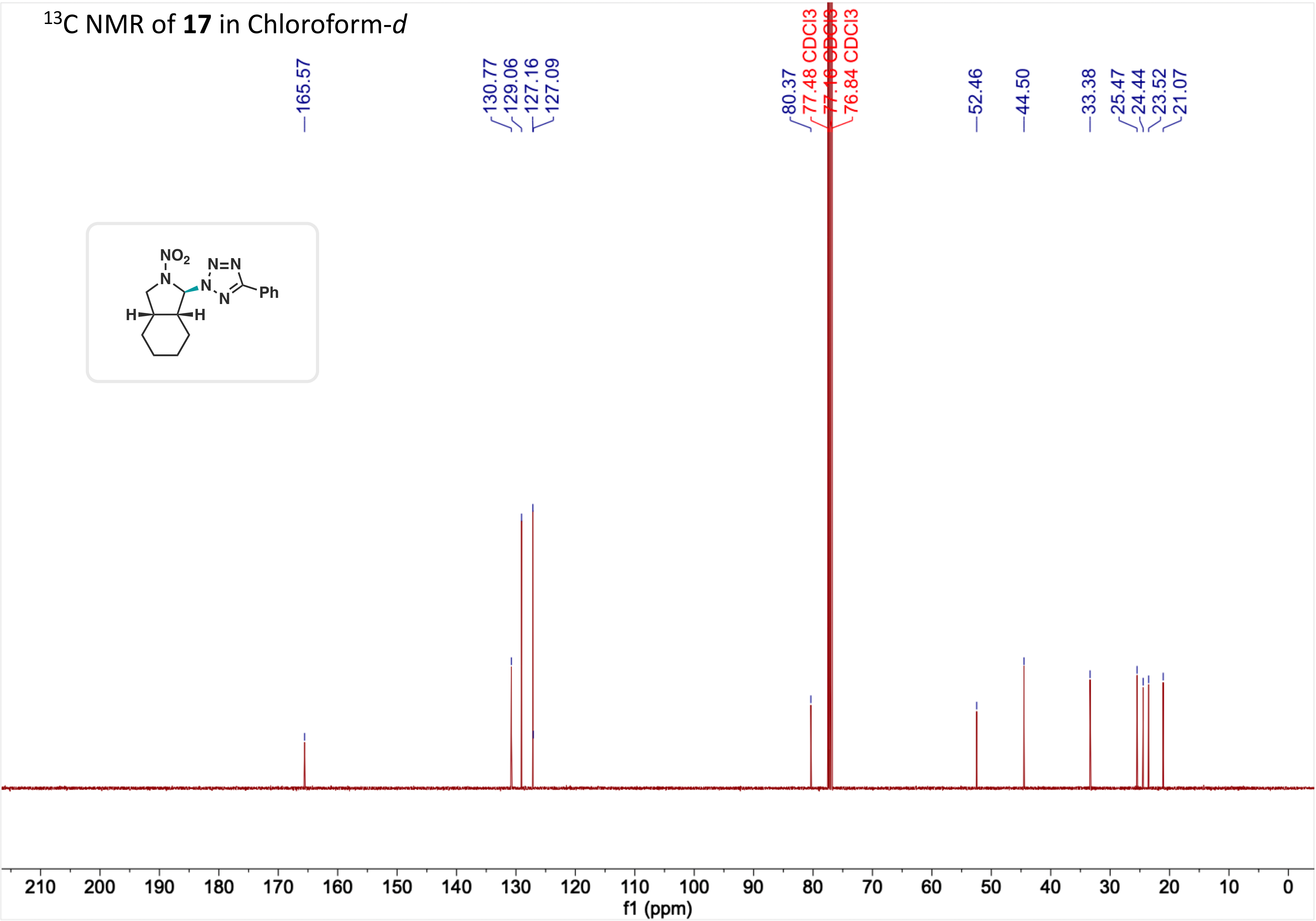

^{1}H NMR of **18** in Chloroform-*d*

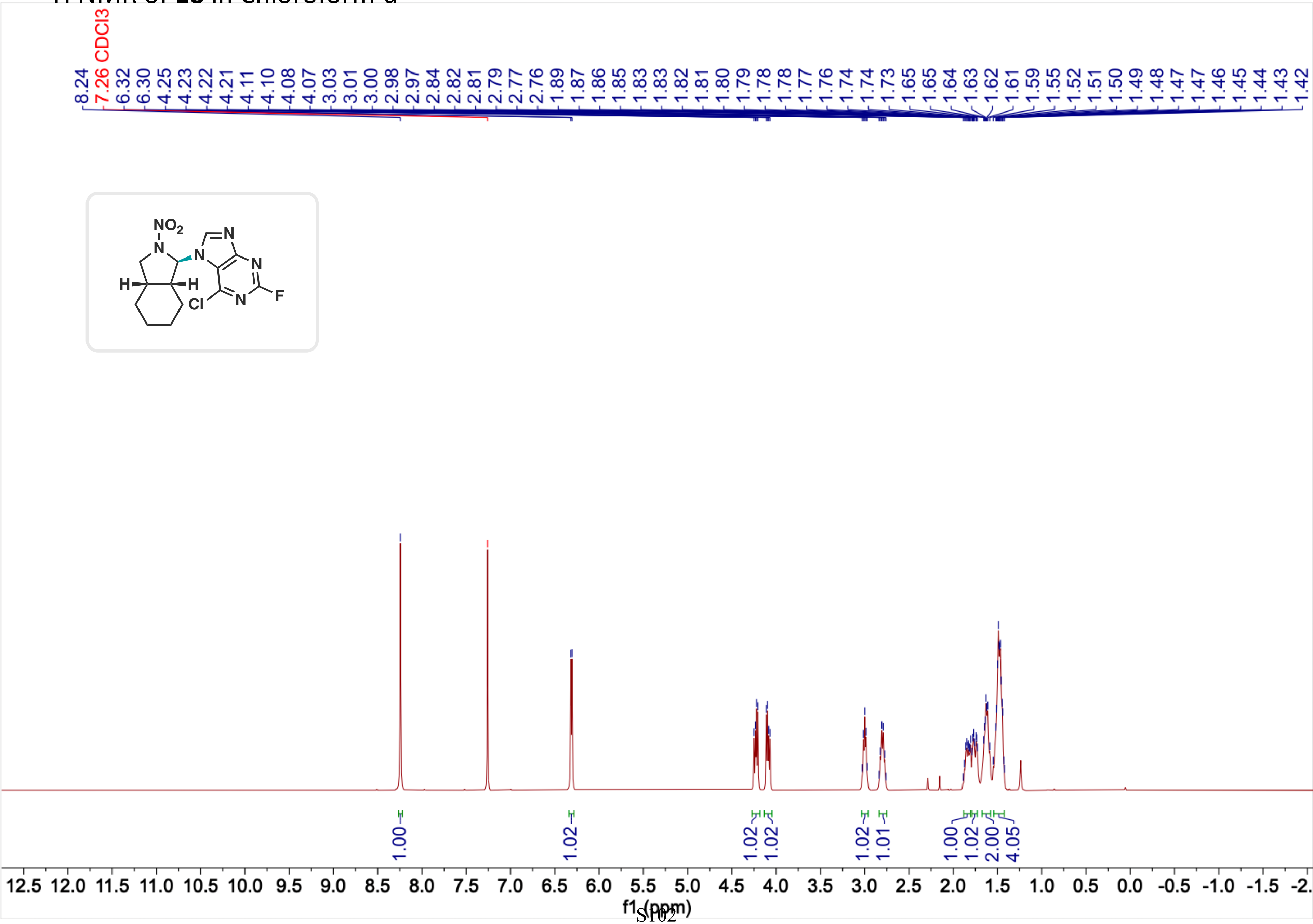

^{19}F NMR of **18** in Chloroform-*d*

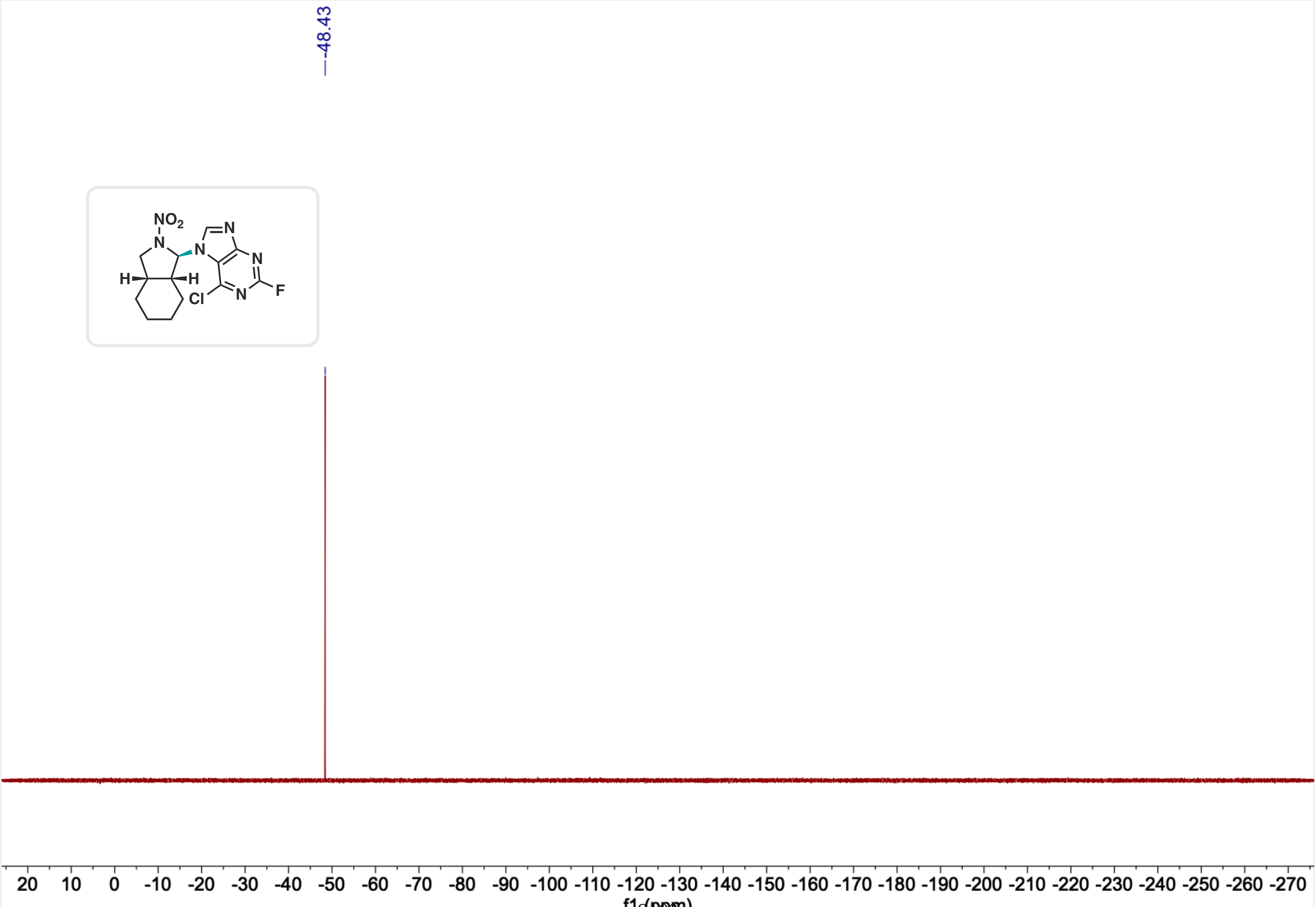

^{13}C NMR of **18** in Chloroform-*d*

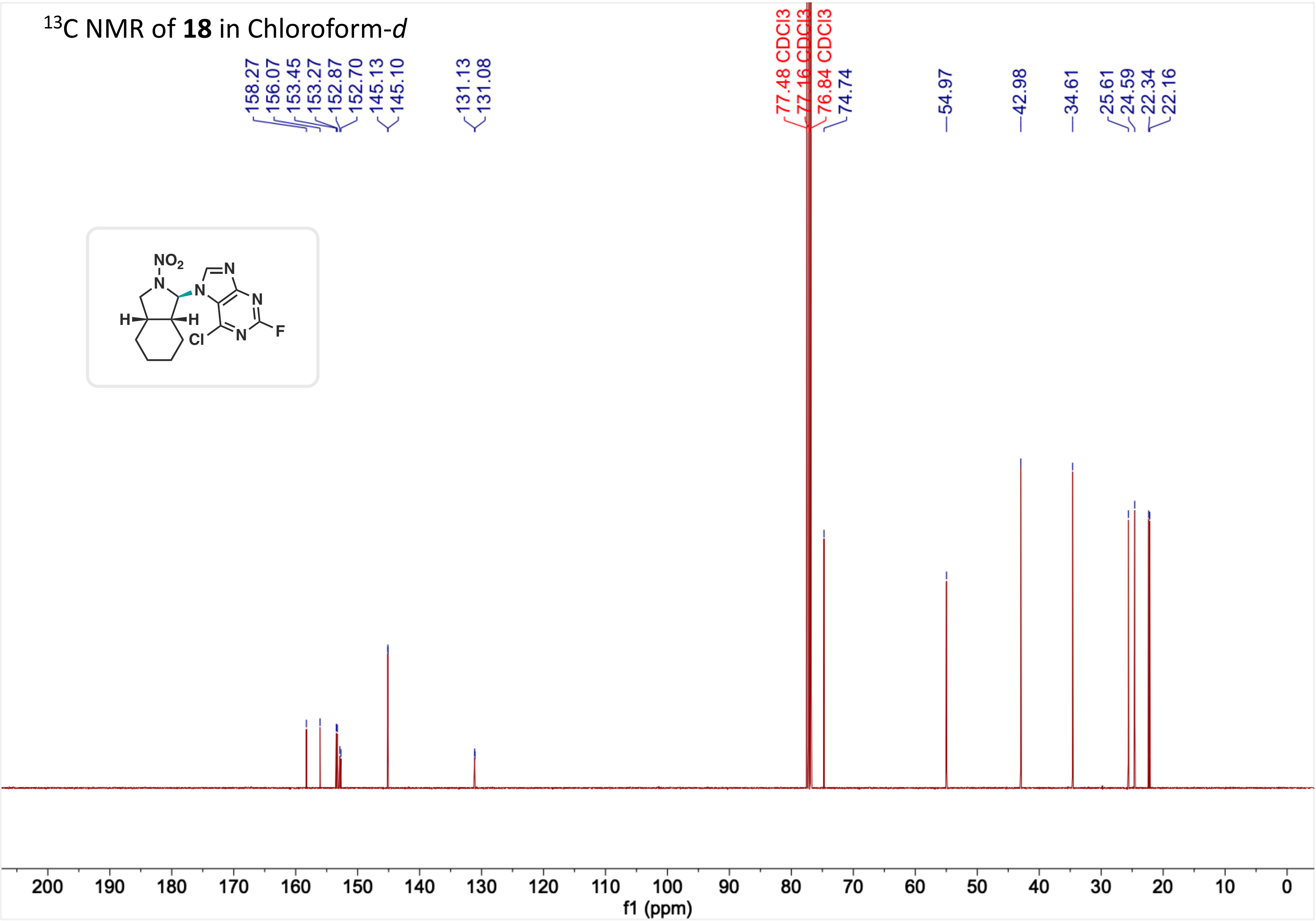

^{1}H NMR of **19** in Chloroform-*d*

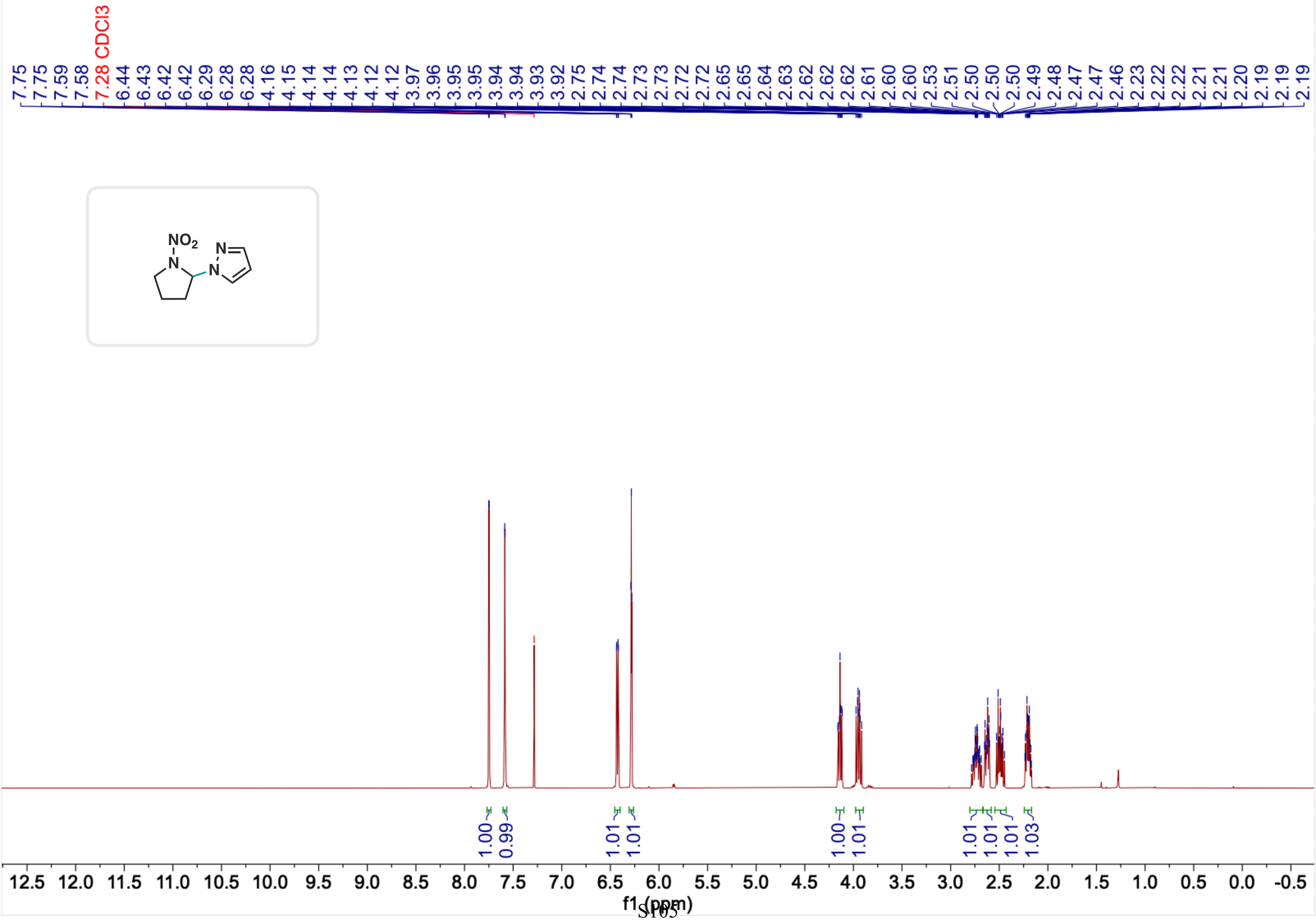

13C NMR of **19** in Chloroform-*d*

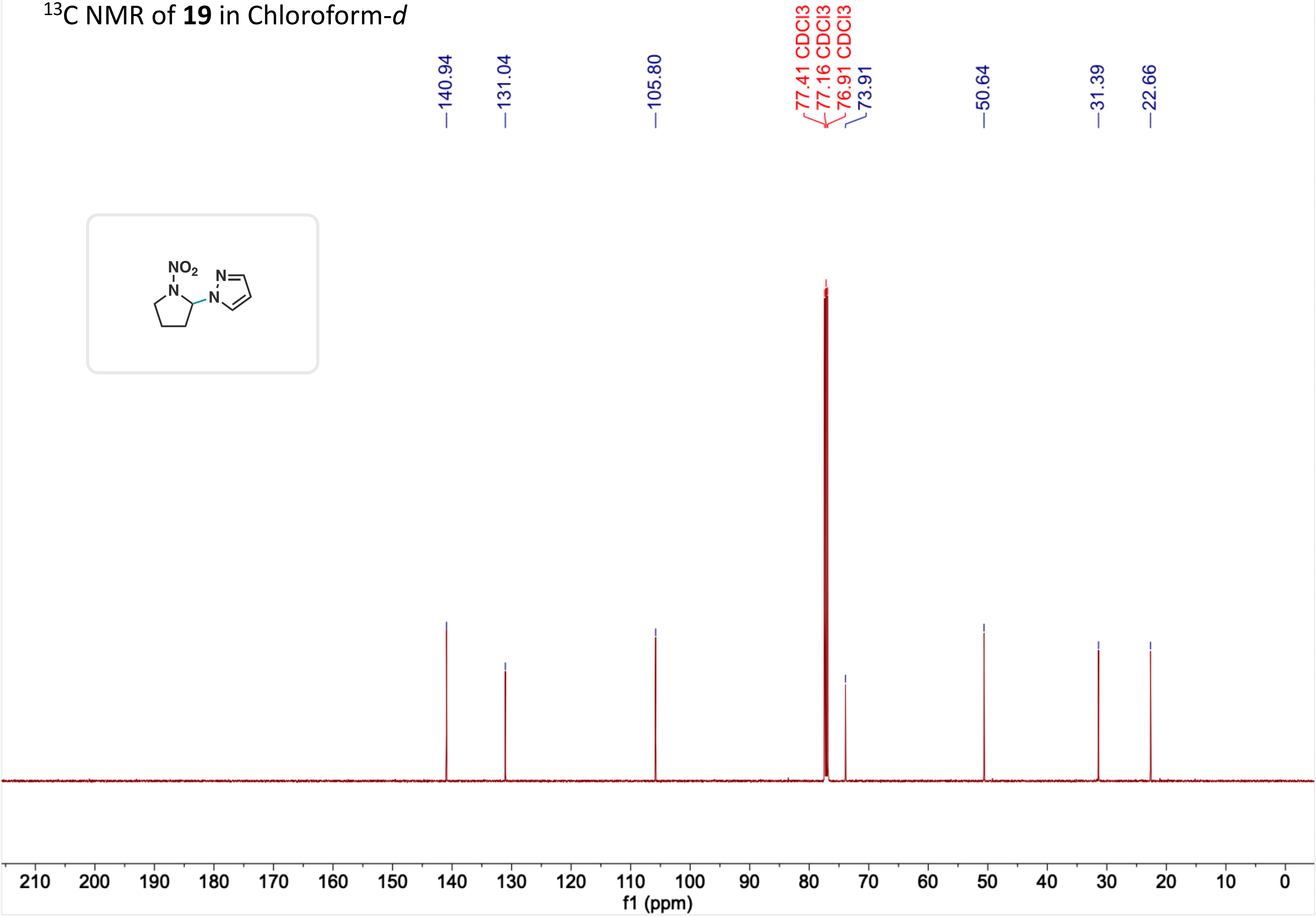

^{1}H NMR of **20** in Chloroform-*d*

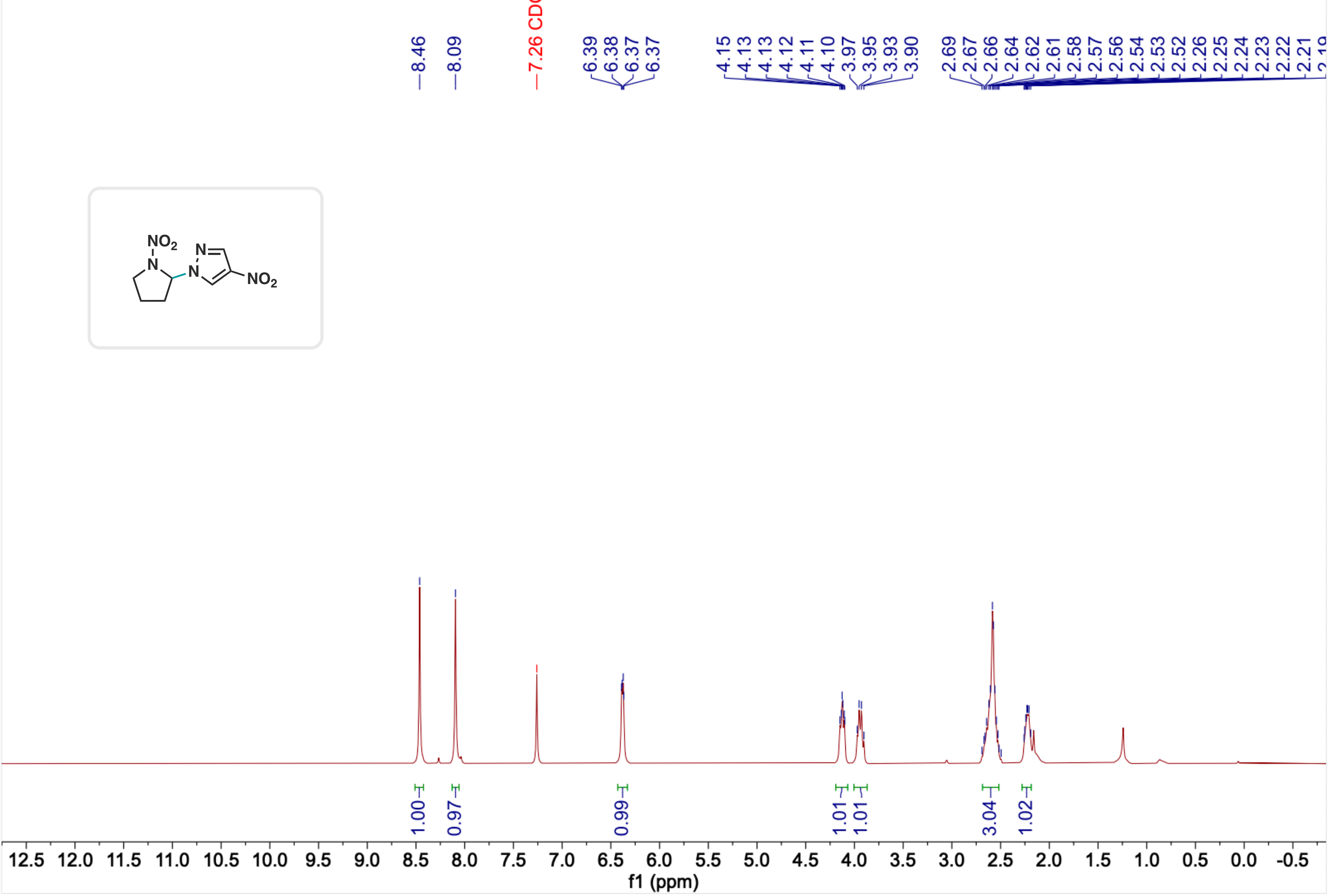

^{13}C NMR of **20** in Chloroform-*d*

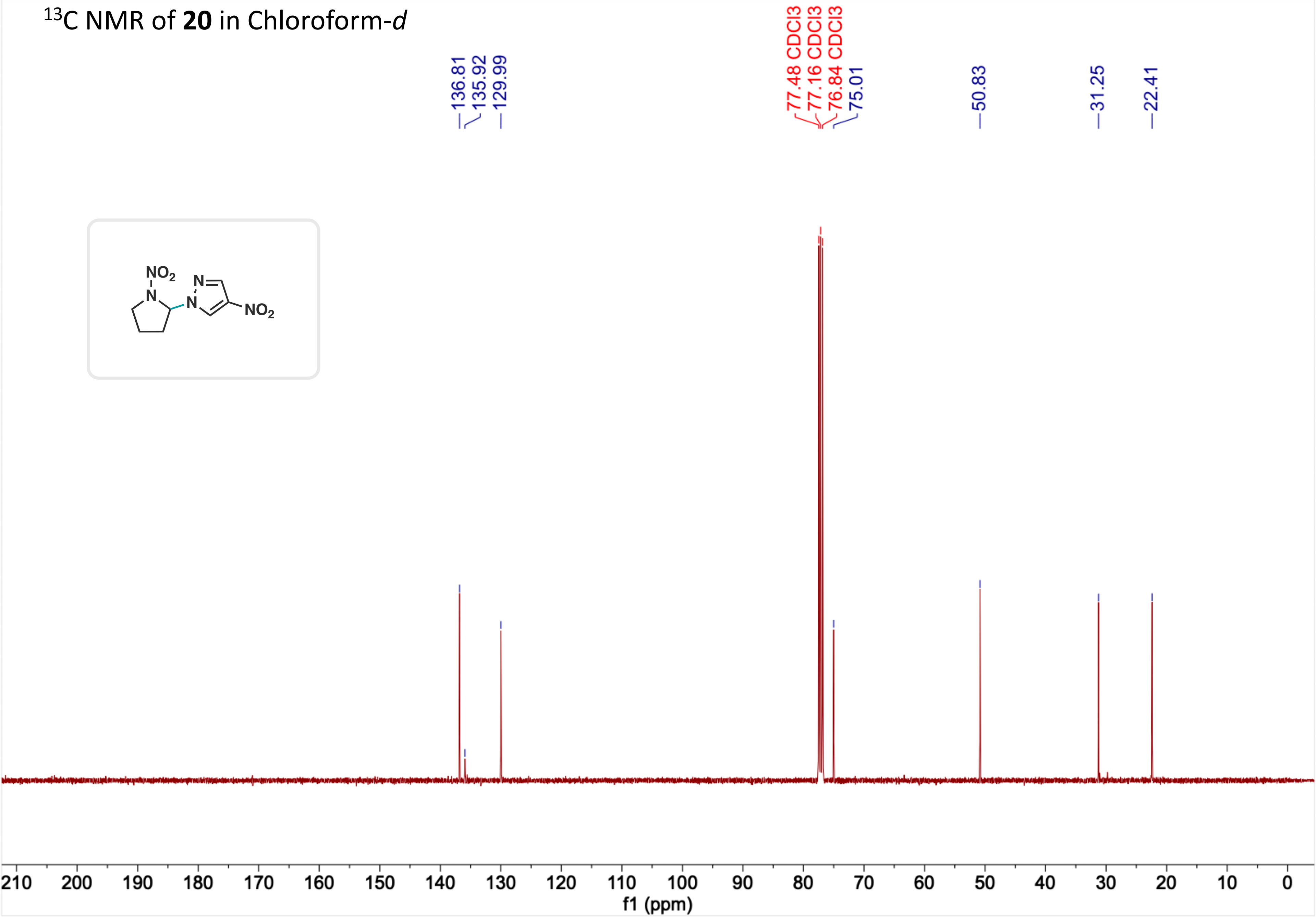

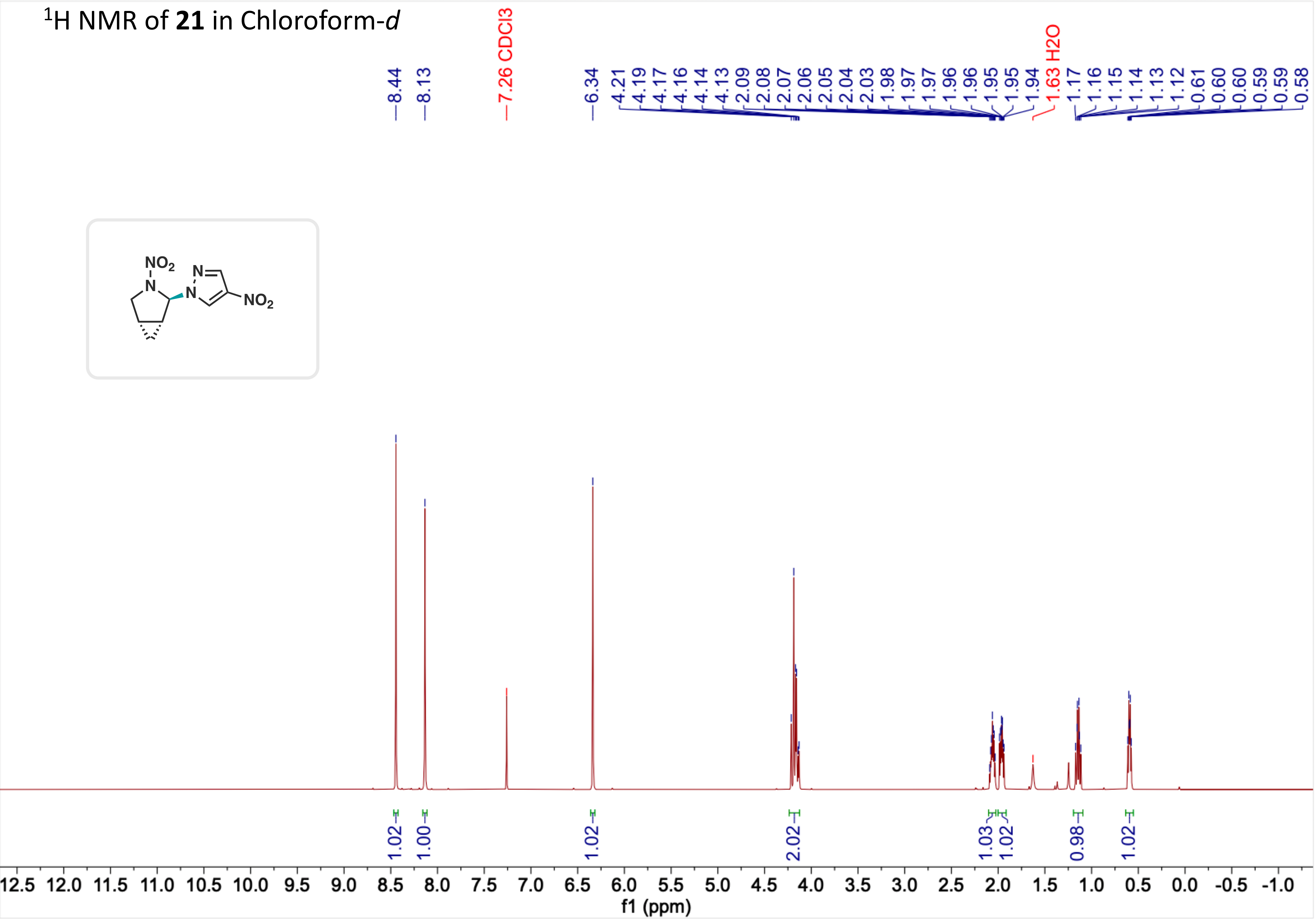

^{1}H NMR of **21** in Chloroform-*d*
8.44
8.13
7.26 CDCl3
6.34
4.21
4.19
4.17
4.16
4.14
4.13
2.09
2.08
2.07
2.06
2.05
2.04
2.03
1.98
1.97
1.97
1.96
1.96
1.95
1.95
1.94
1.63 H2O
1.17
1.16
1.15
1.14
1.13
1.12
0.61
0.60
0.60
0.59
0.59
0.58
NO2
N
N
N
NO2
1.02
1.00
1.02
2.02
1.03
1.02
0.98
1.02
12.5 12.0 11.5 11.0 10.5 10.0 9.5 9.0 8.5 8.0 7.5 7.0 6.5 6.0 5.5 5.0 4.5 4.0 3.5 3.0 2.5 2.0 1.5 1.0 0.5 0.0 -0.5 -1.0
f1 (ppm)

^{13}C NMR of **21** in Chloroform-*d*

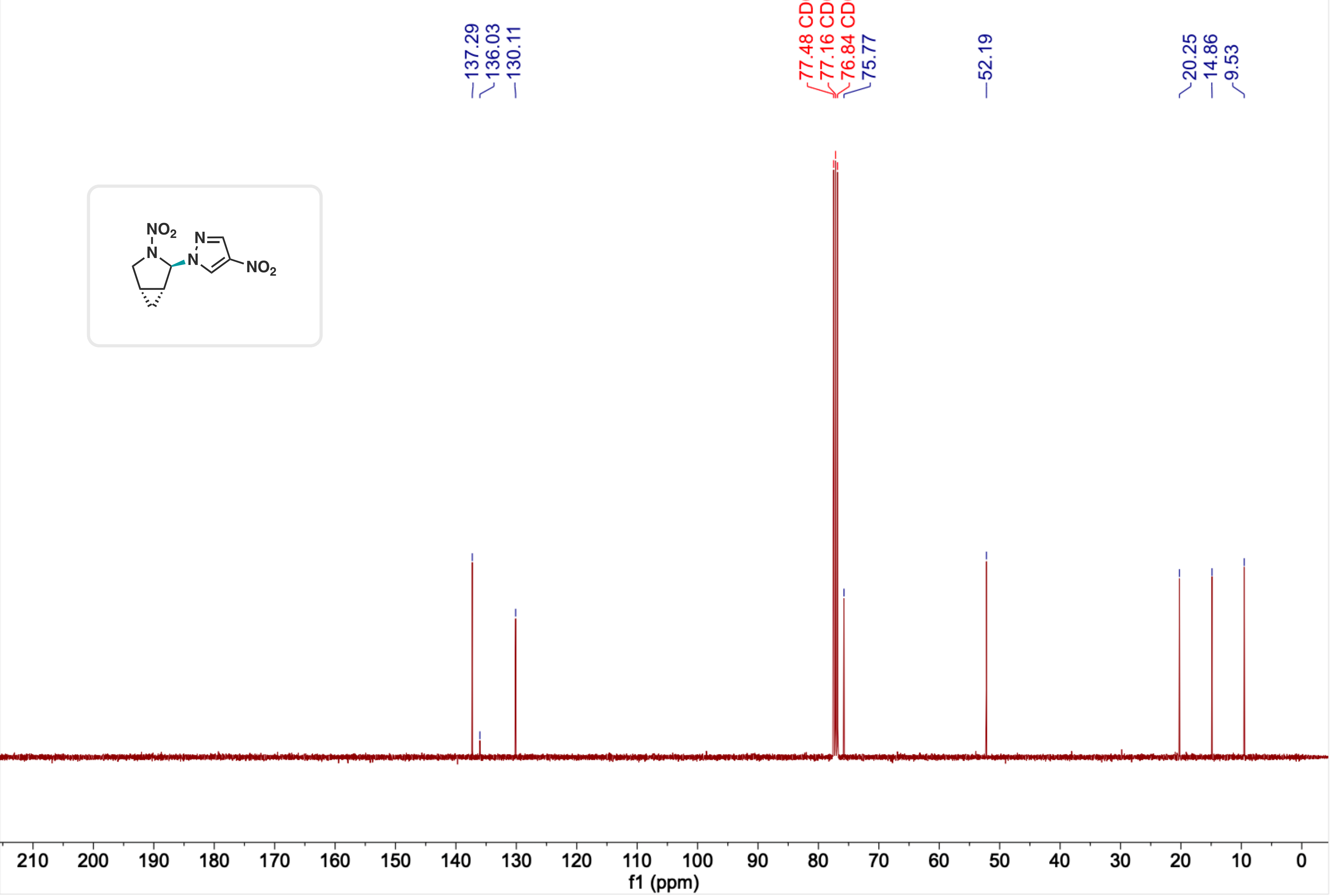

^{1}H NMR of **22** in Acetone-d_6

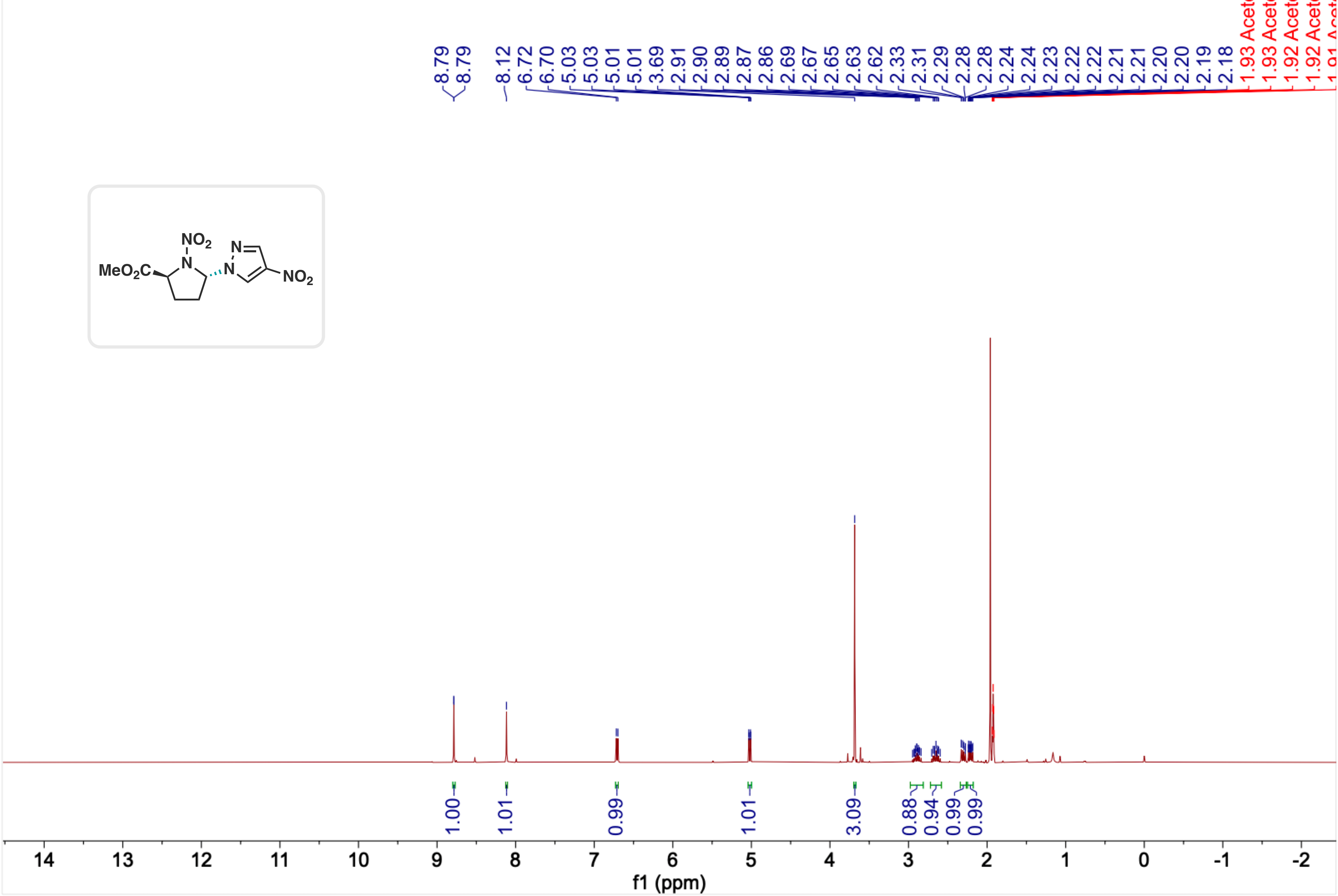

^{13}C NMR of **22** in Acetone-d_6

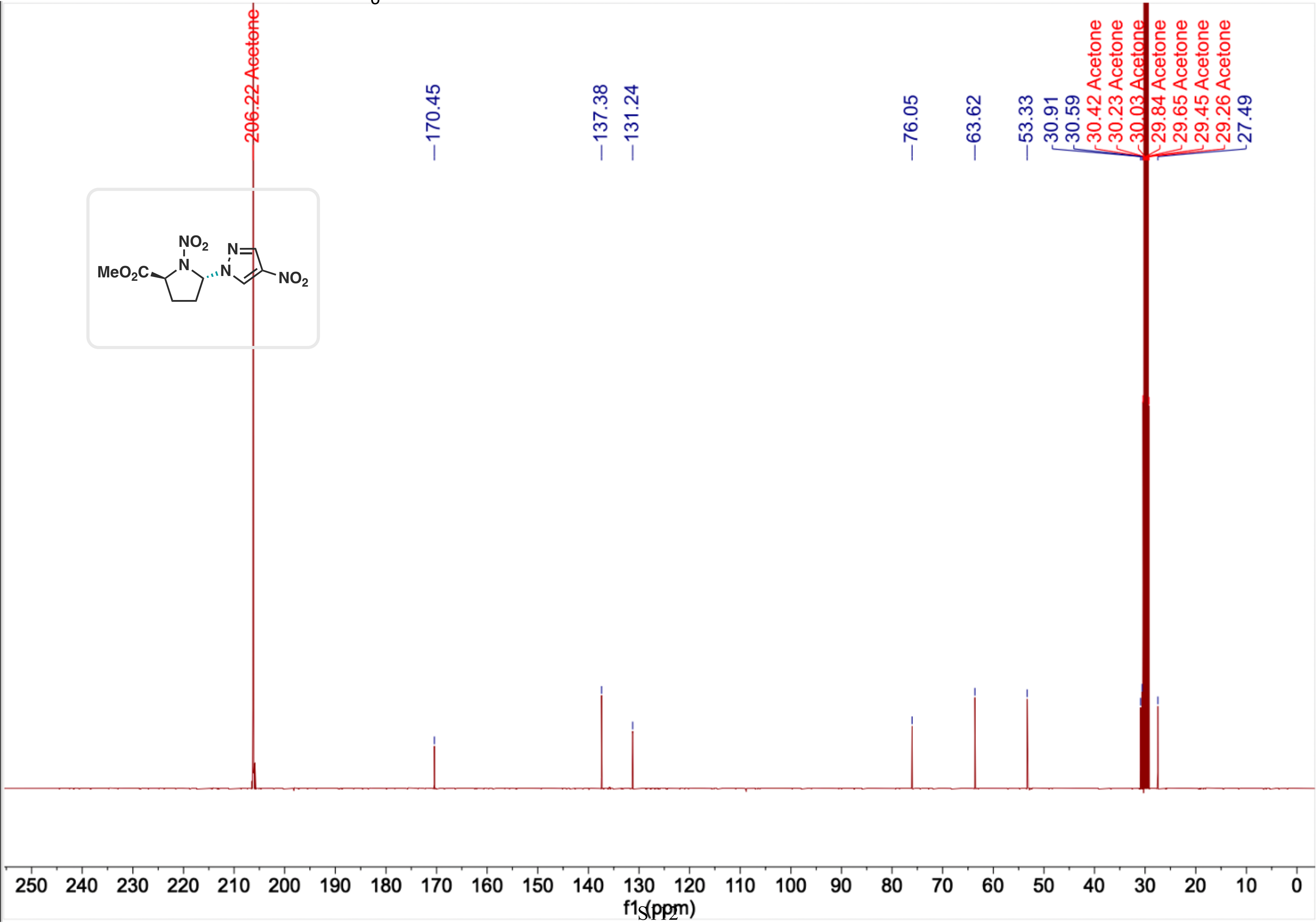

^{1}H NMR of **23** in Chloroform-*d*

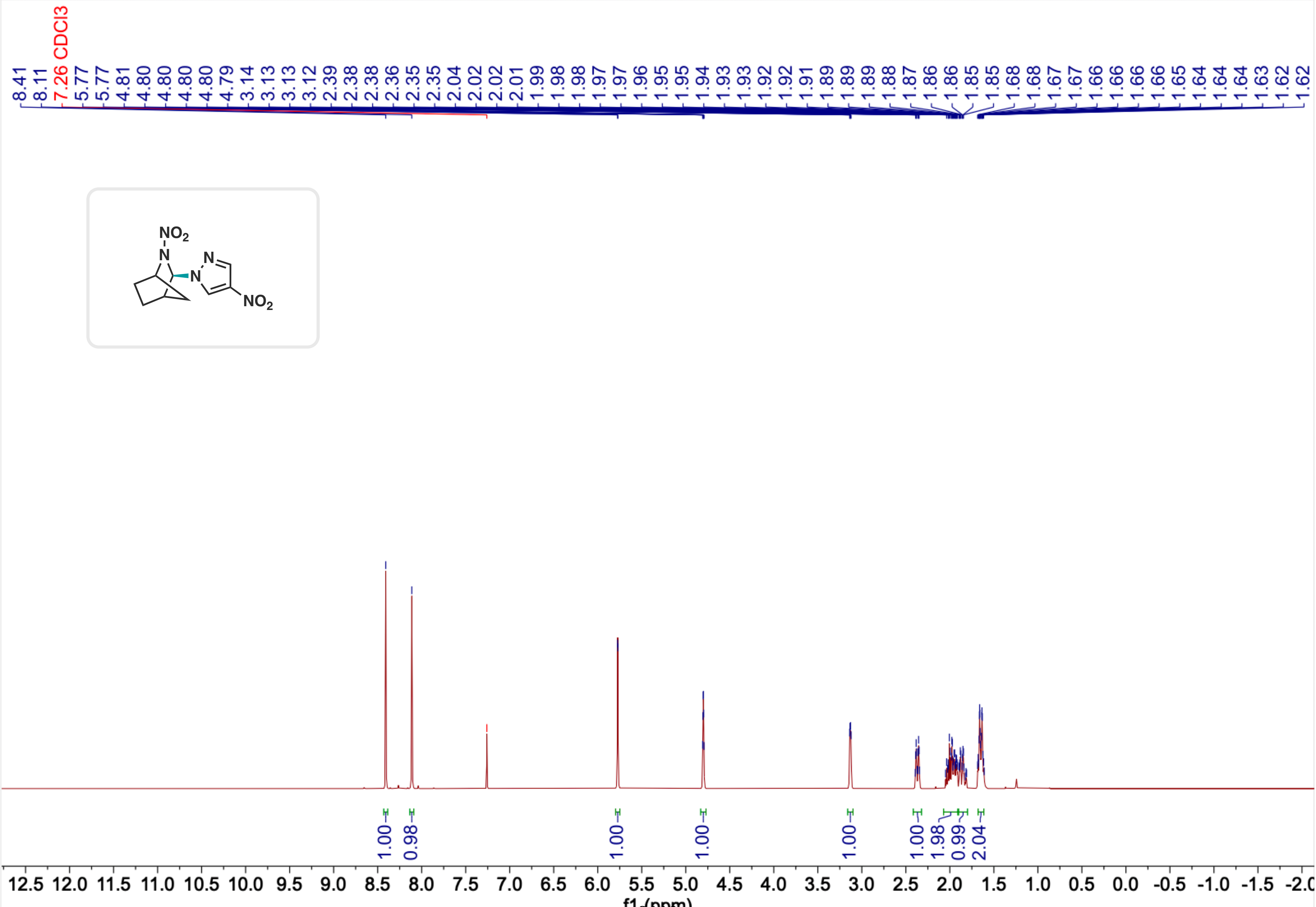

^{13}C NMR of **23** in Chloroform-*d*

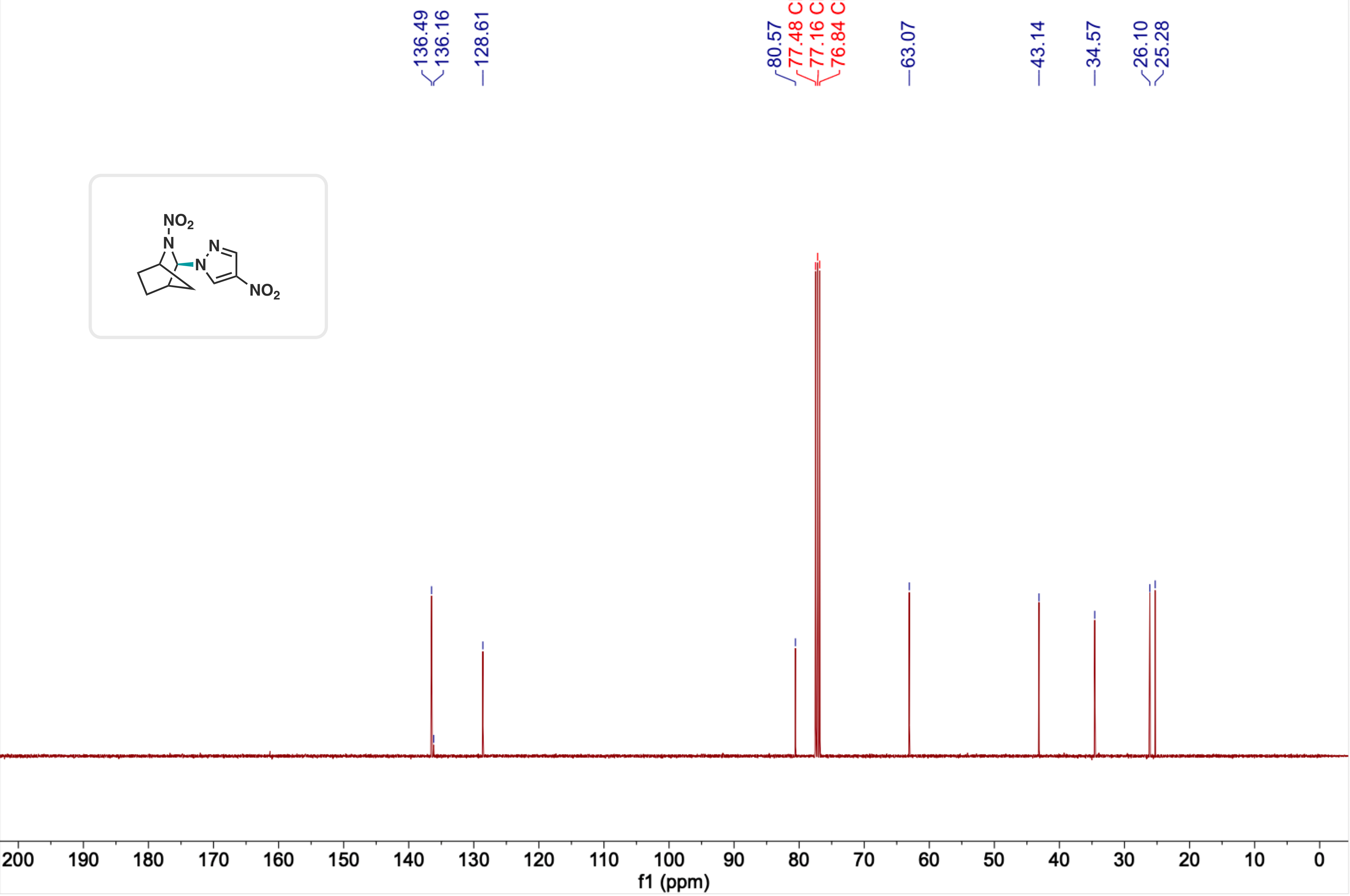

^{1}H NMR of **24** in Chloroform-*d*

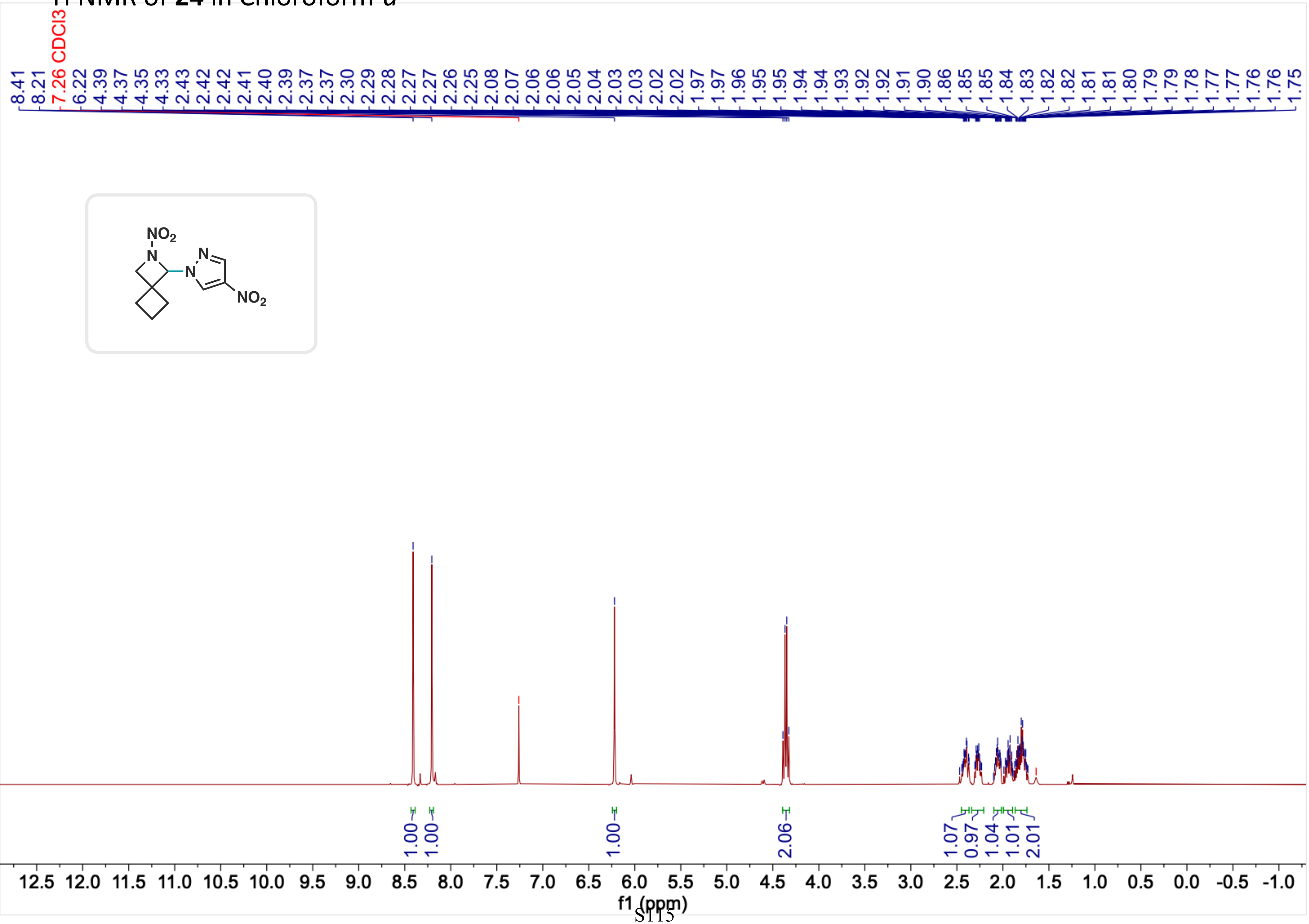

^{13}C NMR of **24** in Chloroform-*d*

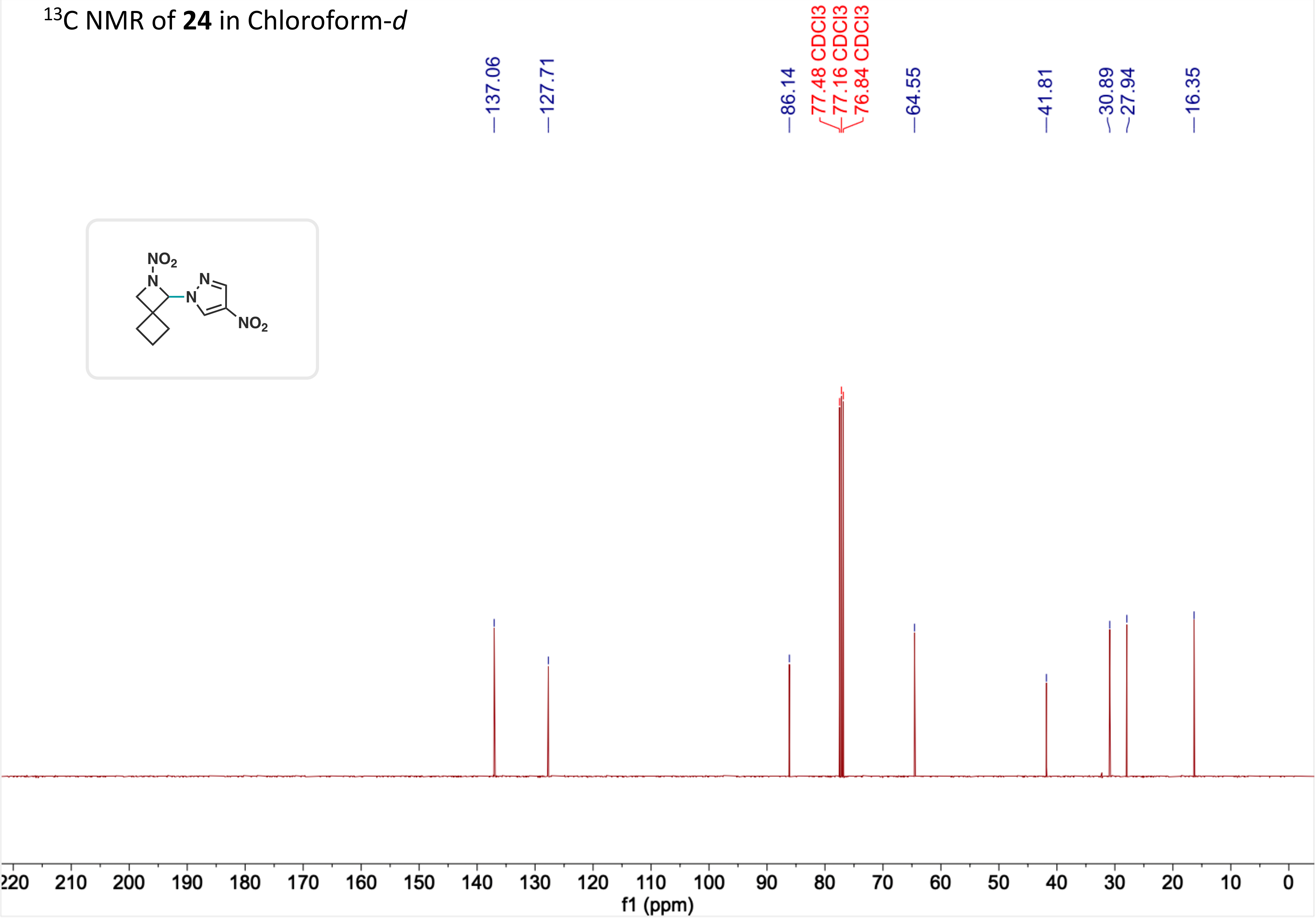

^{1}H NMR of **25** in Chloroform-*d*

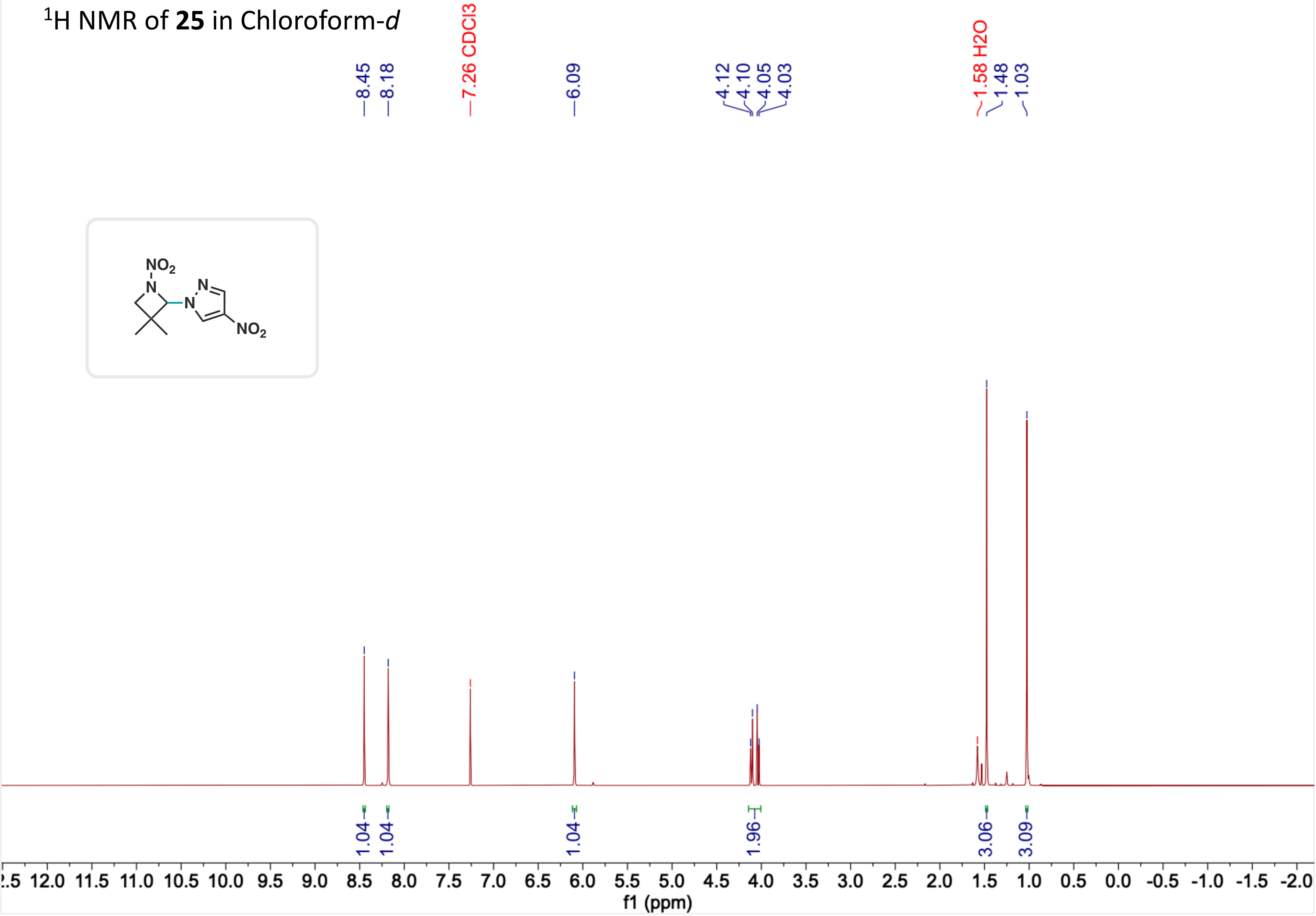

^{13}C NMR of **25** in Chloroform-*d*

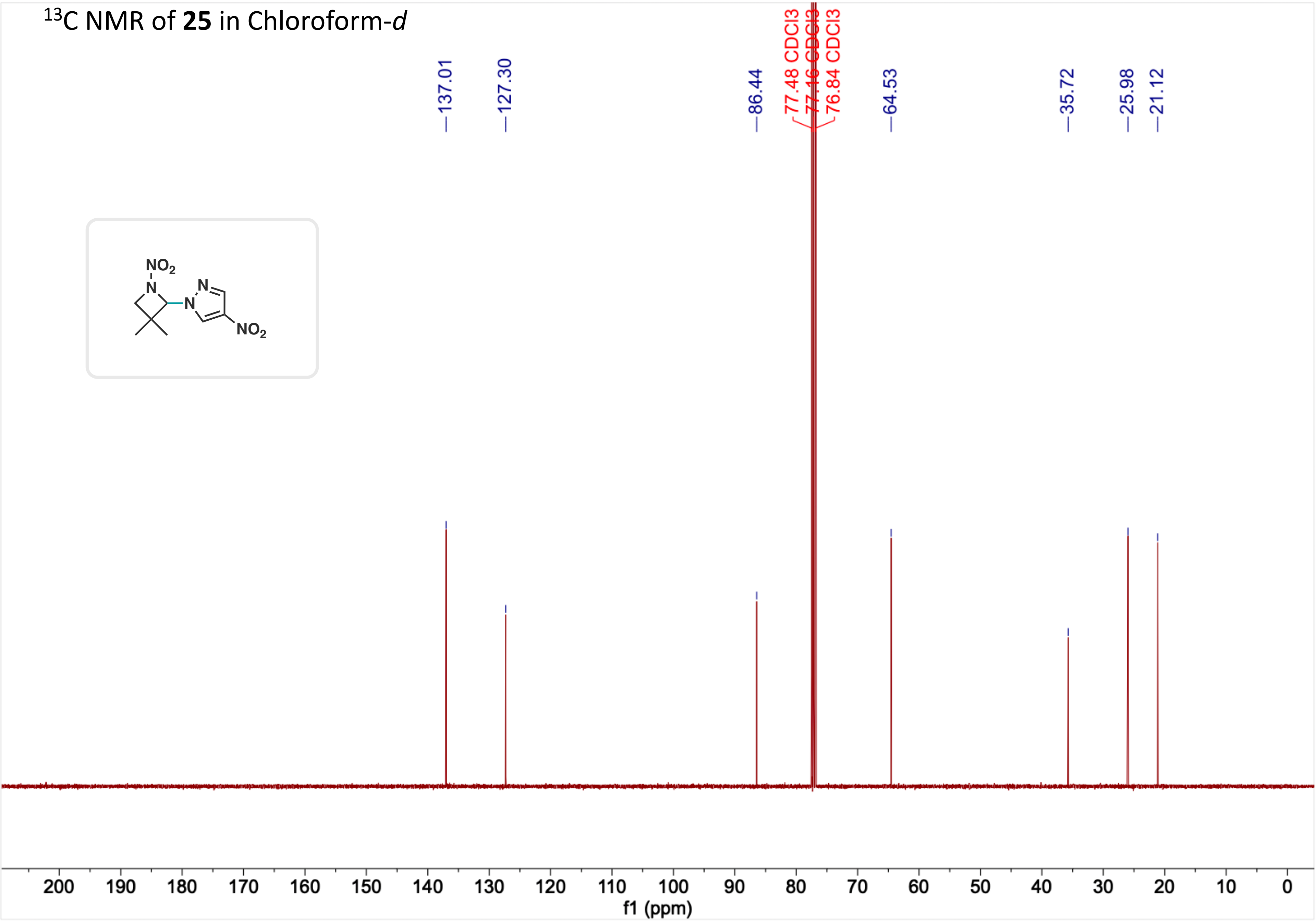

^{1}H NMR of **26** in Acetone-d_6

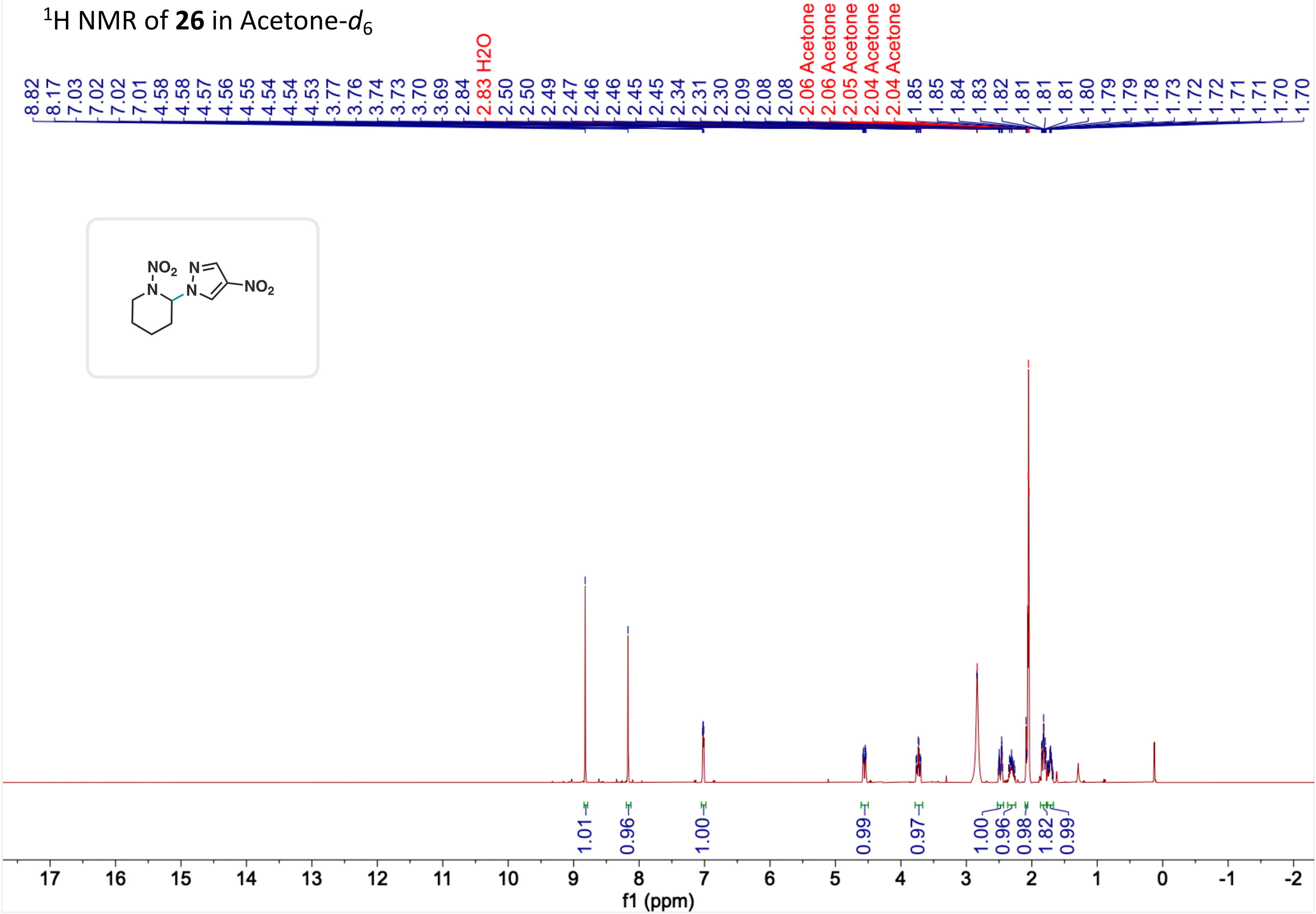

^{13}C NMR of **26** in Acetone-d_6

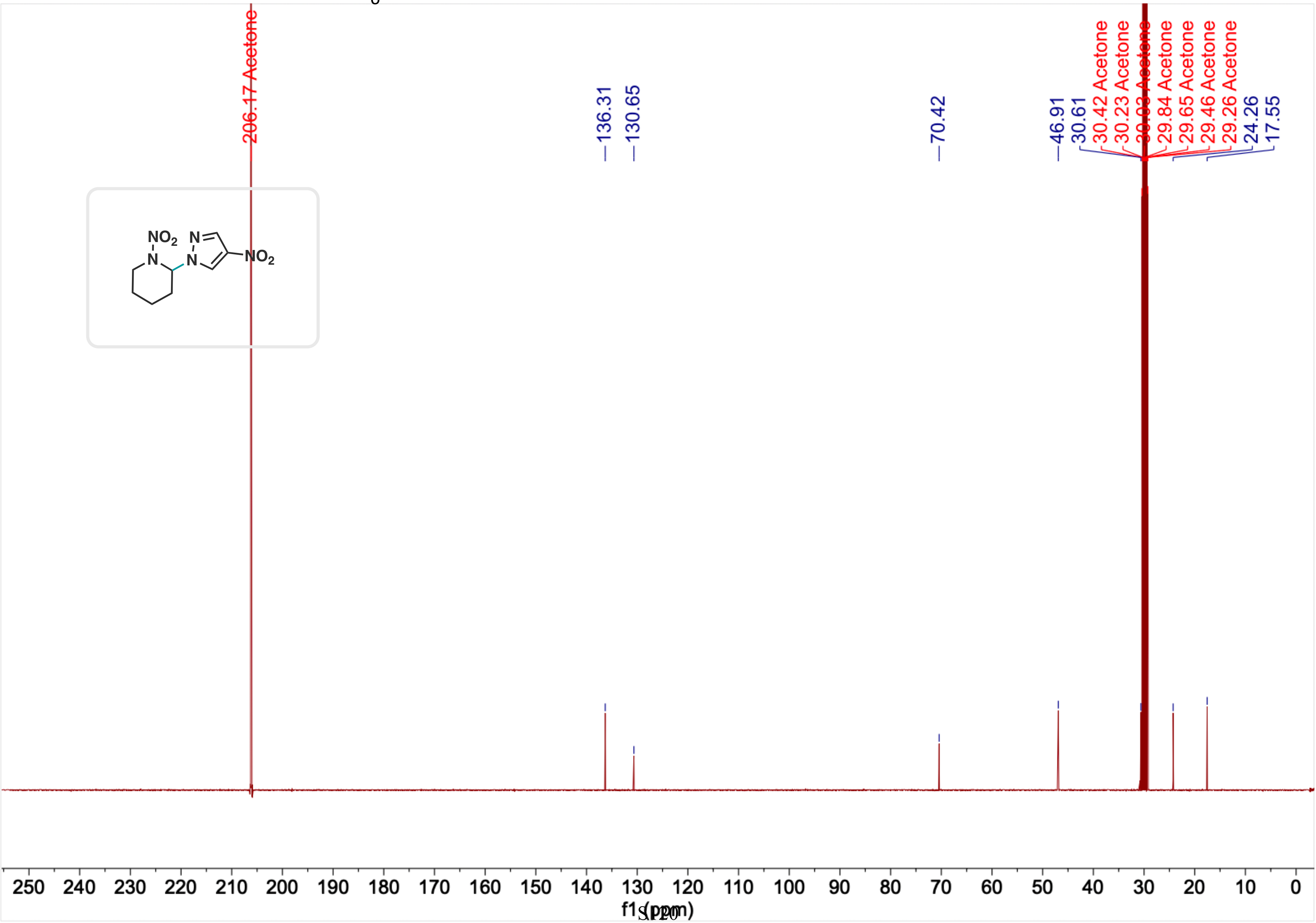

^{1}H NMR of **27** in Chloroform-*d*

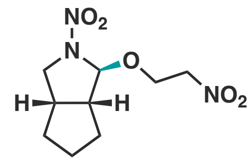

Another set belongs to diastereomer

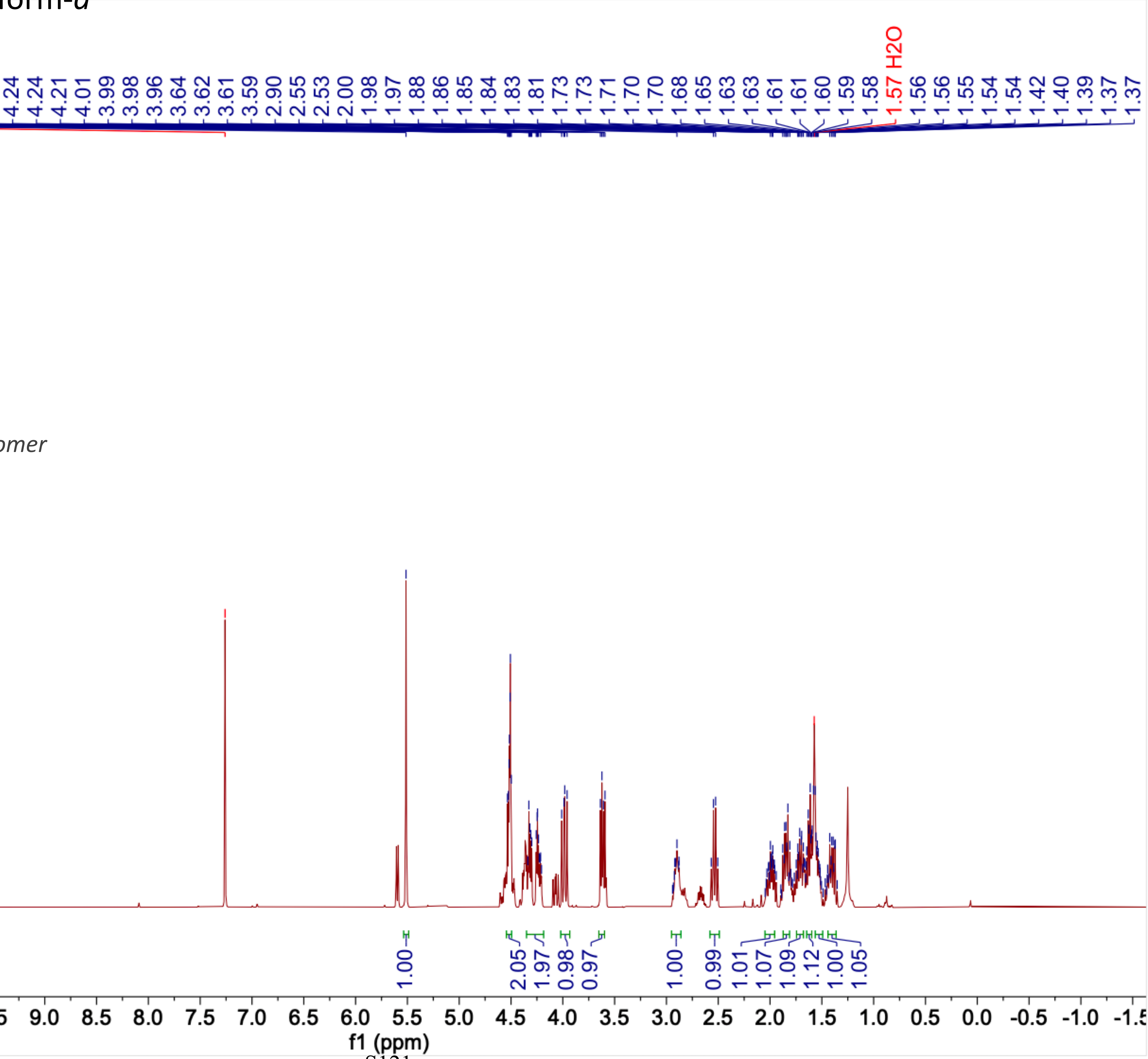

^{13}C NMR of **27** in Chloroform-*d*

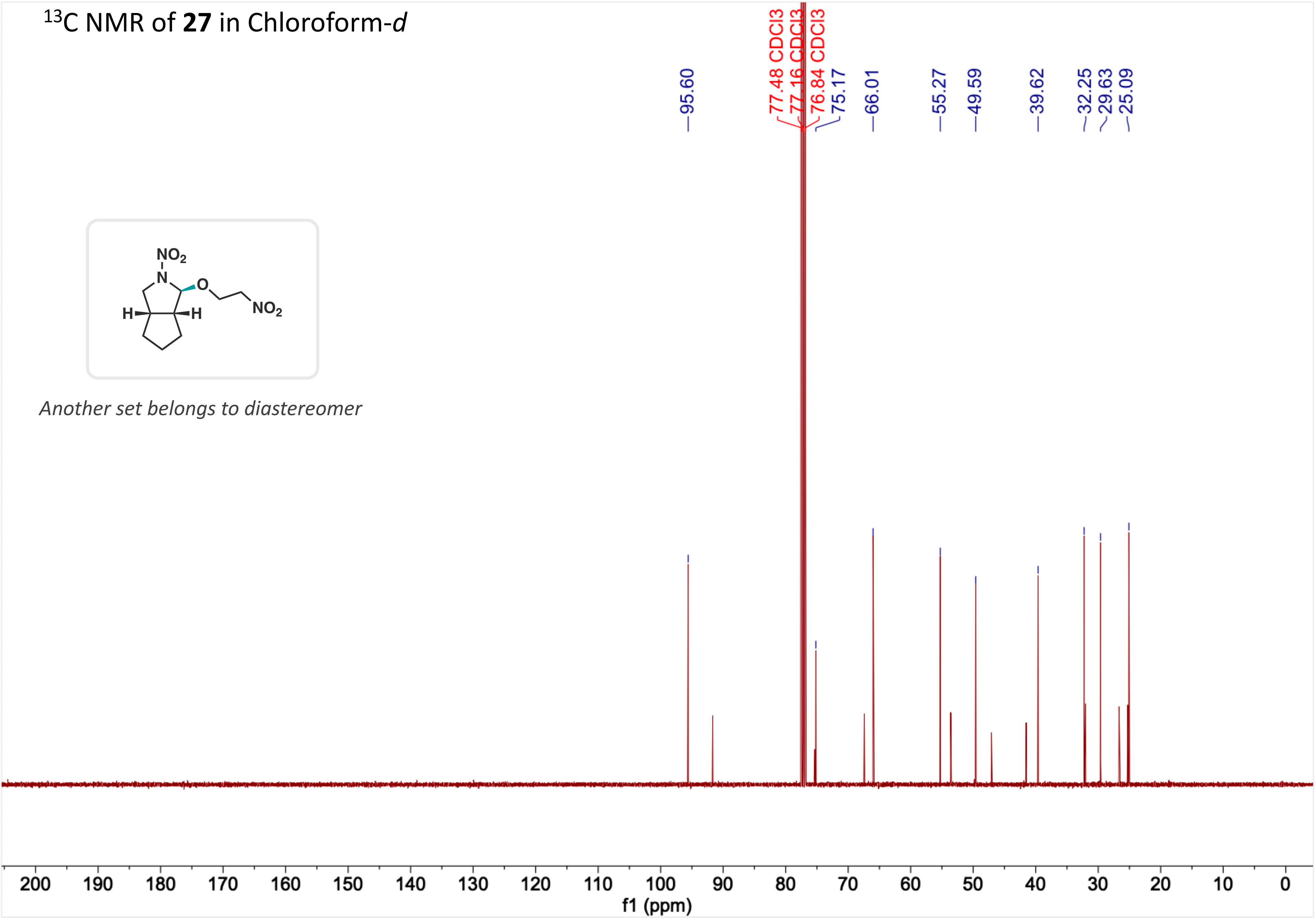

Another set belongs to diastereomer

^{1}H NMR of **28** in Chloroform-*d*

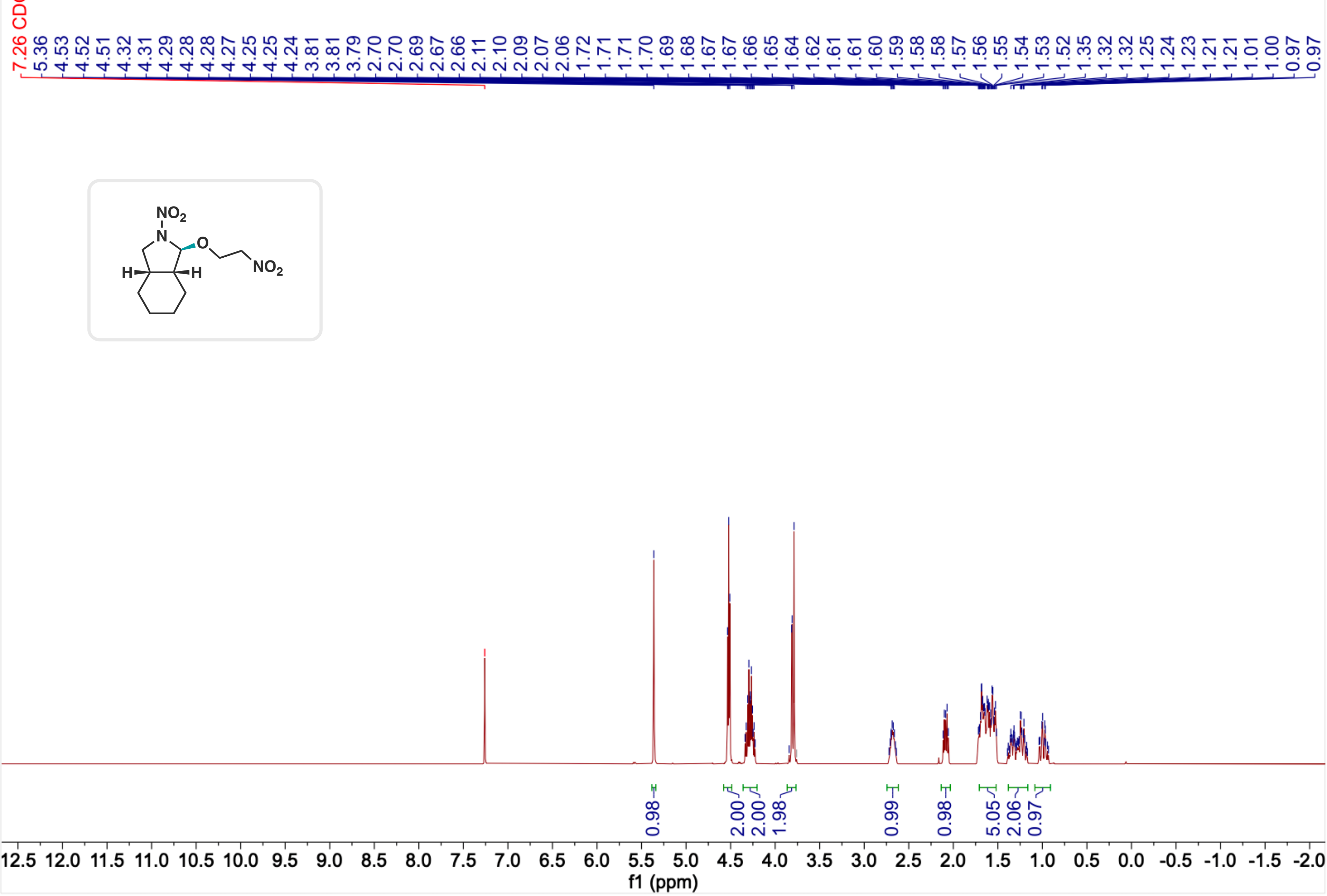

^{13}C NMR of **28** in Chloroform-*d*

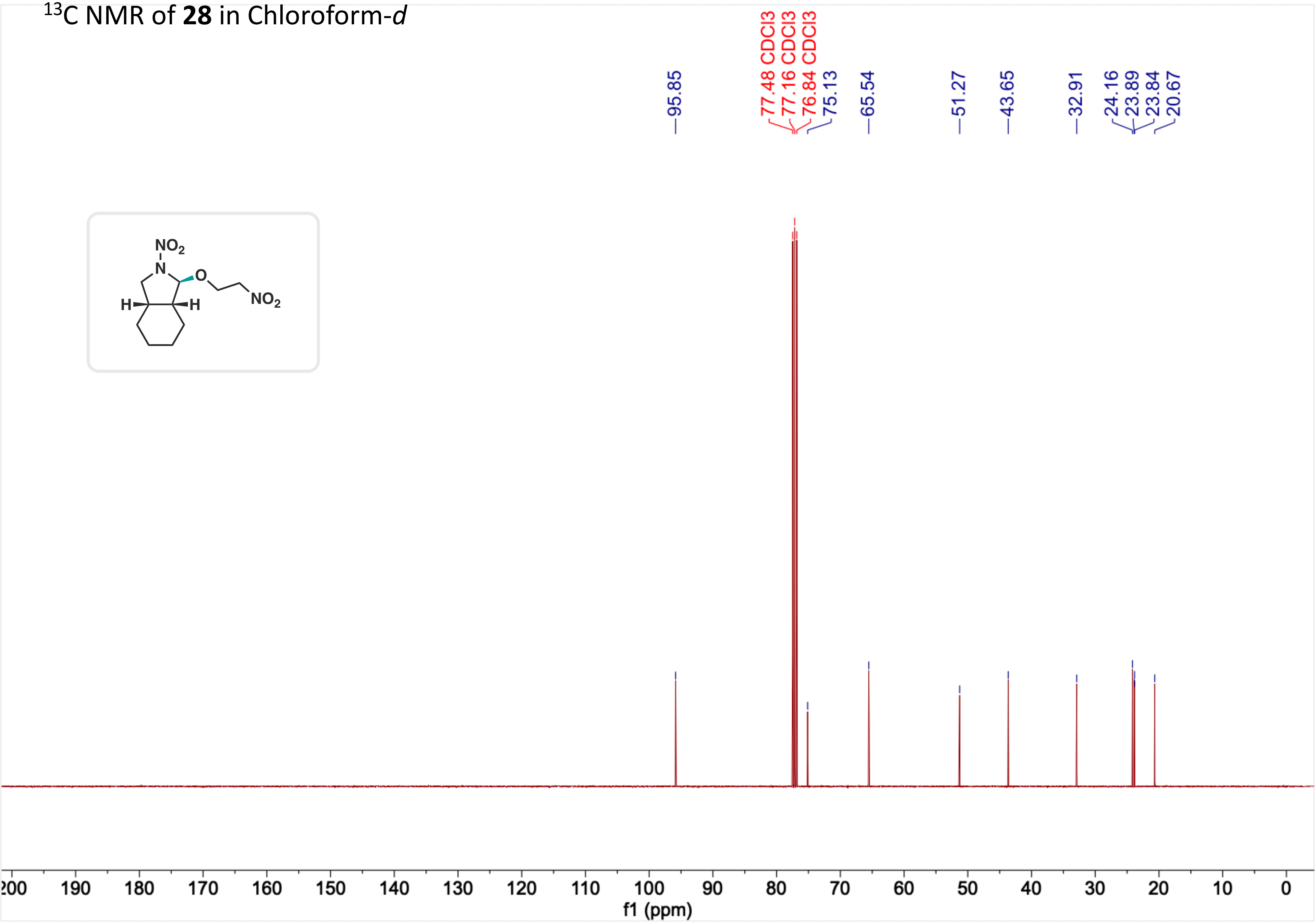

^{1}H NMR of **34** in Chloroform-*d*

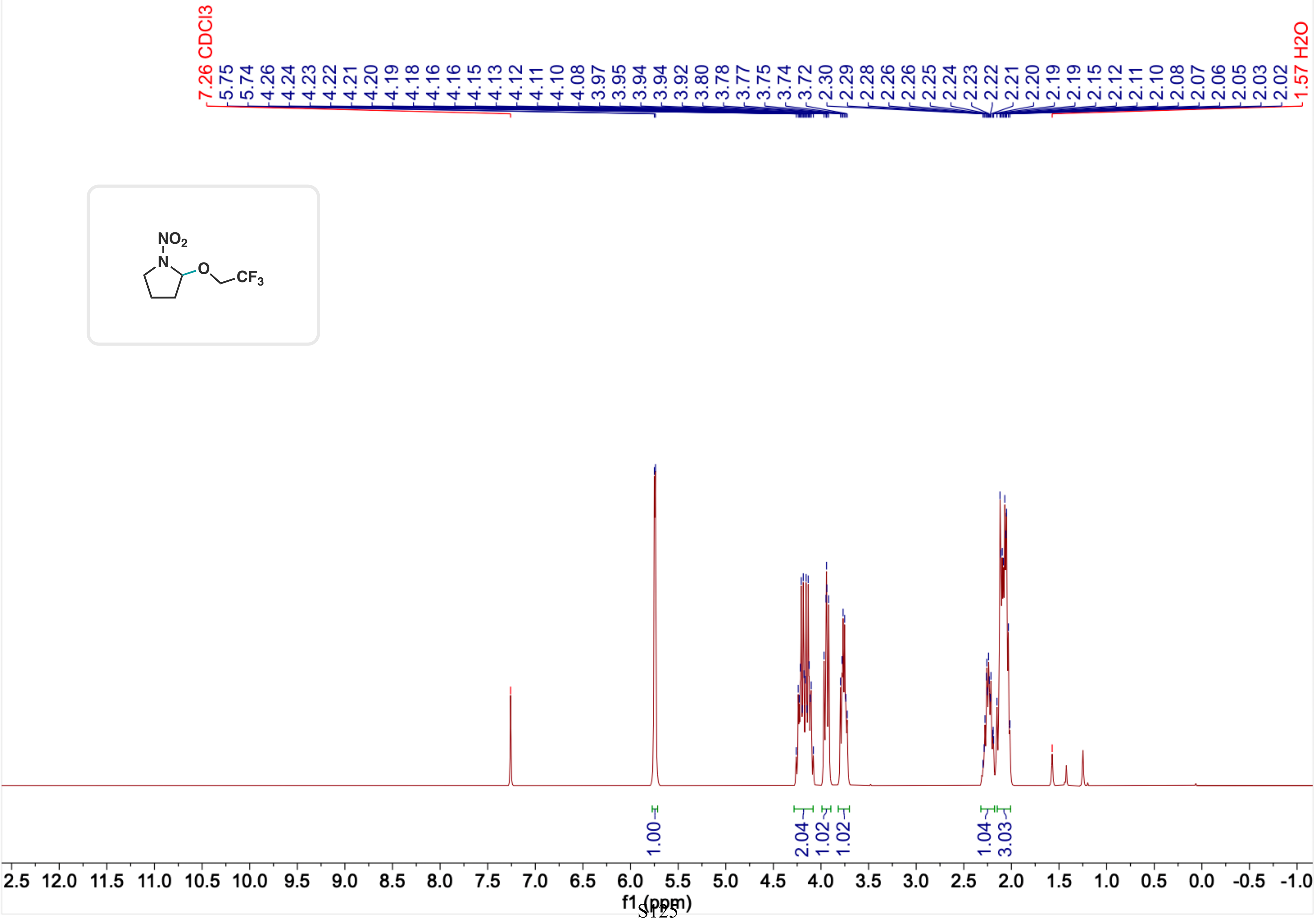

^{19}F NMR of **34** in Chloroform-*d*

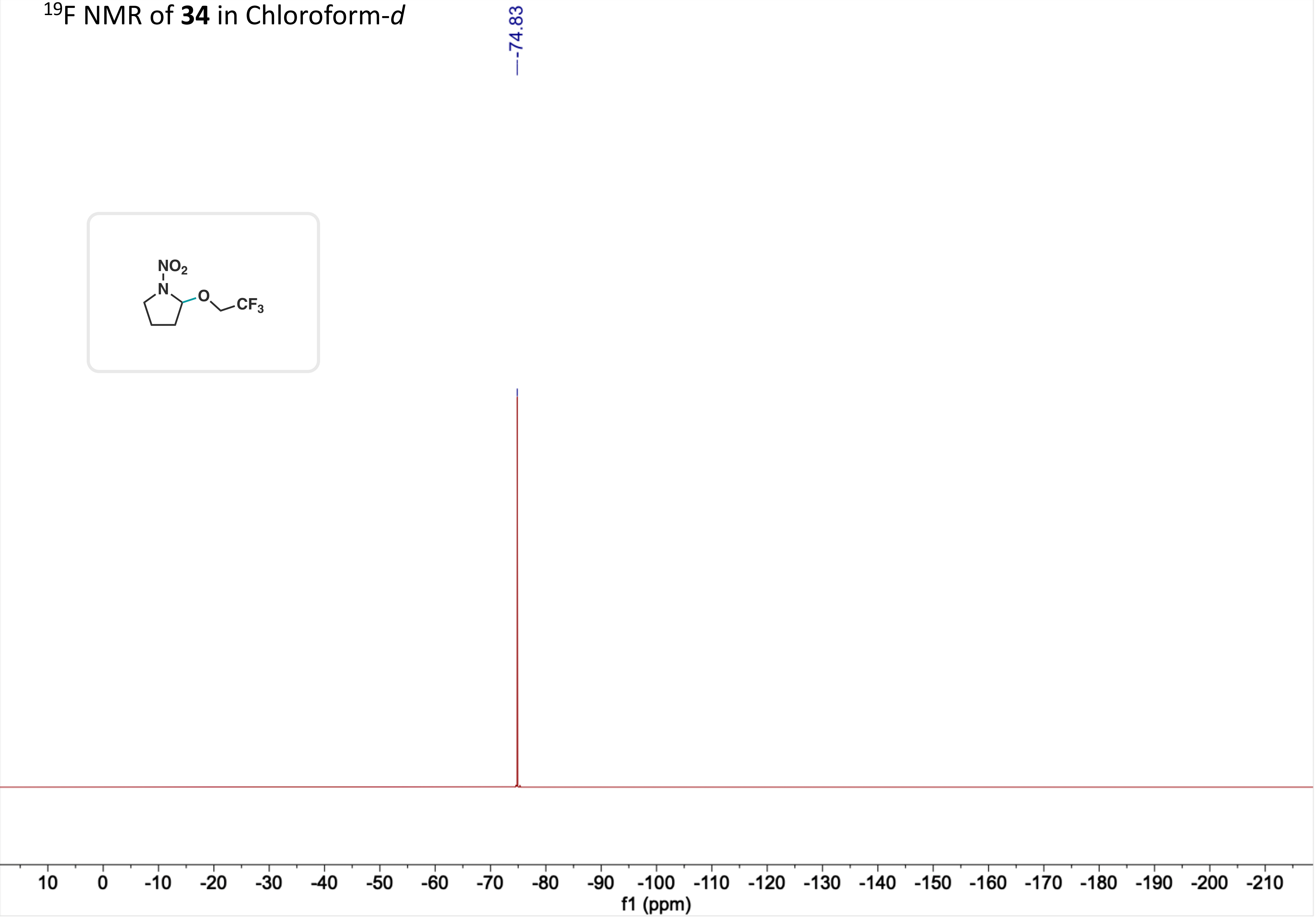

^{13}C NMR of **34** in Chloroform-*d*

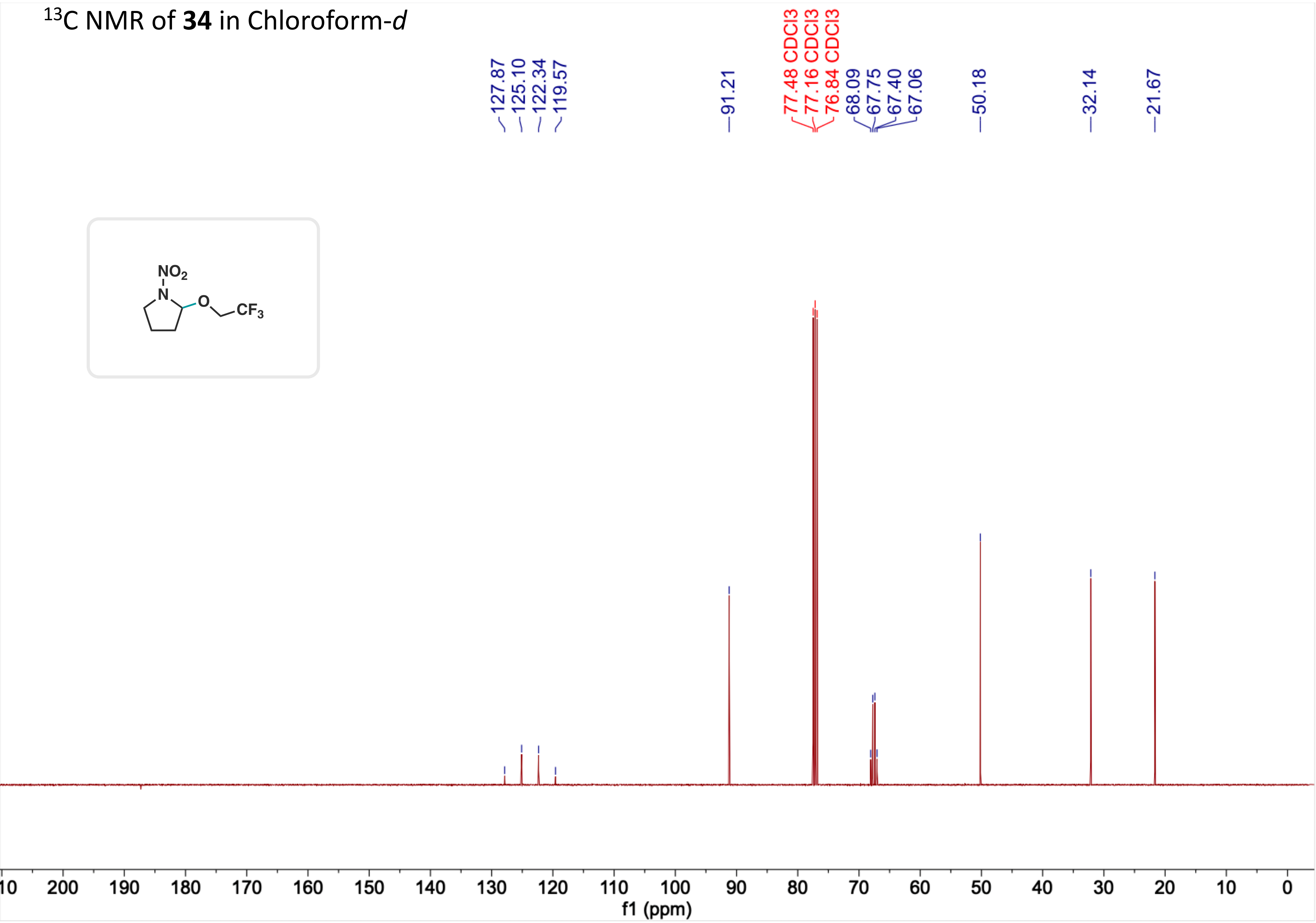

^{1}H NMR of **V-1** in Chloroform-*d*

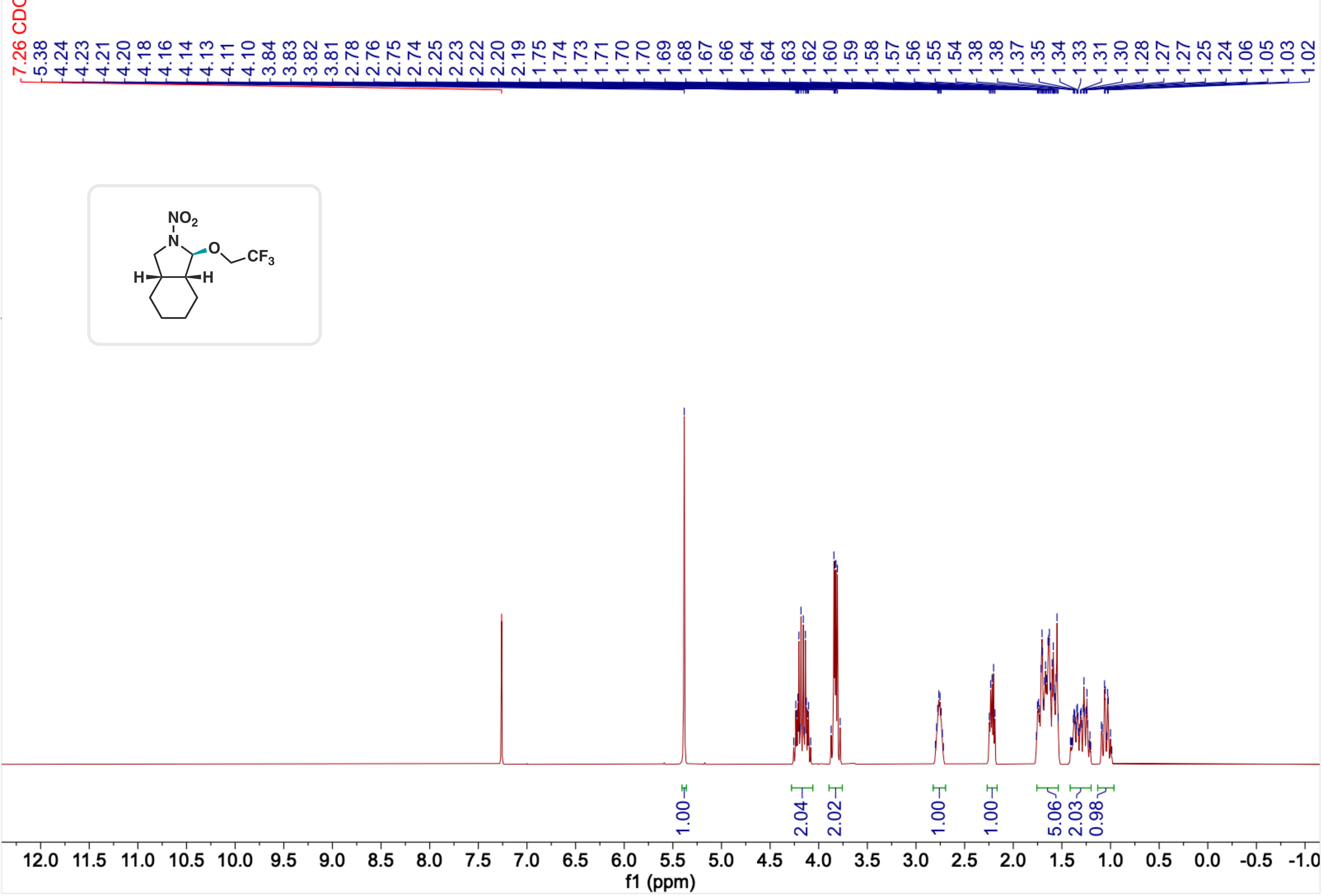

^{19}F NMR of **V-1** in Chloroform-*d*

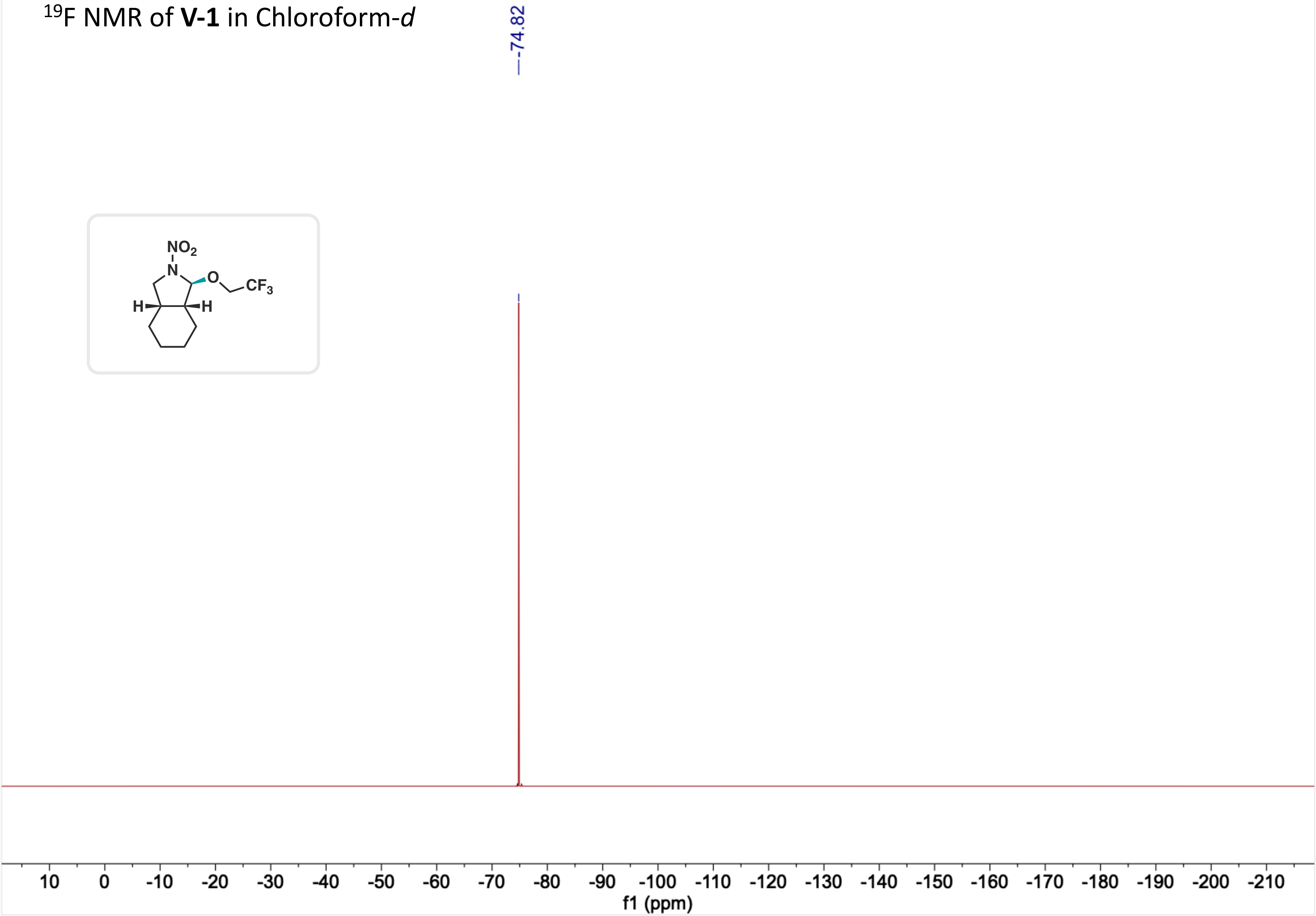

^{13}C NMR of **V-1** in Chloroform-*d*

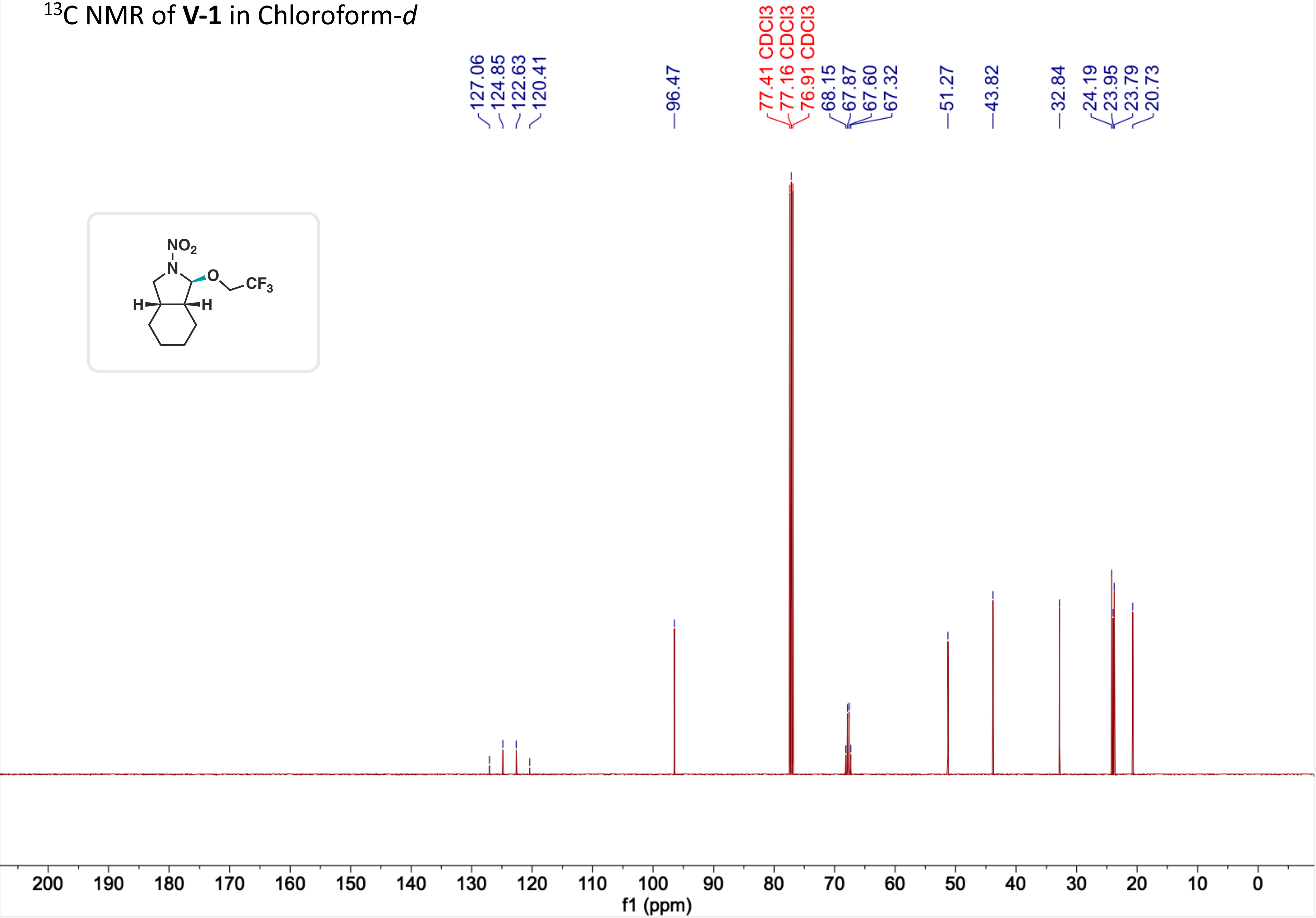

^{1}H NMR of **29** in Acetone-d_6

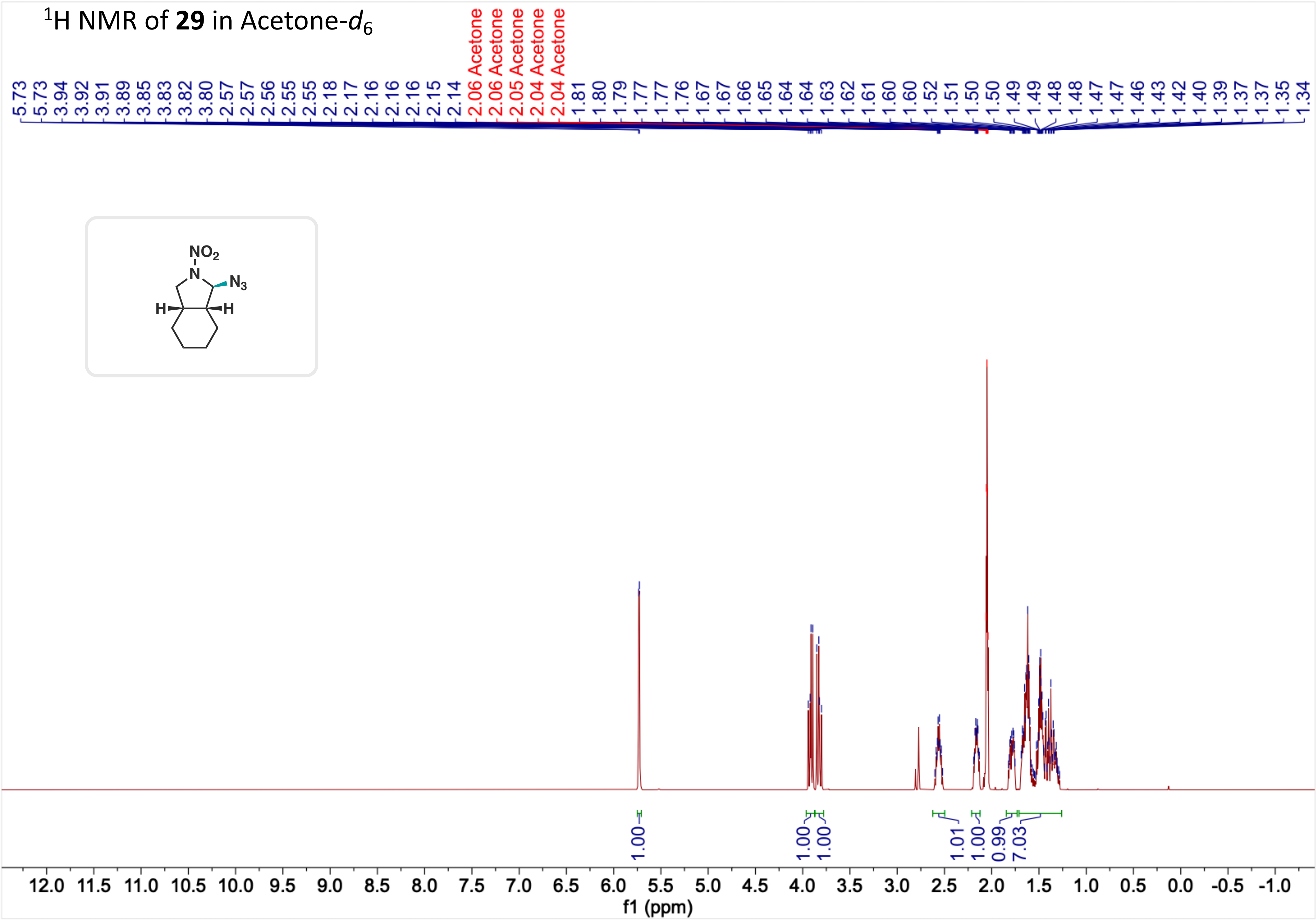

^{13}C NMR of **29** in Acetone-d_6

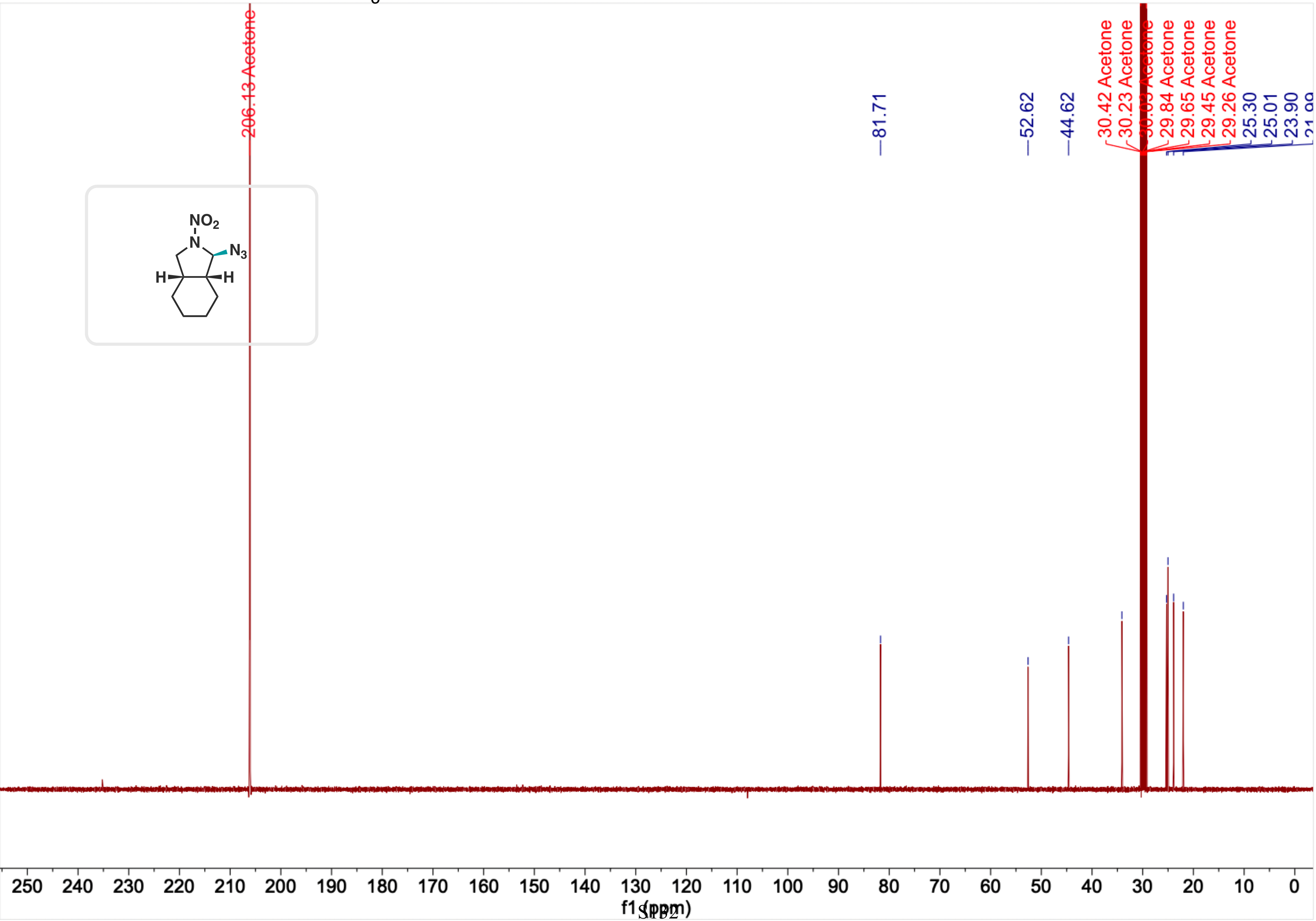

^{1}H NMR of **VII-1** in Chloroform-*d*

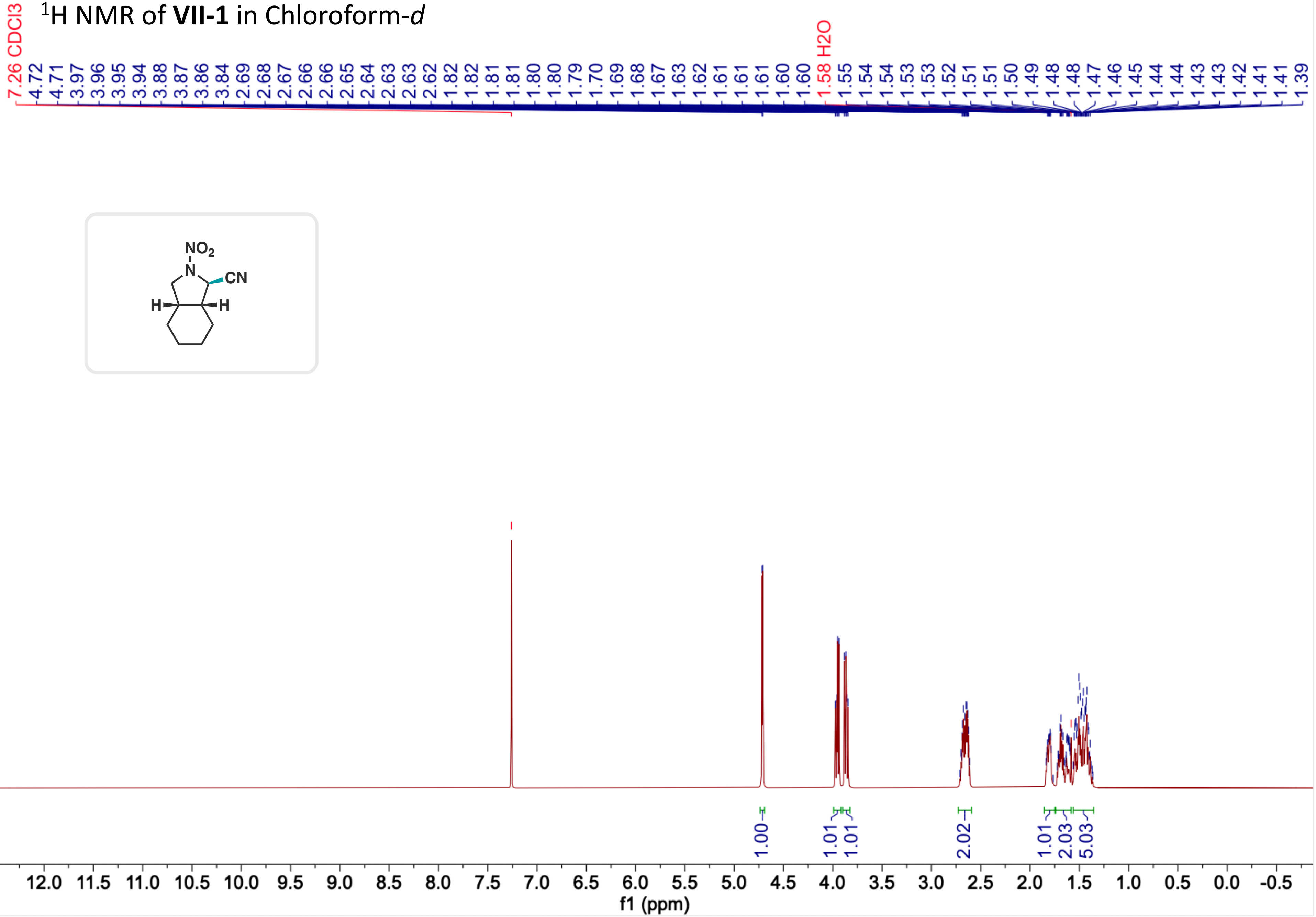

^{13}C NMR of **VII-1** in Chloroform-*d*

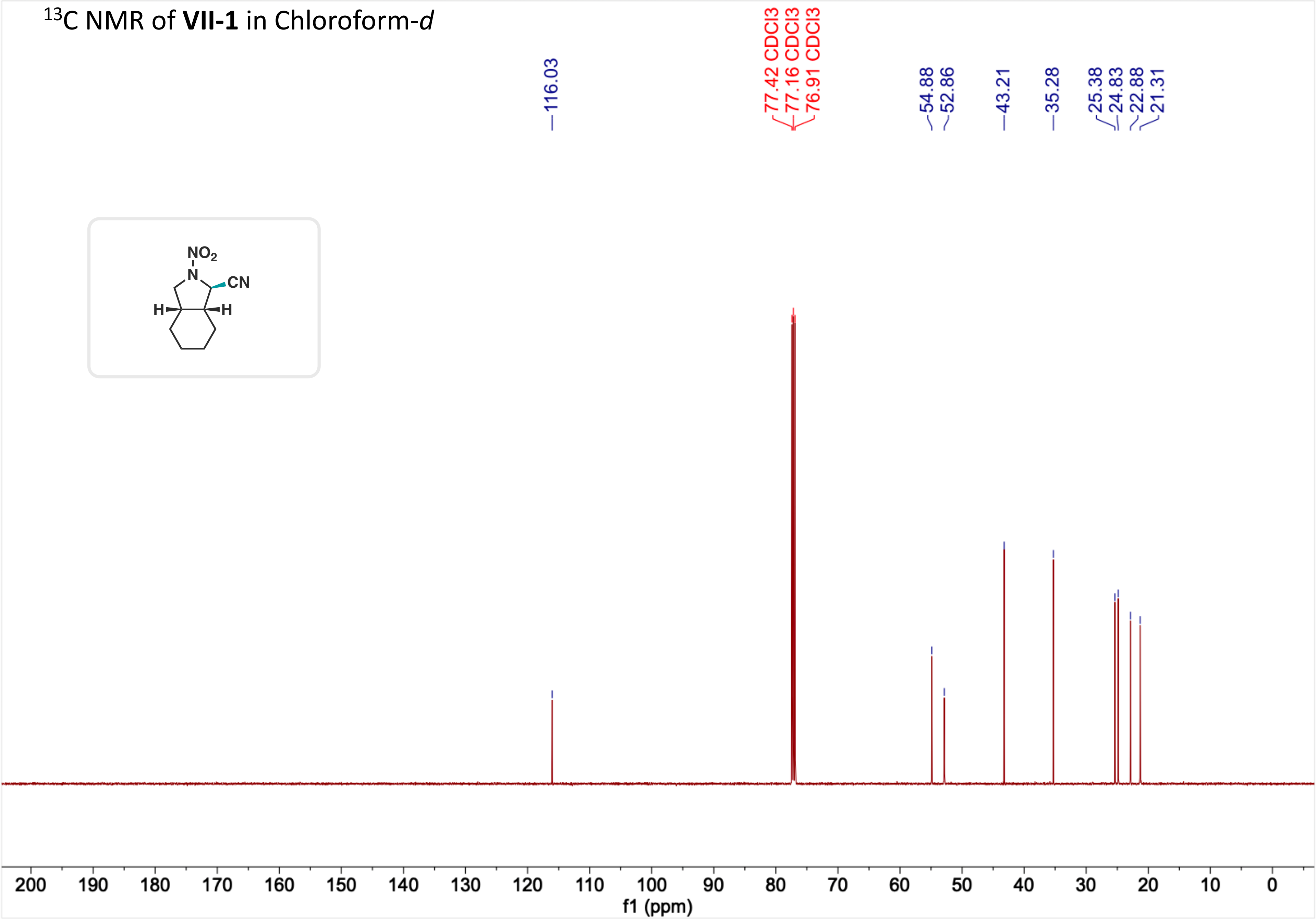

Crude ^{1}H NMR of **30** in Chloroform-*d*

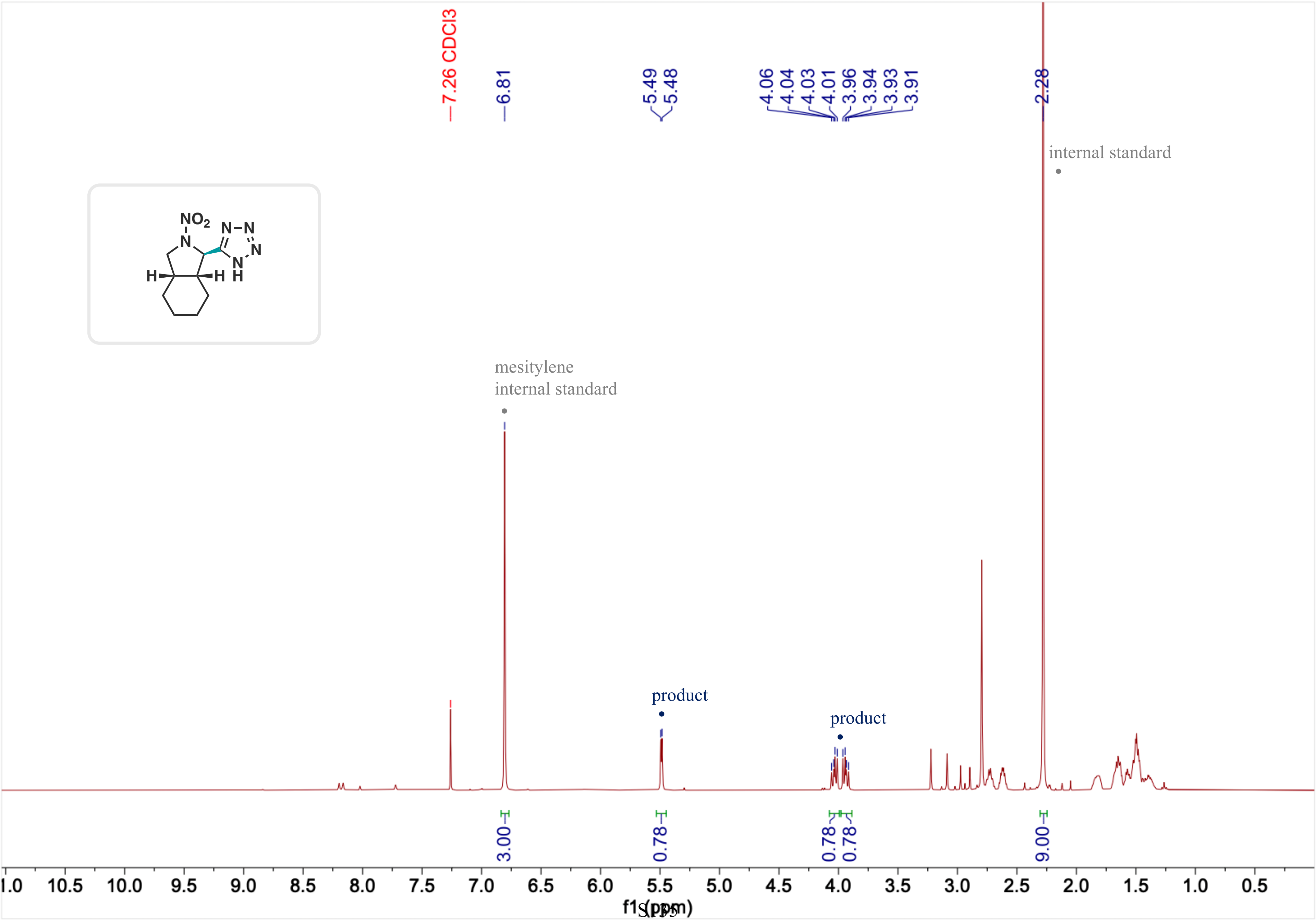

Crude ^{1}H NMR of **30** in Chloroform-*d*

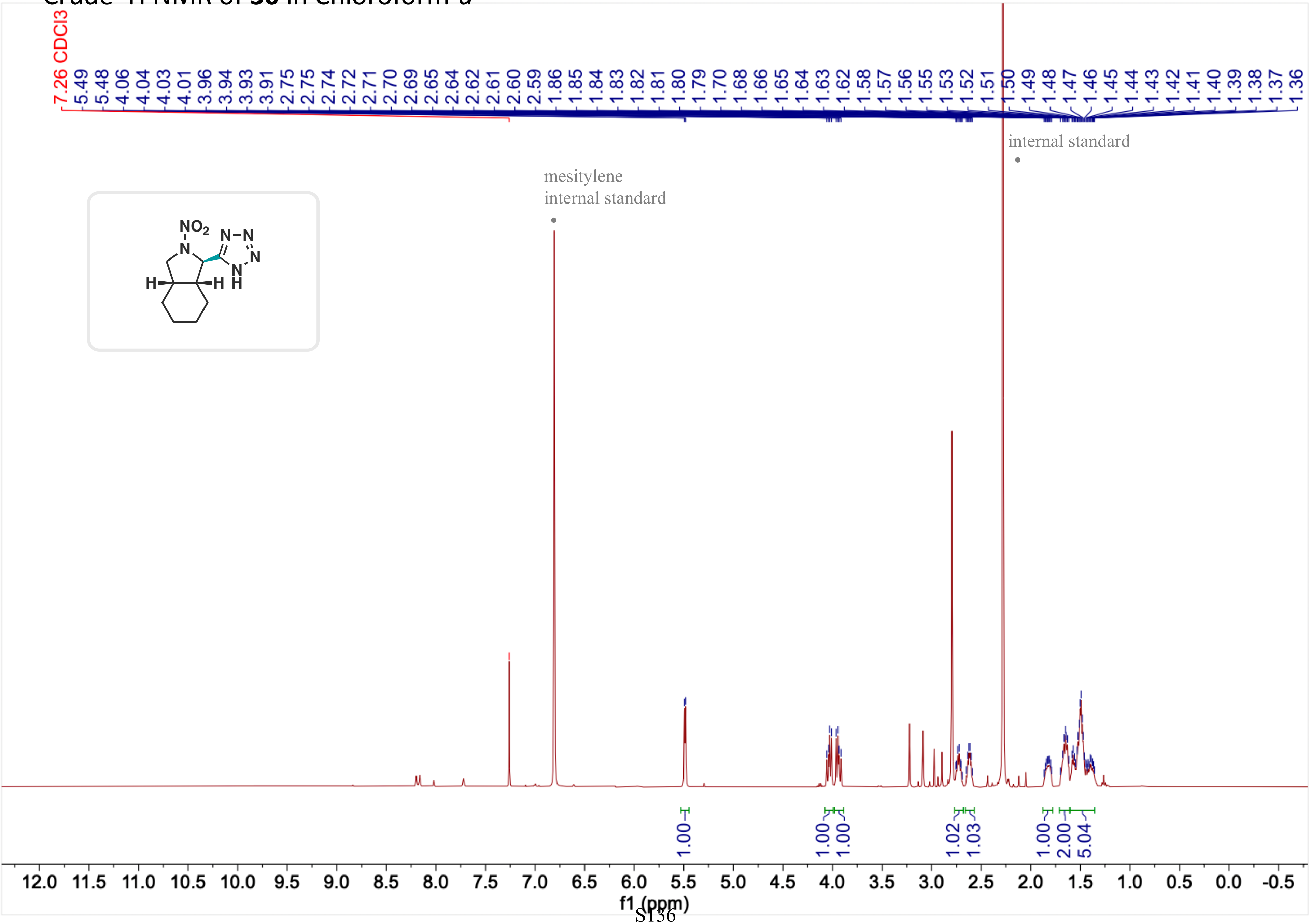

Crude ^{13}C NMR of **30** in Chloroform-*d*

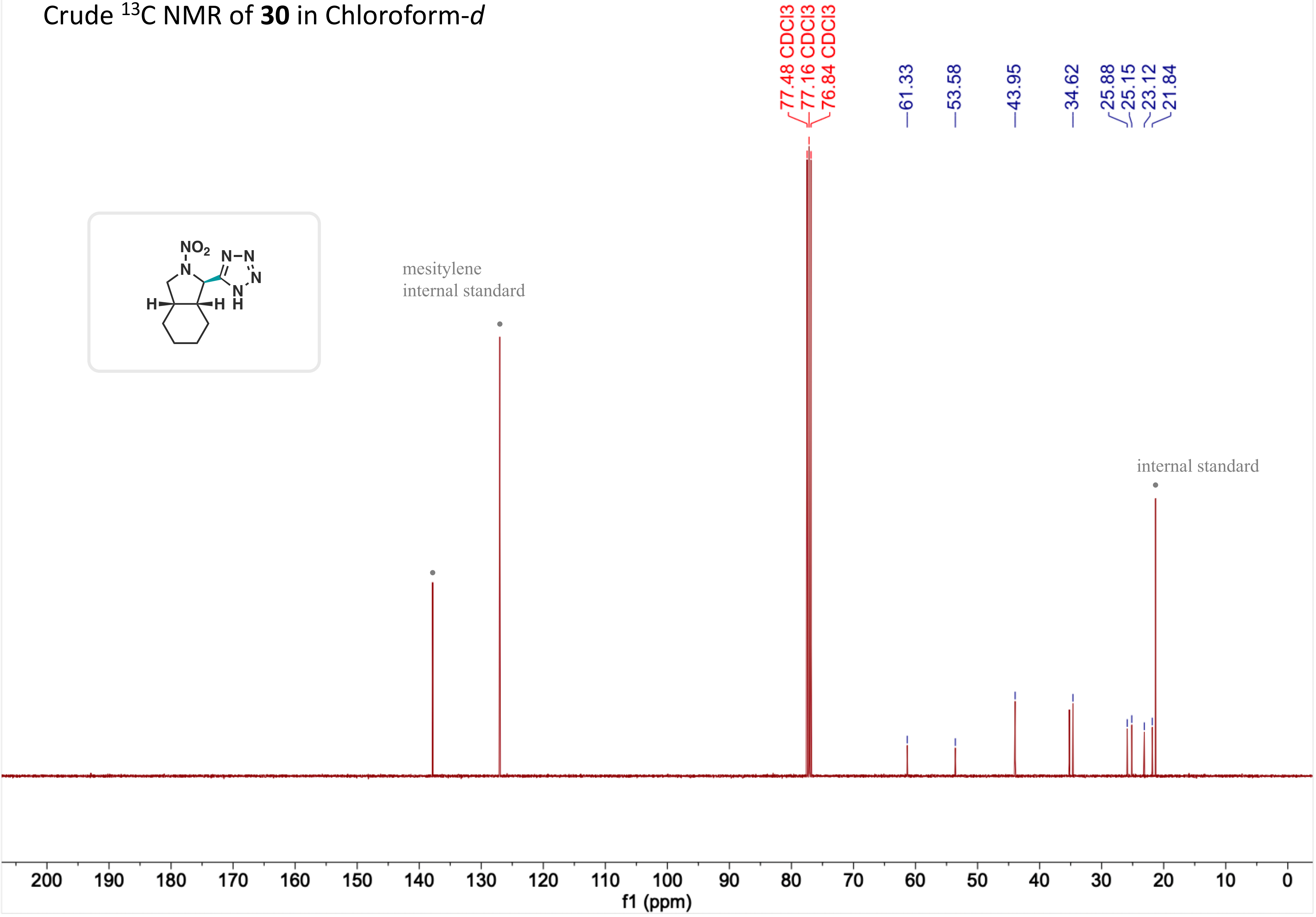

^{1}H-^{13}C HMBC NMR of **30** in Chloroform-*d*

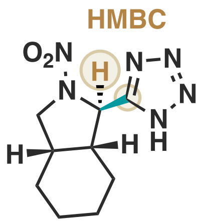

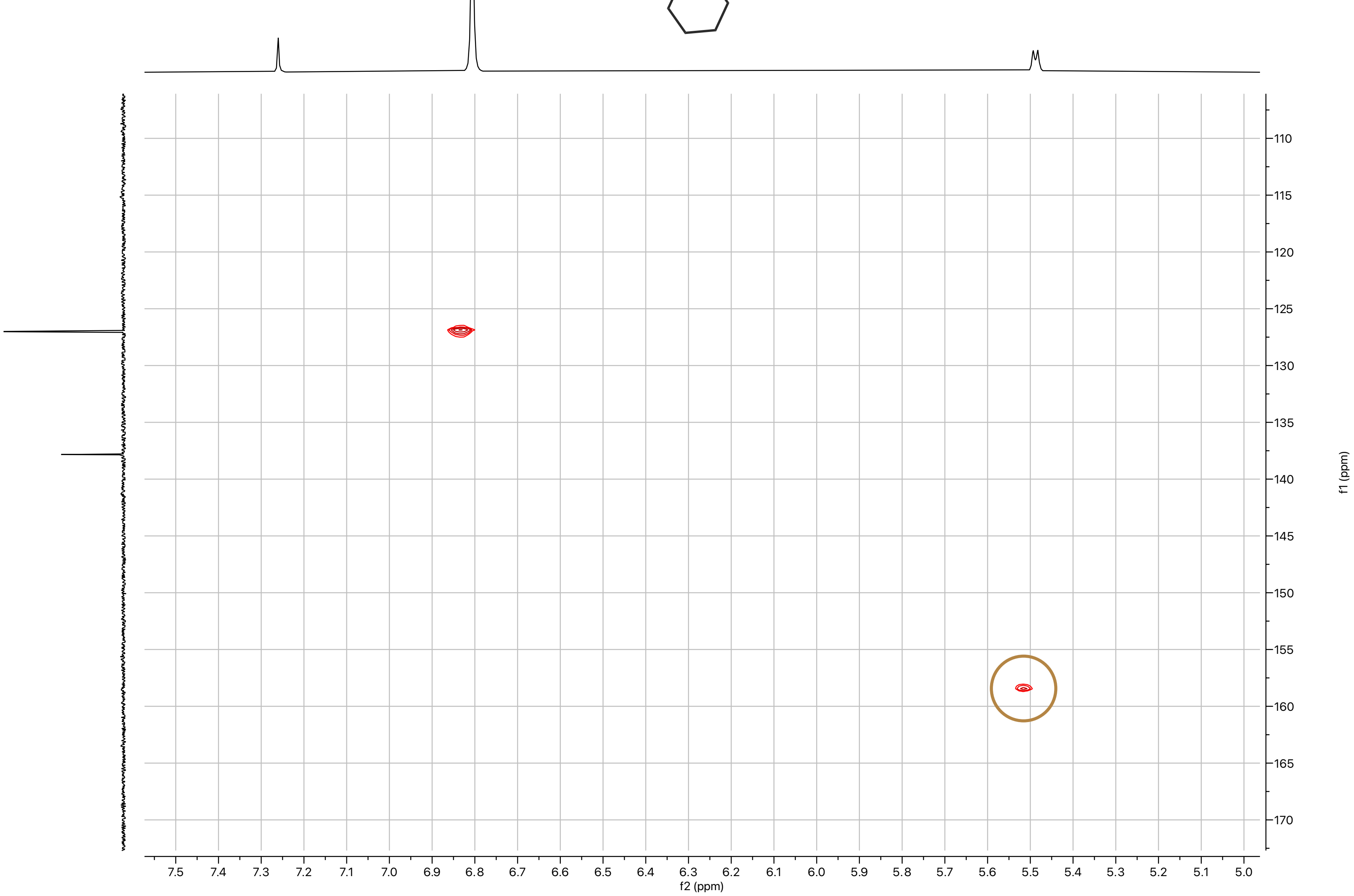

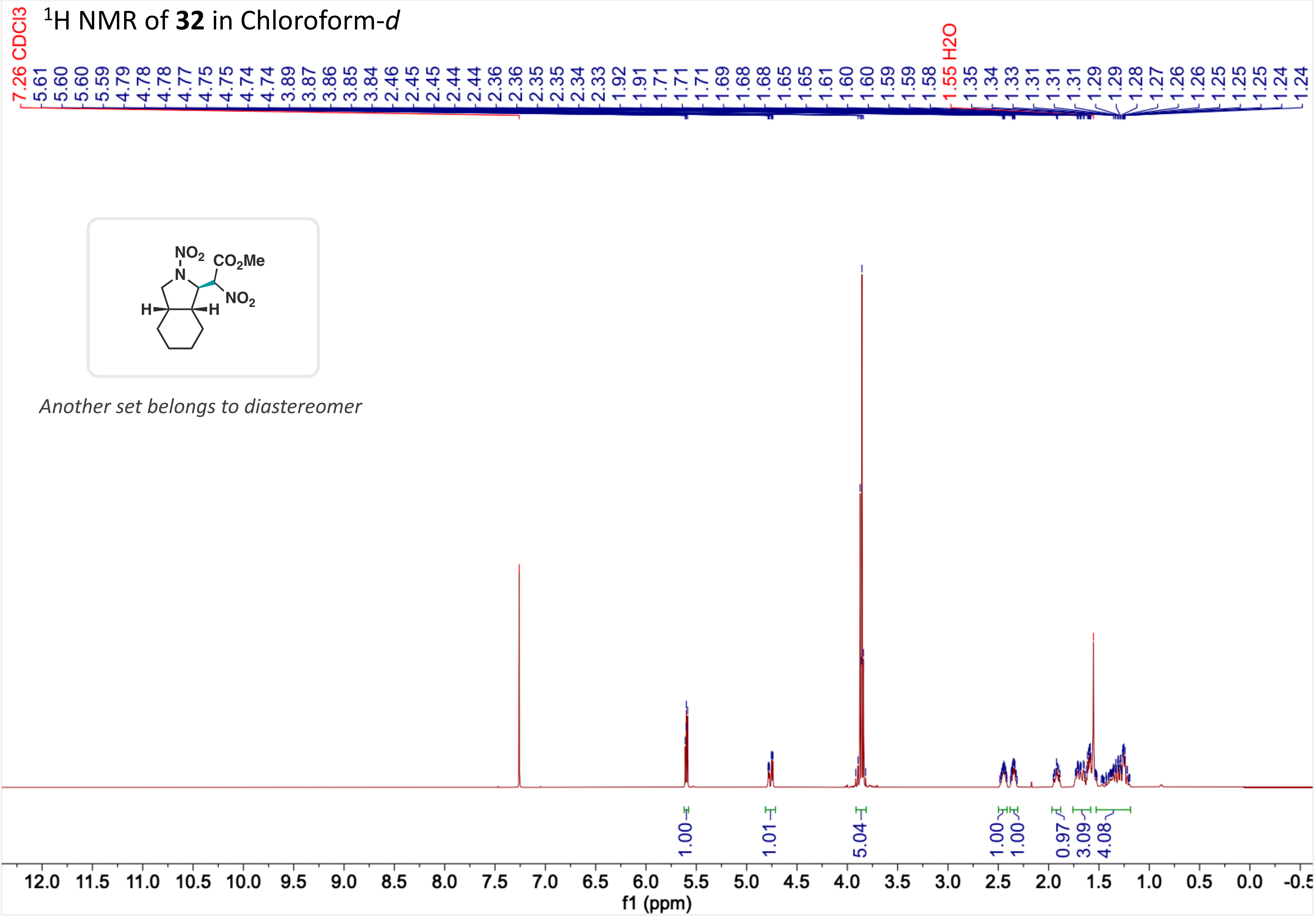

^{1}H NMR of **32** in Chloroform-*d*

^{13}C NMR of **32** in Chloroform-*d*

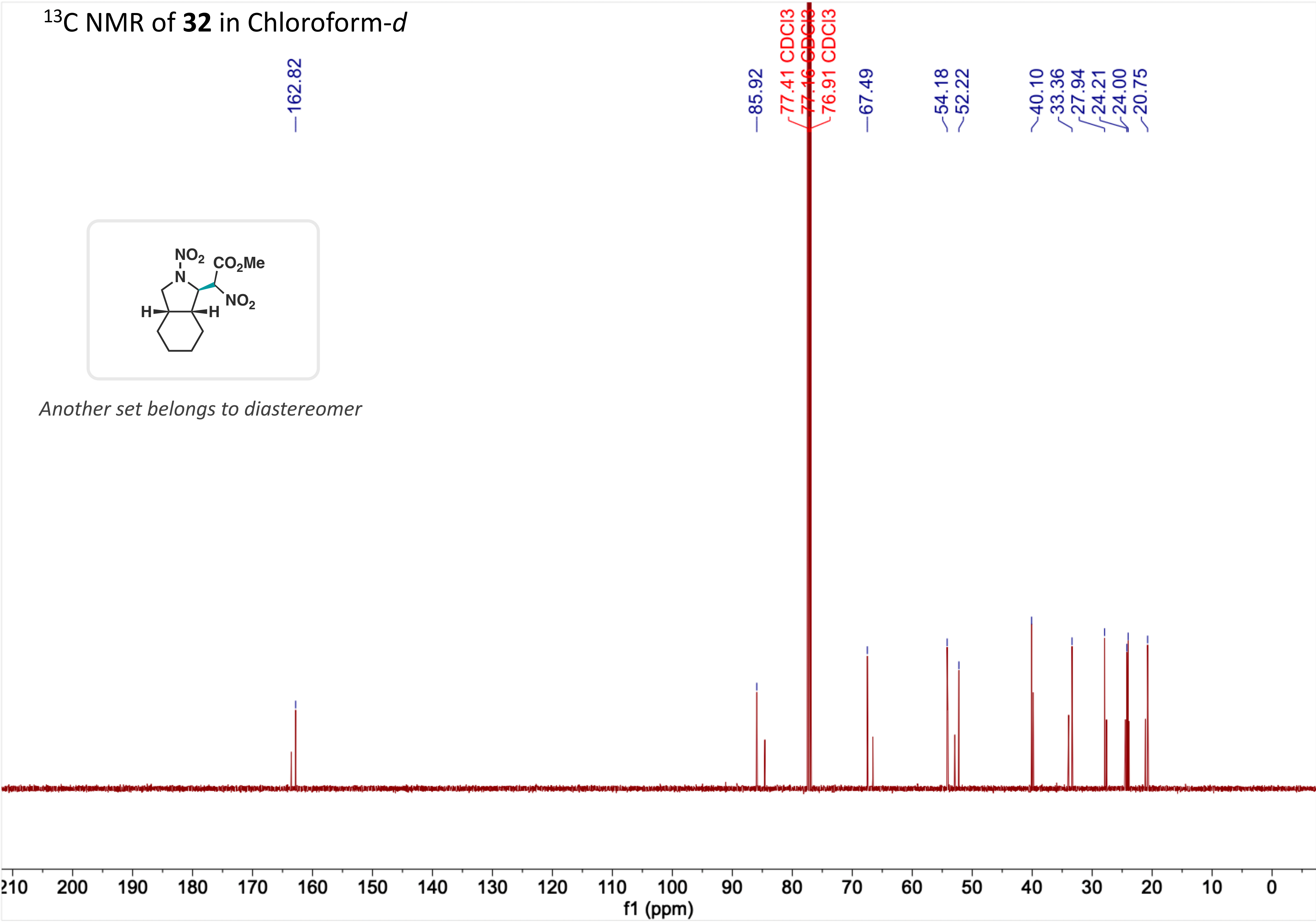

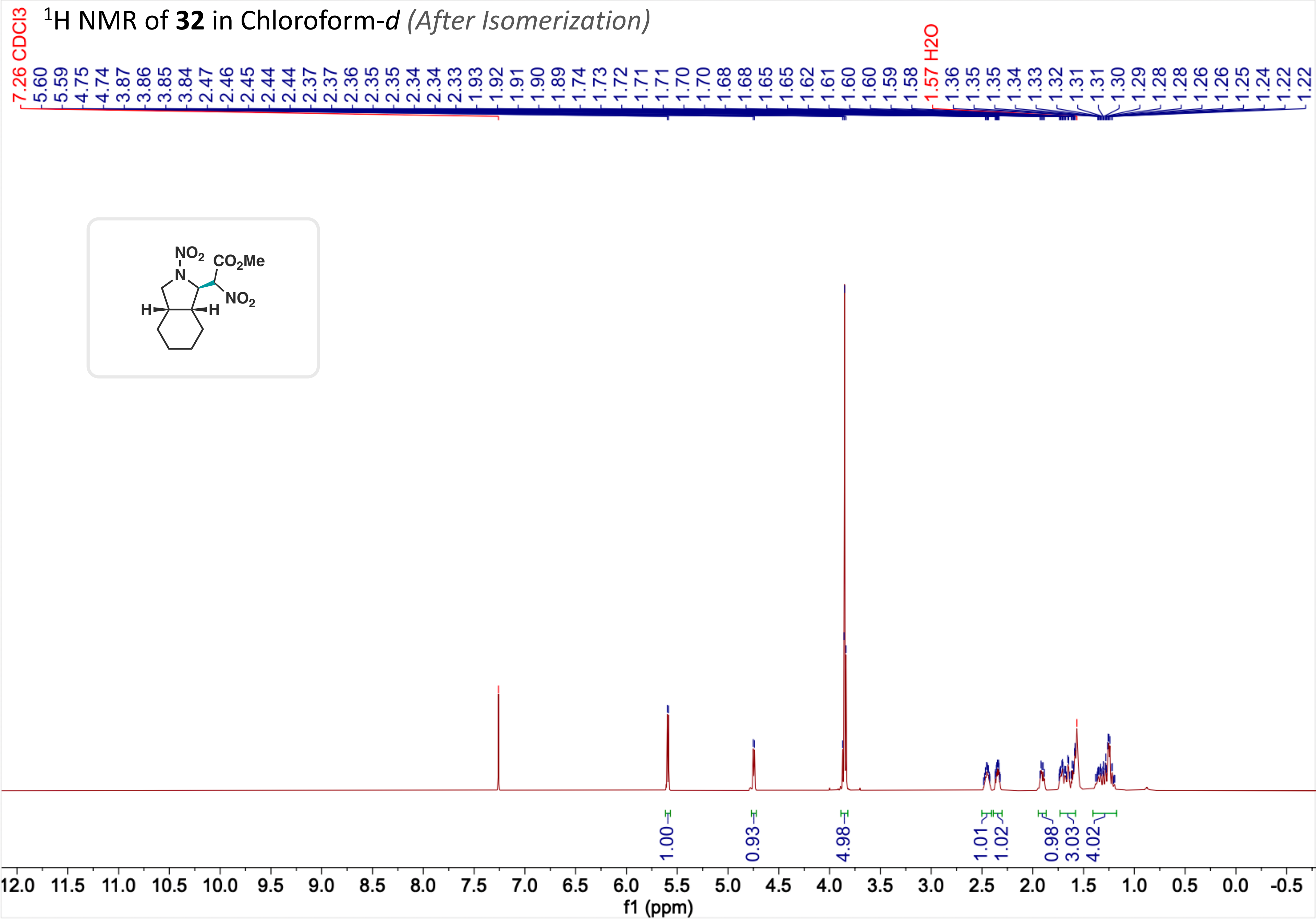

1H NMR of 32 in Chloroform-d (After Isomerization)
7.26 CDCl3
5.60
5.59
4.75
4.74
3.87
3.86
3.85
3.84
2.47
2.46
2.45
2.44
2.44
2.37
2.37
2.36
2.35
2.35
2.34
2.34
2.33
1.93
1.92
1.91
1.90
1.89
1.74
1.73
1.72
1.71
1.71
1.70
1.70
1.68
1.68
1.65
1.65
1.62
1.61
1.60
1.60
1.59
1.58
1.57 H2O
1.36
1.35
1.35
1.34
1.33
1.32
1.31
1.31
1.30
1.29
1.28
1.28
1.26
1.26
1.25
1.24
1.22
1.22
NO2
N
CO2Me
NO2
H
H
1.00
0.93
4.98
1.01
1.02
0.98
3.03
4.02
12.0 11.5 11.0 10.5 10.0 9.5 9.0 8.5 8.0 7.5 7.0 6.5 6.0 5.5 5.0 4.5 4.0 3.5 3.0 2.5 2.0 1.5 1.0 0.5 0.0 -0.5
f1 (ppm)

^{13}C NMR of **32** in Chloroform-*d* *(After Isomerization)*

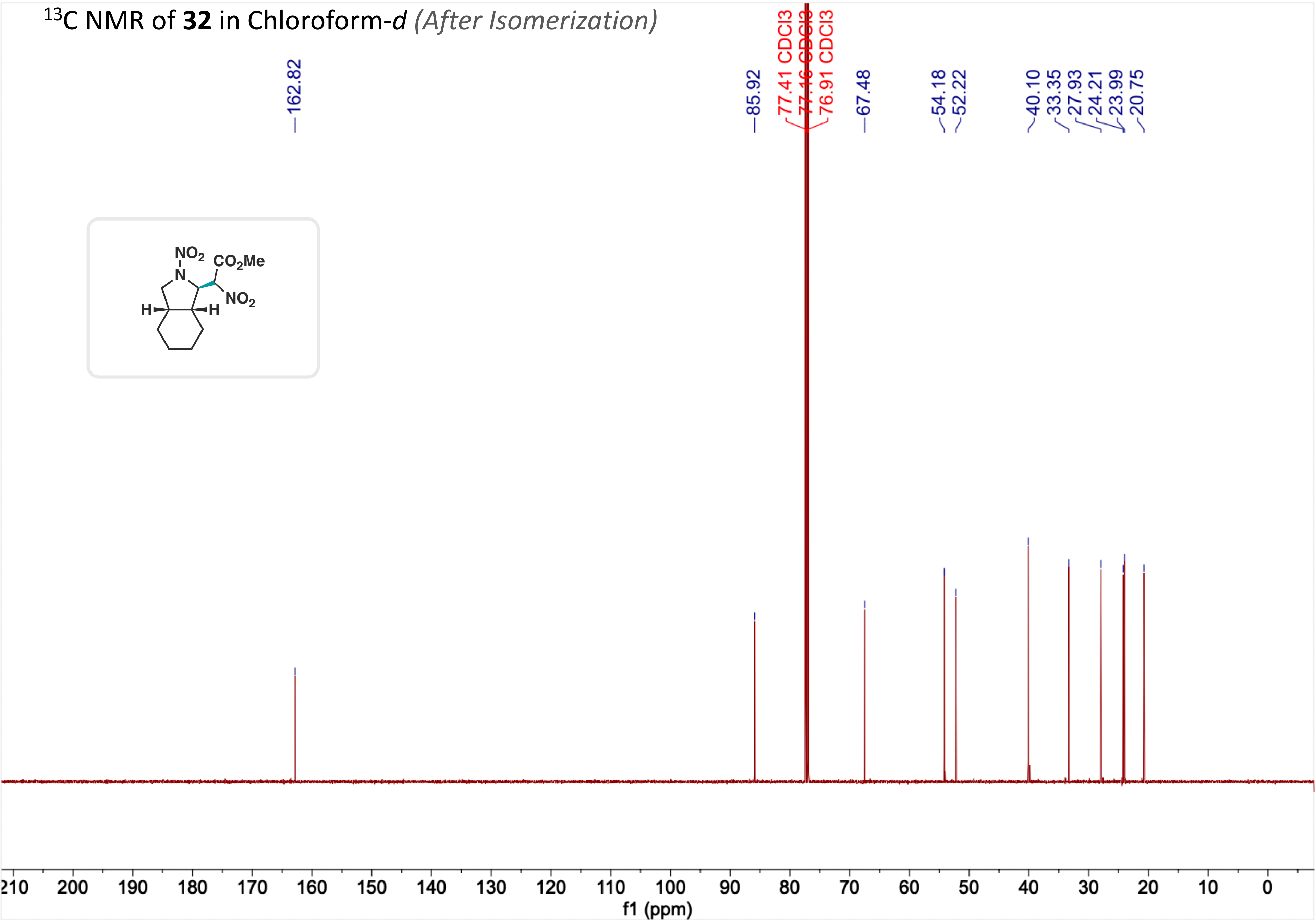

Supplement: Supplementary file 1 — Supporting Information [file ANIE-64-e202515252-s001.pdf]
